# Why are male malaria parasites in such a rush? Sex-specific evolution and host–parasite interactions

**DOI:** 10.1093/emph/eos003

**Published:** 2012-11-26

**Authors:** Shahid M. Khan, Sarah E. Reece, Andrew P. Waters, Chris J. Janse, Szymon Kaczanowski

**Affiliations:** ^1^Leiden Malaria Research Group, Department of Parasitology, LUMC, Albinusdreef 2, 2333 ZA Leiden, The Netherlands; ^2^Centre for Immunity, Infection and Evolution, Institutes of Evolution, Infection and Immunity, School of Biological Sciences, University of Edinburgh, Edinburgh EH9 3JT, UK; ^3^Division of Infection and Immunity, Institute of Biomedical Life Sciences & Wellcome Centre for Molecular Parasitology, University of Glasgow, Glasgow G12 8TA, UK; ^4^Department of Bioinformatics, Institute of Biochemistry and Biophysics, Polish Academy of Sciences, Pawinskiego 5a, 02-106 Warszawa, Poland.

**Keywords:** sex-specific selection, *Plasmodium*, host–parasite coevolution, gene expression

## Abstract

**Background:** Disease-causing organisms are notorious for fast rates of molecular evolution and the ability to adapt rapidly to changes in their ecology. Sex plays a key role in evolution, and recent studies, in humans and other multicellular organisms, document that genes expressed principally or exclusively in males exhibit the fastest rates of adaptive evolution. However, despite the importance of sexual reproduction for many unicellular taxa, sex-biased gene expression and its evolutionary implications have been overlooked.

**Methods:** We analyse genomic data from multiple malaria parasite (*Plasmodium*) species and proteomic data sets from different parasite life cycle stages.

**Results:** The accelerated evolution of male-biased genes has only been examined in multicellular taxa, but our analyses reveal that accelerated evolution in genes with male-specific expression is also a feature of unicellular organisms. This ‘fast-male’ evolution is adaptive and likely facilitated by the male-biased sex ratio of gametes in the mating pool. Furthermore, we propose that the exceptional rates of evolution we observe are driven by interactions between males and host immune responses.

**Conclusions:** We reveal a novel form of host–parasite coevolution that enables parasites to evade host immune responses that negatively impact upon fertility. The identification of parasite genes with accelerated evolution has important implications for the identification of drug and vaccine targets. Specifically, vaccines targeting males will be more vulnerable to parasite evolution than those targeting females or both sexes.

## Abstract (precis)

Host immunity selects for the rapid, adaptive, evolution of genes expressed exclusively in male malaria parasites. Analyses of genomic and proteomic data across multiple malaria species reveals rapid adaptive evolution of genes with sex-biased expression in unicellular parasites. Accelerated evolution enables parasites to cope with host immune responses that reduce fertility.

## INTRODUCTION

Explaining variation in rates of molecular evolution is fundamental to evolutionary biology and is especially important for organisms that respond rapidly to shifts in the environment, such as disease-causing parasites and microbes that perform ecosystem services. In multicellular organisms, genes expressed principally or exclusively in one sex evolve at an accelerated rate compared with genes expressed in both sexes [1]. In particular, rapid adaptive evolution occurs in male-specific genes whose expression is associated with tissues and traits that underpin mating success and fertility [2–5]. This solution, to the problem of different optimal phenotypes in males and females, has been extensively documented for multicellular taxa [6, 7], but whether males and females of dioecious unicellular taxa are also subject to different, or opposing, selection pressures has been overlooked. This is surprising since sexual reproduction is an obligate feature of the life cycles of many parasite species.

There is increasing interest in the reciprocal approach of using an evolutionary framework to understand the biology of parasites and exploiting the novel experimental opportunities provided by these species to test the generality of evolutionary theories. For example, blocking the fertility of gametes to prevent sexual reproduction is a priority target for the development of transmission-blocking interventions against malaria [8–12]. Understanding how selection shapes the evolution of parasite sexes and mating systems is therefore central to identifying the most ‘evolution-proof’ transmission-blocking targets and implementation strategies. For evolutionary biology, distinguishing between the footprints of natural and sexual selection pressures and identifying the biological mechanisms that drive the accelerated evolution observed in male-biased genes has proved difficult. For example, most males produce large numbers of sperm, and so multiple rounds of mitosis could result in a higher mutation rate in males than females. Alternatively, in mammals, genes on the Y chromosome could evolve rapidly simply because the lack of repair during recombination facilitates mutation [1], but X-linked genes could also evolve quickly because recessive mutations are exposed to selection in males [13, 14]. Using unicellular organisms such as malaria (*Plasmodium*) parasites overcomes many of these problems of inference because all life cycle stages, except for a brief zygote phase, are haploid, and sex determination does not involve sex chromosomes or regions of contiguous genes [15]. Therefore, sexual dimorphism in *Plasmodium* is generated solely by differential gene expression, which is observed throughout the genome (Fig. S1). Depending on population structure, the ratio of male to female gametes may be male-biased in *Plasmodium,* but because gametogenesis involves only three more rounds of mitosis for each male compared with each female, the opportunity for mutational bias to result in fast-male evolution is much lower than for many multicellular organisms.

Here, we integrate advances in genomics and proteomics to examine the evolutionary forces on genes encoding features of *Plasmodium* sexual cells. We compare divergence between closely related pairs of species and reveal rapid, adaptive evolution of male-biased genes in *Plasmodium.* The consequences of host–parasite interactions for the evolution of parasite sexual cells are poorly understood, but our analyses suggest a role for host immune responses in driving ‘fast-male’ evolution. Our findings demonstrate the necessity of an evolutionary framework for the development of medical interventions that disrupt disease transmission by preventing parasites from mating.

## METHODOLOGY

We take advantage of proteomic data for malaria parasites [16] to analyse the rate of change of genes that are expressed in different life cycle stages, across several *Plasmodium* species. First, we test whether the rapid evolution of genes exclusively expressed in males occurs in unicellular parasites. Second, having found that male-biased genes evolve more rapidly than female-biased genes, we asked whether selective forces resulting from host–parasite interactions could influence this sex-specific evolution. Third, we investigated whether the rapid evolution of male-biased genes is due to the relaxation of selection constraints or adaptive evolution.

### Generation of data sets

*Plasmodium* parasites replicate asexually in the circulation of the host and must undergo a round of sexual reproduction in the mosquito vector to be transmitted [17, 18]. A small percentage of asexual stages differentiate into sexual stages, termed gametocytes. Male and female gametocytes differentiate into male and female gametes as soon as they are taken up in a mosquito blood meal. Within 10–12 min, each male gametocyte undergoes three rounds of mitosis, producing up to eight gametes, and each female gametocyte differentiates into a single gamete.

**Figure 1.**
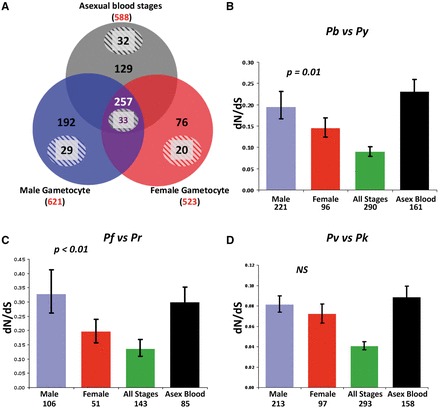
Stage-specific rates of evolution. Estimated as *d*_N_/*d*_S_, for genes expressed in sexual (male, female) and asexual blood stage malaria parasites. (**A**) Pattern of ‘stage-specific’ expression of genes based on proteomes of *P. berghei* sexual and asexual blood stages. Red represents the total number of proteins identified in each proteome. Proteins are subdivided into putative membrane and non-membrane proteins. Numbers inside the circles refer to the number of putative non-membrane proteins (solid background) and putative membrane proteins (shaded background) detected exclusively in proteomes of Males, Females, Asexual Stages or detected in all three stages (All Stages). (**B–D**) Rates of evolution determined by comparing genes from each closely related pair of *Plasmodium* species. The genes used for this analysis are the orthologs of the *P. berghei* genes identified in (A). *P. berghei* and *P. yoelii* (B); *P. falciparum* and *P. reichenowi* (C); *P. vivax* and *P. knowlesi* (D).

Male and female gametocytes each express a specific and distinct set of proteins [16]. Using the proteomes of males, females and asexual blood stages, we distinguished *Plasmodium berghei* genes based on their expression [unambiguous identification of two or more high scoring (MASCOT score >15) peptides] in different stages during intra-erythrocytic development. We classified genes (Fig. 1A; Table S1, PB) as having male-biased expression (termed Male), female-biased expression (Female), expression in asexual and gametocyte stages (All Stages) and expression in asexual blood stages but not in gametocytes (Asexual Blood).

We identified orthologs of the *P. berghei* genes using reciprocal BLAST for the following species: *Plasmodium yoelii, Plasmodium vivax, Plasmodium knowlesi, Plasmodium reichenowi* and *Plasmodium falciparum* (Table S1, PF and PV). We assume stage specificity of gene expression is similar across *Plasmodium* species, as previously suggested for sporozoites [19], asexual stages [20] and gametocytes [21]. We also subdivided each of the four categories into putative membrane and non-membrane proteins, based on the presence of predicted signal peptides, transmembrane domains and glycosylphosphatidylinositol anchors (Table S1, PB, PF and PV).

### *Plasmodium berghei* gene models

The gene models deposited in PlasmoDB are constantly being improved, and during the preparation of this article, a new release of the *P. berghei* genome became available. Therefore, we have adopted the naming convention based on the 8X *P. berghei* ANKA release 2010-06-01 (available at http://plasmodb.org/plasmo/showXmlDataContent.do; jsessionid=3A2D8DAC94289AB19ADA50C7810ADCE?name=XmlQuestions.DataSources). The annotation for *P. berghei* was obtained, with permission, from the Pathogen Sequencing Unit at the Wellcome Trust Sanger Institute.

### Protein selection

We used proteins identified by Khan *et al.* [16] and describe the mapping of these data onto the new release for *P. berghei* in Table S6. We assume that membrane proteins contain more than one TM segment or signal peptide, predicted using hidden Markov models (http://www.cbs.dtu.dk/services/SignalP/) [22]. Orthologous proteomes were predicted using reciprocal BLAST with e-value <1 × 10^−6^ [23]. Sets of proteins containing epitopes were obtained from PlasmoDB [24] as were values of *d*_N_, *d*_S_ and *p*_N_/*p*_S_ for the alignment of 3D7 versus Ghana isolates and the other *P. falciparum* strains (IT, DD2 and HB3).

### Codon alignments

We used predicted sequences of *P. berghei* and *P. vivax* and genomes of *P. yoelii* and *P. knowlesi* obtained from PlasmoDB [23]. Genome regions containing orthologs of analysed sequences were predicted using the BLASTN program. Exons of genes were predicted using the sim4 program [25]. Sequences of exons were aligned using the Needelman and Wunsch algorithm [26]. Codons neighbouring the gaps were removed from alignments. All calculations were performed using perl pipeline. Alignments containing stop codons or <60 codons were removed.

### *d*_N_*/d*_S_ analysis

For each expression class, we compared the rate of non-synonymous (*d*_N_) and synonymous (*d*_S_) nucleotide substitutions of the residue encoding sites of the same gene from pairs of closely related malaria parasites (Table S2). We calculated *d*_N_*/d*_S_ between the rodent malaria parasites *P. berghei* versus *P. yoelii,* the human and non-human primate malaria parasites *P. falciparum* versus *P. reichenowi* and *P. vivax* versus *P. knowlesi.* We used these pairs of closely related species to compare the speed of evolution of different protein classes. The evolution of the gene classes we analyse occurred independently in each pair, and our observations are supported across all species pairs. Estimates of *d*_N_ and *d*_S_ were each obtained using PAML version 3.13d, under a codon-based model with average nucleotide frequencies estimated from the data at each codon position. We calculated *d*_N_*/d*_S_ for genes for which the alignment between the two species was longer than 20 codons. For each pair of orthologous genes, the value of *d*_N_/*d*_S_ was estimated from the ratio of maximum likelihood estimates of the number of non-synonymous substitutions per non-synonymous nucleotide site (*d*_N_) and the number of synonymous substitutions per synonymous nucleotide site (*d*_S_). We also investigated whether any gene models used in our analysis are fragments that have been concatenated with others to form larger and more accurate gene models in the 8X *P. berghei* ANKA release. Of the 809 genes analysed, only three (one expressed in All Stages and two expressed in Males) are now predicted to be part of larger genes. Furthermore, for comparison with the results presented in Table S2, PB (for *P. berghei* versus *P. yoelii*), we have repeated the analyses using the new gene models and the results do not differ (Table S6).

### McDonald–Kreitman test

To formally test for a role of positive selection, we used a modified version of the McDonald–Kreitman test [27, 28]. The parameter *α* refers to the fraction of amino acid substitutions driven by positive selection which is estimated from polymorphism and divergence. The effects of positive selection (*α*) can be distinguished from purifying selection and neutrality by comparing divergence (rate of fixation of non-synonymous mutations; *d*_N_*/d*_S_) between species to the observed relative rate of non-synonymous polymorphisms (*p*_N_*/p*_S_) in natural populations within a species. The data for each gene comprise a 2 × 2 table with rows corresponding to putative selected or non-selected sites and columns corresponding to polymorphism or divergence. We applied a maximum likelihood approach using the ‘distribution of fitness effects’ program (DoFE; [27, 28]). The DoFE-all method uses a maximum likelihood approach that maximizes the number of genes that can be analysed (even those with no polymorphism) and does not sum *D*_N_, *D*_S_, *P*_N_ and *P*_S_ values across genes. This is important for selecting genes under positive selection when signals are weak because it also avoids underestimating *α* when mildly deleterious mutations obscure divergence by inflating polymorphism. A negative value for *α* indicates that there are no amino acid substitutions driven by positive selection and that the majority of polymorphisms are removed due to purifying selection.

### Statistical significance

Statistical significance was based on 10 000 mean values (*d*_N_, *d*_S_, *d*_N_/*d*_S_ and *p*_N_/*p*_S_) for two sets bootstrapped independently and than compared. For the McDonald–Kreitman test, we compared frequencies using the DoFE-all method, which returns a value of alpha and 95% credibility intervals.

## RESULTS

### Fast-male evolution in *Plasmodium*

The *P. berghei* versus *P. yoelii* (*P* = 0.01) and *P. falciparum* versus *P. reichenowi* (*P* = 0.001) comparisons revealed that Male genes are evolving significantly faster than Female genes (Fig. 1B and C). A similar trend, although not significant (*P* = 0.11), is observed in the Male versus Female genes in the *P. vivax* and *P. knowlesi* comparison (Fig. 1D). Values for *d*_N_ and *d*_S_ for all comparisons are plotted in Supplementary Fig. S2, and we obtain qualitatively similar results when analysing *d*_N_ alone. Interestingly, in all comparisons (all *P* > 0.08), Male genes show high average *d*_N_*/d*_S_ values that are comparable to those of Asexual Blood genes. Proteins of Asexual Blood stages have been shown to evolve rapidly due to selective pressures from the host immune system [29]. As expected from observations of multicellular organisms [4], genes commonly expressed (e.g. housekeeping proteins) in All Stages show significantly lower *d*_N_*/d*_S_ values than stage-specific genes, for all comparisons (all *P* < 0.03).

**Figure 2.**
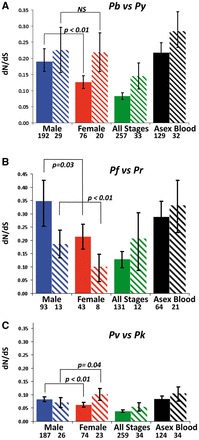
Stage- and location-specific rates of evolution. Estimated as *d*_N_/*d*_S_, for predicted non-membrane (solid bars) and membrane (shaded bars) proteins expressed in sexual (male, female) and asexual blood stage malaria parasites. Genes were classified according to their exclusive detection in *P. berghei* proteomes of Males, Females, Asexual Stages or All Stages (see Fig. 1), The rates of evolution were determined by comparison of genes of the following pairs of *Plasmodium* species: *P. berghei* and *P. yoelii* (**A**); *P. falciparum* and *P. reichenowi* (**B**); *P. vivax* and *P. knowlesi* (**C**).

Genes encoding proteins with a membrane location in Asexual Blood stages have been shown to accumulate mutations faster than non-membrane proteins, which is thought to be due to selective pressures resulting from host immunity [29]. We tested whether the accumulation of mutations was also greater for genes encoding membrane proteins and particularly in genes encoding Male proteins (Fig. S2A–C; Table S2, PB, PF and PV). We find the following patterns (Fig. 2). First, as expected, membrane proteins of Asexual Blood and All Stages evolve faster than their non-membrane proteins (*P* = 0.03 and *P* = 0.01, respectively, for *P. berghei* versus *P. yoelii,* and the same but non-significant trend for the other species comparisons). Second, for Females, genes encoding membrane proteins evolve faster (*P* < 0.01) than genes encoding non-membrane proteins, except in the *P. falciparum* versus *P. reichenowi* comparison where this trend is reversed (*P = *0.01). However, only eight genes are included in the Female membrane subset for *P. falciparum* versus *P. reichenowi*, giving this analysis the least power (>20 genes are compared for the other species pairs). Third, Male genes encoding non-membrane genes are exceptional because there is no significant difference in rates of evolution between non-membrane and membrane proteins (all comparisons, *P* > 0.22). Furthermore, genes encoding Male non-membrane proteins are evolving faster than genes encoding Female non-membrane proteins (*P* < 0.01 for both *P. berghei* versus *P. yoelii* and *P. knowlesi* versus *P. vivax*; *P* = 0.01 for *P. falciparum* versus *P. reichenowi*). In contrast, when comparing only genes encoding membrane proteins, Males are not evolving significantly faster than Females for *P. berghei* versus *P. yoelii* (*P* = 0.46), and Females are evolving faster than Males for the *P. falciparum* versus *P. reichenowi* (P = 0.04) and *P. knowlesi* versus *P. vivax* comparisons (*P* = 0.03).

### Relaxation of constraints

Next, we investigated whether a relaxation of constraints resulting in genetic drift or the fixation of deleterious mutations could explain the accelerated evolution of genes coding for Male non-membrane proteins. First, we compared the rate of synonymous mutations (*d*_S_) according to whether gene expression is Male- or Female-biased and encodes membrane or non-membrane proteins (Table S2, PB, PK and PV). There are no significant differences in *d*_S_ when comparing genes coding for non-membrane proteins of Females with both non-membrane and membrane proteins of Males (*P* > 0.09). This is also the case for genes coding membrane proteins of Females when compared with non-membrane and membrane proteins of Males in the *P. berghei* versus *P. yoelii* comparison (*P* > 0.1). Second, we tested whether the frequency of nonsense mutations (i.e. premature termination and stop codons) differs according to the four different expression categories: Male, Female, All Stages and Asexual Blood. We also tested 789 genes of *P. berghei* and *P. yoelii* for which >20 codons were available (Table S3). A total of 10 (1.3%) contained nonsense mutations. The number of nonsense mutations in Male and Female genes do not significantly differ (*P* > 0.05) from that expected by chance (1.3%), which is consistent with random occurrence of nonsense mutations. The lack of variation in *d*_S_ and the random occurrence of nonsense mutations suggest that the rapid evolution of Male genes is not due to a relaxation of constraints and thus that selection may play a role.

### Host–parasite interactions

We next examined what aspects of parasite ecology could explain the accelerated evolution of Male compared with Female genes, and particularly the exceptional rates of evolution in Male non-membrane genes. When taken up in a blood meal, male and female gametocytes must rapidly differentiate into gametes and mate. Host immune factors (resulting from exposure to circulating gametocytes) are also taken up in the blood meal and can reduce fertility, especially of male gametes, and block transmission [30–32]. In the host, male and female gametocytes are protected inside red blood cells, but in the blood meal, gametocytes become more vulnerable to immune factors because they must exit red blood cells during gametogenesis. In contrast to females, male gametogenesis is complex and involves rapid DNA replication, flagella and gamete construction, extrusion and detachment of gametes [33–35]. Male gametes must then travel through the blood meal to locate and fertilize females.

The more complex activities of males are predicted to make them more vulnerable to host immune factors taken up in the blood meal [36–39]. This could occur because immune factors interact with male proteins with both membrane and non-membrane locations (non-membrane proteins are not exposed in females) and/or immune attack may occur for both sexes, but male proteins may be more easily damaged and the consequences for fertility may be more severe. For example, male fertility is reduced by exposure to transmission-blocking immune factors, such as reactive oxygen species, that affect intracellular processes [40]. Interactions between male proteins and immune factors may also be indirect; for example, antibodies that agglutinate the surface proteins of male gametes may select for faster performance of intracellular proteins involved in flagella formation. Furthermore, senescence or damage may result in male gametocytes inappropriately being activated in the host circulation, which will present both their intracellular and membrane proteins to the host immune system (activated females may only present membrane proteins).

**Figure 3.**
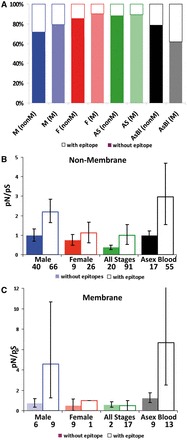
The strength of diversifying selection. Estimated as *p*_N_/*p*_S_, for *P. falciparum* proteins with (solid bars) or without predicted immune epitopes (shaded bars) expressed in sexual (male, female) and asexual blood stage malaria parasites. (**A**) Percentage of proteins containing immune epitopes identified from the Immune Epitope Database and Analysis Resource. Genes were classified according to their exclusive detection in *P. berghei* proteomes of Males, Females, Asexual Stages or All Stages (see Fig. 1). The strength of diversifying selection on *P. falciparum* predicted non-membrane (**B**) and membrane (**C**) proteins.

To investigate whether interactions between parasites and host immune responses could drive the accelerated evolution of genes encoding Male proteins, we first tested whether Male proteins are more immunogenic than those of Females. Because the membrane or non-membrane location of proteins is not a definitive predictor of exposure to host immunity, we classified proteins based on whether they are known immune epitopes. We focused on *P. falciparum* because epitopes for this species have been characterized and experimentally confirmed. These data are available from the Immune Epitope Database and Analysis Resource (http://www.immuneepitope.org/; Table S4). Specifically, we tested whether the proportion of ‘immune epitopes’ of *P. falciparum* proteins differed between Males and Females and their predicted membrane or non-membrane location. This analysis reveals that Male non-membrane proteins have a significantly higher percentage of epitopes compared with those of Females and All Stages (Fig. 3A; Fisher exact test *P* = 0.045 and *P* < 0.0001). Furthermore, the percentage of Male non-membrane proteins with epitopes is comparable to that of Asexual Blood’ stages (Fisher exact test *P* = 0.098). Similar, but non-significant trends are present in the percent of Male membrane proteins with epitopes. This suggests that Male proteins experience greater levels of immune recognition than Female proteins and that the immunogenicity of Males is equivalent to Asexual Blood stages.

We then tested whether the rate of non-synonymous versus synonymous substitutions is higher in genes encoding Male proteins containing epitopes compared with those without epitopes. We analysed the polymorphisms (*p*_N_*/p*_S_*)* in gene sequences for Male membrane and non-membrane proteins, with and without epitopes, from the two most comprehensively sequenced *P. falciparum* strains/isolates (3D7 and Ghana; Table S4). Consistent with other studies [34], we find that *p*_N_*/p*_S_ is high in *Plasmodium* and that genes encoding Male proteins containing epitopes do have a significantly higher *p*_N_*/p*_S_ than Females for both non-membrane (Fig. 3B; *P* = 0.01) and membrane encoding genes (Fig. 3C; *P* < 0.01). Furthermore, the proportion of non-synonymous versus synonymous substitutions is particularly high for Male non-membrane proteins containing epitopes involved in cellular motor machinery such as kinesins or dyneins (Table S4). This suggests that interactions with the host immune system shape genes encoding Male non-membrane proteins.

### Adaptive ‘fast-male’ evolution

Our analyses suggest that accelerated evolution of Male-biased genes encoding both non-membrane and membrane proteins is better explained by selection pressures resulting from interactions with host immunity than a relaxation of constraints. To test if Male-biased genes are evolving under positive selection, we used a modified version of the McDonald–Kreitman test (the ‘DoFE-all method’ [27, 28]) and data from natural isolates of *P. falciparum* (3D7, Dd2, HB3, 7G8, D10, D6, Santa Lucia, K1, RO-33, IT, FCC-2/Hainan, Senegal, IGH-CR14 [41–43]) compared with *P. reichenowi*, and we assume that mutations manifesting as polymorphism occur in more than four isolates. We calculated the fraction of residues fixed by positive selection (*α*) for all genes expressed in Male, Female, All Stages and Asexual Blood classes (Table 1), assuming that all non-synonymous polymorphisms are neutral. When non-synonymous polymorphisms are deleterious and removed from the population, *α* takes a negative value that is not expressed as a proportion (i.e. when *α* is positive, it is expressed as a proportion, but when *α* is negative, it can vary between 0 and infinity). For all expression categories—except for Male genes—*α* is negative (Table 1; Table S5), suggesting that the accelerated evolution of Male genes is driven by positive selection. This is the pattern observed in multicellular taxa: male-biased mutations manifesting as polymorphisms are often subject to positive selection [2–5].

**Table 1.** Adaptive Male evolution

	Number of genes	Genes with frequent polymorphisms	*α*; for frequent polymorphisms
Male			
All	163	21	0.07
Membrane excluded	139	17	0.26
Female			
All	74	8	−0.94
Membrane excluded	57	5	−0.41
Asexual blood			
All	137	25	−0.52
Membrane excluded	109	19	−0.79
All stages			
All	237	18	−5.35
Membrane excluded	211	15	−7.07

Estimates of the fraction (*α*) of fixed non-synonymous polymorphism due to positive selection, with or without restriction to frequent polymorphisms. Natural isolates of *P. falciparum* (Dd2, HB3, 7G8, D10, D6, Santa Lucia, K1, RO-33, IT, FCC-2/Hainan, 3D7, Senegal, IGH-CR14) and *P. reichenowi* are compared. We classify frequent polymorphisms as those observed in four or more isolates.

## DISCUSSION

Our analyses reveal that the accelerated evolution of male-biased genes is not exclusive to multicellular taxa and that sex-specific selection occurs in dioecious single-celled organisms without sex chromosomes. The biology of *Plasmodium* also sheds light on how the rapid and adaptive evolution of male-biased genes occurs. We can rule out a role of sex chromosomes and mutational bias because male gametogenesis only involves three mitoses. However, the ratio of male to female gametes will often be slightly male biased, depending on the multiplicity of infections and presence of transmission-blocking factors [37, 39]. This could result in male gametes behaving as large population (compared with females), resulting in faster removal of deleterious mutations and rapid fixation of beneficial mutations associated with male performance. Multicellular taxa have traditionally been the focus for investigating sex-dependent rates of evolution and their underlying causes, but we demonstrate that *Plasmodium* parasites are also a useful model for research in this area.

Rapid evolution of male traits is often expected to be a response to male–male competition or adaptations to attract females [6, 44], but we show that natural selection driven by host–parasite interactions can shape the evolutionary trajectories of the sexes. How natural and sexual selection interact to affect fitness in a changing environment is a fundamental question in evolutionary biology and has important implications for adaptation and speciation [45]. Studies revealing fast-male evolution in multicellular taxa have implicated sexual selection as a driver [1], but processes such as intra-sex competition, sexual antagonism (conflict) and natural selection can all contribute to driving sex-dependent selection [46]. While our results show that natural selection pressures, in the form of host immune factors, shape sex-dependent selection in *Plasmodium,* sexual selection could also make a contribution. *Plasmodium* populations span from clonal to genetically diverse [47] and infections containing multiple con-specific genotypes provide the opportunity for ‘sperm’ competition between unrelated males. This could be evaluated by comparing male-biased rates of evolution across populations with different inbreeding rates. Another approach lies in the potential offered by unicellular organisms to genetically manipulate fast evolving genes to test whether their functions are adaptations for immune evasion or sperm competition.

How the interplay between phenotypic plasticity and microevolution shapes phenotypes is a key question in evolutionary biology [48] and has implications for the development of transmission-blocking interventions [49, 50]. Evolutionary theory predicts that malaria parasites facultatively increase their investment in males during periods in infections when the host produces immune factors that reduce the fertility of males more than females [36, 51]. By revealing that males are more immunogenic than females, we support this theory and show that, in addition to evading host immunity through phenotypic plasticity in sex ratios [52], parasites can also respond with rapid microevolution. Moreover, by adjusting sex ratios to produce more males when conditions for mating are unfavourable [36, 51], parasites may benefit from maximizing both their immediate mating success and the potential for adaptive evolution in response to the conditions they experience. There is a drive to develop transmission-blocking interventions that, when administered to hosts, kill gametocytes (e.g. gametocidal drugs) or produce immune responses that are taken up in the blood meals of vectors and prevent parasites from mating (e.g. by vaccinating against the antigens of one or both sexes). Our results suggest that a vaccine harnessing host immunity to target males is unlikely to be a novel selection pressure for malaria parasites and that phenotypic plasticity and rapid microevolution could quickly undermine such an intervention.

### Conclusions and implications

Because male-biased genes evolve at an accelerated rate, our results predict that interventions specifically targeting males are more vulnerable to parasite counter-evolution than interventions targeting female or universally expressed genes. Furthermore, our approach, coupled with phenotypic analyses, provides a powerful way to assess the evolutionary potential of candidate antigens and identify slower evolving targets.

## SUPPLEMENTARY DATA

Supplementary data are available at *EMPH* online.

## FUNDING

S.E.R. is supported by a Wellcome Trust Fellowship (WT082234MA); S.K. by a Columb fellowship from the Foundation for Polish Science and supporting grant (1/722/N-COST/2010/0) COST Action of the Polish Ministry of Science); C.J.J. by the EU Seventh Framework Program (FP7/2007-2013; 242095); S.E.R., S.K. and S.M.K. are also supported by the COST Action (BM0802), and S.M.K., A.P.W. and C.J.J. received support from the European Commission (FP7, EVIMalaR Network of Excellence). Funding to pay the Open Access publication charges for this article was provided by xxxxx.

**Conflict of interest**: none declared.

## Supplementary Material

Supplementary Data
