# Supplementary material for: Why are male malaria parasites in such a rush? Sex-specific evolution and host–parasite interactions
Source: Evol Med Public Health. 2012 Nov 26;2013(1):3–13. doi: 10.1093/emph/eos003 (PMC4183958; doi:10.1093/emph/eos003)
Supplement: Supplementary Data [file supp_2013_1_3__index.html]

Supplementary Data 

# Why are male malaria parasites in such a rush?

## Supplementary Data

files

**Files in this Data Supplement:**

- Supplementary Data - zip file
