# Supplementary material for: Why are male malaria parasites in such a rush? Sex-specific evolution and host–parasite interactions
Source: Evol Med Public Health. 2012 Nov 26;2013(1):3–13. doi: 10.1093/emph/eos003 (PMC4183958; doi:10.1093/emph/eos003)
Supplement: Supplementary Data [file supp_eos003_suppl_data.zip › REECE_Table_S4.pdf]

Table A: Pf proteins containing epitopes

Gene	Annotation
	**Orthologs of male non membrane proteins**
PFA0535c	kinesin, putative
PFB0405w	transmission-blocking target antigen s230
PFC0135c	exportin 1, putative
PFC0260w	P-loop containing nucleoside triphospahte hydrolase, putative
PFD0285c	lysine decarboxylase, putative
PFD0590c	DNA polymerase alpha
PFD0875c	conserved Plasmodium protein, unknown function
PFD0905w	conserved Plasmodium protein, unknown function
PFE0415w	transcription factor IIb, putative
PFE0450w	chromosome condensation protein, putative
PFE0465c	RNA polymerase I
PFE0495w	conserved Plasmodium protein, unknown function
PFE1345c	minichromosome maintenance protein 3, putative
PFF0265c	calcium-binding protein, putative
PFF1185w	Smarca -related protein
PFF1470c	DNA polymerase epsilon, catalytic subunit a, putative
PF07_0014	conserved Plasmodium protein, unknown function
MAL7P1.19	ubiquitin transferase, putative
PF07_0055	conserved Plasmodium protein, unknown function
PF07_0101	conserved Plasmodium protein, unknown function
PF08_0108	plasmepsin X
MAL8P1.65	DNA helicase, putative
MAL8P1.34	conserved Plasmodium protein, unknown function
MAL8P1.31	Anamorsin related protein, putative
PFI0260c	dynein heavy chain, putative
PFI0310w	Maf-like protein, putative
PFI0745w	conserved Plasmodium protein, unknown function
PFI1085w	ubiquitin-like protein, putative
PFI1490c	large cyclophilin-like protein
PF10_0224	dynein heavy chain, putative
PF10_0232	Chromodomain-helicase-DNA-binding protein 1 homolog, putative
PF10_0244	formin 2, putative
PF10_0292	conserved Plasmodium protein, unknown function
PF10_0371	conserved Plasmodium protein, unknown function
PF11_0057	conserved Plasmodium protein, unknown function
PF11_0086	MIF4G domain containing protein
PF11_0089	conserved Plasmodium protein, unknown function
PF11_0240	dynein heavy chain, putative
PF11_0381	subtilisin-like protease 2
PF11_0389	calcium-binding protein, putative
PF11_0433	conserved Plasmodium protein, unknown function
PFL0150w	origin recognition complex 1 protein
PFL0350c	conserved Plasmodium protein, unknown function
PFL1180w	chromatin assembly protein (ASF1), putative
PFL1445w	conserved Plasmodium protein, unknown function
PFL2190c	kinesin, putative
MAL13P1.32	MORN repeat protein, putative
MAL13P1.93	conserved Plasmodium protein, unknown function

PF13_0190	conserved Plasmodium protein, unknown function
MAL13P1.216	DNA helicase, putative
MAL13P1.336	conserved Plasmodium protein, unknown function
PF14_0121	conserved Plasmodium protein, unknown function
PF14_0419	conserved Plasmodium protein, unknown function
PF14_0626	dynein beta chain, putative
PF14_0648	conserved Plasmodium protein, unknown function
PF14_0712	conserved Plasmodium protein, unknown function
**Orthologs of male membrane proteins**	
PFB0460c	conserved Plasmodium protein, unknown function
PF08_0113	vacuolar proton translocating ATPase subunit A, putative
PF11_0079	conserved Plasmodium protein, unknown function
PF11_0092	mechanosensitive ion channel protein
MAL13P1.246	conserved Plasmodium membrane protein, unknown function
PF14_0504	conserved Plasmodium protein, unknown function
**Orthologs of female non-membrane proteins**	
PFD0470c	replication factor a protein, putative
PFF0095c	conserved Plasmodium protein, unknown function
PFF0765c	conserved Plasmodium protein, unknown function
MAL7P1.162	dynein heavy chain, putative
PF08_0132	glutamate dehydrogenase, putative
PF08_0126	DNA repair protein rad54, putative
PFI0365w	translation initiation factor SUI1, putative
MAL13P1.83	exportin 1-like protein, putative
MAL13P1.262	conserved Plasmodium protein, unknown function
PF14_0327	methionine aminopeptidase, type II, putative
PF14_0529	gamma-adaptin, putative
PF14_0538	conserved Plasmodium protein, unknown function
**Orthologs of female membrane proteins**	
PFL0655w	conserved Plasmodium membrane protein, unknown function
PF14_0723	LCCL domain-containing protein CCP1
**Orthologs of membrane proteins in all 3 stages**	
PFC0110w	Cytoadherence linked asexual protein 3.2
PFD0660w	phosphoglycerate mutase, putative
PFI0875w	Heat shock protein 70 (HSP70) homologue
PFL1835w	conserved Plasmodium protein, unknown function
**Orthologs of non-membrane proteins in all 3 stages**	
PFA0335w	Rab5c, GTPase
PFA0520c	chromatin assembly factor 1 protein WD40 domain, putative
PFC0140c	N-ethylmaleimide sensitive fusion protein, putative
PFC0520w	26S proteasome regulatory subunit S14, putative
PFE1370w	hsp70 interacting protein, putative
PFF0325c	conserved Plasmodium protein, unknown function
PFF1345w	transportin
PFF1410c	conserved protein, unknown function
PF08_0115	DNAJ protein, putative
PF08_0096	RNA helicase, putative
PF08_0054	heat shock 70 kDa protein
PFI0490c	ran-binding protein, putative
PFI0755c	6-phosphofructokinase, putative
PFI1310w	NAD synthase, putative
PFI1445w	High molecular weight rhoptry protein-2

PF10_0115	QF122 antigen
PF11_0071	RuvB DNA helicase, putative
PF11_0142	ubiquitin domain containing protein
PF11_0177	deubiquinating/deneddylating enzyme
PF11_0188	heat shock protein 90, putative
PF11_0313	60S ribosomal protein P0
PF11_0374	tudor staphylococcal nuclease
PF11_0461	PfRab6, GTPase
PFL0625c	eukaryotic translation initiation factor 3 subunit 10, putative
PFL0895c	conserved Plasmodium protein, unknown function
PFL0930w	clathrin heavy chain, putative
PFL1070c	endoplasmin homolog precursor, putative
PFL1880w	acyl-CoA synthetase, PfACS11
MAL13P1.63	asparagine-rich protein
PF13_0229	aconitase
PF14_0159	Root hair defective 3 GTP-binding protein (RHD3) homolog, putative
PF14_0196	Tetratricopeptide repeat family protein, putative
PF14_0241	basic transcription factor 3b, putative
PF14_0341	glucose-6-phosphate isomerase
PF14_0425	fructose-bisphosphate aldolase
PF14_0627	40S ribosomal protein S3, putative
**Orthologs of non-membrane proteins in asexual blood stages**	
PFB0640c	sec31p putative
PFD0735c	conserved Plasmodium protein, unknown function
PFF1230c	conserved Plasmodium protein, unknown function
PFF1350c	acetyl-CoA synthetase
MAL7P1.38	regulator of chromosome condensation, putative
MAL7P1.119	conserved Plasmodium protein, unknown function
MAL7P1.126	conserved Plasmodium protein, unknown function
PF08_0063	ClpB protein, putative
PFI1155w	conserved Plasmodium protein, unknown function
PF10_0126	conserved Plasmodium protein, unknown function
PF11_0168	moving junction protein
PF11_0171	conserved Plasmodium protein, unknown function
PFL0335c	eukaryotic translation initiation factor 5, putative
PFL1385c	Merozoite Surface Protein 9, MSP-9
PFL1480w	protein transport protein Sec13, putative
PFL1530w	asparagine-rich protein, putative
PFL1605w	pentatricopeptide repeat protein, putative
PFL2355w	conserved Plasmodium protein, unknown function
MAL13P1.19	peptidase, putative
PF13_0137	conserved Plasmodium protein, unknown function
MAL13P1.146	AMP deaminase, putative
PF13_0198	reticulocyte binding protein 2 homolog A
PF13_0219	conserved Plasmodium protein, unknown function
PF13_0233	myosin A
PF13_0350	signal recognition particle receptor alpha subunit, putative
PF14_0102	rhoptry-associated protein 1, RAP1
PF14_0277	coatamer protein, beta subunit, putative
**Orthologs of membrane proteins in asexual blood stages**	
PFD0295c	apical sushi protein, ASP
PFI1475w	merozoite surface protein 1 precursor
PF11_0107	conserved Plasmodium protein, unknown function

PF11_0112	vacuolar sorting protein 35, putative
PF11_0246	conserved Plasmodium protein, unknown function
PF11_0344	apical membrane antigen 1, AMA1
PFL2505c	rhoptry neck protein 3, putative
MAL13P1.60	erythrocyte binding antigen-140
MAL13P1.39	conserved Plasmodium protein, unknown function
PF13_0116	conserved Plasmodium protein, unknown function
PF13_0133	plasmepsin V
PF14_0186	conserved Plasmodium protein, unknown function
PF14_0495	rhoptry neck protein 2

Table B: Fraction of proteins containing epitopes expressed during different stages

Stage	%of proteins containing epitopes recognised by human immune system
Male non membrane	29.3
Male membrane	21.4
Female non membrane	16.8
Female membrane	9.5
Asexual blood stages non membrane	21.1
Asexual blood stages memebrane	38.2
Expressed in 3 stages not membrane	13.6
Expressed in 3 stages membrane	11.8

Table C: Comparison of pn/ps (3d7 versus Ghana isolate) of different proteins of P.falciparum

Gene	product decription	pn/ps	ps	pn
	**Non membrane male genes with epitopes**			
PF14_0712	conserved Plasmodium protein, unknown function	1.5	4	6
PF11_0086	MIF4G domain containing protein	1.18	11	13
PF07_0014	conserved Plasmodium protein, unknown function	4.5	4	18
PF07_0101	conserved Plasmodium protein, unknown function	1	10	10
PFB0405w	transmission-blocking target antigen s230	5.67	3	17
PF14_0419	conserved Plasmodium protein, unknown function	4	3	12
PFL2190c	kinesin, putative	4.67	3	14
MAL7P1,19	ubiquitin transferase, putative	1	5	5
PF11_0240	dynein heavy chain, putative	2.75	4	11
PFD0905w	conserved Plasmodium protein, unknown function	12	0	12
PF14_0626	dynein beta chain, putative	4.5	2	9
PFL1445w	conserved Plasmodium protein, unknown function	2.67	3	8
PF10_0244	formin 2, putative	0	1	0
PFE0495w	conserved Plasmodium protein, unknown function	1	3	3
PFD0875c	conserved Plasmodium protein, unknown function	7	0	7
PF10_0232	Chromodomain-helicase-DNA-binding protein 1 homolog, putative	2.5	2	5
PFE0415w	transcription factor IIb, putative	0	1	0
PFD0590c	DNA polymerase alpha	1.5	2	3
PFA0535c	kinesin, putative	1	2	2
PFE0465c	RNA polymerase I	1	2	2
PFI1085w	ubiquitin-like protein, putative	2	0	2
PFL0350c	conserved Plasmodium protein, unknown function	1.5	2	3
MAL13P1,32	MORN repeat protein, putative	4	1	4
PFI0260c	dynein heavy chain, putative	4	0	4
PFE1345c	minichromosome maintenance protein 3, putative	3	0	3
MAL8P1,34	conserved Plasmodium protein, unknown function	0.5	2	1

PF10_0224	dynein heavy chain, putative	0.5	2	1
PF14_0648	conserved Plasmodium protein, unknown function	1	0	1
PFC0260w	P-loop containing nucleoside triphospahte hydrolase, putative	2	0	2
PF10_0292	conserved Plasmodium protein, unknown function	2	0	2
PF10_0371	conserved Plasmodium protein, unknown function	0	0	0
PFF0265c	calcium-binding protein, putative	1	0	1
PFI0310w	Maf-like protein, putative	1	0	1
PF13_0190	conserved Plasmodium protein, unknown function	0	1	0
PFI1490c	large cyclophilin-like protein	1	0	1
MAL13P1,93	conserved Plasmodium protein, unknown function	1	0	1
MAL8P1,65	DNA helicase, putative	0	1	0
PF11_0381	subtilisin-like protease 2	1	0	1
PFE0450w	chromosome condensation protein, putative	0	1	0
PFF1185w	Smarca -related protein	0	1	0
**Non membrane male genes without epitopes**				
PFB0095c	erythrocyte membrane protein 3	1.38	26	36
PFB0615c	conserved Plasmodium protein, unknown function	2	5	10
PFI0275w	conserved Plasmodium protein, unknown function	7	0	7
PF14_0030	conserved Plasmodium protein, unknown function	0	0	0
PF11_0279	conserved Plasmodium protein, unknown function	7	1	7
PFE0090w	chromosome assembly factor 1, CAF-1	7	1	7
PF11_0243	leucine-rich repeat protein 11, LRR11	1	1	1
PF11_0287	conserved protein, unknown function	0	0	0
PF14_0366	small subunit DNA primase	0	0	0
PF13_0126	translation initiation factor EIF-2B subunit related	2	1	2
MAL13P1,96	chromosome segregation protein, putative	1	0	1
PFI1450c	conserved Plasmodium protein, unknown function	0	0	0
PF08_0100	ruvB-like DNA helicase, putative	0	2	0
PFL0805w	MAC/Perforin, putative	4	0	4
PFF1095w	leucyl tRNA synthase	1	2	2
PFD0250c	Sec24-like protein, putative	2	1	2
PFL1730c	conserved Plasmodium protein, unknown function	0	2	0
MAL13P1,20	conserved Plasmodium protein, unknown function	0	0	0
PF14_0458	conserved Plasmodium protein, unknown function	0	0	0

PFE0595w	prefoldin subunit, putative	0	0	0
PFF1495w	conserved Plasmodium protein, unknown function	0	0	0
PFD0855c	conserved Plasmodium protein, unknown function	2	1	2
MAL8P1,47	conserved Plasmodium protein, unknown function	1	0	1
PF11_0390	conserved Plasmodium protein, unknown function	1	0	1
PF10_0128	WD-repeat protein, putative	1	0	1
PF11_0292	conserved Plasmodium protein, unknown function	0	0	0
PF14_0492	protein phosphatase 2b regulatory subunit, putative	0	0	0
PF11_0524	lsm4 homologue, putative	2	0	2
MAL13P1,57	conserved Plasmodium protein, unknown function	1	1	1
PFE1210c	conserved Plasmodium protein, unknown function	2	0	2
PF13_0060	conserved Plasmodium protein, unknown function	2	0	2
PF07_0068	cysteine desulfurase, putative	2	0	2
PF14_0115	conserved Plasmodium protein, unknown function	2	0	2
MAL7P1,25	cytoskeleton associated protein, putative	0	2	0
PF14_0120	conserved Plasmodium protein, unknown function	1	1	1
PFE0155w	conserved Plasmodium protein, unknown function	0	2	0
MAL8P1,12	conserved Plasmodium protein, unknown function	2	0	2
PFL0445w	conserved Plasmodium protein, unknown function	0	2	0
PFB0400w	transmission-blocking target antigen s230 paralog	1	1	1
MAL13P1,328	DNA topoisomerase VI, B subunit, putative	0	0	0
PF10_0229	conserved Plasmodium protein, unknown function	0	0	0
PF14_0202	dynein-associated protein, putative	0	0	0
PF14_0235	conserved Plasmodium protein, unknown function	0	0	0
PF14_0352	ribonucleoside-diphosphate reductase, large subunit	0	0	0
PFL0190w	ubiquitin conjugating enzyme E2, putative	0	1	0
PFA0345w	centrin-1	0	1	0
MAL13P1,69	septum formation protein MAF homologue, putative	1	0	1
PF11_0440	conserved Plasmodium protein, unknown function	0	1	0
PF11_0147	mitogen-activated protein kinase 2	0	1	0
PF10_0299	glycoprotease, putative	0	1	0
PF08_0129	serine/threonine protein phosphatase, putative	1	0	1
PFI0465c	conserved Plasmodium protein, unknown function	1	0	1
PF11_0056	conserved Plasmodium protein, unknown function	0	1	0
MAL13P1,245	conserved Plasmodium protein, unknown function	1	0	1
MAL7P1,21	origin recognition complex subunit 2, putative	1	0	1

PFI1345c	conserved Plasmodium protein, unknown function	1	0	1
PFB0720c	origin recognition complex subunit 5	0	1	0
PF10_0327	Myb2 protein	1	0	1
PFF0115c	elongation factor G, putative	0	1	0
PF13_0189	conserved Plasmodium protein, unknown function	1	0	1
PF07_0047	cell division cycle ATPase, putative	0	1	0
MAL7P1,131	conserved Plasmodium protein, unknown function	0	0	0
MAL8P1,109	protein phosphatase, putative	0	0	0
PF08_0094	cullin-like protein, putative	0	0	0
PF14_0420	calmodulin-like protein	0	0	0
PFE0455w	phosphatase 1 regulatory subunit, putative	0	0	0
**Membrane male genes with epitopes**				
PFB0460c	conserved Plasmodium protein, unknown function	20	0	20
MAL13P1,246	conserved Plasmodium membrane protein, unknown function	2.5	2	5
PF11_0079	conserved Plasmodium protein, unknown function	2	1	2
PF11_0092	mechanosensitive ion channel protein	1	1	1
PF14_0504	conserved Plasmodium protein, unknown function	1	0	1
PF08_0113	vacuolar proton translocating ATPase subunit A, putative	1	0	1
**Membrane male genes without epitopes**				
PFL1370w	NIMA-related protein kinase, Pfnek-1	0.63	8	5
PFE0340c	rhomboid protease ROM4	2	4	8
MAL8P1,60	conserved Plasmodium protein, unknown function	2	1	2
PF10_0067	conserved Plasmodium protein, unknown function	0	5	0
PF11_0149	rhomboid protease ROM1, putative	0	0	0
PFC0725c	formate-nitrate transporter, putative	1	1	1
PF11_0310	transporter, putative	0	1	0
MAL13P1,22	DNA ligase I	1	0	1
MAL13P1,299	conserved protein, unknown function	0	0	0
MAL8P1,130	conserved Plasmodium membrane protein, unknown function	0	0	0
**Non membrane female genes with epitopes**				

MAL13P1,262	conserved Plasmodium protein, unknown function	2	2	4
MAL7P1,162	dynein heavy chain, putative	0.5	4	2
PF14_0529	gamma-adaptin, putative	0.5	2	1
PFF0765c	conserved Plasmodium protein, unknown function	3	0	3
PF14_0327	methionine aminopeptidase, type II, putative	0	1	0
PFI0365w	translation initiation factor SUI1, putative	1	0	1
PF08_0126	DNA repair protein rad54, putative	1	0	1
MAL13P1,83	exportin 1-like protein, putative	0	0	0
**Non membrane female genes without epitopes**				
PF14_0338	conserved Plasmodium protein, unknown function	0	0	0
MAL7P1,88	thioredoxin-like protein	0	0	0
PF11_0317	structural maintenance of chromosome protein, putative	3	2	6
PFI0315c	dynein light intermediate chain 2, cytosolic	2	2	4
PFI0450c	apoptosis-related protein, putative	0	0	0
PF11_0347	conserved Plasmodium protein, unknown function	1	2	2
PFC0500w	conserved protein, unknown function	0	0	0
PF14_0282	conserved protein, unknown function	3	1	3
PF11_0043	60S ribosomal protein P1, putative	0	1	0
PF14_0586	conserved Plasmodium protein, unknown function	1	1	1
MAL8P1,83	eukaryotictranslation initiation factor, putative	1	0	1
PF10_0040	conserved Plasmodium protein, unknown function	0.5	2	1
PFE1400c	beta adaptin protein, putative	1	1	1
PFA0190c	actin-related protein, ARP1	0	1	0
PFL1685w	conserved Plasmodium protein, unknown function	0	0	0
PFL1630c	conserved Plasmodium protein, unknown function	1	0	1
PFI0245c	conserved Plasmodium protein, unknown function	0	1	0
PF11_0258	co-chaperone GrpE, putative	1	0	1
PF10_0144	prohibitin, putative	0	1	0
PF13_0250	G-beta repeat protein, putative	0	1	0
MAL13P1,164	elongation factor Tu, putative	1	0	1
PFC1065w	conserved Plasmodium protein, unknown function	1	0	1
PF14_0334	NAD(P)H-dependent glutamate synthase, putative	1	0	1
MAL7P1,100	protein kinase	0	0	0
MAL8P1,96	CS domain protein, putative	0	0	0
PFD0311w	cytosolic glyoxalase II	0	0	0

PFI1435w	RNA binding protein, putative	0	0	0
**Membrane female genes with epitopes**				
PFL0655w	conserved Plasmodium membrane protein, unknown function	0	0	0
PF14_0723	LCCL domain-containing protein CCP1	1	1	1
**Membrane female genes without epitopes**				
PFL2405c	PFG377 protein	1.4	5	7
PFE1340w	conserved Plasmodium protein, unknown function	0	0	0
PF13_0248	Pfs47	3	0	3
PFD1035w	steroid dehydrogenase, putative	0	1	0
PFC0381c	conserved Plasmodium protein, unknown function	0	0	0
PFI0935w	DNAJ-like molecular chaperone protein, putative	0	1	0
PFF1265w	oxidoreductase, short-chain dehydrogenase family, putative	0	1	0
MAL7P1,67	conserved Plasmodium protein, unknown function	0	0	0
PF14_0067	LCCL domain-containing protein CCP3	0	1	0
**Non membrane genes with epitopes expressed in all three stages**				
PFE1370w	hsp70 interacting protein, putative	0.5	8	4
PFI1445w	High molecular weight rhoptry protein-2	1.5	2	3
PF11_0188	heat shock protein 90, putative	4	1	4
PF10_0115	QF122 antigen	5	0	5
PFI0490c	ran-binding protein, putative	0	0	0
PFF1345w	transportin	3	1	3
PFL0930w	clathrin heavy chain, putative	0	4	0
PFL0895c	conserved Plasmodium protein, unknown function	0	1	0
PFI0755c	6-phosphofructokinase, putative	0	3	0
PFA0335w	Rab5c, GTPase	1	0	1
PFI1310w	NAD synthase, putative	2	0	2
MAL13P1,63	asparagine-rich protein	0	1	0
PF11_0142	ubiquitin domain containing protein	0	0	0
PF14_0627	40S ribosomal protein S3, putative	0	0	0
PF11_0071	RuvB DNA helicase, putative	0	1	0

PFF1410c	conserved protein, unknown function	0	1	0
PFC0140c	N-ethylmaleimide sensitive fusion protein, putative	1	0	1
PF14_0159	Root hair defective 3 GTP-binding protein (RHD3) homolog, putative	1	0	1
PF11_0374	tudor staphylococcal nuclease	1	0	1
PFL0625c	eukaryotic translation initiation factor 3 subunit 10, putative	0	1	0
**Non membrane genes without epitopes expressed in all three stages**				
PF07_0029	heat shock protein 86	0	0	0
MAL8P1,69	14-3-3 protein, putative	0	0	0
PFB0445c	DEAD box helicase, UAP56	0	0	0
PFI0165c	DEAD/DEAH box helicase, putative	0.2	5	1
PF14_0448	40S ribosomal protein S2, putative	1	0	1
PFL1465c	Heat shock protein hslv	0	0	0
PF14_0321	ABC transporter, putative	1	0	1
PF14_0301	conserved protein, unknown function	1	0	1
PFC0271c	glutaredoxin 1	0	0	0
PFE0915c	proteasome subunit beta type 1, putative	0	0	0
PF13_0268	60S ribosomal protein L17, putative	1	0	1
PFL1110c	CAMP-dependent protein kinase regulatory subunit, putative	1	0	1
MAL8P1,125	tyrosyl-tRNA synthetase, putative	0	0	0
PF08_0081	conserved Plasmodium protein, unknown function	0	0	0
PF10_0063	DNA/RNA-binding protein, putative	0	0	0
PFL1550w	lipoamide dehydrogenase	0	0	0
PFD0830w	bifunctional dihydrofolate reductase-thymidylate synthase	3	0	3
PF07_0112	proteasome subunit alpha type 5, putative	0	0	0
PF11_0208	phosphoglycerate mutase, putative	0	0	0
PF11_0272	40S ribosomal protein S18, putative	0	0	0
PFA0400c	beta3 proteasome subunit, putative	0	0	0
PFC1020c	40S ribosomal protein S3A, putative	2	0	2
PFC0870w	elongation factor 1 (EF-1), putative	1	0	1
PFF0420c	proteasome subunit alpha type 2, putative	0	1	0
PF08_0109	proteasome subunit alpha type 5, putative	2	0	2
PFI1170c	thioredoxin reductase	1	1	1
PFE1250w	acyl-CoA synthetase, PfACS10	2	0	2
PF10_0340	methionine-tRNA ligase, putative	1	1	1
PFC0350c	TCP-1/cpn60 chaperonin family	0	1	0

PF14_0632	26S proteasome subunit, putative	2	0	2
PF11_0465	dynamin-like protein	0	1	0
PF13_0044	carbamoyl phosphate synthetase	1	1	1
MAL13P1,283	TCP-1/cpn60 chaperonin family, putative	0	0	0
PF13_0262	lysine - tRNA ligase	0	0	0
PF14_0296	60S ribosomal protein L14, putative	0	0	0
PF14_0401	tRNA binding protein, putative	0	0	0
PFD0950w	ran binding protein 1, putative	0	0	0
PFL1245w	ubiquitin-activating enzyme E1, putative	0	0	0
PFL2005w	replication factor C subunit 4	0	0	0
PFC0975c	peptidyl-prolyl cis-trans isomerase	1	0	1
PF07_0079	60S ribosomal protein L11a, putative	0	1	0
PFF1335c	4-methyl-5(B-hydroxyethyl)-thiazol monophosphate biosynthesis enzyme	1	0	1
PF10_0325	haloacid dehalogenase-like hydrolase, putative	1	0	1
PFL0110c	mitochondrial phosphate carrier protein	0	1	0
MAL7P1,81	eukaryotic translation initiation factor 3 37,28 kDa subunit, putative	0	1	0
PF14_0615	ATP synthase (C/AC39) subunit, putative	1	0	1
PFE0350c	60S ribosomal protein L4, putative	1	0	1
PF13_0143	phosphoribosylpyrophosphate synthetase	1	0	1
PF10_0081	26S proteasome regulatory subunit 4, putative	1	0	1
PF11_0302	conserved Plasmodium protein, unknown function	1	0	1
PF14_0192	glutathione reductase	1	0	1
PFD0305c	vacuolar ATP synthase subunit b	0	1	0
PFF1155w	hexokinase	0	1	0
PFI1020c	inosine-5'-monophosphate dehydrogenase	1	0	1
PFC0190c	EH (Eps15 homology) protein	0	1	0
PF10_0077	eukaryotic translation initiation factor 3 subunit 7, putative	0	1	0
PFL1425w	t-complex protein 1, gamma subunit, putative	0	1	0
PFB0795w	ATP synthase F1, alpha subunit, putative	0	1	0
PFA0145c	aspartyl-tRNA synthetase, putative	1	0	1
PF11_0351	heat shock protein hsp70 homologue	1	0	1
PFF0250w	RNA binding protein, putative	1	0	1
PFF0940c	cell division cycle protein 48 homologue, putative	0	1	0
PF07_0033	Cg4 protein	0	1	0
PF11_0375	conserved Plasmodium protein, unknown function	1	0	1
PF11_0396	Protein phosphatase 2C	0	1	0

PFB0260w	proteasome 26S regulatory subunit, putative	0	1	0
PFE1195w	karyopherin beta	0	1	0
MAL13P1,233	nucleic acid binding protein, putative	0	0	0
MAL13P1,270	proteasome subunit, putative	0	0	0
MAL13P1,92	40S ribosomal protein S15/S19, putative	0	0	0
MAL7P1,122	conserved GTP-binding protein, putative	0	0	0
PF07_0072	calcium-dependent protein kinase 4	0	0	0
PF07_0080	40S ribosomal protein S10, putative	0	0	0
PF08_0110	PfRab18, GTPase	0	0	0
PF10_0043	60S ribosomal protein L13, putative	0	0	0
PF10_0084	tubulin beta chain, putative	0	0	0
PF10_0153	heat shock protein 60	0	0	0
PF11_0065	40S ribosomal protein S4, putative	0	0	0
PF13_0214	elongation factor 1-gamma, putative	0	0	0
PF14_0142	serine/threonine protein phosphatase	0	0	0
PF14_0359	HSP40, subfamily A, putative	0	0	0
PF14_0655	helicase 45	0	0	0
PF14_0716	proteosome subunit alpha type 1, putative	0	0	0
PFC0290w	40S ribosomal protein S23, putative	0	0	0
PFC0300c	60S ribosomal protein L7, putative	0	0	0
PFC0735w	40S ribosomal protein S15A, putative	0	0	0
PFD1055w	40S ribosomal protein S19, putative	0	0	0
PFE1005w	40S ribosomal protein S9, putative	0	0	0
PFF0530w	transketolase	0	0	0
PFI0630w	26S proteasome regulatory subunit, putative	0	0	0
PFI1175c	RNA binding protein, putative	0	0	0
PFL0310c	eukaryotic translation initiation factor 3 subunit 8, putative	0	0	0
PFL0900c	arginyl-tRNA synthetase, putative	0	0	0
PFL1720w	serine hydroxymethyltransferase	0	0	0
	**Membrane genes with epitopes expressed in all three stages**			
PFL1835w	conserved Plasmodium protein, unknown function	1	3	3
PFD0660w	phosphoglycerate mutase, putative	0	5	0
	**Membrane genes without epitopes expressed in all three stages**			

PFL0865w	conserved protein, unknown function	1	3	3
MAL13P1,351	conserved Plasmodium protein, unknown function	2	0	2
PFB0210c	hexose transporter, PfHT1	0	2	0
PFL0590c	non-SERCA-type Ca2+ -transporting P-ATPase	2	0	2
PF11_0055	conserved protein, unknown function	0	0	0
PFE0850c	60S ribosomal protein L12, putative	0	0	0
PF11_0164	peptidyl-prolyl cis-trans isomerase	0	1	0
PFL1845c	calcyclin binding protein, putative	0	1	0
PF10_0366	ADP/ATP transporter on adenylate translocase	1	0	1
PF11_0301	spermidine synthase	0	1	0
PF11_0098	endoplasmic reticulum-resident calcium binding protein	1	0	1
PF14_0075	plasmepsin IV	1	0	1
PFE1405c	eukaryotic translation initiation factor 3, subunit 6, putative	1	0	1
MAL8P1,105	conserved protein, unknown function	1	0	1
PFA0310c	calcium-transporting ATPase, putative	0	1	0
PF13_0119	Rab11a, GTPase	0	0	0
PF14_0230	60S ribosomal protein L5, putative	0	0	0
**Non membrane genes with epitopes expressed in asexual blood stages**				
MAL13P1,19	peptidase, putative	16.5	2	33
PFF1350c	acetyl-CoA synthetase	1.5	6	9
PF13_0137	conserved Plasmodium protein, unknown function	3.33	3	10
PFL1605w	pentatricopeptide repeat protein, putative	8	0	8
PFD0735c	conserved Plasmodium protein, unknown function	5	1	5
PFL1530w	asparagine-rich protein, putative	4	1	4
PF14_0102	rhoptry-associated protein 1, RAP1	4	0	4
MAL7P1,126	conserved Plasmodium protein, unknown function	1	0	1
PF11_0171	conserved Plasmodium protein, unknown function	2	0	2
PF13_0233	myosin A	2	0	2
PF08_0063	ClpB protein, putative	1	1	1
PF11_0168	moving junction protein	0	1	0
PF14_0277	coatamer protein, beta subunit, putative	0	0	0
PFF1230c	conserved Plasmodium protein, unknown function	1	0	1
MAL13P1,146	AMP deaminase, putative	0	1	0
PFB0640c	sec31p putative	0	0	0

Non membrane genes without epitopes expressed in asexual blood stages				
MAL8P1,73	conserved Plasmodium protein, unknown function	1	1	1
PFB0680w	rhoptry neck protein 6	2	0	2
PFF0460w	conserved Plasmodium protein, unknown function	4	1	4
PF13_0315	RNA binding protein, putative	0	0	0
PFF0625w	nucleolar GTP-binding protein 1, putative	0.33	3	1
PFL0035c	acyl-CoA synthetase, PfACS7	4	0	4
PFC0950c	peptidase, putative	3	1	3
PFB0355c	serine repeat antigen 2 (SERA-2)	3	0	3
PF14_0224	serine/threonine protein phosphatase	0	0	0
PF11_0268	kelch motif containing protein, putative	3	0	3
PFF0950w	conserved Plasmodium protein, unknown function	2	1	2
PF13_0345	aminomethyltransferase, mitochondrial precursor	1	0	1
PFF0675c	myosin E	0.5	2	1
PF11_0295	farnesyl pyrophosphate synthase, putative	0	0	0
PFD0725c	arsenical pump-driving ATPase, putative	0	2	0
PFB0200c	aminotransferase, classes I and II, putative	0	2	0
PFL2245w	signal recognition particle, beta subunit, putative	0	1	0
PF14_0527	conserved Plasmodium protein, unknown function	2	0	2
PFL2250c	rac-beta serine/threonine protein kinase, PfPKB	1	1	1
PF11_0212	tRNA nucleotidyltransferase, putative	1	1	1
PF08_0086	RNA binding protein, putative	2	0	2
MAL13P1,121	adenosine-diphosphatase	1	0	1
PF14_0088	aldo/keto reductase, putative	2	0	2
PF11_0189	insulinase, putative	1	1	1
PFL0975w	conserved Plasmodium protein, unknown function	1	1	1
PF08_0091	conserved Plasmodium protein, unknown function	0	0	0
PFL2225w	myosin A tail domain interacting protein	1	0	1
PFE1285w	membrane skeletal protein IMC1-related	1	0	1
PFD0605c	conserved Plasmodium protein, unknown function	0	1	0
PFI1130c	DNA-directed RNA polymerase II, putative	1	0	1
PF10_0306	MORN repeat protein, putative	1	0	1
MAL13P1,221	aspartate carbamoyltransferase	1	0	1
PF10_0272	60S ribosomal protein L3, putative	1	0	1
PFI0820c	RNA binding protein, putative	1	0	1

PFI0195c	GTPase activating protein, GAP	1	0	1
PF11_0047	actin-like protein, putative	0	1	0
PFC0340w	DNA polymerase epsilon subunit B, putative	0	1	0
PF13_0253	ethanolamine-phosphate cytidylyltransferase, putative	0	1	0
PF13_0234	phosphoenolpyruvate carboxykinase	1	0	1
MAL13P1,344	RNAse L inhibitor protein, putative	1	0	1
PFE0605c	glutathione synthetase	1	0	1
PF10_0331	Sec1 family protein, putative	1	0	1
PF14_0616	ATP-dependent protease la, putative	0	1	0
PF13_0323	binding protein, putative	1	0	1
PFF1055c	conserved Plasmodium protein, unknown function	0	1	0
MAL8P1,55	conserved Plasmodium protein, unknown function	1	0	1
PF14_0657	conserved Plasmodium protein, unknown function	1	0	1
PF10_0246	conserved Plasmodium protein, unknown function	1	0	1
PFL1490w	Atypical protein kinase, RIO family, putative	1	0	1
PF14_0344	conserved Plasmodium protein, unknown function	1	0	1
PF14_0428	histidine --tRNA ligase, putative	1	0	1
PF10_0099	conserved Plasmodium protein, unknown function	1	0	1
MAL8P1,62	conserved Plasmodium protein, unknown function	0	0	0
PF10_0268	merozoite capping protein 1	0	0	0
PF11_0114	actin-like protein homolog, ALP1 homolog	0	0	0
**Membrane genes with epitopes expressed in asexual blood stages**				
PFI1475w	merozoite surface protein 1 precursor	10.67	3	32
PF11_0344	apical membrane antigen 1, AMA1	30	1	30
MAL13P1,39	conserved Plasmodium protein, unknown function	8.5	2	17
PF14_0495	rhoptry neck protein 2	2.71	7	19
PF14_0186	conserved Plasmodium protein, unknown function	0	0	0
PFL2505c	rhoptry neck protein 3, putative	2	2	4
PF11_0107	conserved Plasmodium protein, unknown function	3	0	3
PFD0295c	apical sushi protein, ASP	1	0	1
PF13_0133	plasmepsin V	1	1	1
PF11_0112	vacuolar sorting protein 35, putative	1	0	1

Membrane genes without epitopes expressed in asexual blood stages				
PF14_0455	multidrug resistance protein 2 (heavy metal transport family)	3	1	3
PF13_0265	conserved Plasmodium protein, unknown function	4	0	4
PFE1445c	conserved Plasmodium protein, unknown function	0	3	0
PFD0720w	conserved ARM repeats protein, unknown function	0	0	0
PF13_0270	conserved Plasmodium protein, unknown function	1	0	1
PF14_0660	protein phosphatase, putative	1	0	1
PFD0240c	6-cysteine protein, putative	0	1	0
PF13_0032	hydrolase, putative	1	0	1
PF10_0130	conserved Plasmodium protein, unknown function	0	1	0
PF14_0417	HSP90	1	0	1
PF11_0203	peptidase, putative	1	0	1
PFL1700c	V-type K+-independent H+-translocating inorganic pyrophosphatase	1	0	1
PF14_0530	ferlin, putative	1	0	1

Table D: pn/ps for membrane proteins

Set	Number of genes	Mean pn/ps	CI 95%	CI 95%
male proteins with epitopes	6	4.5833	1.2500	10.6667
male proteins without epitopes	9	0.7367	0.3333	1.1811
female proteins with epitopes	1	1.0000	1.0000	1.0000
female proteins without epitopes	9	0.4889	0.0000	1.1333
asexual blood stage proteins with epitopes	9	6.6533	2.5189	12.1345
asexual blood stage proteins without epitopes	13	1.2308	0.7692	1.7692
expressed in all 3 stages proteins with epitopes	2	0.5000	0.0000	1.0000
expressed in all 3 stages proteins without epitopes	17	0.5882	0.3529	0.8824

Table E: pn/ps comparisons for membrane proteins

Set 1	Mean pn/ps	Set 2	Mean pn/ps	Pvalue Set 1 > Set 2
Male proteins without epitopes	0.7367	Asexual proteins without epitopes	1.2308	0.1078
Male proteins without epitopes	0.7367	Asexual proteins with epitopes	6.6533	0.0001
Male proteins without epitopes	0.7367	Male proteins without epitopes	0.7367	0.4888
Male proteins without epitopes	0.7367	Male proteins with epitopes	4.5833	0.0067
Male proteins without epitopes	0.7367	Female proteins without epitopes	0.4889	0.7323
Male proteins without epitopes	0.7367	Female proteins with epitopes	1	0.1426
Male proteins without epitopes	0.7367	Expressed in all 3 stages without epitopes	0.5882	0.6902
Male proteins without epitopes	0.7367	Expressed in all 3 stages proteins with epitopes	0.5	0.6962
Male proteins with epitopes	4.5833	Asexual proteins without epitopes	1.2308	0.917
Male proteins with epitopes	4.5833	Asexual proteins with epitopes	6.6533	0.3002
Male proteins with epitopes	4.5833	Male proteins without epitopes	0.7367	0.9937
Male proteins with epitopes	4.5833	Male proteins with epitopes	4.5833	0.4819
Male proteins with epitopes	4.5833	Female proteins without epitopes	0.4889	0.9958
Male proteins with epitopes	4.5833	Female proteins with epitopes	1	0.9838
Male proteins with epitopes	4.5833	Expressed in all 3 stages without epitopes	0.5882	0.9998
Male proteins with epitopes	4.5833	Expressed in all 3 stages proteins with epitopes	0.5	0.9954

Female proteins without epitopes	0.4889	Asexual proteins without epitopes	1.2308	0.0508
Female proteins without epitopes	0.4889	Asexual proteins with epitopes	6.6533	0.0001
Female proteins without epitopes	0.4889	Male proteins without epitopes	0.7367	0.2667
Female proteins without epitopes	0.4889	Male proteins with epitopes	4.5833	0.003
Female proteins without epitopes	0.4889	Female proteins without epitopes	0.4889	0.4535
Female proteins without epitopes	0.4889	Female proteins with epitopes	1	0.0488
Female proteins without epitopes	0.4889	Expressed in all 3 stages without epitopes	0.5882	0.3622
Female proteins without epitopes	0.4889	Expressed in all 3 stages proteins with epitopes	0.5	0.4205
Female proteins with epitopes	1	Asexual proteins without epitopes	1.2308	0.2013
Female proteins with epitopes	1	Asexual proteins with epitopes	6.6533	0
Female proteins with epitopes	1	Male proteins without epitopes	0.7367	0.8301
Female proteins with epitopes	1	Male proteins with epitopes	4.5833	0
Female proteins with epitopes	1	Female proteins without epitopes	0.4889	0.923
Female proteins with epitopes	1	Female proteins with epitopes	1	0
Female proteins with epitopes	1	Expressed in all 3 stages without epitopes	0.5882	0.985
Female proteins with epitopes	1	Expressed in all 3 stages proteins with epitopes	0.5	0.752
Asexual proteins without epitopes	1.2308	Asexual proteins without epitopes	1.2308	0.4639
Asexual proteins without epitopes	1.2308	Asexual proteins with epitopes	6.6533	0.0022
Asexual proteins without epitopes	1.2308	Male proteins without epitopes	0.7367	0.8823
Asexual proteins without epitopes	1.2308	Male proteins with epitopes	4.5833	0.0865
Asexual proteins without epitopes	1.2308	Female proteins without epitopes	0.4889	0.9464
Asexual proteins without epitopes	1.2308	Female proteins with epitopes	1	0.717
Asexual proteins without epitopes	1.2308	Expressed in all 3 stages without epitopes	0.5882	0.9707
Asexual proteins without epitopes	1.2308	Expressed in all 3 stages proteins with epitopes	0.5	0.9257
Asexual proteins with epitopes	6.6533	Asexual proteins without epitopes	1.2308	0.9983
Asexual proteins with epitopes	6.6533	Asexual proteins with epitopes	6.6533	0.4981
Asexual proteins with epitopes	6.6533	Male proteins without epitopes	0.7367	1
Asexual proteins with epitopes	6.6533	Male proteins with epitopes	4.5833	0.7003
Asexual proteins with epitopes	6.6533	Female proteins without epitopes	0.4889	1
Asexual proteins with epitopes	6.6533	Female proteins with epitopes	1	0.9999
Asexual proteins with epitopes	6.6533	Expressed in all 3 stages without epitopes	0.5882	1
Expressed in all 3 stages without epitopes	0.5882	Asexual proteins without epitopes	1.2308	0.026
Expressed in all 3 stages without epitopes	0.5882	Asexual proteins with epitopes	6.6533	0
Expressed in all 3 stages without epitopes	0.5882	Male proteins without epitopes	0.7367	0.3215
Expressed in all 3 stages without epitopes	0.5882	Male proteins with epitopes	4.5833	0.0001
Expressed in all 3 stages without epitopes	0.5882	Female proteins without epitopes	0.4889	0.6313

Expressed in all 3 stages without epitopes	0.5882	Female proteins with epitopes	1	0.0063
Expressed in all 3 stages without epitopes	0.5882	Expressed in all 3 stages without epitopes	0.5882	0.4574
Expressed in all 3 stages without epitopes	0.5882	Expressed in all 3 stages proteins with epitopes	0.5	0.5966
Expressed in all 3 stages proteins with epitopes	0.5	Asexual proteins without epitopes	1.2308	0.0493
Expressed in all 3 stages proteins with epitopes	0.5	Asexual proteins with epitopes	6.6533	0
Expressed in all 3 stages proteins with epitopes	0.5	Male proteins without epitopes	0.7367	0.294
Expressed in all 3 stages proteins with epitopes	0.5	Male proteins with epitopes	4.5833	0
Expressed in all 3 stages proteins with epitopes	0.5	Female proteins without epitopes	0.4889	0.5448
Expressed in all 3 stages proteins with epitopes	0.5	Female proteins with epitopes	1	0
Expressed in all 3 stages proteins with epitopes	0.5	Expressed in all 3 stages without epitopes	0.5882	0.398
Expressed in all 3 stages proteins with epitopes	0.5	Expressed in all 3 stages proteins with epitopes	0.5	0.3111

Table F: pn/ps for non-membrane proteins

Set	Number of genes	Mean pn/ps	CI 95%	CI 95%
male proteins with epitopes	40	2.20475	1.6442375	2.8420125
male proteins without epitopes	66	0.990606061	0.696969697	1.32030303
female proteins with epitopes	9	1.111111111	0.611111111	1.666666667
female proteins without epitopes	26	0.75	0.461538462	1.057692308
asexual blood stage proteins with epitopes	17	2.960588235	1.567764706	4.735294118
asexual blood stage proteins without epitopes	55	0.996909091	0.787636364	1.221090909
expressed in all 3 stages proteins with epitopes	20	1	0.5	1.55
expressed in all 3 stages proteins without epitopes	91	0.375824176	0.274725275	0.485714286

Table G: pn/ps comparisons for non-membrane proteins

Set 1	Mean pn/ps	Set 2	Mean pn/ps	Pvalue Set 1 > Set 2
Male proteins without epitopes	0.9906	Asexual proteins without epitopes	0.9969	0.4735
Male proteins without epitopes	0.9906	Asexual proteins with epitopes	2.9606	0.0052
Male proteins without epitopes	0.9906	Male proteins without epitopes	0.9906	0.4977
Male proteins without epitopes	0.9906	Male proteins with epitopes	2.2048	0.0008
Male proteins without epitopes	0.9906	Female proteins without epitopes	0.75	0.8232
Male proteins without epitopes	0.9906	Female proteins with epitopes	1.1111	0.3827
Male proteins without epitopes	0.9906	Expressed in all 3 stages without epitopes	0.3758	0.9999
Male proteins without epitopes	0.9906	Expressed in all 3 stages proteins with epitopes	1	0.5013
Male proteins with epitopes	2.2048	Asexual proteins without epitopes	0.9969	0.9998
Male proteins with epitopes	2.2048	Asexual proteins with epitopes	2.9606	0.2394
Male proteins with epitopes	2.2048	Male proteins without epitopes	0.9906	0.9993
Male proteins with epitopes	2.2048	Male proteins with epitopes	2.2048	0.5014
Male proteins with epitopes	2.2048	Female proteins without epitopes	0.75	0.9999
Male proteins with epitopes	2.2048	Female proteins with epitopes	1.1111	0.9902
Male proteins with epitopes	2.2048	Expressed in all 3 stages without epitopes	0.3758	1

Male proteins with epitopes	2.2048	Expressed in all 3 stages proteins with epitopes	1	0.9934
Female proteins without epitopes	0.75	Asexual proteins without epitopes	0.9969	0.1302
Female proteins without epitopes	0.75	Asexual proteins with epitopes	2.9606	0.0006
Female proteins without epitopes	0.75	Male proteins without epitopes	0.9906	0.1758
Female proteins without epitopes	0.75	Male proteins with epitopes	2.2048	0
Female proteins without epitopes	0.75	Female proteins without epitopes	0.75	0.4861
Female proteins without epitopes	0.75	Female proteins with epitopes	1.1111	0.16
Female proteins without epitopes	0.75	Expressed in all 3 stages without epitopes	0.3758	0.981
Female proteins without epitopes	0.75	Expressed in all 3 stages proteins with epitopes	1	0.2505
Female proteins with epitopes	1.1111	Asexual proteins without epitopes	0.9969	0.6183
Female proteins with epitopes	1.1111	Asexual proteins with epitopes	2.9606	0.0156
Female proteins with epitopes	1.1111	Male proteins without epitopes	0.9906	0.6202
Female proteins with epitopes	1.1111	Male proteins with epitopes	2.2048	0.0123
Female proteins with epitopes	1.1111	Female proteins without epitopes	0.75	0.8406
Female proteins with epitopes	1.1111	Female proteins with epitopes	1.1111	0.4883
Female proteins with epitopes	1.1111	Expressed in all 3 stages without epitopes	0.3758	0.9933
Female proteins with epitopes	1.1111	Expressed in all 3 stages proteins with epitopes	1	0.6046
Asexual proteins without epitopes	0.9969	Asexual proteins without epitopes	0.9969	0.4939
Asexual proteins without epitopes	0.9969	Asexual proteins with epitopes	2.9606	0.0042
Asexual proteins without epitopes	0.9969	Male proteins without epitopes	0.9906	0.5236
Asexual proteins without epitopes	0.9969	Male proteins with epitopes	2.2048	0.0002
Asexual proteins without epitopes	0.9969	Female proteins without epitopes	0.75	0.8726
Asexual proteins without epitopes	0.9969	Female proteins with epitopes	1.1111	0.3773
Asexual proteins without epitopes	0.9969	Expressed in all 3 stages without epitopes	0.3758	1
Asexual proteins without epitopes	0.9969	Expressed in all 3 stages proteins with epitopes	1	0.5106
Asexual proteins with epitopes	2.9606	Asexual proteins without epitopes	0.9969	0.9965
Asexual proteins with epitopes	2.9606	Asexual proteins with epitopes	2.9606	0.4929
Asexual proteins with epitopes	2.9606	Male proteins without epitopes	0.9906	0.9928
Asexual proteins with epitopes	2.9606	Male proteins with epitopes	2.2048	0.7562
Asexual proteins with epitopes	2.9606	Female proteins without epitopes	0.75	0.9991
Asexual proteins with epitopes	2.9606	Female proteins with epitopes	1.1111	0.9818
Asexual proteins with epitopes	2.9606	Expressed in all 3 stages without epitopes	0.3758	1
Asexual proteins with epitopes	2.9606	Expressed in all 3 stages proteins with epitopes	1	0.9892
Expressed in all 3 stages without epitopes	0.3758	Asexual proteins without epitopes	0.9969	0
Expressed in all 3 stages without epitopes	0.3758	Asexual proteins with epitopes	2.9606	0
Expressed in all 3 stages without epitopes	0.3758	Male proteins without epitopes	0.9906	0.0001

Expressed in all 3 stages without epitopes	0.3758	Male proteins with epitopes	2.2048	0
Expressed in all 3 stages without epitopes	0.3758	Female proteins without epitopes	0.75	0.0163
Expressed in all 3 stages without epitopes	0.3758	Female proteins with epitopes	1.1111	0.0062
Expressed in all 3 stages without epitopes	0.3758	Expressed in all 3 stages without epitopes	0.3758	0.4947
Expressed in all 3 stages without epitopes	0.3758	Expressed in all 3 stages proteins with epitopes	1	0.0173
Expressed in all 3 stages proteins with epitopes	1	Asexual proteins without epitopes	0.9969	0.4884
Expressed in all 3 stages proteins with epitopes	1	Asexual proteins with epitopes	2.9606	0.0109
Expressed in all 3 stages proteins with epitopes	1	Male proteins without epitopes	0.9906	0.4987
Expressed in all 3 stages proteins with epitopes	1	Male proteins with epitopes	2.2048	0.0051
Expressed in all 3 stages proteins with epitopes	1	Female proteins without epitopes	0.75	0.7453
Expressed in all 3 stages proteins with epitopes	1	Female proteins with epitopes	1.1111	0.3931
Expressed in all 3 stages proteins with epitopes	1	Expressed in all 3 stages without epitopes	0.3758	0.9817
Expressed in all 3 stages proteins with epitopes	1	Expressed in all 3 stages proteins with epitopes	1	0.489

Table H: Male genes sorted by pn/ps (3d7 versus Ghana isolate) for different proteins of F

Gene	product decription	pn/ps	ps	pn	Location	Epitope?
PFB0460c	conserved Plasmodium protein, unknown function	20	0	20	membrane	epitope
PFD0905w	conserved Plasmodium protein, unknown function	12	0	12	non-membrane	epitope
PFI0275w	conserved Plasmodium protein, unknown function	7	0	7	non-membrane	no epitope
PFE0090w	chromosome assembly factor 1, CAF-1	7	1	7	non-membrane	no epitope
PFD0875c	conserved Plasmodium protein, unknown function	7	0	7	non-membrane	epitope
PF11_0279	conserved Plasmodium protein, unknown function	7	1	7	non-membrane	no epitope
PFB0405w	transmission-blocking target antigen s230	5.67	3	17	non-membrane	epitope
PFL2190c	kinesin, putative	4.67	3	14	non-membrane	epitope
PF14_0626	dynein beta chain, putative	4.5	2	9	non-membrane	epitope
PF07_0014	conserved Plasmodium protein, unknown function	4.5	4	18	non-membrane	epitope
PFL0805w	MAC/Perforin, putative	4	0	4	non-membrane	no epitope
PFI0260c	dynein heavy chain, putative	4	0	4	non-membrane	epitope
PF14_0419	conserved Plasmodium protein, unknown function	4	3	12	non-membrane	epitope
MAL13P1,32	MORN repeat protein, putative	4	1	4	non-membrane	epitope
PFE1345c	minichromosome maintenance protein 3, putative	3	0	3	non-membrane	epitope
PF11_0240	dynein heavy chain, putative	2.75	4	11	non-membrane	epitope
PFL1445w	conserved Plasmodium protein, unknown function	2.67	3	8	non-membrane	epitope
PF10_0232	Chromodomain-helicase-DNA-binding protein 1 homolog, putative	2.5	2	5	non-membrane	epitope
MAL13P1,246	conserved Plasmodium membrane protein, unknown function	2.5	2	5	membrane	epitope
PFI1085w	ubiquitin-like protein, putative	2	0	2	non-membrane	epitope
PFE1210c	conserved Plasmodium protein, unknown function	2	0	2	non-membrane	no epitope
PFE0340c	rhomboid protease ROM4	2	4	8	membrane	epitope
PFD0855c	conserved Plasmodium protein, unknown function	2	1	2	non-membrane	no epitope
PFD0250c	Sec24-like protein, putative	2	1	2	non-membrane	no epitope
PFC0260w	P-loop containing nucleoside triphospahte hydrolase, putative	2	0	2	non-membrane	epitope
PFB0615c	conserved Plasmodium protein, unknown function	2	5	10	non-membrane	no epitope
PF14_0115	conserved Plasmodium protein, unknown function	2	0	2	non-membrane	no epitope
PF13_0126	translation initiation factor EIF-2B subunit related	2	1	2	non-membrane	no epitope
PF13_0060	conserved Plasmodium protein, unknown function	2	0	2	non-membrane	no epitope
PF11_0524	lsm4 homologue, putative	2	0	2	non-membrane	no epitope
PF11_0079	conserved Plasmodium protein, unknown function	2	1	2	membrane	epitope

PF10_0292	conserved Plasmodium protein, unknown function	2	0	2	non-membrane	epitope
PF07_0068	cysteine desulfurase, putative	2	0	2	non-membrane	no epitope
MAL8P1,60	conserved Plasmodium protein, unknown function	2	1	2	membrane	epitope
MAL8P1,12	conserved Plasmodium protein, unknown function	2	0	2	non-membrane	no epitope
PFL0350c	conserved Plasmodium protein, unknown function	1.5	2	3	non-membrane	epitope
PFD0590c	DNA polymerase alpha	1.5	2	3	non-membrane	epitope
PF14_0712	conserved Plasmodium protein, unknown function	1.5	4	6	non-membrane	epitope
PFB0095c	erythrocyte membrane protein 3	1.38	26	36	non-membrane	no epitope
PF11_0086	MIF4G domain containing protein	1.18	11	13	non-membrane	epitope
PFI1490c	large cyclophilin-like protein	1	0	1	non-membrane	epitope
PFI1345c	conserved Plasmodium protein, unknown function	1	0	1	non-membrane	no epitope
PFI0465c	conserved Plasmodium protein, unknown function	1	0	1	non-membrane	no epitope
PFI0310w	Maf-like protein, putative	1	0	1	non-membrane	epitope
PFF1095w	leucyl tRNA synthase	1	2	2	non-membrane	no epitope
PFF0265c	calcium-binding protein, putative	1	0	1	non-membrane	epitope
PFE0495w	conserved Plasmodium protein, unknown function	1	3	3	non-membrane	epitope
PFE0465c	RNA polymerase I	1	2	2	non-membrane	epitope
PFC0725c	formate-nitrate transporter, putative	1	1	1	membrane	epitope
PFB0400w	transmission-blocking target antigen s230 paralog	1	1	1	non-membrane	no epitope
PFA0535c	kinesin, putative	1	2	2	non-membrane	epitope
PF14_0648	conserved Plasmodium protein, unknown function	1	0	1	non-membrane	epitope
PF14_0504	conserved Plasmodium protein, unknown function	1	0	1	membrane	epitope
PF14_0120	conserved Plasmodium protein, unknown function	1	1	1	non-membrane	no epitope
PF13_0189	conserved Plasmodium protein, unknown function	1	0	1	non-membrane	no epitope
PF11_0390	conserved Plasmodium protein, unknown function	1	0	1	non-membrane	no epitope
PF11_0381	subtilisin-like protease 2	1	0	1	non-membrane	epitope
PF11_0243	leucine-rich repeat protein 11, LRR11	1	1	1	non-membrane	no epitope
PF11_0092	mechanosensitive ion channel protein	1	1	1	membrane	epitope
PF10_0327	Myb2 protein	1	0	1	non-membrane	no epitope
PF10_0128	WD-repeat protein, putative	1	0	1	non-membrane	no epitope
PF08_0129	serine/threonine protein phosphatase, putative	1	0	1	non-membrane	no epitope
PF08_0113	vacuolar proton translocating ATPase subunit A, putative	1	0	1	membrane	epitope
PF07_0101	conserved Plasmodium protein, unknown function	1	10	10	non-membrane	epitope
MAL8P1,47	conserved Plasmodium protein, unknown function	1	0	1	non-membrane	no epitope
MAL7P1,21	origin recognition complex subunit 2, putative	1	0	1	non-membrane	no epitope
MAL7P1,19	ubiquitin transferase, putative	1	5	5	non-membrane	epitope
MAL13P1,96	chromosome segregation protein, putative	1	0	1	non-membrane	no epitope

MAL13P1,93	conserved Plasmodium protein, unknown function	1	0	1	non-membrane	epitope
MAL13P1,69	septum formation protein MAF homologue, putative	1	0	1	non-membrane	no epitope
MAL13P1,57	conserved Plasmodium protein, unknown function	1	1	1	non-membrane	no epitope
MAL13P1,245	conserved Plasmodium protein, unknown function	1	0	1	non-membrane	no epitope
MAL13P1,22	DNA ligase I	1	0	1	membrane	epitope
PFL1370w	NIMA-related protein kinase, Pfnek-1	0.63	8	5	membrane	epitope
PF10_0224	dynein heavy chain, putative	0.5	2	1	non-membrane	epitope
MAL8P1,34	conserved Plasmodium protein, unknown function	0.5	2	1	non-membrane	epitope
PF10_0244	formin 2, putative	0	1	0	non-membrane	epitope
PFL1730c	conserved Plasmodium protein, unknown function	0	2	0	non-membrane	no epitope
PFL0445w	conserved Plasmodium protein, unknown function	0	2	0	non-membrane	no epitope
PFL0190w	ubiquitin conjugating enzyme E2, putative	0	1	0	non-membrane	no epitope
PFI1450c	conserved Plasmodium protein, unknown function	0	0	0	non-membrane	no epitope
PFF1495w	conserved Plasmodium protein, unknown function	0	0	0	non-membrane	no epitope
PFF1185w	Smarca -related protein	0	1	0	non-membrane	epitope
PFF0115c	elongation factor G, putative	0	1	0	non-membrane	no epitope
PFE0595w	prefoldin subunit, putative	0	0	0	non-membrane	no epitope
PFE0455w	phosphatase 1 regulatory subunit, putative	0	0	0	non-membrane	no epitope
PFE0450w	chromosome condensation protein, putative	0	1	0	non-membrane	epitope
PFE0415w	transcription factor IIb, putative	0	1	0	non-membrane	epitope
PFE0155w	conserved Plasmodium protein, unknown function	0	2	0	non-membrane	no epitope
PFB0720c	origin recognition complex subunit 5	0	1	0	non-membrane	no epitope
PFA0345w	centrin-1	0	1	0	non-membrane	no epitope
PF14_0492	protein phosphatase 2b regulatory subunit, putative	0	0	0	non-membrane	no epitope
PF14_0458	conserved Plasmodium protein, unknown function	0	0	0	non-membrane	no epitope
PF14_0420	calmodulin-like protein	0	0	0	non-membrane	no epitope
PF14_0366	small subunit DNA primase	0	0	0	non-membrane	no epitope
PF14_0352	ribonucleoside-diphosphate reductase, large subunit	0	0	0	non-membrane	no epitope
PF14_0235	conserved Plasmodium protein, unknown function	0	0	0	non-membrane	no epitope
PF14_0202	dynein-associated protein, putative	0	0	0	non-membrane	no epitope
PF14_0030	conserved Plasmodium protein, unknown function	0	0	0	non-membrane	no epitope
PF13_0190	conserved Plasmodium protein, unknown function	0	1	0	non-membrane	epitope
PF11_0440	conserved Plasmodium protein, unknown function	0	1	0	non-membrane	no epitope
PF11_0310	transporter, putative	0	1	0	membrane	epitope
PF11_0292	conserved Plasmodium protein, unknown function	0	0	0	non-membrane	no epitope
PF11_0287	conserved protein, unknown function	0	0	0	non-membrane	no epitope
PF11_0149	rhomboid protease ROM1, putative	0	0	0	membrane	epitope

PF11_0147	mitogen-activated protein kinase 2	0	1	0	non-membrane	no epitope
PF11_0056	conserved Plasmodium protein, unknown function	0	1	0	non-membrane	no epitope
PF10_0371	conserved Plasmodium protein, unknown function	0	0	0	non-membrane	epitope
PF10_0299	glycoprotease, putative	0	1	0	non-membrane	no epitope
PF10_0229	conserved Plasmodium protein, unknown function	0	0	0	non-membrane	no epitope
PF10_0067	conserved Plasmodium protein, unknown function	0	5	0	membrane	epitope
PF08_0100	ruvB-like DNA helicase, putative	0	2	0	non-membrane	no epitope
PF08_0094	cullin-like protein, putative	0	0	0	non-membrane	no epitope
PF07_0047	cell division cycle ATPase, putative	0	1	0	non-membrane	no epitope
MAL8P1,65	DNA helicase, putative	0	1	0	non-membrane	epitope
MAL8P1,130	conserved Plasmodium membrane protein, unknown function	0	0	0	membrane	epitope
MAL8P1,109	protein phosphatase, putative	0	0	0	non-membrane	no epitope
MAL7P1,25	cytoskeleton associated protein, putative	0	2	0	non-membrane	no epitope
MAL7P1,131	conserved Plasmodium protein, unknown function	0	0	0	non-membrane	no epitope
MAL13P1,328	DNA topoisomerase VI, B subunit, putative	0	0	0	non-membrane	no epitope
MAL13P1,299	conserved protein, unknown function	0	0	0	membrane	epitope
MAL13P1,20	conserved Plasmodium protein, unknown function	0	0	0	non-membrane	no epitope
