# Supplementary material for: Why are male malaria parasites in such a rush? Sex-specific evolution and host–parasite interactions
Source: Evol Med Public Health. 2012 Nov 26;2013(1):3–13. doi: 10.1093/emph/eos003 (PMC4183958; doi:10.1093/emph/eos003)
Supplement: Supplementary Data [file supp_eos003_suppl_data.zip › REECE_Table_S5.pdf]

Table A: dn, ds, pn, ps

Stage	Gene	Dn	Pn	Ds	Ps
**male**					
Male non membrane	MAL13P1.20	0	0	9	0
Male non membrane	MAL13P1.245	9	0	14	0
Male non membrane	MAL13P1.279	1	0	8	0
Male non membrane	MAL13P1.302	9	0	10	0
Male non membrane	MAL13P1.32	65	0	31	0
Male non membrane	MAL13P1.328	2	0	2	0
Male non membrane	MAL13P1.336	18	0	18	0
Male non membrane	MAL13P1.347	15	0	9	0
Male non membrane	MAL13P1.69	5	0	2	0
Male non membrane	MAL13P1.93	32	0	27	0
Male non membrane	MAL13P1.96	8	0	32	0
Male non membrane	MAL7P1.19	56	0	60	0
Male non membrane	MAL7P1.21	15	0	13	0
Male non membrane	MAL7P1.25	6	0	3	1
Male non membrane	MAL7P1.77	31	2	19	0
Male non membrane	MAL8P1.109	1	0	2	0
Male non membrane	MAL8P1.12	9	0	5	0
Male non membrane	MAL8P1.31	4	0	5	0
Male non membrane	MAL8P1.34	9	0	20	0
Male non membrane	MAL8P1.46	0	0	7	0
Male non membrane	MAL8P1.47	0	0	1	0
Male non membrane	MAL8P1.65	19	0	12	0
Male non membrane	PF07_0014	53	0	27	0
Male non membrane	PF07_0047	21	1	26	4
Male non membrane	PF07_0055	9	0	13	0
Male non membrane	PF07_0101	79	0	42	0
Male non membrane	PF08_0094	4	0	6	0
Male non membrane	PF08_0100	7	0	18	0
Male non membrane	PF08_0108	9	1	22	4
Male non membrane	PF08_0129	10	0	21	0
Male non membrane	PF10_0224	13	0	12	0
Male non membrane	PF10_0229	0	0	0	0
Male non membrane	PF10_0232	41	0	42	0
Male non membrane	PF10_0244	60	0	53	0
Male non membrane	PF10_0292	106	0	93	1
Male non membrane	PF10_0327	23	0	31	0
Male non membrane	PF11_0056	23	0	33	0
Male non membrane	PF11_0057	35	0	13	0
Male non membrane	PF11_0086	132	1	132	0
Male non membrane	PF11_0089	27	0	9	0
Male non membrane	PF11_0147	5	0	14	0
Male non membrane	PF11_0243	9	0	15	0
Male non membrane	PF11_0253	13	0	10	0
Male non membrane	PF11_0279	81	0	26	0
Male non membrane	PF11_0280	0	0	1	0
Male non membrane	PF11_0287	2	0	2	0
Male non membrane	PF11_0318	8	0	47	1
Male non membrane	PF11_0360	11	0	6	0
Male non membrane	PF11_0381	32	1	35	0
Male non membrane	PF11_0440	17	0	15	0
Male non membrane	PF13_0043	9	0	3	0
Male non membrane	PF13_0060	15	0	6	0
Male non membrane	PF13_0126	47	0	27	0
Male non membrane	PF13_0189	50	0	39	0
Male non membrane	PF13_0301	6	0	5	0
Male non membrane	PF13_0306	2	0	0	0
Male non membrane	PF13_0308	15	0	15	0
Male non membrane	PF14_0030	12	0	5	0
Male non membrane	PF14_0062	50	0	27	0
Male non membrane	PF14_0081	56	0	67	0
Male non membrane	PF14_0115	15	0	11	0
Male non membrane	PF14_0243a	6	0	11	0
Male non membrane	PF14_0280	2	0	2	0
Male non membrane	PF14_0287	8	0	4	0

Male non membrane	PF14_0309	6	0	6	0
Male non membrane	PF14_0332	14	0	7	0
Male non membrane	PF14_0352	2	0	30	0
Male non membrane	PF14_0366	1	0	1	0
Male non membrane	PF14_0384	4	0	6	0
Male non membrane	PF14_0419	9	0	9	0
Male non membrane	PF14_0420	2	0	3	0
Male non membrane	PF14_0458	5	0	5	0
Male non membrane	PF14_0560	3	0	7	0
Male non membrane	PF14_0626	13	0	15	0
Male non membrane	PF14_0648	51	0	31	0
Male non membrane	PFA0535c	25	0	37	0
Male non membrane	PFB0095c	141	0	27	0
Male non membrane	PFB0250w	10	0	26	0
Male non membrane	PFB0400w	98	0	98	0
Male non membrane	PFB0405w	26	0	32	0
Male non membrane	PFB0720c	22	0	34	0
Male non membrane	PFC0135c	9	0	31	0
Male non membrane	PFC0260w	6	0	9	0
Male non membrane	PFC0305w	11	0	9	0
Male non membrane	PFC0595c	2	0	10	0
Male non membrane	PFC0720w	13	0	13	0
Male non membrane	PFD0250c	24	1	27	0
Male non membrane	PFD0285c	99	2	99	2
Male non membrane	PFD0420c	20	0	26	0
Male non membrane	PFD0525w	2	0	7	0
Male non membrane	PFD0590c	17	0	7	0
Male non membrane	PFD0855c	31	0	4	0
Male non membrane	PFD0875c	46	1	49	0
Male non membrane	PFD0905w	8	0	10	0
Male non membrane	PFE0090w	11	1	17	1
Male non membrane	PFE0155w	17	0	9	0
Male non membrane	PFE0285c	1	0	1	0
Male non membrane	PFE0415w	11	0	10	1
Male non membrane	PFE0450w	38	0	37	0
Male non membrane	PFE0455w	0	0	3	0
Male non membrane	PFE0465c	31	0	32	2
Male non membrane	PFE0495w	7	0	11	0
Male non membrane	PFE0520c	11	0	8	0
Male non membrane	PFE0540w	4	0	7	0
Male non membrane	PFE1175w	7	0	10	0
Male non membrane	PFE1210c	20	1	7	0
Male non membrane	PFE1345c	6	0	31	0
Male non membrane	PFF0115c	6	0	14	0
Male non membrane	PFF0375c	11	0	7	0
Male non membrane	PFF0500c	4	0	9	0
Male non membrane	PFF0750w	17	0	57	0
Male non membrane	PFF1095w	65	0	118	0
Male non membrane	PFF1150w	2	0	6	0
Male non membrane	PFF1185w	32	0	41	0
Male non membrane	PFF1285w	31	0	22	0
Male non membrane	PFF1470c	17	0	49	0
Male non membrane	PFF1495w	7	1	9	0
Male non membrane	PFI0260c	21	0	64	0
Male non membrane	PFI0275w	27	0	20	1
Male non membrane	PFI0310w	6	0	9	0
Male non membrane	PFI0465c	16	0	10	0
Male non membrane	PFI0745w	9	0	12	0
Male non membrane	PFI1080w	16	0	15	0
Male non membrane	PFI1085w	15	0	10	0
Male non membrane	PFI1165c	4	0	3	0
Male non membrane	PFI1345c	23	0	19	0
Male non membrane	PFI1350c	2	0	1	0
Male non membrane	PFL0150w	8	0	20	0
Male non membrane	PFL0190w	0	0	5	0
Male non membrane	PFL0350c	36	0	16	0
Male non membrane	PFL0445w	5	0	25	0
Male non membrane	PFL0580w	1	0	22	0
Male non membrane	PFL0660w	10	0	0	0
Male non membrane	PFL1180w	0	0	2	0

Male non membrane	PFL1445w	70	0	29	0
Male non membrane	PFL1730c	49	0	24	0
Male non membrane	PFL1980c	9	0	13	0
Male non membrane	PFL2095w	2	0	1	0
Male non membrane	PFL2190c	15	0	8	0
Male membrane	MAL13P1.172	7	0	12	1
Male membrane	MAL13P1.177	3	0	3	0
Male membrane	MAL13P1.22	14	0	33	0
Male membrane	MAL13P1.246	32	0	37	0
Male membrane	MAL13P1.271	1	0	7	0
Male membrane	MAL13P1.299	2	0	8	0
Male membrane	MAL8P1.130	18	0	11	0
Male membrane	MAL8P1.27	1	0	2	0
Male membrane	MAL8P1.60	11	0	5	0
Male membrane	PF07_0087	7	0	19	0
Male membrane	PF08_0113	30	0	86	0
Male membrane	PF10_0067	4	0	11	0
Male membrane	PF11_0079	47	0	18	0
Male membrane	PF11_0092	49	0	44	0
Male membrane	PF11_0310	27	1	27	0
Male membrane	PF13_0252	7	0	16	0
Male membrane	PF14_0596	8	0	20	0
Male membrane	PFB0460c	91	1	43	0
Male membrane	PFC0725c	6	0	14	0
Male membrane	PFD1110w	1	0	10	0
Male membrane	PFE0340c	29	0	32	0
Male membrane	PFE0555w	13	1	11	0
Male membrane	PFE0965c	6	0	7	0
Male membrane	PFL1370w	3	0	6	0
**Female**					
Female non membrane	MAL13P1.164	18	0	20	0
Female non membrane	MAL13P1.262	41	0	14	0
Female non membrane	MAL13P1.83	15	0	12	0
Female non membrane	MAL7P1.100	0	0	1	0
Female non membrane	MAL7P1.162	6	0	15	0
Female non membrane	MAL7P1.88	10	0	7	0
Female non membrane	MAL8P1.85	5	0	8	0
Female non membrane	MAL8P1.96	12	0	5	0
Female non membrane	PF08_0126	30	0	54	0
Female non membrane	PF08_0132	21	0	28	1
Female non membrane	PF10_0031	34	0	19	0
Female non membrane	PF10_0040	57	0	39	0
Female non membrane	PF10_0144	11	0	9	0
Female non membrane	PF10_0245	10	1	23	0
Female non membrane	PF11_0043	0	0	3	0
Female non membrane	PF11_0097	3	0	7	0
Female non membrane	PF11_0145	5	0	9	0
Female non membrane	PF11_0317	74	0	80	0
Female non membrane	PF13_0121	8	0	10	0
Female non membrane	PF13_0346	0	0	1	0
Female non membrane	PF14_0174	2	0	13	0
Female non membrane	PF14_0282	13	0	32	0
Female non membrane	PF14_0288	1	0	5	0
Female non membrane	PF14_0327	7	0	31	0
Female non membrane	PF14_0334	69	0	81	0
Female non membrane	PF14_0349	2	0	2	0
Female non membrane	PF14_0477	0	0	32	0
Female non membrane	PF14_0529	25	0	48	1
Female non membrane	PF14_0538	35	0	44	0
Female non membrane	PF14_0586	3	0	9	0
Female non membrane	PF14_0646	0	0	1	0
Female non membrane	PFA0190c	1	0	16	0
Female non membrane	PFA0445w	15	0	23	0
Female non membrane	PFB0500c	3	0	2	0
Female non membrane	PFB0885w	0	0	0	0
Female non membrane	PFC0500w	21	0	18	0
Female non membrane	PFC0535w	1	0	7	0
Female non membrane	PFC0775w	0	0	2	0
Female non membrane	PFD0470c	22	0	52	0
Female non membrane	PFD0530c	14	0	9	0

Female non membrane	PFD0705c	5	2	7	0
Female non membrane	PFE0690c	4	0	21	0
Female non membrane	PFE1400c	5	0	49	0
Female non membrane	PFF0095c	41	0	38	0
Female non membrane	PFF0765c	27	0	19	0
Female non membrane	PFI0315c	38	0	16	0
Female non membrane	PFI0365w	7	0	6	0
Female non membrane	PFI0655c	4	0	7	0
Female non membrane	PFI0735c	8	0	33	0
Female non membrane	PFI1330c	3	0	4	0
Female non membrane	PFI1365w	4	0	19	1
Female non membrane	PFI1435w	16	0	6	0
Female non membrane	PFL0955c	3	0	1	0
Female non membrane	PFL0965c	3	0	12	0
Female non membrane	PFL1555w	3	0	2	0
Female non membrane	PFL1630c	5	0	7	0
Female non membrane	PFL1685w	1	0	4	0
Female membrane	PF13_0248	3	2	4	0
Female membrane	PF14_0067	21	0	43	1
Female membrane	PF14_0375	5	0	4	0
Female membrane	PF14_0617	5	0	5	0
Female membrane	PF14_0723	9	0	31	0
Female membrane	PFC0381c	16	0	5	0
Female membrane	PFD1035w	7	0	3	0
Female membrane	PFE0645w	5	0	7	0
Female membrane	PFE1340w	2	0	5	0
Female membrane	PFF0170w	6	0	21	0
Female membrane	PFF1265w	3	0	10	0
Female membrane	PFI0385c	11	0	14	0
Female membrane	PFI0935w	2	0	3	0
Female membrane	PFI1370c	6	0	28	0
Female membrane	PFL0595c	6	0	2	0
Female membrane	PFL0655w	2	0	4	0
Female membrane	PFL2405c	64	0	84	0
**Asexual blood stages**					
Asexual blood stages non membrane	MAL13P1.121	29	1	22	0
Asexual blood stages non membrane	MAL13P1.146	11	0	41	1
Asexual blood stages non membrane	MAL13P1.19	87	0	28	0
Asexual blood stages non membrane	MAL13P1.221	12	0	10	0
Asexual blood stages non membrane	MAL13P1.33	21	0	6	0
Asexual blood stages non membrane	MAL13P1.341	8	0	8	0
Asexual blood stages non membrane	MAL13P1.344	8	0	44	0
Asexual blood stages non membrane	MAL7P1.119	27	0	23	0
Asexual blood stages non membrane	MAL7P1.126	65	0	38	0
Asexual blood stages non membrane	MAL8P1.55	40	0	30	0
Asexual blood stages non membrane	MAL8P1.62	18	0	10	0
Asexual blood stages non membrane	MAL8P1.73	57	0	48	0
Asexual blood stages non membrane	PF07_0057	1	0	5	0
Asexual blood stages non membrane	PF08_0063	10	0	13	0
Asexual blood stages non membrane	PF08_0086	31	0	23	0
Asexual blood stages non membrane	PF10_0028-b	3	0	2	0
Asexual blood stages non membrane	PF10_0039	0	0	3	0
Asexual blood stages non membrane	PF10_0085	6	0	18	0
Asexual blood stages non membrane	PF10_0087	1	0	10	0
Asexual blood stages non membrane	PF10_0093	18	1	11	0
Asexual blood stages non membrane	PF10_0099	41	0	46	0
Asexual blood stages non membrane	PF10_0166	7	0	7	0
Asexual blood stages non membrane	PF10_0217	8	0	7	0
Asexual blood stages non membrane	PF10_0268	14	0	13	0
Asexual blood stages non membrane	PF10_0272	3	0	12	0
Asexual blood stages non membrane	PF10_0306	4	0	21	0
Asexual blood stages non membrane	PF10_0331	13	0	15	0
Asexual blood stages non membrane	PF11_0047	5	0	8	0
Asexual blood stages non membrane	PF11_0114	17	0	20	0
Asexual blood stages non membrane	PF11_0168	57	0	28	0
Asexual blood stages non membrane	PF11_0171	10	1	16	0
Asexual blood stages non membrane	PF11_0189	10	0	13	0
Asexual blood stages non membrane	PF11_0212	13	0	12	0
Asexual blood stages non membrane	PF11_0268	17	0	2	0
Asexual blood stages non membrane	PF11_0295	15	0	8	0

Asexual blood stages non membrane	PF11_0362	54	2	15	0
Asexual blood stages non membrane	PF13_0099	14	0	4	0
Asexual blood stages non membrane	PF13_0137	18	0	16	0
Asexual blood stages non membrane	PF13_0198	102	0	29	0
Asexual blood stages non membrane	PF13_0219	7	0	8	0
Asexual blood stages non membrane	PF13_0233	8	0	20	0
Asexual blood stages non membrane	PF13_0234	4	0	15	0
Asexual blood stages non membrane	PF13_0238	3	0	21	0
Asexual blood stages non membrane	PF13_0315	1	0	14	0
Asexual blood stages non membrane	PF13_0323	22	0	22	0
Asexual blood stages non membrane	PF13_0324	16	1	30	0
Asexual blood stages non membrane	PF13_0350	6	0	25	0
Asexual blood stages non membrane	PF14_0020	4	0	15	0
Asexual blood stages non membrane	PF14_0038	0	0	2	0
Asexual blood stages non membrane	PF14_0064	2	0	2	0
Asexual blood stages non membrane	PF14_0068	2	0	13	0
Asexual blood stages non membrane	PF14_0088	64	0	12	0
Asexual blood stages non membrane	PF14_0102	50	2	17	0
Asexual blood stages non membrane	PF14_0224	10	0	10	0
Asexual blood stages non membrane	PF14_0261	2	0	9	1
Asexual blood stages non membrane	PF14_0277	7	0	33	0
Asexual blood stages non membrane	PF14_0344	6	0	4	0
Asexual blood stages non membrane	PF14_0346	4	0	21	0
Asexual blood stages non membrane	PF14_0360	0	0	19	0
Asexual blood stages non membrane	PF14_0428	18	0	23	1
Asexual blood stages non membrane	PF14_0476	3	0	6	0
Asexual blood stages non membrane	PF14_0527	24	0	14	0
Asexual blood stages non membrane	PFA0260c	1	0	3	0
Asexual blood stages non membrane	PFA0440w	2	0	1	0
Asexual blood stages non membrane	PFB0200c	12	0	13	0
Asexual blood stages non membrane	PFB0355c	87	1	44	0
Asexual blood stages non membrane	PFB0490c	2	0	8	0
Asexual blood stages non membrane	PFB0640c	45	1	57	0
Asexual blood stages non membrane	PFB0745w	48	0	22	1
Asexual blood stages non membrane	PFC0340w	14	0	19	0
Asexual blood stages non membrane	PFC0365w	8	0	8	0
Asexual blood stages non membrane	PFC0400w	1	0	2	0
Asexual blood stages non membrane	PFC0950c	85	0	54	0
Asexual blood stages non membrane	PFD0515w	6	0	10	0
Asexual blood stages non membrane	PFD0605c	4	0	14	0
Asexual blood stages non membrane	PFD0725c	4	0	7	0
Asexual blood stages non membrane	PFD0735c	2	0	5	0
Asexual blood stages non membrane	PFD1070w	5	0	15	0
Asexual blood stages non membrane	PFE0255w	9	0	12	0
Asexual blood stages non membrane	PFE0375w	15	0	45	0
Asexual blood stages non membrane	PFE0605c	5	0	3	0
Asexual blood stages non membrane	PFE0865c	1	0	9	0
Asexual blood stages non membrane	PFE1085w	2	0	14	0
Asexual blood stages non membrane	PFE1285w	3	0	8	0
Asexual blood stages non membrane	PFF0625w	15	0	40	2
Asexual blood stages non membrane	PFF0675c	127	0	65	0
Asexual blood stages non membrane	PFF0950w	16	2	6	1
Asexual blood stages non membrane	PFF1055c	33	1	25	0
Asexual blood stages non membrane	PFF1350c	9	1	14	2
Asexual blood stages non membrane	PFI0190w	1	0	3	0
Asexual blood stages non membrane	PFI0195c	10	0	6	0
Asexual blood stages non membrane	PFI0820c	5	0	14	0
Asexual blood stages non membrane	PFI1130c	5	0	8	0
Asexual blood stages non membrane	PFI1685w	0	0	6	0
Asexual blood stages non membrane	PFI1700c	22	0	21	0
Asexual blood stages non membrane	PFL0035c	186	4	109	0
Asexual blood stages non membrane	PFL0335c	18	0	10	0
Asexual blood stages non membrane	PFL1065c	7	0	10	0
Asexual blood stages non membrane	PFL1480w	24	0	27	0
Asexual blood stages non membrane	PFL1490w	37	0	26	0
Asexual blood stages non membrane	PFL1530w	28	0	8	0
Asexual blood stages non membrane	PFL1605w	66	0	41	0
Asexual blood stages non membrane	PFL1785c	9	0	14	0
Asexual blood stages non membrane	PFL2100w	8	5	8	2
Asexual blood stages non membrane	PFL2225w	3	0	7	0

Asexual blood stages non membrane	PFL2230c	17	0	15	1
Asexual blood stages non membrane	PFL2245w	2	0	8	0
Asexual blood stages non membrane	PFL2250c	38	0	42	0
Asexual blood stages non membrane	PFL2355w	19	0	14	0
Asexual blood stages membrane	PF14_0325	6	0	9	0
Asexual blood stages membrane	PF14_0495	7	0	12	0
Asexual blood stages membrane	PF14_0530	44	0	36	0
Asexual blood stages membrane	PF14_0660	8	0	7	0
Asexual blood stages membrane	PFD0240c	32	0	19	0
Asexual blood stages membrane	PFD0720w	2	0	1	0
Asexual blood stages membrane	PFE1445c	4	2	33	3
Asexual blood stages membrane	PFI1475w	36	16	17	1
Asexual blood stages membrane	PFL0410w	37	0	17	0
Asexual blood stages membrane	PFL1700c	7	0	10	0
Asexual blood stages membrane	PFL2505c	28	0	15	0
Asexual blood stages membrane	MAL13P1.309	8	0	3	0
Asexual blood stages membrane	MAL13P1.39	4	0	5	0
Asexual blood stages membrane	MAL13P1.60	100	0	26	0
Asexual blood stages membrane	MAL8P1.53	2	0	5	0
Asexual blood stages membrane	PF10_0363	17	0	30	0
Asexual blood stages membrane	PF11_0067	69	0	95	0
Asexual blood stages membrane	PF11_0107	72	0	39	0
Asexual blood stages membrane	PF11_0112	8	0	18	0
Asexual blood stages membrane	PF11_0203	33	0	34	1
Asexual blood stages membrane	PF11_0246	34	0	12	1
Asexual blood stages membrane	PF11_0344	49	26	20	2
Asexual blood stages membrane	PF13_0102	11	0	29	0
Asexual blood stages membrane	PF13_0116	10	0	17	0
Asexual blood stages membrane	PF13_0133	6	0	2	0
Asexual blood stages membrane	PF13_0265	69	0	28	0
Asexual blood stages membrane	PF13_0270	14	0	2	0
Asexual blood stages membrane	PF14_0065	0	0	5	0
**All stages**					
All stages non-membrane	MAL13P1.135	1	0	8	0
All stages non-membrane	MAL13P1.190	5	0	15	0
All stages non-membrane	MAL13P1.233	9	0	4	0
All stages non-membrane	MAL13P1.237	3	0	10	0
All stages non-membrane	MAL13P1.270	2	0	4	0
All stages non-membrane	MAL13P1.283	0	0	7	0
All stages non-membrane	MAL13P1.92	0	0	7	0
All stages non-membrane	MAL7P1.122	2	0	10	0
All stages non-membrane	MAL7P1.81	0	0	16	1
All stages non-membrane	MAL8P1.125	8	0	11	0
All stages non-membrane	MAL8P1.17	4	0	25	0
All stages non-membrane	MAL8P1.40	3	0	5	0
All stages non-membrane	PF07_0029	6	0	44	0
All stages non-membrane	PF07_0033	13	0	33	0
All stages non-membrane	PF07_0072	1	0	32	0
All stages non-membrane	PF07_0073	10	0	14	0
All stages non-membrane	PF07_0079	1	0	7	0
All stages non-membrane	PF07_0080	1	0	1	0
All stages non-membrane	PF07_0112	2	0	21	0
All stages non-membrane	PF07_0117	4	0	18	0
All stages non-membrane	PF08_0019	0	0	4	0
All stages non-membrane	PF08_0071	1	0	3	0
All stages non-membrane	PF08_0074	0	0	1	1
All stages non-membrane	PF08_0075	0	0	3	0
All stages non-membrane	PF08_0081	1	0	8	0
All stages non-membrane	PF08_0087	1	0	9	0
All stages non-membrane	PF08_0096	9	0	20	1
All stages non-membrane	PF08_0110	3	0	7	0
All stages non-membrane	PF08_0115	39	0	39	0
All stages non-membrane	PF10_0063	2	0	5	0
All stages non-membrane	PF10_0068	0	0	9	0
All stages non-membrane	PF10_0077	17	0	44	0
All stages non-membrane	PF10_0081	2	0	13	0
All stages non-membrane	PF10_0084	0	0	9	0
All stages non-membrane	PF10_0086	1	0	8	0
All stages non-membrane	PF10_0111	2	0	1	0
All stages non-membrane	PF10_0115	31	0	20	0

All stages non-membrane	PF10_0123	8	0	22	0
All stages non-membrane	PF10_0149	1	0	8	0
All stages non-membrane	PF10_0153	1	0	19	0
All stages non-membrane	PF10_0174	31	0	33	0
All stages non-membrane	PF10_0187	0	0	1	0
All stages non-membrane	PF10_0203	0	0	4	0
All stages non-membrane	PF10_0210	10	0	7	0
All stages non-membrane	PF10_0264	5	0	9	0
All stages non-membrane	PF10_0289	4	0	10	0
All stages non-membrane	PF10_0325	3	0	5	0
All stages non-membrane	PF10_0340	7	0	14	0
All stages non-membrane	PF11_0142	6	0	11	0
All stages non-membrane	PF11_0177	16	0	19	0
All stages non-membrane	PF11_0183	0	0	6	0
All stages non-membrane	PF11_0188	37	0	38	0
All stages non-membrane	PF11_0208	4	0	5	0
All stages non-membrane	PF11_0250	1	0	12	0
All stages non-membrane	PF11_0270	39	0	65	0
All stages non-membrane	PF11_0293	0	0	2	0
All stages non-membrane	PF11_0302	26	0	28	0
All stages non-membrane	PF11_0312	1	0	1	0
All stages non-membrane	PF11_0313	5	0	18	0
All stages non-membrane	PF11_0314	1	0	9	0
All stages non-membrane	PF11_0331	1	0	15	0
All stages non-membrane	PF11_0351	1	0	0	0
All stages non-membrane	PF11_0374	6	0	9	0
All stages non-membrane	PF11_0375	25	0	18	0
All stages non-membrane	PF11_0396	18	1	16	0
All stages non-membrane	PF11_0437	2	0	0	0
All stages non-membrane	PF11_0461	0	0	4	0
All stages non-membrane	PF11_0465	2	0	43	0
All stages non-membrane	PF13_0044	56	0	48	0
All stages non-membrane	PF13_0065	1	0	10	0
All stages non-membrane	PF13_0143	18	0	11	0
All stages non-membrane	PF13_0213	3	0	9	0
All stages non-membrane	PF13_0224	1	0	7	0
All stages non-membrane	PF13_0228	0	0	6	0
All stages non-membrane	PF13_0229	22	0	67	0
All stages non-membrane	PF13_0232	8	0	23	0
All stages non-membrane	PF13_0242	3	0	1	0
All stages non-membrane	PF13_0262	15	0	42	0
All stages non-membrane	PF13_0268	2	0	7	0
All stages non-membrane	PF13_0269	7	0	19	0
All stages non-membrane	PF13_0282	2	0	5	0
All stages non-membrane	PF13_0287	0	0	12	0
All stages non-membrane	PF13_0305	0	0	25	0
All stages non-membrane	PF13_0316	1	0	4	0
All stages non-membrane	PF13_0322	13	0	13	0
All stages non-membrane	PF13_0349	0	0	6	0
All stages non-membrane	PF14_0053	0	0	10	0
All stages non-membrane	PF14_0083	7	0	4	0
All stages non-membrane	PF14_0104	4	0	14	0
All stages non-membrane	PF14_0141	5	0	5	0
All stages non-membrane	PF14_0146	8	0	7	0
All stages non-membrane	PF14_0164	5	0	18	0
All stages non-membrane	PF14_0192	3	0	9	0
All stages non-membrane	PF14_0196	18	1	11	0
All stages non-membrane	PF14_0231	1	0	3	0
All stages non-membrane	PF14_0242	7	0	6	0
All stages non-membrane	PF14_0296	2	0	3	0
All stages non-membrane	PF14_0301	3	0	5	0
All stages non-membrane	PF14_0321	17	0	6	0
All stages non-membrane	PF14_0324	7	0	29	0
All stages non-membrane	PF14_0359	4	0	14	0
All stages non-membrane	PF14_0368	1	0	4	0
All stages non-membrane	PF14_0391	0	0	1	0
All stages non-membrane	PF14_0393	1	0	9	0
All stages non-membrane	PF14_0401	7	0	12	0
All stages non-membrane	PF14_0425	11	0	2	0
All stages non-membrane	PF14_0439	9	0	6	0

All stages non-membrane	PF14_0448	0	0	15	0
All stages non-membrane	PF14_0486	4	0	18	0
All stages non-membrane	PF14_0511	26	0	19	0
All stages non-membrane	PF14_0520	6	0	13	0
All stages non-membrane	PF14_0585	0	0	0	0
All stages non-membrane	PF14_0598	26	0	16	0
All stages non-membrane	PF14_0615	1	1	5	0
All stages non-membrane	PF14_0627	1	0	6	0
All stages non-membrane	PF14_0632	20	0	16	0
All stages non-membrane	PF14_0655	2	0	7	0
All stages non-membrane	PF14_0676	0	0	5	0
All stages non-membrane	PF14_0716	11	0	9	0
All stages non-membrane	PFA0145c	10	1	22	0
All stages non-membrane	PFA0230c	12	0	4	0
All stages non-membrane	PFA0400c	2	0	7	0
All stages non-membrane	PFA0520c	3	0	14	0
All stages non-membrane	PFB0260w	1	0	5	0
All stages non-membrane	PFB0445c	5	0	13	0
All stages non-membrane	PFB0525w	15	0	16	0
All stages non-membrane	PFB0635w	9	0	34	0
All stages non-membrane	PFB0795w	19	0	31	0
All stages non-membrane	PFB0815w	2	0	31	0
All stages non-membrane	PFB0840w	0	0	7	0
All stages non-membrane	PFC0190c	0	0	11	0
All stages non-membrane	PFC0285c	5	0	47	0
All stages non-membrane	PFC0290w	1	0	8	0
All stages non-membrane	PFC0300c	3	0	4	0
All stages non-membrane	PFC0350c	3	0	39	0
All stages non-membrane	PFC0395w	6	0	13	0
All stages non-membrane	PFC0520w	60	0	32	0
All stages non-membrane	PFC0635c	0	0	1	0
All stages non-membrane	PFC0735w	0	0	5	0
All stages non-membrane	PFC0745c	3	0	8	0
All stages non-membrane	PFC0870w	1	1	4	0
All stages non-membrane	PFC0900w	4	0	13	0
All stages non-membrane	PFC0975c	0	0	2	0
All stages non-membrane	PFC1020c	2	0	5	0
All stages non-membrane	PFD0305c	0	0	33	0
All stages non-membrane	PFD0830w	11	0	7	0
All stages non-membrane	PFD0950w	5	0	7	0
All stages non-membrane	PFE0545c	2	0	1	0
All stages non-membrane	PFE0660c	5	0	9	0
All stages non-membrane	PFE0885w	6	0	34	0
All stages non-membrane	PFE0975c	0	1	6	0
All stages non-membrane	PFE1005w	1	0	7	0
All stages non-membrane	PFE1050w	2	0	10	0
All stages non-membrane	PFE1195w	2	0	30	1
All stages non-membrane	PFE1250w	2	3	8	0
All stages non-membrane	PFE1370w	11	0	35	0
All stages non-membrane	PFF0250w	5	0	22	0
All stages non-membrane	PFF0325c	124	0	68	0
All stages non-membrane	PFF0340c	9	0	10	0
All stages non-membrane	PFF0420c	0	0	0	0
All stages non-membrane	PFF0430w	3	0	29	0
All stages non-membrane	PFF0435w	28	0	10	0
All stages non-membrane	PFF0530w	6	0	14	0
All stages non-membrane	PFF0885w	1	0	5	0
All stages non-membrane	PFF0895w	9	0	14	0
All stages non-membrane	PFF0940c	1	0	13	0
All stages non-membrane	PFF1155w	1	0	7	0
All stages non-membrane	PFF1300w	8	0	10	0
All stages non-membrane	PFF1345w	27	1	14	0
All stages non-membrane	PFF1410c	28	0	34	0
All stages non-membrane	PFI0155c	2	0	4	0
All stages non-membrane	PFI0165c	21	0	41	0
All stages non-membrane	PFI0370c	2	0	19	0
All stages non-membrane	PFI0490c	1	0	15	0
All stages non-membrane	PFI0630w	4	0	10	0
All stages non-membrane	PFI0755c	7	0	14	0
All stages non-membrane	PFI0895c	4	0	12	0

All stages non-membrane	PFI0930c	13	0	1	0
All stages non-membrane	PFI1020c	3	0	20	0
All stages non-membrane	PFI1105w	19	0	1	0
All stages non-membrane	PFI1170c	1	0	9	0
All stages non-membrane	PFI1175c	8	0	6	0
All stages non-membrane	PFI1270w	1	0	12	0
All stages non-membrane	PFI1310w	9	0	18	0
All stages non-membrane	PFI1445w	30	0	26	0
All stages non-membrane	PFL0110c	14	0	13	0
All stages non-membrane	PFL0185c	1	0	11	0
All stages non-membrane	PFL0210c	1	0	10	0
All stages non-membrane	PFL0280c	4	0	5	0
All stages non-membrane	PFL0310c	13	0	19	0
All stages non-membrane	PFL0625c	6	0	27	0
All stages non-membrane	PFL0670c	10	0	20	0
All stages non-membrane	PFL0725w	1	0	6	0
All stages non-membrane	PFL0895c	8	1	62	0
All stages non-membrane	PFL0900c	19	0	18	0
All stages non-membrane	PFL0930w	14	0	16	0
All stages non-membrane	PFL1070c	1	0	8	0
All stages non-membrane	PFL1110c	6	0	6	0
All stages non-membrane	PFL1170w	8	0	8	0
All stages non-membrane	PFL1245w	18	0	37	0
All stages non-membrane	PFL1270w	1	0	12	0
All stages non-membrane	PFL1420w	7	0	6	0
All stages non-membrane	PFL1425w	9	0	36	2
All stages non-membrane	PFL1465c	3	0	6	0
All stages non-membrane	PFL1550w	17	0	22	0
All stages non-membrane	PFL1725w	5	0	6	0
All stages non-membrane	PFL2005w	0	0	3	0
All stages non-membrane	PFL2060c	5	0	19	0
All stages non-membrane	PFL2215w	3	0	11	0
All stages non-membrane	PFL2275c	2	0	5	0
All stages non-membrane	PFL2345c	6	0	11	0
All stages membrane	MAL13P1.56	16	0	60	0
All stages membrane	MAL8P1.128	1	0	2	0
All stages membrane	PF08_0031	2	0	9	0
All stages membrane	PF10_0366	1	1	4	0
All stages membrane	PF11_0055	5	0	9	0
All stages membrane	PF11_0098	6	0	9	0
All stages membrane	PF11_0164	6	0	0	0
All stages membrane	PF11_0174	8	1	30	0
All stages membrane	PF11_0301	2	0	12	0
All stages membrane	PF11_0352	8	0	3	0
All stages membrane	PF13_0119	0	0	2	0
All stages membrane	PF13_0141	39	0	7	0
All stages membrane	PF14_0075	10	0	22	0
All stages membrane	PF14_0230	6	0	9	0
All stages membrane	PF14_0678	7	0	4	0
All stages membrane	PFA0310c	14	0	39	0
All stages membrane	PFB0210c	5	0	43	0
All stages membrane	PFC0275w	3	0	22	0
All stages membrane	PFD0660w	12	0	0	0
All stages membrane	PFE0850c	4	0	10	0
All stages membrane	PFE1405c	16	0	33	0
All stages membrane	PFI0235w	1	0	6	0
All stages membrane	PFI0875w	5	0	25	0
All stages membrane	PFL0590c	14	0	16	1
All stages membrane	PFL0865w	5	0	3	0
All stages membrane	PFL1835w	34	0	16	0
All stages membrane	PFL1845c	2	0	9	0
