# Supplementary material for: Why are male malaria parasites in such a rush? Sex-specific evolution and host–parasite interactions
Source: Evol Med Public Health. 2012 Nov 26;2013(1):3–13. doi: 10.1093/emph/eos003 (PMC4183958; doi:10.1093/emph/eos003)
Supplement: Supplementary Data [file supp_eos003_suppl_data.zip › REECE_Table_S6.pdf]

Pb/Py Table A: dn/ds for stage-specific sets

Set	Number of genes	Mean dn/ds	CI 95%	CI 95%
Male	227	0.1947	0.1776	0.2134
Female	97	0.1529	0.1336	0.1743
Expressed in 3 stages	297	0.0927	0.0818	0.1045
Asexual blood stages	165	0.2373	0.2109	0.2658

Pb/Py Table B: dn/ds comparisons for stage-specific sets

Set 1	Mean dn/ds	Set 2	Mean dn/ds	Pvalue Set 1 > Set 2
Asexual blood stages	0.2373	Asexual blood stages	0.2373	0.4993
Asexual blood stages	0.2373	Male	0.1947	0.9831
Asexual blood stages	0.2373	Female	0.1529	1
Asexual blood stages	0.2373	Expressed in 3 stages	0.0927	1
Male	0.1947	Asexual blood stages	0.2373	0.0142
Male	0.1947	Male	0.1947	0.5092
Male	0.1947	Female	0.1529	0.9924
Male	0.1947	Expressed in 3 stages	0.0927	1
Female	0.1529	Asexual blood stages	0.2373	0
Female	0.1529	Male	0.1947	0.0055
Female	0.1529	Female	0.1529	0.5044
Female	0.1529	Expressed in 3 stages	0.0927	1
Expressed in 3 stages	0.0927	Asexual blood stages	0.2373	0
Expressed in 3 stages	0.0927	Male	0.1947	0
Expressed in 3 stages	0.0927	Female	0.1529	0
Expressed in 3 stages	0.0927	Expressed in 3 stages	0.0927	0.5048

Pb/Py Table C: dn/ds for all sets

Set	Number of genes	Mean dn/ds	CI 95%	CI 95%
Membrane male	31	0.2897	0.2040	0.3876
Male non membrane	196	0.1796	0.1659	0.1937
Membrane Female	26	0.2231	0.1726	0.2798
Female non membrane	71	0.1272	0.1096	0.1450
Membrane expressed in 3 stages	35	0.2074	0.1496	0.2714
Expressed in 3 stages non membra	262	0.0773	0.0688	0.0869
Membrane asexual	55	0.3260	0.2702	0.3842
Asexual non membrane	110	0.1930	0.1666	0.2215

Pb/Py Table D: dn/ds comparisons for all sets

Set 1	Mean dn/ds	Set 2	Mean dn/ds	Pvalue Set 1 > Set 2
Asexual blood stages non membra	0.193	Asexual blood :	0.193	0.5006
Asexual blood stages non membra	0.193	Asexual blood :	0.326	0.0001
Asexual blood stages non membra	0.193	Male non mem	0.1796	0.7551
Asexual blood stages non membra	0.193	Male membran	0.2897	0.0399
Asexual blood stages non membra	0.193	Female non me	0.1272	0.9993
Asexual blood stages non membra	0.193	Female membr	0.2231	0.2117
Asexual blood stages non membra	0.193	Expressed in 3	0.0773	1
Asexual blood stages non membra	0.193	Expressed in 3	0.2074	0.3783
Asexual blood stages membrane	0.326	Asexual blood :	0.193	0.9999
Asexual blood stages membrane	0.326	Asexual blood :	0.326	0.5031
Asexual blood stages membrane	0.326	Male non mem	0.1796	1
Asexual blood stages membrane	0.326	Male membran	0.2897	0.7108
Asexual blood stages membrane	0.326	Female non me	0.1272	1
Asexual blood stages membrane	0.326	Female membr	0.2231	0.9839
Asexual blood stages membrane	0.326	Expressed in 3	0.0773	1

Asexual blood stages membrane	0.326	Expressed in 3	0.2074	0.9898
Male non membrane	0.1796	Asexual blood :	0.193	0.2451
Male non membrane	0.1796	Asexual blood :	0.326	0
Male non membrane	0.1796	Male non mem	0.1796	0.5006
Male non membrane	0.1796	Male membran	0.2897	0.0135
Male non membrane	0.1796	Female non me	0.1272	0.9999
Male non membrane	0.1796	Female membr	0.2231	0.09
Male non membrane	0.1796	Expressed in 3	0.0773	1
Male non membrane	0.1796	Expressed in 3	0.2074	0.24
Male membrane	0.2897	Asexual blood :	0.193	0.9636
Male membrane	0.2897	Asexual blood :	0.326	0.2842
Male membrane	0.2897	Male non mem	0.1796	0.9887
Male membrane	0.2897	Male membran	0.2897	0.4958
Male membrane	0.2897	Female non me	0.1272	1
Male membrane	0.2897	Female membr	0.2231	0.8426
Male membrane	0.2897	Expressed in 3	0.0773	1
Male membrane	0.2897	Expressed in 3	0.2074	0.8943
Female non membrane	0.1272	Asexual blood :	0.193	0.0002
Female non membrane	0.1272	Asexual blood :	0.326	0
Female non membrane	0.1272	Male non mem	0.1796	0.0001
Female non membrane	0.1272	Male membran	0.2897	0.0001
Female non membrane	0.1272	Female non me	0.1272	0.4947
Female non membrane	0.1272	Female membr	0.2231	0.0003
Female non membrane	0.1272	Expressed in 3	0.0773	1
Female non membrane	0.1272	Expressed in 3	0.2074	0.0089
Female membrane	0.2231	Asexual blood :	0.193	0.7952
Female membrane	0.2231	Asexual blood :	0.326	0.0166
Female membrane	0.2231	Male non mem	0.1796	0.9113
Female membrane	0.2231	Male membran	0.2897	0.1533
Female membrane	0.2231	Female non me	0.1272	0.9992
Female membrane	0.2231	Female membr	0.2231	0.504
Female membrane	0.2231	Expressed in 3	0.0773	1
Female membrane	0.2231	Expressed in 3	0.2074	0.6317
Expressed in 3 stages non membra	0.0773	Asexual blood :	0.193	0
Expressed in 3 stages non membra	0.0773	Asexual blood :	0.326	0
Expressed in 3 stages non membra	0.0773	Male non mem	0.1796	0

Expressed in 3 stages non membra	0.0773	Male membran	0.2897	0
Expressed in 3 stages non membra	0.0773	Female non me	0.1272	0
Expressed in 3 stages non membra	0.0773	Female membr	0.2231	0
Expressed in 3 stages non membra	0.0773	Expressed in 3	0.0773	0.4968
Expressed in 3 stages non membra	0.0773	Expressed in 3	0.2074	0
Expressed in 3 stages membrane	0.2074	Asexual blood	0.193	0.626
Expressed in 3 stages membrane	0.2074	Asexual blood	0.326	0.0097
Expressed in 3 stages membrane	0.2074	Male non mem	0.1796	0.7607
Expressed in 3 stages membrane	0.2074	Male membran	0.2897	0.1043
Expressed in 3 stages membrane	0.2074	Female non me	0.1272	0.9895
Expressed in 3 stages membrane	0.2074	Female membr	0.2231	0.3722
Expressed in 3 stages membrane	0.2074	Expressed in 3	0.0773	1
Expressed in 3 stages membrane	0.2074	Expressed in 3	0.2074	0.5062

Pb/Py Table E: dn/ds for all sets BIRs are excluded from membrane males

Set	Number of genes	Mean dn/ds	CI 95%	CI 95%
Membrane male	29	0.2188	0.1718	0.2701
Male non membrane	196	0.1796	0.1662	0.1936
Membrane Female	26	0.2231	0.1729	0.2801
Female non membrane	71	0.1272	0.1099	0.1451
Membrane expressed in 3 stages	35	0.2074	0.1521	0.2700
Expressed in 3 stages non membrane	262	0.0773	0.0689	0.0869
Membrane asexual	55	0.3260	0.2710	0.3858
Asexual non membrane	110	0.1930	0.1673	0.2221

Pb/Py Table F: dn/ds comparisons for all sets

Set 1	Mean dn/ds	Set 2	Mean dn/ds	Pvalue Set 1 > Set 2
Asexual blood stages non membrane	0.193	Asexual blc	0.193	0.5001
Asexual blood stages non membrane	0.193	Asexual blc	0.326	0.0001
Asexual blood stages non membrane	0.193	Male non m	0.1796	0.762
Asexual blood stages non membrane	0.193	Male memb	0.2188	0.2275
Asexual blood stages non membrane	0.193	Female nor	0.1272	0.9995
Asexual blood stages non membrane	0.193	Female me	0.2231	0.2101
Asexual blood stages non membrane	0.193	Expressed	0.0773	1
Asexual blood stages non membrane	0.193	Expressed	0.2074	0.3725
Asexual blood stages membrane	0.326	Asexual blc	0.193	0.9998
Asexual blood stages membrane	0.326	Asexual blc	0.326	0.4969
Asexual blood stages membrane	0.326	Male non m	0.1796	1
Asexual blood stages membrane	0.326	Male memb	0.2188	0.9893
Asexual blood stages membrane	0.326	Female nor	0.1272	1

Asexual blood stages membrane	0.326	Female me	0.2231	0.9845
Asexual blood stages membrane	0.326	Expressed	0.0773	1
Asexual blood stages membrane	0.326	Expressed	0.2074	0.988
Male non membrane	0.1796	Asexual blc	0.193	0.2404
Male non membrane	0.1796	Asexual blc	0.326	0
Male non membrane	0.1796	Male non m	0.1796	0.5088
Male non membrane	0.1796	Male memb	0.2188	0.1028
Male non membrane	0.1796	Female nor	0.1272	1
Male non membrane	0.1796	Female me	0.2231	0.0966
Male non membrane	0.1796	Expressed	0.0773	1
Male non membrane	0.1796	Expressed	0.2074	0.2344
Male membrane	0.2188	Asexual blc	0.193	0.7634
Male membrane	0.2188	Asexual blc	0.326	0.0102
Male membrane	0.2188	Male non m	0.1796	0.8934
Male membrane	0.2188	Male memb	0.2188	0.5052
Male membrane	0.2188	Female nor	0.1272	0.9992
Male membrane	0.2188	Female me	0.2231	0.4691
Male membrane	0.2188	Expressed	0.0773	1
Male membrane	0.2188	Expressed	0.2074	0.6019
Female non membrane	0.1272	Asexual blc	0.193	0.0004
Female non membrane	0.1272	Asexual blc	0.326	0
Female non membrane	0.1272	Male non m	0.1796	0
Female non membrane	0.1272	Male memb	0.2188	0.0006
Female non membrane	0.1272	Female nor	0.1272	0.4997
Female non membrane	0.1272	Female me	0.2231	0.0002
Female non membrane	0.1272	Expressed	0.0773	1
Female non membrane	0.1272	Expressed	0.2074	0.0095
Female membrane	0.2231	Asexual blc	0.193	0.7954
Female membrane	0.2231	Asexual blc	0.326	0.0169
Female membrane	0.2231	Male non m	0.1796	0.9081
Female membrane	0.2231	Male memb	0.2188	0.5315
Female membrane	0.2231	Female nor	0.1272	0.9994
Female membrane	0.2231	Female me	0.2231	0.5033
Female membrane	0.2231	Expressed	0.0773	1
Female membrane	0.2231	Expressed	0.2074	0.6232
Expressed in 3 stages non membrane	0.0773	Asexual blc	0.193	0
Expressed in 3 stages non membrane	0.0773	Asexual blc	0.326	0

Expressed in 3 stages non membrane	0.0773	Male non m	0.1796	0
Expressed in 3 stages non membrane	0.0773	Male memb	0.2188	0
Expressed in 3 stages non membrane	0.0773	Female nor	0.1272	0
Expressed in 3 stages non membrane	0.0773	Female me	0.2231	0
Expressed in 3 stages non membrane	0.0773	Expressed	0.0773	0.5029
Expressed in 3 stages non membrane	0.0773	Expressed	0.2074	0
Expressed in 3 stages membrane	0.2074	Asexual blc	0.193	0.6254
Expressed in 3 stages membrane	0.2074	Asexual blc	0.326	0.0118
Expressed in 3 stages membrane	0.2074	Male non m	0.1796	0.7606
Expressed in 3 stages membrane	0.2074	Male memb	0.2188	0.3992
Expressed in 3 stages membrane	0.2074	Female nor	0.1272	0.9904
Expressed in 3 stages membrane	0.2074	Female me	0.2231	0.3742
Expressed in 3 stages membrane	0.2074	Expressed	0.0773	1
Expressed in 3 stages membrane	0.2074	Expressed	0.2074	0.5035

Pb/Py Table G: ds for all sets genes

Set	Number of genes	Mean ds	CI 95%	CI 95%
Membrane male	31	0.1545	0.1323	0.1776
Male non membrane	196	0.1617	0.1519	0.1717
Membrane female	26	0.1487	0.1255	0.1739
Female non membrane	71	0.1652	0.1526	0.1780
Membrane expressed in 3 stages	35	0.1655	0.1448	0.1873
Expressed in 3 stages non membrane	262	0.1679	0.1598	0.1763
Membrane asexual	55	0.1514	0.1374	0.1663
Asexual non membrane	110	0.1583	0.1466	0.1704

Pb/Py Table H: ds comparisons for all sets

Set 1	Mean ds	Set 2	Mean ds	Pvalue Set 1 > Set 2
Asexual blood stages non membrane	0.1583	Asexual blood stages non membrane	0.1583	0.5035
Asexual blood stages non membrane	0.1583	Asexual blood stages membrane	0.1514	0.7411
Asexual blood stages non membrane	0.1583	Male non membrane	0.1617	0.3555
Asexual blood stages non membrane	0.1583	Male membrane	0.1545	0.6009
Asexual blood stages non membrane	0.1583	Female non membrane	0.1652	0.2525
Asexual blood stages non membrane	0.1583	Female membrane	0.1487	0.7286
Asexual blood stages non membrane	0.1583	Expressed in 3 stages non membrane	0.1679	0.1364
Asexual blood stages non membrane	0.1583	Expressed in 3 stages membrane	0.1655	0.3187
Asexual blood stages membrane	0.1514	Asexual blood stages non membrane	0.1583	0.2721
Asexual blood stages membrane	0.1514	Asexual blood stages membrane	0.1514	0.4887
Asexual blood stages membrane	0.1514	Male non membrane	0.1617	0.1711
Asexual blood stages membrane	0.1514	Male membrane	0.1545	0.4286
Asexual blood stages membrane	0.1514	Female non membrane	0.1652	0.1162
Asexual blood stages membrane	0.1514	Female membrane	0.1487	0.5662

Asexual blood stages membrane	0.1514	Expressed in 3 stages non membrane	0.1679	0.0554
Asexual blood stages membrane	0.1514	Expressed in 3 stages membrane	0.1655	0.1826
Male non membrane	0.1617	Asexual blood stages non membrane	0.1583	0.6402
Male non membrane	0.1617	Asexual blood stages membrane	0.1514	0.8297
Male non membrane	0.1617	Male non membrane	0.1617	0.5031
Male non membrane	0.1617	Male membrane	0.1545	0.6944
Male non membrane	0.1617	Female non membrane	0.1652	0.3644
Male non membrane	0.1617	Female membrane	0.1487	0.7941
Male non membrane	0.1617	Expressed in 3 stages non membrane	0.1679	0.2102
Male non membrane	0.1617	Expressed in 3 stages membrane	0.1655	0.3973
Male membrane	0.1545	Asexual blood stages non membrane	0.1583	0.3983
Male membrane	0.1545	Asexual blood stages membrane	0.1514	0.5697
Male membrane	0.1545	Male non membrane	0.1617	0.3074
Male membrane	0.1545	Male membrane	0.1545	0.5045
Male membrane	0.1545	Female non membrane	0.1652	0.2413
Male membrane	0.1545	Female membrane	0.1487	0.6084
Male membrane	0.1545	Expressed in 3 stages non membrane	0.1679	0.18
Male membrane	0.1545	Expressed in 3 stages membrane	0.1655	0.2798
Female non membrane	0.1652	Asexual blood stages non membrane	0.1583	0.7479
Female non membrane	0.1652	Asexual blood stages membrane	0.1514	0.8771
Female non membrane	0.1652	Male non membrane	0.1617	0.6395
Female non membrane	0.1652	Male membrane	0.1545	0.7666
Female non membrane	0.1652	Female non membrane	0.1652	0.4956
Female non membrane	0.1652	Female membrane	0.1487	0.8326
Female non membrane	0.1652	Expressed in 3 stages non membrane	0.1679	0.3872
Female non membrane	0.1652	Expressed in 3 stages membrane	0.1655	0.5015
Female membrane	0.1487	Asexual blood stages non membrane	0.1583	0.2733
Female membrane	0.1487	Asexual blood stages membrane	0.1514	0.4329
Female membrane	0.1487	Male non membrane	0.1617	0.2072
Female membrane	0.1487	Male membrane	0.1545	0.3872
Female membrane	0.1487	Female non membrane	0.1652	0.1664
Female membrane	0.1487	Female membrane	0.1487	0.4991
Female membrane	0.1487	Expressed in 3 stages non membrane	0.1679	0.1195
Female membrane	0.1487	Expressed in 3 stages membrane	0.1655	0.1931
Expressed in 3 stages non membrane	0.1679	Asexual blood stages non membrane	0.1583	0.8601
Expressed in 3 stages non membrane	0.1679	Asexual blood stages membrane	0.1514	0.9458

Expressed in 3 stages non membrane	0.1679	Male non membrane	0.1617	0.7864
Expressed in 3 stages non membrane	0.1679	Male membrane	0.1545	0.8186
Expressed in 3 stages non membrane	0.1679	Female non membrane	0.1652	0.6196
Expressed in 3 stages non membrane	0.1679	Female membrane	0.1487	0.8876
Expressed in 3 stages non membrane	0.1679	Expressed in 3 stages non membrane	0.1679	0.4995
Expressed in 3 stages non membrane	0.1679	Expressed in 3 stages membrane	0.1655	0.5765
Expressed in 3 stages membrane	0.1655	Asexual blood stages non membrane	0.1583	0.6814
Expressed in 3 stages membrane	0.1655	Asexual blood stages membrane	0.1514	0.8133
Expressed in 3 stages membrane	0.1655	Male non membrane	0.1617	0.5888
Expressed in 3 stages membrane	0.1655	Male membrane	0.1545	0.7165
Expressed in 3 stages membrane	0.1655	Female non membrane	0.1652	0.4924
Expressed in 3 stages membrane	0.1655	Female membrane	0.1487	0.8018
Expressed in 3 stages membrane	0.1655	Expressed in 3 stages non membrane	0.1679	0.4132
Expressed in 3 stages membrane	0.1655	Expressed in 3 stages membrane	0.1655	0.5084
