# Supplementary material for: Why are male malaria parasites in such a rush? Sex-specific evolution and host–parasite interactions
Source: Evol Med Public Health. 2012 Nov 26;2013(1):3–13. doi: 10.1093/emph/eos003 (PMC4183958; doi:10.1093/emph/eos003)
Supplement: Supplementary Data [file supp_eos003_suppl_data.zip › REECE_SD_files.pdf]

Supplementary data

Figure S1: Distribution of genes with sex-biased expression. The 14 chromosomes of *P. berghei* are shown as numerals with male-specific genes are indicated in blue, female specific genes in pink, and genes expressed in both males and females are in black. The random or non-random distribution of genes was analysed by determining the distance between gene pairs (of either 'male' or 'female' genes) within the genome as defined as the number of intervening genes. The tendency for genes of the same category (i.e. male or female) to cluster was measured by calculating the sum of the minimal distances measured between gene-pairs from their respective category. The sum of these distances was then compared to that of these same genes in a 1000 randomly permutated genomes and the probability of non-random clustering was calculated; the significance values for male gene clustering was calculated as P=0.1 and for female genes as P=0.4, indicating a random distribution of the gender-specific genes across the chromosomes.

Figure S2: d_N and d_S for each closely related pair of *Plasmodium* species: *P. berghei* and *P. yoelii* (A, B); *P. falciparum* and *P. reichenowi* (C, D); *P. vivax* and *P. knowlesi* (E, F).

Table S1 PB: *P. berghei* genes: gene model, stage of expression identified in a proteomics analysis, d_N/d_S and d_S (comparison of *P. berghei* versus *P. yoelii*) and predicted membrane localization of the proteins.

Table S1 PF: *P. falciparum* orthologs of the *P. berghei* genes shown in Table S1 PB, and d_N, d_S and d_N/d_S (comparison of *P. falciparum* versus *P. reichenowi)*: Genes are subdivided according to the stage of expression (see different tables/sheets: 'Male', 'Female', All stages' and Asexual blood') and to the predicted membrane localisation of the encoded proteins.

Table S1 PV: *P. vivax* orthologs of the *P. berghei* genes shown in Table S1 PB, and d_N, d_S and d_N/d_S (comparison of *P. vivax* versus *P. knowlesi)*: Genes are subdivided according to the stage of

expression (see different tables/sheets: ‘Male’, ‘Female’, All stages’ and Asexual blood’) and according to the predicted membrane localisation of the encoded proteins.

Table S2 PB: Mean d_N/d_S and d_S values and 95% confidence intervals (CI), for the comparison of genes of *P. berghei* versus *P. yoelii,* according to the stage of expression and predicted (non-)membrane localisation. A, B: d_N/d_S for stage-specific gene sets; C, D: d_N/d_S for stage-specific gene sets including predicted (non-)membrane proteins; E, F: d_S values for all sets of genes.

Table S2 PF: Mean d_N/d_S and d_S values and 95% confidence intervals (CI), for the comparison of genes of *P. falciparum* versus *P. reichenowi*, according to the stage of expression and predicted (non-)membrane localisation. A, B: d_N/d_S for stage-specific gene sets; C, D: d_N/d_S for stage-specific gene sets including predicted (non-)membrane proteins; E, F: d_S values for all sets of genes.

Table S2 PV: Mean d_N/d_S and d_S values and 95% confidence intervals (CI), for the comparison of genes of *P. vivax* versus *P. knowlesi*, according to the stage of expression and predicted (non-)membrane localisation. A, B: d_N/d_S for stage-specific gene sets; C, D: d_N/d_S for stage-specific gene sets including predicted (non-)membrane proteins; E, F: d_S values for all sets of genes.

Table S3: List of genes containing non-sense mutations in *P. berghei* and *P. yoelii* for which more than 20 codons are available.

Table S4: A: *P. falciparum* orthologs of the *P. berghei* genes shown in Table S1 PB that contain epitopes (available from the Immune Epitope Database and Analysis. Genes are subdivided according to the stage of expression (‘Male’, ‘Female’, All stages’ and Asexual blood’) and to the predicted membrane localisation of the encoded proteins. B: Percentage of proteins containing epitopes according to the stage of expression and predicted (non-)membrane localisation. C: *pn /ps*, *pn and ps* values of *P. falciparum* orthologs (comparison of 3d7 versus the Ghana isolate) of the *P. berghei*

genes shown in Table S1 PB). Genes are subdivided according to the stage of expression and to the predicted membrane localisation of the encoded proteins. D, E: Mean *pn/ps* values and 95% confidence intervals (CI) of *P. falciparum* proteins with a predicted membrane localisation of the encoded proteins. Genes are subdivided according to the stage of expression (Table S1 PB) and the presence of epitopes (see Table S4A). F, G: Mean *pn/ps* values and 95% confidence intervals (CI) of *P. falciparum* proteins with a predicted membrane localisation of the encoded proteins. Genes are subdivided according to the stage of expression (Table S1 PB) and the presence of epitopes (see Table S4A). H: *pn/ps* values of *P. falciparum* orthologs of *P. berghei* male genes (comparison of 3d7 versus the Ghana isolate)

Table S5: Polymorphism data used in McDonald-Kreitman test to compare the relative probability of fixation of non-synonymous mutations (d_N/d_S) between species (*P. falciparum* and *P. reichenowi*) to the observed relative probability of non-synonymous polymorphisms (p_N/p_S) in natural populations within a species (*P. falciparum* strains 3d7, Dd2, Hb3, 7G8, D10, D6, SL, K1, RO-33, IT, FCC-2/Hainan, Senegal, IGH-CR14).

Table S6: Repeat of the analyses comparing *P. berghei* versus *P. yoelii,* according to the stage of expression and predicted (non-)membrane localisation, using new gene models from PlasmoDB 7.1. As in Table S2 PB, mean d_N/d_S and d_S values and 95% confidence intervals (CI) are presented. A, B: d_N/d_S for stage-specific gene sets; C, D: d_N/d_S for stage-specific gene sets including predicted (non-)membrane proteins; E, F: d_N/d_S for stage-specific gene sets including predicted (non-)membrane proteins but excluding BIR's (as 1 BIR gene is now predicted to have a membrane, instead of non-membrane, location); G,H d_S values for all sets of genes.

SOM
Figure 1

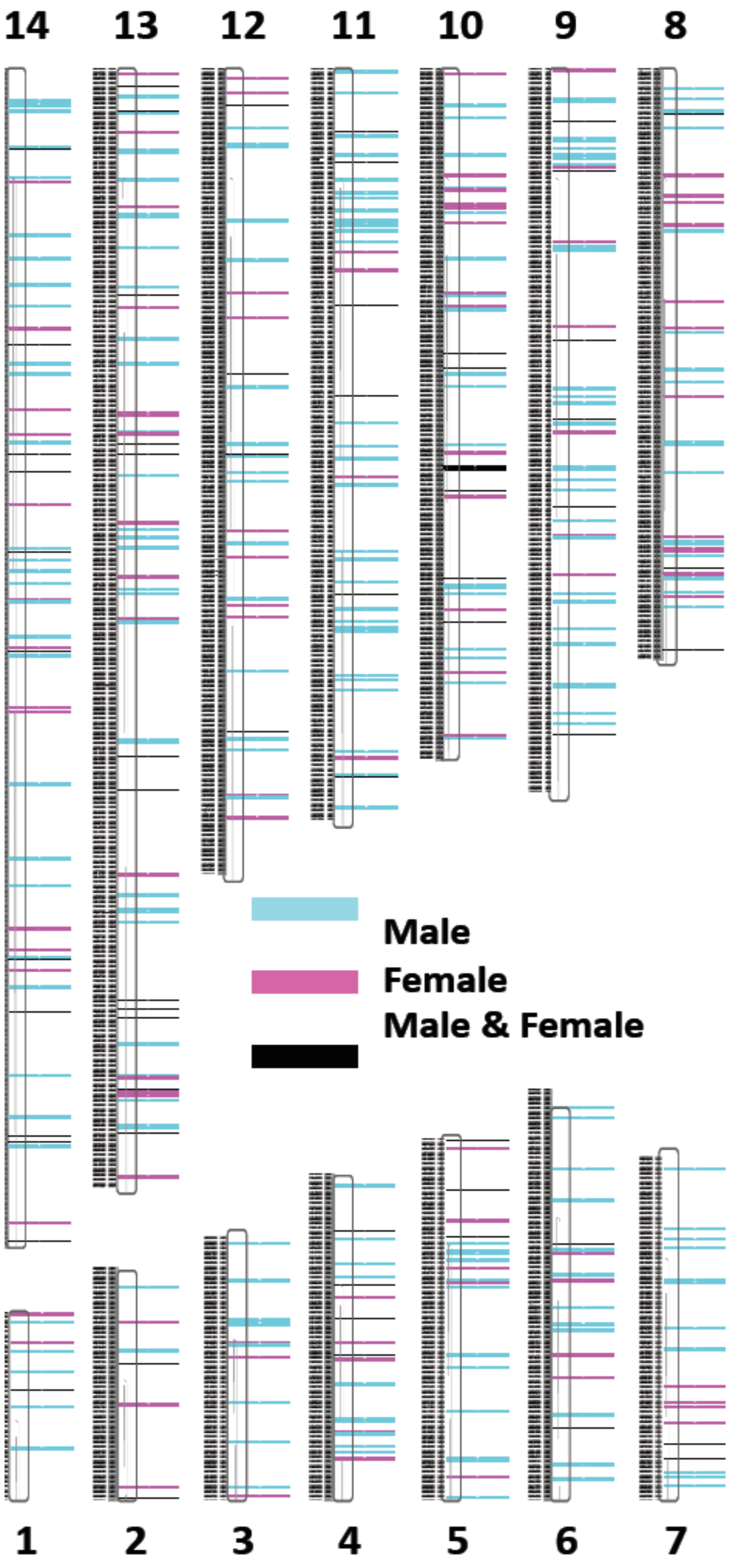

SOM
Figure 2

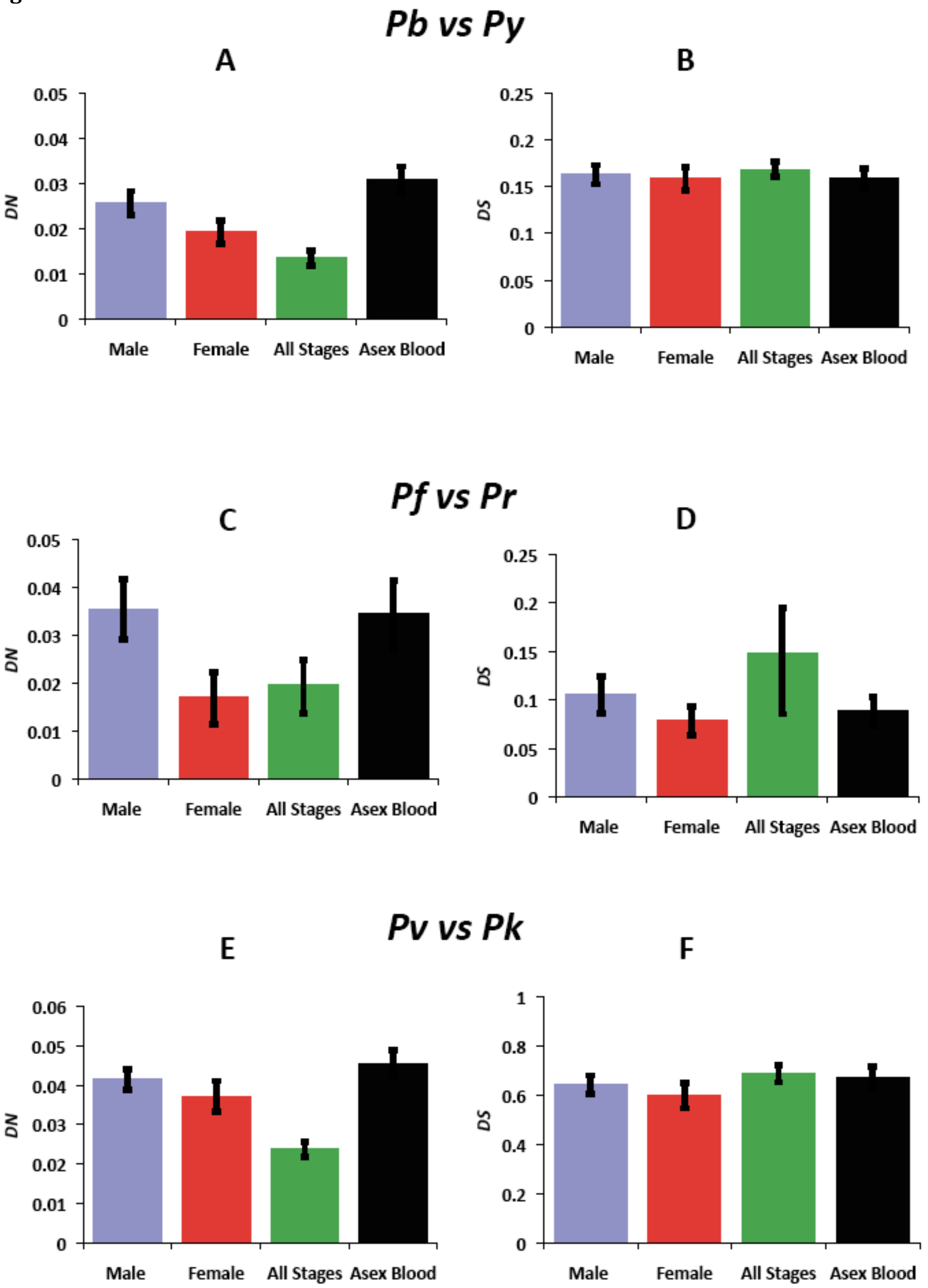

Pb vs Py
A
0.05
0.04
0.03
0.02
0.01
0
DN
Male
Female
All Stages
Asex Blood
B
0.25
0.2
0.15
0.1
0.05
0
DS
Male
Female
All Stages
Asex Blood
Pf vs Pr
C
0.05
0.04
0.03
0.02
0.01
0
DN
Male
Female
All Stages
Asex Blood
D
0.25
0.2
0.15
0.1
0.05
0
DS
Male
Female
All Stages
Asex Blood
Pv vs Pk
E
0.06
0.05
0.04
0.03
0.02
0.01
0
DN
Male
Female
All Stages
Asex Blood
F
1
0.8
0.6
0.4
0.2
0
DS
Male
Female
All Stages
Asex Blood
