# Supplementary material for: Why are male malaria parasites in such a rush? Sex-specific evolution and host–parasite interactions
Source: Evol Med Public Health. 2012 Nov 26;2013(1):3–13. doi: 10.1093/emph/eos003 (PMC4183958; doi:10.1093/emph/eos003)
Supplement: Supplementary Data [file supp_eos003_suppl_data.zip › REECE_Table_S1_PB.pdf]

Pb/Py Table A: ds, dn/ds, expression stages & location of P. berghei genes

Plasmodb ID	Annotation	Set	ds	dn/ds	Precicted membrane localization
PBANKA_120700	nucleolar protein NOP5, putative	Expressed in asexual blood stages	0.0974	0.0787	-
PBANKA_031300	conserved Plasmodium protein, unknown function	Expressed in asexual blood stages	0.3367	0.2170	-
PBANKA_145950	myosin A tail domain interacting protein MTIP, putative	Expressed in asexual blood stages	0.0871	0.3352	-
PBANKA_124060	membrane skeletal protein, putative	Expressed in asexual blood stages	0.1329	0.2780	-
PBANKA_144800	conserved Plasmodium protein, unknown function	Expressed in asexual blood stages	0.2100	0.1728	-
PBANKA_144560	protein kinase, putative	Expressed in asexual blood stages	0.0885	0.2902	-
PBANKA_092250	conserved Plasmodium protein, unknown function	Expressed in asexual blood stages	0.1014	0.2636	-
PBANKA_101930	conserved Plasmodium protein, unknown function	Expressed in asexual blood stages	0.2320	0.0426	-
PBANKA_040680	PRP19-like protein, putative	Expressed in asexual blood stages	0.2228	0.0893	-
PBANKA_011220	myosin-like protein, putative	Expressed in asexual blood stages	0.0999	0.4369	-
PBANKA_101490	coatamer protein, beta subunit, putative	Expressed in asexual blood stages	0.2961	0.1090	-
PBANKA_092980	insulinase, putative	Expressed in asexual blood stages	0.1892	0.1692	-
PBANKA_142590	conserved Plasmodium protein, unknown function	Expressed in asexual blood stages	0.0915	0.6297	-
PBANKA_070980	conserved Plasmodium protein, unknown function	Expressed in asexual blood stages	0.1896	0.1085	+
PBANKA_060530	eukaryotic translation initiation factor 5 putative	Expressed in asexual blood stages	0.1829	0.0373	-
PBANKA_030230	aminotransferase, putative	Expressed in asexual blood stages	0.1482	0.3084	-
PBANKA_093200	moving junction protein, putative	Expressed in asexual blood stages	0.1101	0.5599	-
PBANKA_062140	conserved Plasmodium protein, unknown function	Expressed in asexual blood stages	0.2983	0.1947	-
PBANKA_113710	arginine methyltransferase 5 putative	Expressed in asexual blood stages	0.1212	0.1617	-
PBANKA_093160	conserved Plasmodium protein, unknown function	Expressed in asexual blood stages	0.1395	0.0557	-
PBANKA_130890	histidine -- tRNA ligase, putative	Expressed in asexual blood stages	0.1376	0.1259	-
PBANKA_060670	cysteine repeat modular protein 3	Expressed in asexual blood stages	0.0824	0.6990	+
PBANKA_090080	reticulocyte binding protein, putative	Expressed in asexual blood stages	0.1161	0.7432	-
PBANKA_050140	conserved Plasmodium protein, unknown function	Expressed in asexual blood stages	0.0641	0.6681	-
PBANKA_145990	signal recognition particle, beta subunit, putative	Expressed in asexual blood stages	0.2442	0.0284	-
PBANKA_041750	60S ribosomal protein L32, putative	Expressed in asexual blood stages	0.0713	0.0491	-
PBANKA_113720	Sec24 subunit, putative	Expressed in asexual blood stages	0.0968	0.1375	-
PBANKA_101040	conserved Plasmodium protein, unknown function	Expressed in asexual blood stages	0.1751	0.2293	+
PBANKA_031100	COPII-coated vesicle component	Expressed in asexual blood stages	0.2008	0.1810	-
PBANKA_111180	glutathione synthetase, putative	Expressed in asexual blood stages	0.2058	0.0380	-

PBANKA_133740	adenosine-diphosphatase, putative	Expressed in asexual blood stages	0.1037	0.2720	-
PBANKA_011120	nucleolar GTP-binding protein 1 putative	Expressed in asexual blood stages	0.2234	0.1136	-
PBANKA_110720	cell differentiation protein rcd1, putative	Expressed in asexual blood stages	0.2076	0.1587	-
PBANKA_123630	DEAD-box subfamily ATP-dependent helicase, putative	Expressed in asexual blood stages	0.2173	0.1638	-
PBANKA_134910	merozoite surface protein 7	Expressed in asexual blood stages	0.1803	0.4606	+
PBANKA_132830	ATP-dependent protease la, putative	Expressed in asexual blood stages	0.1190	0.0513	-
PBANKA_061970	conserved Plasmodium protein, unknown function	Expressed in asexual blood stages	0.2466	0.2362	-
PBANKA_113810	14-3-2003 protein, putative	Expressed in asexual blood stages	0.0850	0.2662	+
PBANKA_102400	conserved Plasmodium protein, unknown function	Expressed in asexual blood stages	0.1389	0.1663	+
PBANKA_061350	conserved Plasmodium protein, unknown function	Expressed in asexual blood stages	0.2199	0.1360	-
PBANKA_142500	RNA-binding protein, putative	Expressed in asexual blood stages	0.0940	0.3561	-
PBANKA_114350	ribosome biogenesis protein MRT4, putative	Expressed in asexual blood stages	0.0669	0.4379	-
PBANKA_132730	conserved Plasmodium protein, unknown function	Expressed in asexual blood stages	0.2104	0.3938	-
PBANKA_120790	protein phosphatase, putative	Expressed in asexual blood stages	0.0415	1..5952	-
PBANKA_140230	conserved Plasmodium protein, unknown function	Expressed in asexual blood stages	0.1149	0.3474	-
PBANKA_145960	conserved Plasmodium protein, unknown function	Expressed in asexual blood stages	0.1355	0.1884	-
PBANKA_103540	conserved Plasmodium protein, unknown function	Expressed in asexual blood stages	0.1494	0.0109	+
PBANKA_071700	ATPase, putative	Expressed in asexual blood stages	0.3080	0.0486	-
PBANKA_114410	RNAse L inhibitor protein, putative	Expressed in asexual blood stages	0.0431	0.1607	-
PBANKA_133240	phosphoesterase, putative	Expressed in asexual blood stages	0.1059	0.2403	+
PBANKA_050660	pre-mRNA splicing factor, putative	Expressed in asexual blood stages	0.1106	0.2698	-
PBANKA_141730	DnaJ/SEC63 protein, putative	Expressed in asexual blood stages	0.1049	0.1240	+
PBANKA_041740	GTPase activating protein, putative	Expressed in asexual blood stages	0.1367	0.1442	-
PBANKA_136330	conserved Plasmodium protein, unknown function	Expressed in asexual blood stages	0.2161	0.4181	+
PBANKA_146490	rhoptry neck protein 3 putative	Expressed in asexual blood stages	0.1154	0.3779	+
PBANKA_031170	rhoptry neck protein 6 putative	Expressed in asexual blood stages	0.1750	0.4610	-
PBANKA_083370	conserved Plasmodium protein, unknown function	Expressed in asexual blood stages	0.1858	0.0781	-
PBANKA_020460	photosensitized INA-labeled protein 1 putative	Expressed in asexual blood stages	0.0819	0.3586	-
PBANKA_061080	eukaryotic initiation factor 2a, putative	Expressed in asexual blood stages	0.1854	0.1189	-
PBANKA_031180	long-chain acyl-CoA synthase, putative	Expressed in asexual blood stages	0.2455	0.3672	-
PBANKA_140520	conserved Plasmodium protein, unknown function	Expressed in asexual blood stages	0.0544	0.5414	-
PBANKA_111680	conserved Plasmodium protein, unknown function	Expressed in asexual blood stages	0.0395	0.5060	-
PBANKA_093710	vacuolar sorting protein 35 putative	Expressed in asexual blood stages	0.3158	0.0967	+
PBANKA_144650	asparagine-rich protein, putative	Expressed in asexual blood stages	0.2374	0.1701	-

PBANKA_112650	acetyl-CoA synthetase, putative	Expressed in asexual blood stages	0.0867	0.2409	-
PBANKA_112080	conserved Plasmodium protein, unknown function	Expressed in asexual blood stages	0.2622	0.1915	-
PBANKA_081770	RNA-binding protein, putative	Expressed in asexual blood stages	0.2977	0.0836	-
PBANKA_092780	tRNA nucleotidyltransferase, putative	Expressed in asexual blood stages	0.1550	0.1216	-
PBANKA_100850	translocon component PTEX150	Expressed in asexual blood stages	0.1550	0.4845	-
PBANKA_051140	merozoite capping protein 1 putative	Expressed in asexual blood stages	0.3819	0.0883	-
PBANKA_135670	conserved Plasmodium protein, unknown function	Expressed in asexual blood stages	0.1066	0.0794	-
PBANKA_143720	conserved Plasmodium protein, unknown function	Expressed in asexual blood stages	0.0741	0.2075	-
PBANKA_111880	conserved Plasmodium protein, unknown function	Expressed in asexual blood stages	0.1569	0.2503	-
PBANKA_083600	vesicle transport protein, putative	Expressed in asexual blood stages	0.2364	0.1698	-
PBANKA_092140	conserved Plasmodium protein, unknown function	Expressed in asexual blood stages	0.2291	0.1646	-
PBANKA_093690	actin-like protein, putative	Expressed in asexual blood stages	0.2198	0.1102	-
PBANKA_121130	conserved Plasmodium protein, unknown function	Expressed in asexual blood stages	0.0764	0.0302	-
PBANKA_141590	conserved Plasmodium protein, unknown function	Expressed in asexual blood stages	0.1077	0.4552	-
PBANKA_040460	activator of Hsp90 ATPase, putative	Expressed in asexual blood stages	0.1329	0.0975	-
PBANKA_144540	transport protein Sec13, putative	Expressed in asexual blood stages	0.0880	0.2953	-
PBANKA_040630	DNA polymerase epsilon subunit b, putative	Expressed in asexual blood stages	0.1203	0.1433	-
PBANKA_143550	conserved Plasmodium protein, unknown function	Expressed in asexual blood stages	0.0834	0.4008	-
PBANKA_071310	conserved Plasmodium protein, unknown function	Expressed in asexual blood stages	0.2510	0.1460	-
PBANKA_103550	vacuolar sorting protein 29 putative	Expressed in asexual blood stages	0.0955	0.0000	-
PBANKA_120080	RNA-binding protein, putative	Expressed in asexual blood stages	0.1511	0.0958	-
PBANKA_112430	conserved Plasmodium protein, unknown function	Expressed in asexual blood stages	0.1818	0.2210	-
PBANKA_120200	membrane skeletal protein, putative	Expressed in asexual blood stages	0.1359	0.1298	-
PBANKA_100820	cGMP-dependent protein kinase	Expressed in asexual blood stages	0.1061	0.0198	-
PBANKA_103510	fibrillarin, putative	Expressed in asexual blood stages	0.2265	0.0159	-
PBANKA_120730	diphthine synthase, putative	Expressed in asexual blood stages	0.0873	0.1187	-
PBANKA_104010	choline kinase, putative	Expressed in asexual blood stages	0.0549	0.3192	-
PBANKA_100360	apical sushi protein, putative	Expressed in asexual blood stages	0.1034	0.4184	+
PBANKA_091980	farnesyl pyrophosphate synthase, putative	Expressed in asexual blood stages	0.1498	0.1017	-
PBANKA_092850	peptidase, putative	Expressed in asexual blood stages	0.1202	0.0753	+
PBANKA_135570	myosin A	Expressed in asexual blood stages	0.0955	0.0893	-
PBANKA_142110	transcription elongation factor s-II, putative	Expressed in asexual blood stages	0.1346	0.1585	-
PBANKA_094330	actin-related protein, putative	Expressed in asexual blood stages	0.1108	0.1675	-
PBANKA_071120	conserved Plasmodium protein, unknown function	Expressed in asexual blood stages	0.0790	0.3999	-

PBANKA_135770	aspartate carbamoyltransferase, putative	Expressed in asexual blood stages	0.0653	0.2216	-
PBANKA_102460	liver specific protein 1	Expressed in asexual blood stages	0.0954	0.5827	+
PBANKA_081540	conserved Plasmodium protein, unknown function	Expressed in asexual blood stages	0.1942	0.0000	-
PBANKA_030510	serine repeat antigen 1	Expressed in asexual blood stages	0.0901	0.8590	-
PBANKA_051780	Sec1 family protein, putative	Expressed in asexual blood stages	0.0884	0.1249	-
PBANKA_131370	serine/threonine protein kinase, putative	Expressed in asexual blood stages	0.1806	0.0078	-
PBANKA_102010	PP1-like protein serine/threonine phosphatase, putative	Expressed in asexual blood stages	0.2760	0.0374	-
PBANKA_093750	conserved Plasmodium protein, unknown function	Expressed in asexual blood stages	0.1649	0.1425	+
PBANKA_114130	aminomethyltransferase, putative	Expressed in asexual blood stages	0.1274	0.1686	-
PBANKA_051520	MORN repeat containing protein	Expressed in asexual blood stages	0.1197	0.1138	-
PBANKA_103800	cytochrome c, putative	Expressed in asexual blood stages	0.0780	0.1067	-
PBANKA_071000	conserved Plasmodium protein, unknown function	Expressed in asexual blood stages	0.4893	0.1182	-
PBANKA_136050	ethanolamine-phosphate cytidylyltransferase, putative	Expressed in asexual blood stages	0.0939	0.1136	-
PBANKA_082390	DNA-directed RNA polymerase II, putative	Expressed in asexual blood stages	0.1522	0.0966	-
PBANKA_146000	serine/threonine protein kinase, putative	Expressed in asexual blood stages	0.1459	0.2939	-
PBANKA_145150	conserved Plasmodium protein, unknown function	Expressed in asexual blood stages	0.1826	0.1131	-
PBANKA_131930	ferlin, putative	Expressed in asexual blood stages	0.0827	0.2608	+
PBANKA_080710	band 7-related protein, putative	Expressed in asexual blood stages	0.0780	0.1388	-
PBANKA_122210	regulator of chromosome condensation protein, putative	Expressed in asexual blood stages	0.2393	0.2026	-
PBANKA_124360	conserved Plasmodium protein, unknown function	Expressed in asexual blood stages	0.1905	0.1256	+
PBANKA_052000	pyruvate kinase 2 putative	Expressed in asexual blood stages	0.0985	0.1721	+
PBANKA_010790	conserved Plasmodium protein, unknown function	Expressed in asexual blood stages	0.2324	0.4181	-
PBANKA_050740	orotidine-monophosphate-decarboxylase, putative	Expressed in asexual blood stages	0.0925	0.2489	-
PBANKA_041410	inorganic pyrophosphatase, putative	Expressed in asexual blood stages	0.3178	0.1003	-
PBANKA_134440	AMP deaminase, putative	Expressed in asexual blood stages	0.2217	0.0713	-
PBANKA_144330	merozoite surface protein 9 putative	Expressed in asexual blood stages	0.2322	0.2351	-
PBANKA_103950	U1 small nuclear ribonucleoprotein C, putative	Expressed in asexual blood stages	0.1344	0.3220	-
PBANKA_133870	plasmepsin V, putative	Expressed in asexual blood stages	0.2018	0.1471	+
PBANKA_131900	conserved Plasmodium protein, unknown function	Expressed in asexual blood stages	0.1195	0.3523	-
PBANKA_103340	oxidoreductase, putative	Expressed in asexual blood stages	0.1004	0.0654	-
PBANKA_120860	conserved Plasmodium protein, unknown function	Expressed in asexual blood stages	0.1378	0.2448	-
PBANKA_071720	conserved Plasmodium protein, unknown function	Expressed in asexual blood stages	0.2425	0.2092	-
PBANKA_135590	phosphoenolpyruvate carboxykinase, putative	Expressed in asexual blood stages	0.1183	0.0838	-
PBANKA_110480	actin-related protein, putative	Expressed in asexual blood stages	0.1660	0.2265	-

PBANKA_052310	eukaryotic initiation factor, putative	Expressed in asexual blood stages	0.2513	0.0443	-
PBANKA_040770	60S acidic ribosomal protein P2, putative	Expressed in asexual blood stages	0.2417	0.0540	-
PBANKA_094130	translocon component PTEX88, putative	Expressed in asexual blood stages	0.2783	0.2717	+
PBANKA_091500	apical membrane antigen 1	Expressed in asexual blood stages	0.1148	0.4745	+
PBANKA_114250	signal recognition particle receptor alpha subunit, putative	Expressed in asexual blood stages	0.1557	0.1484	-
PBANKA_051190	60S ribosomal protein L3, putative	Expressed in asexual blood stages	0.2529	0.0254	-
PBANKA_131570	rhoptry neck protein 2	Expressed in asexual blood stages	0.0942	0.4357	+
PBANKA_103210	rhoptry-associated protein 1 putative	Expressed in asexual blood stages	0.1426	0.8707	-
PBANKA_083100	merozoite surface protein 1	Expressed in asexual blood stages	0.1125	0.5387	+
PBANKA_040360	conserved Plasmodium protein, unknown function	Expressed in asexual blood stages	0.2015	0.1088	+
PBANKA_091340	protein phosphatase 2C, putative	Expressed in asexual blood stages	0.0727	0.8913	-
PBANKA_133270	duffy-binding protein	Expressed in asexual blood stages	0.1273	0.6619	+
PBANKA_133920	conserved Plasmodium protein, unknown function	Expressed in asexual blood stages	0.5180	0.1296	-
PBANKA_071420	ClpB protein, putative	Expressed in asexual blood stages	0.2186	0.0300	-
PBANKA_113570	RNA-binding protein, putative	Expressed in asexual blood stages	0.1108	0.0882	-
PBANKA_131170	multidrug resistance protein 2 putative	Expressed in asexual blood stages	0.1893	0.1258	+
PBANKA_140470	conserved Plasmodium protein, unknown function	Expressed in asexual blood stages	0.0729	0.1483	+
PBANKA_140690	conserved Plasmodium protein, unknown function	Expressed in asexual blood stages	0.1123	0.4663	+
PBANKA_082440	conserved Plasmodium protein, unknown function	Expressed in asexual blood stages	0.1789	0.1087	-
PBANKA_144980	inorganic pyrophosphatase, putative	Expressed in asexual blood stages	0.1695	0.2028	+
PBANKA_145700	ubiquitin-conjugating enzyme, putative	Expressed in asexual blood stages	0.0650	0.1657	-
PBANKA_100780	exosome complex exonuclease rrp4, putative	Expressed in asexual blood stages	0.0877	0.0248	-
PBANKA_083560	cAMP-dependent protein kinase, putative	Expressed in asexual blood stages	0.3977	0.0116	-
PBANKA_092480	conserved Plasmodium protein, unknown function	Expressed in asexual blood stages	0.1238	0.2002	+
PBANKA_121700	peptidase, putative	Expressed in asexual blood stages	0.1094	0.1318	-
PBANKA_146200	conserved Plasmodium protein, unknown function	Expressed in asexual blood stages	0.2293	0.1699	-
PBANKA_100260	pfs45-48 related protein, putative	Expressed in asexual blood stages	0.1108	0.3484	+
PBANKA_133210	conserved Plasmodium protein, unknown function	Expressed in asexual blood stages	0.1087	0.2459	-
PBANKA_123210	splicing factor, putative	Expressed in asexual blood stages	0.0928	0.0564	-
PBANKA_072080	conserved Plasmodium protein, unknwon function	Expressed in male gametocytes	0.2524	0.0856	-
PBANKA_094010	conserved Plasmodium protein, unknown function	Expressed in male gametocytes	0.1626	0.4310	+
PBANKA_103050	conserved Plasmodium protein, unknown function	Expressed in male gametocytes	0.0975	0.3605	-
PBANKA_040620	conserved Plasmodium protein, unknown function	Expressed in male gametocytes	0.1216	0.4123	-
PBANKA_144300	serine/threonine protein kinase, putative	Expressed in male gametocytes	0.1201	0.1153	+

PBANKA_041210	serine/threonine protein phosphatase, putative	Expressed in male gametocytes	0.0863	0.0280	-
PBANKA_142720	protein phosphatase 2C, putative	Expressed in male gametocytes	0.1166	0.1854	-
PBANKA_140260	DNA ligase I, putative	Expressed in male gametocytes	0.0858	0.1236	+
PBANKA_093350	rhomboid 1	Expressed in male gametocytes	0.0985	0.4428	+
PBANKA_081080	conserved Plasmodium protein, unknown function	Expressed in male gametocytes	0.1184	0.2170	-
PBANKA_112350	Smarca-related protein, putative	Expressed in male gametocytes	0.2119	0.0846	-
PBANKA_041580	conserved Plasmodium protein, unknown function	Expressed in male gametocytes	0.1740	0.1975	-
PBANKA_123020	cyclin-dependent protein kinase, putative	Expressed in male gametocytes	0.3602	0.0506	-
PBANKA_061430	cysteine desulfurase, putative	Expressed in male gametocytes	0.1309	0.2181	-
PBANKA_041500	Maf-like protein, putative	Expressed in male gametocytes	0.2658	0.1380	-
PBANKA_091100	calcium-binding protein, putative	Expressed in male gametocytes	0.0966	0.2661	-
PBANKA_050920	formin 2 putative	Expressed in male gametocytes	0.7031	0.0866	-
PBANKA_071100	conserved Plasmodium protein, unknown function	Expressed in male gametocytes	0.1557	0.2544	+
PBANKA_010860	step II splicing factor, putative	Expressed in male gametocytes	0.1419	0.1089	-
PBANKA_111010	topoisomerase I, putative	Expressed in male gametocytes	0.0702	0.1818	-
PBANKA_040150	exportin 1 putative	Expressed in male gametocytes	0.2053	0.0365	-
PBANKA_101830	dynein-associated protein, putative	Expressed in male gametocytes	0.1185	0.0891	-
PBANKA_114480	conserved Plasmodium protein, unknown function	Expressed in male gametocytes	0.1128	0.3974	-
PBANKA_083130	peptidyl-prolyl cis-trans isomerase 4 putative	Expressed in male gametocytes	0.1448	0.1587	-
PBANKA_100280	Sec24-like protein, putative	Expressed in male gametocytes	0.0901	0.2287	-
PBANKA_144890	DNA polymerase epsilon subunit b, putative	Expressed in male gametocytes	0.2741	0.0768	-
PBANKA_133760	translation initiation factor EIF-2B subunit, putative	Expressed in male gametocytes	0.1321	0.1952	-
PBANKA_113100	V-type ATPase, putative	Expressed in male gametocytes	0.3566	0.0532	+
PBANKA_142650	cullin-like protein, putative	Expressed in male gametocytes	0.0982	0.1424	-
PBANKA_081920	organelle ribosomal protein L3 precursor, putative	Expressed in male gametocytes	0.2978	0.0291	-
PBANKA_111160	conserved Plasmodium protein, unknown function	Expressed in male gametocytes	0.3776	0.0712	-
PBANKA_082890	conserved Plasmodium protein, unknown function	Expressed in male gametocytes	0.1139	0.3458	-
PBANKA_130260	small subunit DNA primase, putative	Expressed in male gametocytes	0.0799	0.2055	-
PBANKA_093930	conserved Plasmodium protein, unknown function	Expressed in male gametocytes	0.1125	0.1177	-
PBANKA_114280	conserved Plasmodium protein, unknown function	Expressed in male gametocytes	0.2171	0.1247	-
PBANKA_030610	transmission-blocking target antigen s230, putative	Expressed in male gametocytes	0.1016	0.4416	-
PBANKA_091830	transporter, putative	Expressed in male gametocytes	0.0950	0.2004	+
PBANKA_093370	mitogen-activated protein kinase 2	Expressed in male gametocytes	0.0795	0.1866	-
PBANKA_090620	conserved Plasmodium protein, unknown function	Expressed in male gametocytes	0.1390	0.1266	-

PBANKA_062360	BIR protein	Expressed in male gametocytes	0.1512	0.9940	-
PBANKA_132900	conserved Plasmodium protein, unknown function	Expressed in male gametocytes	0.1318	0.2127	-
PBANKA_110900	RNA polymerase I, putative	Expressed in male gametocytes	0.2463	0.1860	-
PBANKA_052390	conserved Plasmodium protein, unknown function	Expressed in male gametocytes	0.2035	0.0294	+
PBANKA_132690	DNA polymerase alpha subunit, putative	Expressed in male gametocytes	0.0898	0.0720	-
PBANKA_050730	dynein heavy chain, putative	Expressed in male gametocytes	0.0832	0.1088	-
PBANKA_132620	conserved Plasmodium protein, unknown function	Expressed in male gametocytes	0.1066	0.1788	+
PBANKA_141070	conserved Plasmodium protein, unknown function	Expressed in male gametocytes	0.0976	0.1492	-
PBANKA_092150	small nuclear ribonucleoprotein F, putative	Expressed in male gametocytes	0.1656	0.0000	-
PBANKA_051460	glycoprotease, putative	Expressed in male gametocytes	0.2155	0.1433	-
PBANKA_110880	phosphatase 1 regulatory subunit, putative	Expressed in male gametocytes	0.1199	0.1553	-
PBANKA_120480	thioredoxin, putative	Expressed in male gametocytes	0.2765	0.0394	-
PBANKA_080520	cytoskeleton associated protein, putative	Expressed in male gametocytes	0.1019	0.2820	-
PBANKA_082460	conserved Plasmodium protein, unknown function	Expressed in male gametocytes	0.2542	0.1383	-
PBANKA_030600	transmission-blocking target antigen, putative	Expressed in male gametocytes	0.1215	0.2193	-
PBANKA_120490	conserved Plasmodium protein, unknown function	Expressed in male gametocytes	0.2682	0.3850	+
PBANKA_103080	conserved Plasmodium protein, unknown function	Expressed in male gametocytes	0.1319	0.2903	-
PBANKA_071210	DNA helicase, putative	Expressed in male gametocytes	0.3133	0.0797	-
PBANKA_142940	dynein light chain 1 putative	Expressed in male gametocytes	0.1705	0.0000	-
PBANKA_031050	conserved Plasmodium protein, unknown function	Expressed in male gametocytes	0.1837	0.4022	-
PBANKA_140290	conserved Plasmodium protein, unknown function	Expressed in male gametocytes	0.1584	0.1749	-
PBANKA_101460	phosphotyrosyl phosphatase activator, putative	Expressed in male gametocytes	0.2751	0.1129	-
PBANKA_113750	actin-depolymerizing factor, putative	Expressed in male gametocytes	0.0656	0.1982	-
PBANKA_070640	conserved Plasmodium protein, unknown function	Expressed in male gametocytes	0.1392	0.1491	-
PBANKA_145690	translation initiation factor SUI1, putative	Expressed in male gametocytes	0.0158	0.0000	-
PBANKA_100610	flap endonuclease 1 putative	Expressed in male gametocytes	0.0980	0.1286	-
PBANKA_060280	ubiquitin-conjugating enzyme e2, putative	Expressed in male gametocytes	0.2647	0.0000	-
PBANKA_070810	outer arm dynein light chain 2 putative	Expressed in male gametocytes	0.3769	0.0229	-
PBANKA_124180	minichromosome maintenance protein 3 putative	Expressed in male gametocytes	0.3238	0.0106	-
PBANKA_082300	ubiquitin-like protein, putative	Expressed in male gametocytes	0.2246	0.2978	-
PBANKA_145880	kinesin, putative	Expressed in male gametocytes	0.2409	0.1022	-
PBANKA_122340	conserved Plasmodium protein, unknown function	Expressed in male gametocytes	0.1794	0.2405	+
PBANKA_122160	cell division cycle ATPase, putative	Expressed in male gametocytes	0.2012	0.1093	-
PBANKA_061020	DNA replication licensing factor MCM5, putative	Expressed in male gametocytes	0.1281	0.0192	-

PBANKA_094230	conserved Plasmodium protein, unknown function	Expressed in male gametocytes	0.2218	0.1825	-
PBANKA_061160	ribonucleoside-diphosphate reductase, large subunit, putative	Expressed in male gametocytes	0.2610	0.0431	-
PBANKA_062200	conserved Plasmodium protein, unknown function	Expressed in male gametocytes	0.2507	0.1402	-
PBANKA_103790	conserved Plasmodium protein, unknown function	Expressed in male gametocytes	0.1108	0.4474	-
PBANKA_103410	DNA repair helicase, putative	Expressed in male gametocytes	0.1535	0.0677	-
PBANKA_070540	translation initiation factor IF-3, putative	Expressed in male gametocytes	0.0509	0.1165	+
PBANKA_103900	conserved Plasmodium protein, unknown function	Expressed in male gametocytes	0.1127	0.3754	-
PBANKA_130810	calmodulin-like protein	Expressed in male gametocytes	0.0713	0.2169	-
PBANKA_121840	SNARE protein, putative	Expressed in male gametocytes	0.0488	0.0836	+
PBANKA_070600	conserved protein, unknown function	Expressed in male gametocytes	0.1103	0.1557	-
PBANKA_040560	EB1 homolog, putative	Expressed in male gametocytes	0.2524	0.1831	-
PBANKA_050780	conserved Plasmodium protein, unknown function	Expressed in male gametocytes	0.0704	0.2093	-
PBANKA_121150	conserved Plasmodium protein, unknown function	Expressed in male gametocytes	0.1011	0.2464	-
PBANKA_050970	conserved Plasmodium protein, unknown function	Expressed in male gametocytes	0.3124	0.1248	-
PBANKA_135640	DNA helicase, putative, pseudogene	Expressed in male gametocytes	0.1073	0.2269	-
PBANKA_122250	plasmepsin X	Expressed in male gametocytes	0.1428	0.1613	-
PBANKA_060560	conserved Plasmodium protein, unknown function	Expressed in male gametocytes	0.1127	0.0152	-
PBANKA_140240	conserved Plasmodium protein, unknown function	Expressed in male gametocytes	0.1074	0.1207	-
PBANKA_141330	ubiquitin-like protein, putative	Expressed in male gametocytes	0.2310	0.1557	-
PBANKA_101220	methyltransferase, putative	Expressed in male gametocytes	0.4235	0.0676	-
PBANKA_100340	lysine decarboxylase, putative	Expressed in male gametocytes	0.0658	0.1954	-
PBANKA_142830	conserved Plasmodium protein, unknown function	Expressed in male gametocytes	0.0842	0.3825	-
PBANKA_090690	conserved Plasmodium protein, unknown function	Expressed in male gametocytes	0.1489	0.1576	-
PBANKA_113690	SUMO ligase, putative	Expressed in male gametocytes	0.1243	0.1719	-
PBANKA_131670	conserved Plasmodium protein, unknown function	Expressed in male gametocytes	0.0761	0.2237	+
PBANKA_020630	centrin, putative	Expressed in male gametocytes	0.1347	0.0241	-
PBANKA_132010	conserved Plasmodium protein, unknown function	Expressed in male gametocytes	0.0899	0.2160	-
PBANKA_141690	chromosome segregation protein, putative	Expressed in male gametocytes	0.0730	0.1107	-
PBANKA_093860	casein kinase II, alpha subunit, putative	Expressed in male gametocytes	0.1658	0.0126	-
PBANKA_080230	ubiquitin transferase, putative	Expressed in male gametocytes	0.1614	0.2618	-
PBANKA_145460	conserved Plasmodium protein, unknown function	Expressed in male gametocytes	0.0867	0.2845	-
PBANKA_110870	chromosome condensation protein, putative	Expressed in male gametocytes	0.2285	0.1154	-
PBANKA_110960	conserved Plasmodium protein, unknown function	Expressed in male gametocytes	0.1038	0.3073	-
PBANKA_111040	conserved Plasmodium protein, unknown function	Expressed in male gametocytes	0.0598	0.1289	-

PBANKA_092770	conserved Plasmodium protein, unknown function	Expressed in male gametocytes	0.0514	3..8242	-
PBANKA_091740	PF16 protein, putative	Expressed in male gametocytes	0.1561	0.0394	-
PBANKA_136160	conserved Plasmodium protein, unknown function	Expressed in male gametocytes	0.2267	0.1671	+
PBANKA_103370	conserved Plasmodium protein, unknown function	Expressed in male gametocytes	0.1220	0.2805	-
PBANKA_130180	conserved Plasmodium protein, unknown function	Expressed in male gametocytes	0.1217	0.3371	-
PBANKA_010420	calcium-binding protein, putative	Expressed in male gametocytes	0.2831	0.0310	-
PBANKA_122740	protein phosphatase, putative	Expressed in male gametocytes	0.2912	0.0723	-
PBANKA_113320	cdc2-related kinase 2	Expressed in male gametocytes	0.0890	0.0571	-
PBANKA_140500	conserved Plasmodium protein, unknown function	Expressed in male gametocytes	0.1017	0.3476	-
PBANKA_120940	conserved Plasmodium protein, unknown function	Expressed in male gametocytes	0.0436	0.3547	-
PBANKA_051740	myb2 protein, putative	Expressed in male gametocytes	0.1063	0.1590	-
PBANKA_110160	chromosome assembly factor 1 putative	Expressed in male gametocytes	0.1062	0.2603	-
PBANKA_050810	chromodomain-helicase-DNA-binding protein 1 putative	Expressed in male gametocytes	0.2076	0.0818	-
PBANKA_031520	small nuclear ribonucleoprotein, putative	Expressed in male gametocytes	0.4925	0.0313	-
PBANKA_082290	conserved Plasmodium protein, unknown function	Expressed in male gametocytes	0.1898	0.1292	-
PBANKA_062130	conserved Plasmodium protein, unknown function	Expressed in male gametocytes	0.4333	0.1406	-
PBANKA_092080	conserved Plasmodium protein, unknown function	Expressed in male gametocytes	0.3516	0.1213	-
PBANKA_113260	ubiquitin conjugating enzyme, putative	Expressed in male gametocytes	0.1716	0.0159	-
PBANKA_092160	conserved Plasmodium protein, unknown function	Expressed in male gametocytes	0.0888	0.0837	-
PBANKA_130970	helicase, putative	Expressed in male gametocytes	0.0950	0.1445	-
PBANKA_131540	protein phosphatase 2b regulatory subunit, putative	Expressed in male gametocytes	0.0717	0.0399	-
PBANKA_142770	DNA helicase, putative	Expressed in male gametocytes	0.1029	0.0691	-
PBANKA_091170	subtilisin-like protease 2	Expressed in male gametocytes	0.1090	0.3927	-
PBANKA_080300	origin recognition complex subunit 2 putative	Expressed in male gametocytes	0.1249	0.0000	-
PBANKA_091080	conserved Plasmodium protein, unknown function	Expressed in male gametocytes	0.1413	0.1395	-
PBANKA_093900	ion channel protein, putative	Expressed in male gametocytes	0.1147	0.1963	+
PBANKA_113380	DNA helicase, putative	Expressed in male gametocytes	0.0985	0.0693	-
PBANKA_052070	conserved Plasmodium protein, unknown function	Expressed in male gametocytes	0.0856	0.1626	-
PBANKA_136150	conserved Plasmodium protein, unknown function	Expressed in male gametocytes	0.1846	0.0644	-
PBANKA_102240	dynein-associated protein, putative	Expressed in male gametocytes	0.2321	0.1911	-
PBANKA_061400	conserved Plasmodium protein, unknown function	Expressed in male gametocytes	0.3477	0.0935	-
PBANKA_130450	conserved Plasmodium protein, unknown function	Expressed in male gametocytes	0.1206	0.1138	-
PBANKA_134810	conserved Plasmodium protein, unknown function	Expressed in male gametocytes	0.1824	0.3024	+
PBANKA_051390	conserved Plasmodium protein, unknown function	Expressed in male gametocytes	0.2006	0.3147	-

PBANKA_120390	DnaJ protein, putative	Expressed in male gametocytes	0.2389	0.1494	-
PBANKA_143240	perforin like protein 2	Expressed in male gametocytes	0.1151	0.4260	-
PBANKA_070200	conserved Plasmodium protein, unknown function	Expressed in male gametocytes	0.1664	0.1893	-
PBANKA_093360	dynein light chain 2 putative	Expressed in male gametocytes	0.0250	0.4600	-
PBANKA_133130	conserved Plasmodium protein, unknown function	Expressed in male gametocytes	0.1425	0.3286	-
PBANKA_134940	conserved Plasmodium protein, unknown function	Expressed in male gametocytes	0.1594	0.1407	+
PBANKA_041610	dynein heavy chain, putative	Expressed in male gametocytes	0.1187	0.0800	-
PBANKA_083270	adenylate kinase, putative	Expressed in male gametocytes	0.2389	0.1462	-
PBANKA_061320	DNA polymerase alpha, putative	Expressed in male gametocytes	0.2670	0.1065	-
PBANKA_091300	conserved Plasmodium protein, unknown function	Expressed in male gametocytes	0.1063	0.2044	-
PBANKA_112170	leucyl tRNA synthase, putative	Expressed in male gametocytes	0.2736	0.1358	-
PBANKA_061860	conserved Plasmodium protein, unknown function	Expressed in male gametocytes	0.1802	0.1842	+
PBANKA_092510	conserved Plasmodium protein, unknown function	Expressed in male gametocytes	0.1796	0.0701	-
PBANKA_030710	conserved Plasmodium protein, unknown function	Expressed in male gametocytes	0.2401	0.1942	+
PBANKA_112280	ribonuclease HII, putative	Expressed in male gametocytes	0.2668	0.0185	-
PBANKA_144470	conserved Plasmodium protein, unknown function	Expressed in male gametocytes	0.0648	0.2744	-
PBANKA_010270	conserved Plasmodium protein, unknown function	Expressed in male gametocytes	0.0948	0.2139	-
PBANKA_101390	conserved Plasmodium protein, unknown function	Expressed in male gametocytes	0.1264	0.2544	-
PBANKA_100800	conserved Plasmodium protein, unknown function	Expressed in male gametocytes	0.0791	0.0369	-
PBANKA_110800	transcription factor IIb, putative	Expressed in male gametocytes	0.1531	0.1920	-
PBANKA_030330	conserved Plasmodium protein, unknown function	Expressed in male gametocytes	0.3525	0.0829	-
PBANKA_093990	lsm4 homologue, putative	Expressed in male gametocytes	0.1561	0.1016	-
PBANKA_030590	conserved Plasmodium protein, unknown function	Expressed in male gametocytes	0.1652	0.1690	+
PBANKA_130590	ADP-ribosylation-like factor, putative	Expressed in male gametocytes	0.0828	0.0000	-
PBANKA_020270	kinesin, putative	Expressed in male gametocytes	0.1437	0.2669	-
PBANKA_052060	enhancer of rudimentary homolog, putative	Expressed in male gametocytes	0.1181	0.1689	-
PBANKA_141640	conserved Plasmodium protein, unknown function	Expressed in male gametocytes	0.0994	0.3085	-
PBANKA_132570	thioredoxin-like protein, putative	Expressed in male gametocytes	0.0767	0.0000	-
PBANKA_060200	origin recognition complex 1 protein, putative	Expressed in male gametocytes	0.2816	0.1072	-
PBANKA_143150	conserved Plasmodium protein, unknown function	Expressed in male gametocytes	0.0932	0.0899	-
PBANKA_134890	conserved Plasmodium protein, unknown function	Expressed in male gametocytes	0.1970	0.0791	-
PBANKA_050780	conserved Plasmodium protein, unknown function	Expressed in male gametocytes	0.0925	0.2626	-
PBANKA_123400	vacuolar ATP synthetase, putative	Expressed in male gametocytes	0.1296	0.0000	+
PBANKA_010620	conserved Plasmodium protein, unknown function	Expressed in male gametocytes	0.1944	0.1249	-

PBANKA_143940	chromatin assembly protein, putative	Expressed in male gametocytes	0.2887	0.1115	-
PBANKA_112910	DNA polymerase epsilon, putative	Expressed in male gametocytes	0.1021	0.1045	-
PBANKA_141210	septum formation protein MAF homologue, putative	Expressed in male gametocytes	0.1055	0.1776	-
PBANKA_041430	conserved Plasmodium protein, unknown function	Expressed in male gametocytes	0.1127	0.1538	-
PBANKA_123910	conserved Plasmodium protein, unknown function	Expressed in male gametocytes	0.2123	0.4600	-
PBANKA_091360	conserved Plasmodium protein, unknown function	Expressed in male gametocytes	0.1276	0.2973	-
PBANKA_112530	conserved Plasmodium protein, unknown function	Expressed in male gametocytes	0.1254	0.0360	-
PBANKA_031250	origin recognition complex subunit 5 putative	Expressed in male gametocytes	0.2503	0.0686	-
PBANKA_121850	conserved Plasmodium protein, unknown function	Expressed in male gametocytes	0.0675	0.1632	-
PBANKA_092570	conserved Plasmodium protein, unknown function	Expressed in male gametocytes	0.1053	0.2242	-
PBANKA_131200	conserved Plasmodium protein, unknown function	Expressed in male gametocytes	0.1131	0.2066	-
PBANKA_132240	conserved Plasmodium protein, unknown function	Expressed in male gametocytes	0.0474	0.0817	-
PBANKA_130440	allantoicase, putative	Expressed in male gametocytes	0.1174	0.0891	-
PBANKA_083030	conserved Plasmodium protein, unknown function	Expressed in male gametocytes	0.1528	0.1050	-
PBANKA_090890	vacuolar ATP synthase subunit f, putative	Expressed in male gametocytes	0.3012	0.0000	-
PBANKA_140660	CCAAT-binding transcription factor, putative	Expressed in male gametocytes	0.0953	0.2013	-
PBANKA_082820	conserved Plasmodium protein, unknown function	Expressed in male gametocytes	0.2465	0.2752	-
PBANKA_130800	conserved Plasmodium protein, unknown function	Expressed in male gametocytes	0.0782	0.4577	-
PBANKA_071970	conserved Plasmodium protein, unknown function	Expressed in male gametocytes	0.1573	0.0661	-
PBANKA_113640	conserved Plasmodium protein, unknown function	Expressed in male gametocytes	0.0845	0.1217	+
PBANKA_090880	conserved Plasmodium protein, unknown function	Expressed in male gametocytes	0.1203	0.2374	-
PBANKA_114140	conserved Plasmodium protein, unknown function	Expressed in male gametocytes	0.1003	0.2868	-
PBANKA_103570	conserved Plasmodium protein, unknown function	Expressed in male gametocytes	0.1975	0.1264	-
PBANKA_040430	P-loop containing nucleoside triphospahte hydrolase, putative	Expressed in male gametocytes	0.2372	0.1943	-
PBANKA_145040	conserved Plasmodium protein, unknown function	Expressed in male gametocytes	0.2594	0.1586	-
PBANKA_110650	rhomboid protease, putative	Expressed in male gametocytes	0.1978	0.0745	+
PBANKA_142070	conserved Plasmodium protein, unknown function	Expressed in male gametocytes	0.0651	0.0000	-
PBANKA_133750	conserved Plasmodium protein, unknown function	Expressed in male gametocytes	0.2505	0.2272	-
PBANKA_110290	conserved Plasmodium protein, unknown function	Expressed in male gametocytes	0.1284	0.1591	-
PBANKA_041440	formate-nitrite transporter, putative	Expressed in male gametocytes	0.3329	0.0702	+
PBANKA_113360	dynein light chain, putative	Expressed in male gametocytes	0.1127	0.1041	-
PBANKA_081620	conserved Plasmodium protein, unknown function	Expressed in male gametocytes	0.0644	0.0000	-
PBANKA_081850	conserved Plasmodium protein, unknown function	Expressed in male gametocytes	0.2084	0.1383	-
PBANKA_103040	conserved Plasmodium protein, unknown function	Expressed in male gametocytes	0.0898	0.3595	-

PBANKA_111070	stearoyl-CoA delta 9 desaturase, putative, pseudogene	Expressed in male gametocytes	0.1277	0.1553	+
PBANKA_122380	vacuolar proton-translocating ATPase subunit A, putative	Expressed in male gametocytes	0.2443	0.0706	+
PBANKA_100970	conserved Plasmodium protein, unknown function	Expressed in male gametocytes	0.0429	1..1291	-
PBANKA_120990	conserved Plasmodium protein, unknown function	Expressed in male gametocytes	0.0715	0.7801	+
PBANKA_130040	BIR protein	Expressed in male gametocytes	0.1356	1..604	+
PBANKA_093960	MIF4G domain protein, putative	Expressed in male gametocytes	0.2401	0.1108	-
PBANKA_123840	conserved Plasmodium protein, unknown function	Expressed in male gametocytes	0.1993	0.0366	-
PBANKA_080150	conserved Plasmodium protein, unknown function	Expressed in male gametocytes	0.1120	0.0896	-
PBANKA_140960	conserved Plasmodium protein, unknown function	Expressed in male gametocytes	0.0574	0.5149	-
PBANKA_010120	elongation factor G, putative	Expressed in male gametocytes	0.1196	0.1493	-
PBANKA_134880	conserved Plasmodium protein, unknown function	Expressed in male gametocytes	0.2825	0.0852	-
PBANKA_060740	conserved Plasmodium protein, unknown function	Expressed in male gametocytes	0.1601	0.1237	-
PBANKA_081440	conserved Plasmodium protein, unknown function	Expressed in female gametocytes	0.1533	0.2450	-
PBANKA_100110	ubiquitin-specific protease, putative	Expressed in female gametocytes	0.0982	0.1099	-
PBANKA_082000	DNAJ-like molecular chaperone protein, putative	Expressed in female gametocytes	0.2270	0.0894	+
PBANKA_020930	actin-related protein	Expressed in female gametocytes	0.1938	0.0000	-
PBANKA_091760	structural maintenance of chromosome protein, putative, fragment	Expressed in female gametocytes	0.2907	0.0119	-
PBANKA_130070	'LCCL/lectin adhesive-like protein 2'	Expressed in female gametocytes	0.0973	0.1664	+
PBANKA_143490	phosphatidylethanolamine-binding protein, putative	Expressed in female gametocytes	0.1988	0.0964	-
PBANKA_094360	60S acidic ribosomal protein, putative	Expressed in female gametocytes	0.1218	0.1261	-
PBANKA_103520	Scavenger Receptor-like protein	Expressed in female gametocytes	0.1329	0.1548	+
PBANKA_136470	conserved Plasmodium protein, unknown function	Expressed in female gametocytes	0.1681	0.2126	-
PBANKA_100910	conserved Plasmodium protein, unknown function	Expressed in female gametocytes	0.0905	0.1253	-
PBANKA_071540	conserved Plasmodium protein, unknown function	Expressed in female gametocytes	0.1981	0.0700	-
PBANKA_133110	conserved Plasmodium protein, unknown function	Expressed in female gametocytes	0.2265	0.0990	-
PBANKA_142930	conserved Plasmodium protein, unknown function	Expressed in female gametocytes	0.0796	0.2242	+
PBANKA_061190	histidine triad protein, putative	Expressed in female gametocytes	0.1184	0.0775	-
PBANKA_121310	prohibitin, putative	Expressed in female gametocytes	0.1085	0.0000	-
PBANKA_040980	conserved Plasmodium protein, unknown function	Expressed in female gametocytes	0.1772	0.1407	-
PBANKA_021400	dynein heavy chain, putative	Expressed in female gametocytes	0.1112	0.0918	-
PBANKA_093390	glyoxalase I, putative	Expressed in female gametocytes	0.0799	0.0985	-
PBANKA_146300	osmiophilic body protein	Expressed in female gametocytes	0.1545	0.4111	+
PBANKA_143520	conserved Plasmodium protein, unknown function	Expressed in female gametocytes	0.0708	0.2488	-
PBANKA_080940	P1 nuclease, putative	Expressed in female gametocytes	0.1974	0.0851	+

PBANKA_030700	60S ribosomal protein L37ae, putative	Expressed in female gametocytes	0.2616	0.0210	-
PBANKA_140680	40S ribosomal protein S27, putative	Expressed in female gametocytes	0.1776	0.0000	-
PBANKA_122820	glutamate dehydrogenase, putative	Expressed in female gametocytes	0.2119	0.0914	-
PBANKA_010230	calcium antiporter, putative	Expressed in female gametocytes	0.1743	0.0382	+
PBANKA_101380	cytochrome c oxidase subunit II precursor, putative	Expressed in female gametocytes	0.1102	0.0788	-
PBANKA_141910	dihydrolipamide succinyltransferase component of 2-oxoglutarate dehydrogenase,	Expressed in female gametocytes	0.0683	0.2202	-
PBANKA_082860	cytochrome c oxidase subunit, putative	Expressed in female gametocytes	0.2027	0.0188	-
PBANKA_143200	signal recognition particle 19 kD protein, putative	Expressed in female gametocytes	0.0648	0.2405	-
PBANKA_052240	steroid dehydrogenase, putative	Expressed in female gametocytes	0.1609	0.1104	+
PBANKA_031560	40S ribosomal protein S30, putative	Expressed in female gametocytes	0.1054	0.0000	-
PBANKA_020450	LCCL domain-containing protein	Expressed in female gametocytes	0.1667	0.1066	-
PBANKA_082790	conserved Plasmodium protein, unknown function	Expressed in female gametocytes	0.3335	0.0947	-
PBANKA_120110	conserved Plasmodium protein, unknown function	Expressed in female gametocytes	0.0720	0.4091	-
PBANKA_040730	conserved Plasmodium protein, unknown function	Expressed in female gametocytes	0.0884	0.2804	+
PBANKA_131380	signal recognition particle 54 kDa protein, putative	Expressed in female gametocytes	0.2727	0.0311	-
PBANKA_083000	RNA-binding protein, putative	Expressed in female gametocytes	0.2570	0.0348	-
PBANKA_081600	NADH dehydrogenase, putative	Expressed in female gametocytes	0.2694	0.1712	-
PBANKA_010080	conserved Plasmodium protein, unknown function	Expressed in female gametocytes	0.0915	0.3518	-
PBANKA_144700	cytochrome b5, putative	Expressed in female gametocytes	0.0547	0.8393	-
PBANKA_081050	apoptosis-related protein, putative	Expressed in female gametocytes	0.2151	0.0452	-
PBANKA_101020	methionine aminopeptidase, type II, putative	Expressed in female gametocytes	0.0940	0.1713	-
PBANKA_114170	60S ribosomal protein L40/UBI, putative	Expressed in female gametocytes	0.2474	0.0272	-
PBANKA_123130	metabolite/drug transporter, putative	Expressed in female gametocytes	0.0993	0.1227	+
PBANKA_092360	co-chaperone GrpE, putative	Expressed in female gametocytes	0.1619	0.1390	-
PBANKA_071520	eukaryotic translation initiation factor, putative	Expressed in female gametocytes	0.0749	0.0267	-
PBANKA_092910	conserved Plasmodium protein, unknown function	Expressed in female gametocytes	0.2603	0.1302	-
PBANKA_130760	dephospho-CoA kinase, putative	Expressed in female gametocytes	0.0787	0.0409	+
PBANKA_082870	phosphatidylserine decarboxylase, putative	Expressed in female gametocytes	0.3727	0.0442	+
PBANKA_132510	conserved Plasmodium protein, unknown function	Expressed in female gametocytes	0.1774	0.2033	-
PBANKA_141440	exportin-T, putative	Expressed in female gametocytes	0.2051	0.0836	-
PBANKA_102280	glycine--tRNA ligase, putative	Expressed in female gametocytes	0.2553	0.1129	-
PBANKA_144840	conserved Plasmodium protein, unknown function	Expressed in female gametocytes	0.3782	0.0690	-
PBANKA_132020	conserved Plasmodium protein, unknown function	Expressed in female gametocytes	0.0933	0.2479	-
PBANKA_071660	conserved Plasmodium protein, unknown function	Expressed in female gametocytes	0.1955	0.0589	-

PBANKA_061670	serine/threonine protein kinase	Expressed in female gametocytes	0.2619	0.0000	-
PBANKA_101440	conserved Plasmodium protein, unknown function	Expressed in female gametocytes	0.2125	0.1705	-
PBANKA_111270	conserved Plasmodium protein, unknown function	Expressed in female gametocytes	0.2253	0.2084	+
PBANKA_120210	conserved Plasmodium protein, unknown function	Expressed in female gametocytes	0.1171	0.2372	-
PBANKA_142440	CS domain protein, putative	Expressed in female gametocytes	0.1142	0.1927	-
PBANKA_111350	Rab1a, putative	Expressed in female gametocytes	0.1428	0.0380	-
PBANKA_124170	conserved Plasmodium protein, unknown function	Expressed in female gametocytes	0.1793	0.3073	+
PBANKA_132840	conserved Plasmodium protein, unknown function	Expressed in female gametocytes	0.0716	0.2554	+
PBANKA_134560	elongation factor Tu, putative	Expressed in female gametocytes	0.1829	0.0679	-
PBANKA_041060	60S ribosomal protein L26, putative	Expressed in female gametocytes	0.1324	0.0000	-
PBANKA_050850	conserved Plasmodium protein, unknown function	Expressed in female gametocytes	0.3029	0.0000	-
PBANKA_135890	conserved Plasmodium protein, unknown function	Expressed in female gametocytes	0.0943	0.1605	-
PBANKA_144950	conserved Plasmodium protein, unknown function	Expressed in female gametocytes	0.0947	0.1125	-
PBANKA_102520	pseudouridine synthase, putative	Expressed in female gametocytes	0.1061	0.0551	-
PBANKA_093850	succinyl-CoA synthetase alpha subunit, putative	Expressed in female gametocytes	0.1509	0.0326	-
PBANKA_100400	cytosolic glyoxalase II, putative	Expressed in female gametocytes	0.1277	0.0481	-
PBANKA_100950	NAD(P)H-dependent glutamate synthase, putative	Expressed in female gametocytes	0.0915	0.1421	-
PBANKA_131920	gamma-adaptin, putative	Expressed in female gametocytes	0.0980	0.2893	-
PBANKA_130350	conserved Plasmodium protein, unknown function	Expressed in female gametocytes	0.1216	0.1270	+
PBANKA_050590	conserved Plasmodium protein, unknown function	Expressed in female gametocytes	0.2123	0.2156	-
PBANKA_135990	conserved Plasmodium protein, unknown function	Expressed in female gametocytes	0.1339	0.0398	-
PBANKA_083180	elongation factor Tu, putative	Expressed in female gametocytes	0.1488	0.2213	-
PBANKA_100720	replication factor a protein, putative	Expressed in female gametocytes	0.1321	0.1614	-
PBANKA_061560	conserved Plasmodium protein, unknown function	Expressed in female gametocytes	0.1160	0.1340	-
PBANKA_041640	conserved Plasmodium protein, unknown function	Expressed in female gametocytes	0.1900	0.0281	-
PBANKA_103160	conserved Plasmodium protein, unknown function	Expressed in female gametocytes	0.0972	0.4263	+
PBANKA_112490	oxidoreductase, putative	Expressed in female gametocytes	0.1216	0.1169	+
PBANKA_080900	translation initiation factor SUI1, putative	Expressed in female gametocytes	0.1558	0.1382	-
PBANKA_123060	conserved Plasmodium protein, unknown function	Expressed in female gametocytes	0.2616	0.2111	-
PBANKA_100810	GTPase, putative	Expressed in female gametocytes	0.0887	0.2266	-
PBANKA_091470	conserved Plasmodium protein, unknown function	Expressed in female gametocytes	0.2196	0.1373	-
PBANKA_142030	conserved Plasmodium protein, unknown function	Expressed in female gametocytes	0.0407	0.5843	+
PBANKA_122680	DNA repair protein rad54, putative	Expressed in female gametocytes	0.1960	0.0689	-
PBANKA_050930	glucosamine--fructose-6-phosphate aminotransferase, putative	Expressed in female gametocytes	0.1403	0.1145	-

PBANKA_124270	beta adaptin protein, putative	Expressed in female gametocytes	0.1952	0.0746	-
PBANKA_080760	40S ribosomal protein S11, putative	Expressed in female gametocytes	0.0359	0.0000	-
PBANKA_135970	Pfs47 homologue, putative	Expressed in female gametocytes	0.1247	0.5902	+
PBANKA_041490	dynein light intermediate chain 2 putative	Expressed in female gametocytes	0.2608	0.1833	-
PBANKA_050170	phosphomannomutase, putative	Expressed in female gametocytes	0.0721	0.2356	-
PBANKA_030800	Rab5a, GTPase, putative	Expressed in female gametocytes	0.1174	0.1141	-
PBANKA_040840	conserved Plasmodium protein, unknown function	Expressed in male and female gametocytes	0.1881	0.1345	-
PBANKA_141560	DNA replication licensing factor, putative	Expressed in male and female gametocytes	0.0911	0.0576	-
PBANKA_040380	conserved Plasmodium protein, unknown function	Expressed in male and female gametocytes	0.3019	0.2043	+
PBANKA_110710	subtilisin-like protease 1 putative	Expressed in male and female gametocytes	0.1385	0.2427	-
PBANKA_090560	translation initiation factor eIF-1A, putative	Expressed in male and female gametocytes	0.2125	0.0326	-
PBANKA_082970	guanylate kinase, putative	Expressed in male and female gametocytes	0.3517	0.0693	-
PBANKA_103010	actin II, putative	Expressed in male and female gametocytes	0.1482	0.0159	-
PBANKA_143220	male development gene 1	Expressed in male and female gametocytes	0.0971	0.3408	+
PBANKA_110510	DNA repair protein, putative	Expressed in male and female gametocytes	0.2966	0.0334	-
PBANKA_071780	60S ribosomal protein L15, putative	Expressed in male and female gametocytes	0.3260	0.0000	-
PBANKA_135450	60S ribosomal protein L18-2, putative	Expressed in male and female gametocytes	0.0590	0.3080	-
PBANKA_145780	ADP-ribosylation factor, putative	Expressed in male and female gametocytes	0.0394	0.4366	-
PBANKA_130250	conserved protein, unknown function	Expressed in male and female gametocytes	0.0416	0.2780	-
PBANKA_102390	glutathione s-transferase, putative	Expressed in male and female gametocytes	0.0776	0.0482	-
PBANKA_130130	conserved Plasmodium protein, unknown function	Expressed in male and female gametocytes	0.0983	0.0851	-
PBANKA_061920	secreted ookinete protein, putative	Expressed in male and female gametocytes	0.0812	0.3306	+
PBANKA_071880	small GTP-binding protein sar1, putative	Expressed in male and female gametocytes	0.1722	0.0292	-
PBANKA_010540	coatomer alpha subunit, putative	Expressed in male and female gametocytes	0.2508	0.1418	-
PBANKA_111520	conserved Plasmodium protein, unknown function	Expressed in male and female gametocytes	0.1588	0.1648	-
PBANKA_092820	conserved Plasmodium protein, unknown function	Expressed in male and female gametocytes	0.1925	0.1931	-
PBANKA_142670	dihydropteroate synthetase, putative	Expressed in male and female gametocytes	0.1143	0.2043	-
PBANKA_040670	activator of Hsp90 ATPase, putative	Expressed in male and female gametocytes	0.2247	0.0948	-
PBANKA_021450	conserved Plasmodium protein, unknown function	Expressed in male and female gametocytes	0.1146	0.0801	-
PBANKA_101790	phosphoenolpyruvate carboxylase, putative	Expressed in male and female gametocytes	0.2222	0.0967	-
PBANKA_103230	cytidine triphosphate synthetase, putative	Expressed in male and female gametocytes	0.0669	0.2799	-
PBANKA_132120	ATPase, putative	Expressed in male and female gametocytes	0.0662	0.1729	-
PBANKA_094110	conserved Plasmodium protein, unknown function	Expressed in male and female gametocytes	0.0553	0.0000	+
PBANKA_123820	DnaJ protein, putative	Expressed in male and female gametocytes	0.1095	0.1032	+

PBANKA_140510	vacuolar ATP synthase subunit h, putative	Expressed in male and female gametocytes	0.1151	0.0348	-
PBANKA_122240	ubiquitin regulatory protein, putative	Expressed in male and female gametocytes	0.0245	0.6122	-
PBANKA_133840	vacuolar ATP synthase subunit g, putative	Expressed in male and female gametocytes	0.0653	0.0635	-
PBANKA_080310	DNA replication licensing factor, putative	Expressed in male and female gametocytes	0.1096	0.0414	-
PBANKA_120270	RNA-binding protein, putative	Expressed in male and female gametocytes	0.1616	0.2856	-
PBANKA_102350	conserved Plasmodium protein, unknown function	Expressed in male and female gametocytes	0.1837	0.0498	-
PBANKA_111340	secreted ookinete protein, putative	Expressed in male and female gametocytes	0.1348	0.2698	-
PBANKA_114290	Skp1 family protein, putative	Expressed in male and female gametocytes	0.2186	0.0411	-
PBANKA_121770	development of zygote inhibited	Expressed in male and female gametocytes	0.0820	0.0000	-
PBANKA_092430	endoplasmic reticulum oxidoreductin, putative	Expressed in male and female gametocytes	0.4350	0.0539	+
PBANKA_112020	pyridoxine/pyridoxal 5-phosphate biosynthesis enzyme, putative	Expressed in male and female gametocytes	0.1306	0.0368	-
PBANKA_113160	replication licensing factor, putative	Expressed in male and female gametocytes	0.1902	0.1096	-
PBANKA_144900	conserved Plasmodium protein, unknown function	Expressed in male and female gametocytes	0.2087	0.2401	-
PBANKA_050690	conserved Plasmodium protein, unknown function	Expressed in male and female gametocytes	0.0676	0.5883	-
PBANKA_131270	conserved Plasmodium protein, unknown function	Expressed in male and female gametocytes	0.1264	0.2189	+
PBANKA_080430	DNA mismatch repair protein	Expressed in male and female gametocytes	0.0690	0.2593	-
PBANKA_123790	mitochondrial processing peptidase alpha subunit, putative	Expressed in male and female gametocytes	0.1076	0.0376	-
PBANKA_100200	conserved Plasmodium protein, unknown function	Expressed in male and female gametocytes	0.1152	0.1274	-
PBANKA_145160	ubiquitin activating enzyme, putative	Expressed in male and female gametocytes	0.0974	0.1809	-
PBANKA_020720	vacuolar ATP synthase subunit c, putative	Expressed in male and female gametocytes	0.3190	0.0476	-
PBANKA_132070	signal peptide peptidase	Expressed in male and female gametocytes	0.0937	0.0854	+
PBANKA_083530	vacuolar ATP synthase subunit e, putative	Expressed in male and female gametocytes	0.2257	0.1460	-
PBANKA_041040	glycogen synthase kinase 3 putative	Expressed in male and female gametocytes	0.1736	0.1244	+
PBANKA_142130	4-nitrophenylphosphatase, putative	Expressed in male and female gametocytes	0.1038	0.1708	-
PBANKA_050440	conserved Plasmodium protein, unknown function	Expressed in male and female gametocytes	0.1989	0.2388	+
PBANKA_135630	conserved Plasmodium protein, unknown function	Expressed in male and female gametocytes	0.0787	0.2081	-
PBANKA_136200	glutamate - tRNA ligase, putative	Expressed in male and female gametocytes	0.1883	0.1005	-
PBANKA_146420	thymidylate kinase, putative	Expressed in male and female gametocytes	0.2726	0.1254	-
PBANKA_050130	DNA polymerase delta catalytic subunit, putative	Expressed in male and female gametocytes	0.1360	0.0570	-
PBANKA_141510	cholinephosphate cytidylyltransferase, putative	Expressed in male and female gametocytes	0.1585	0.1057	-
PBANKA_091900	26S proteasome regulatory complex subunit, putative	Expressed in male and female gametocytes	0.1597	0.0661	-
PBANKA_113190	serine/threonine protein phosphatase, putative	Expressed in male and female gametocytes	0.2066	0.1915	-
PBANKA_060990	heat shock protein DNAJ homologue Pfj4, putative	Expressed in male and female gametocytes	0.1707	0.1085	-
PBANKA_142240	importin beta, putative	Expressed in male and female gametocytes	0.1641	0.0215	-

PBANKA_134040	oxidoreductase, putative	Expressed in male and female gametocytes	0.1149	0.0690	-
PBANKA_135410	Rab GTPase 11b	Expressed in male and female gametocytes	0.2275	0.0665	-
PBANKA_135960	transmission blocking target antigen precursor	Expressed in male and female gametocytes	0.0825	0.3848	+
PBANKA_145830	kinesin-like protein	Expressed in male and female gametocytes	0.1846	0.1433	-
PBANKA_135500	vacuolar ATP synthase subunit d, putative	Expressed in male and female gametocytes	0.1007	0.1341	-
PBANKA_101320	ATP-specific succinyl-CoA synthetase beta subunit, putative	Expressed in female gametocytes and asexual blood stag	0.1018	0.0454	-
PBANKA_031240	DNA-directed RNA polymerase II second largest subunit, putative	Expressed in female gametocytes and asexual blood stag	0.1825	0.0300	-
PBANKA_120190	40S ribosomal protein S20e, putative	Expressed in female gametocytes and asexual blood stag	0.1003	0.0000	-
PBANKA_110140		Expressed in female gametocytes and asexual blood stag	0.1115	1..596	+
PBANKA_111630	malate quinone oxidoreductase, putative	Expressed in female gametocytes and asexual blood stag	0.2080	0.0977	-
PBANKA_082350	glutamine synthetase, putative	Expressed in female gametocytes and asexual blood stag	0.2549	0.0895	-
PBANKA_082310	S-adenosylmethionine synthetase, putative	Expressed in female gametocytes and asexual blood stag	0.1691	0.0000	-
PBANKA_112780	DnaJ protein, putative	Expressed in female gametocytes and asexual blood stag	0.1774	0.1118	-
PBANKA_103180	conserved Plasmodium protein, unknown function	Expressed in female gametocytes and asexual blood stag	0.1232	0.1679	+
PBANKA_110420	3-methyl-2-oxobutanoate dehydrogenase (lipoamide), putative	Expressed in female gametocytes and asexual blood stag	0.0974	0.1451	-
PBANKA_132630	cytochrome c1 precursor, putative	Expressed in female gametocytes and asexual blood stag	0.0851	0.0000	+
PBANKA_130220	translocation protein sec62, putative	Expressed in female gametocytes and asexual blood stag	0.1153	0.1389	+
PBANKA_132100	conserved Plasmodium protein, unknown function	Expressed in female gametocytes and asexual blood stag	0.1003	0.1046	-
PBANKA_030910	peptide chain release factor subunit 1 putative	Expressed in female gametocytes and asexual blood stag	0.4629	0.0023	-
PBANKA_111140	myo-inositol 1-phosphate synthase, putative	Expressed in female gametocytes and asexual blood stag	0.3807	0.0469	-
PBANKA_144680	chaperonin CPN60, mitochondrial precursor, putative	Expressed in female gametocytes and asexual blood stag	0.1322	0.0261	+
PBANKA_110340	60S ribosomal protein L31, putative	Expressed in female gametocytes and asexual blood stag	0.2254	0.0364	-
PBANKA_061890	conserved Plasmodium protein, unknown function	Expressed in female gametocytes and asexual blood stag	0.1585	0.1167	+
PBANKA_010960	conserved Plasmodium protein, unknown function	Expressed in female gametocytes and asexual blood stag	0.0929	0.1405	-
PBANKA_114360	alanine - tRNA ligase, putative	Expressed in female gametocytes and asexual blood stag	0.1008	0.2037	-
PBANKA_130330	ubiquinol-cytochrome c reductase, iron-sulfur subunit, putative	Expressed in female gametocytes and asexual blood stag	0.1042	0.0390	-
PBANKA_112550	conserved Plasmodium protein, unknown function	Expressed in female gametocytes and asexual blood stag	0.1096	0.0350	-
PBANKA_142060	histone H2B, putative	Expressed in female gametocytes and asexual blood stag	0.0848	0.0000	-
PBANKA_134330	proteasome subunit beta type 7 precursor, putative	Expressed in female gametocytes and asexual blood stag	0.0558	0.0487	-
PBANKA_103960	proteosome subunit, putative	Expressed in female gametocytes and asexual blood stag	0.0826	0.0834	-
PBANKA_121090	phosphoglucomutase, putative	Expressed in female gametocytes and asexual blood stag	0.1181	0.1882	-
PBANKA_030430	adenylosuccinate lyase, putative	Expressed in female gametocytes and asexual blood stag	0.1903	0.1621	-
PBANKA_102980	N-myristoyltransferase, putative	Expressed in female gametocytes and asexual blood stag	0.0738	0.1037	-
PBANKA_010560	translation initiation factor IF-2, putative	Expressed in female gametocytes and asexual blood stag	0.2145	0.0716	-

PBANKA_111650	mitochondrial import receptor subunit, putative	Expressed in female gametocytes and asexual blood stag	0.2211	0.0885	-
PBANKA_041700	signal peptidase, putative	Expressed in female gametocytes and asexual blood stag	0.1398	0.0554	+
PBANKA_112070	nascent polypeptide associated complex alpha chain, putative	Expressed in female gametocytes and asexual blood stag	0.1865	0.0899	-
PBANKA_134000	dihydrofolate synthase/folylpolyglutamate synthase, putative	Expressed in female gametocytes and asexual blood stag	0.1187	0.1539	-
PBANKA_141000	26S proteasome regulatory subunit 7 putative	Expressed in female gametocytes and asexual blood stag	0.1016	0.0103	-
PBANKA_070150	prohibitin, putative	Expressed in female gametocytes and asexual blood stag	0.1101	0.0000	-
PBANKA_140980	ATP synthase gamma chain, mitochondrial precursor, putative	Expressed in female gametocytes and asexual blood stag	0.0799	0.0590	-
PBANKA_071570	lysine decarboxylase-like protein, putative	Expressed in female gametocytes and asexual blood stag	0.0810	0.3828	+
PBANKA_092490	translation elongation factor EF-1, subunit alpha, putative	Expressed in female gametocytes and asexual blood stag	0.1806	0.0281	-
PBANKA_130970	helicase, putative	Expressed in female gametocytes and asexual blood stag	0.0990	0.0755	-
PBANKA_135900	sec61 alpha subunit, putative	Expressed in female gametocytes and asexual blood stag	0.0743	0.0210	+
PBANKA_092280	small nuclear ribonucleoprotein D1, putative	Expressed in female gametocytes and asexual blood stag	0.1911	0.0000	-
PBANKA_093240	berghepain-2	Expressed in female gametocytes and asexual blood stag	0.0818	0.6002	+
PBANKA_083440	organelle processing peptidase, putative	Expressed in female gametocytes and asexual blood stag	0.1837	0.0890	-
PBANKA_122030	lysophospholipase, putative	Expressed in female gametocytes and asexual blood stag	0.2302	0.2057	-
PBANKA_111930	conserved Plasmodium protein, unknown function	Expressed in female gametocytes and asexual blood stag	0.0608	0.2908	-
PBANKA_114400	proteasome regulatory subunit, putative	Expressed in female gametocytes and asexual blood stag	0.1208	0.0125	-
PBANKA_122800	1-cys peroxiredoxin, putative	Expressed in female gametocytes and asexual blood stag	0.0628	0.4222	-
PBANKA_020920	parasite-infected erythrocyte surface protein	Expressed in female gametocytes and asexual blood stag	0.3696	0.1129	+
PBANKA_122120	60S ribosomal protein L34a, putative	Expressed in female gametocytes and asexual blood stag	0.1436	0.0236	-
PBANKA_091370	coatomer delta subunit, putative	Expressed in female gametocytes and asexual blood stag	0.0871	0.0326	-
PBANKA_061790	ferrodoxin reductase-like protein, putative	Expressed in female gametocytes and asexual blood stag	0.3011	0.1191	-
PBANKA_141110	branched-chain alpha keto-acid dehydrogenase, putative	Expressed in female gametocytes and asexual blood stag	0.0995	0.1370	-
PBANKA_111530	metabolite/drug transporter, putative	Expressed in female gametocytes and asexual blood stag	0.1209	0.0777	+
PBANKA_070680	DnaJ protein, putative	Expressed in female gametocytes and asexual blood stag	0.1212	0.1490	+
PBANKA_051700	early transcribed membrane protein	Expressed in female gametocytes and asexual blood stag	0.1364	1..410	+
PBANKA_092320	conserved Plasmodium protein, unknown function	Expressed in female gametocytes and asexual blood stag	0.2792	0.0667	+
PBANKA_102230	mannose-1-phosphate guanyltransferase, putative	Expressed in female gametocytes and asexual blood stag	0.5003	0.1003	-
PBANKA_081340	conserved Plasmodium protein, unknown function	Expressed in female gametocytes and asexual blood stag	0.2420	0.1214	+
PBANKA_040230	dihydrolipoamide acyltransferase, putative	Expressed in female gametocytes and asexual blood stag	0.3673	0.0727	-
PBANKA_050670	citrate synthase, mitochondrial precursor, putative	Expressed in female gametocytes and asexual blood stag	0.1108	0.1931	-
PBANKA_083300	conserved Plasmodium protein, unknown function	Expressed in female gametocytes and asexual blood stag	0.1228	0.1981	-
PBANKA_110300	Ser/Arg-rich splicing factor, putative	Expressed in female gametocytes and asexual blood stag	0.2125	0.0568	-
PBANKA_090390	coat protein, gamma subunit, putative	Expressed in female gametocytes and asexual blood stag	0.4606	0.1041	-

PBANKA_131550	sortilin, putative	Expressed in female gametocytes and asexual blood stag	0.1016	0.1336	+
PBANKA_071010	2-oxoglutarate dehydrogenase e1, putative	Expressed in female gametocytes and asexual blood stag	0.2061	0.0329	-
PBANKA_133830	60S ribosomal protein L6, putative	Expressed in female gametocytes and asexual blood stag	0.1561	0.0535	-
PBANKA_142430	conserved Plasmodium protein, unknown function	Expressed in female gametocytes and asexual blood stag	0.1020	0.0897	-
PBANKA_111230	Rab1b, putative	Expressed in female gametocytes and asexual blood stag	0.1625	0.0198	+
PBANKA_122660	tubulin gamma chain, putative	Expressed in male gametocytes and asexual blood stages	0.2871	0.0033	-
PBANKA_071590	ubiquitin carboxyl-terminal hydrolase a, putative	Expressed in male gametocytes and asexual blood stages	0.1433	0.1339	-
PBANKA_131740	p23 co-chaperone, putative	Expressed in male gametocytes and asexual blood stages	0.1222	0.0678	+
PBANKA_052090	antigen UB05, putative	Expressed in male gametocytes and asexual blood stages	0.0733	0.1290	+
PBANKA_132680	replication factor C3, putative	Expressed in male gametocytes and asexual blood stages	0.0787	0.0557	-
PBANKA_132050	V-type H(+)-translocating pyrophosphatase, putative	Expressed in male gametocytes and asexual blood stages	0.0990	0.2030	+
PBANKA_101060	calmodulin, putative	Expressed in male gametocytes and asexual blood stages	0.1345	0.0000	-
PBANKA_093650	replication factor C subunit 5 putative	Expressed in male gametocytes and asexual blood stages	0.1926	0.0957	-
PBANKA_090450	ubiquitin activating enzyme (E1) subunit Aos1, putative	Expressed in male gametocytes and asexual blood stages	0.1377	0.0990	-
PBANKA_123220	transcriptional regulator, putative	Expressed in male gametocytes and asexual blood stages	0.1655	0.0550	-
PBANKA_082030	protein disulfide isomerase, putative	Expressed in male gametocytes and asexual blood stages	0.1135	0.2550	+
PBANKA_050450	cytoplasmic dynein intermediate chain, putative	Expressed in male gametocytes and asexual blood stages	0.1970	0.1219	-
PBANKA_081350	conserved Plasmodium protein, unknown function	Expressed in male gametocytes and asexual blood stages	0.1509	0.1991	-
PBANKA_120900	eukaryotic translation initiation factor 2 beta, putative	Expressed in male gametocytes and asexual blood stages	0.1031	0.0826	-
PBANKA_133640	DnaJ protein, putative	Expressed in male gametocytes and asexual blood stages	0.1237	0.1909	-
PBANKA_093120	heat shock protein 101 putative	Expressed in male gametocytes and asexual blood stages	0.1442	0.0694	+
PBANKA_101140	DNA topoisomerase II, putative	Expressed in male gametocytes and asexual blood stages	0.1178	0.0865	-
PBANKA_081300	falstatin, putative	Expressed in male gametocytes and asexual blood stages	0.1055	0.6308	-
PBANKA_051450	26S proteasome subunit, putative	Expressed in male gametocytes and asexual blood stages	0.2592	0.1078	-
PBANKA_081200	DNA primase, large subunit, putative	Expressed in male gametocytes and asexual blood stages	0.0846	0.1253	-
PBANKA_103060	conserved Plasmodium protein, unknown function	Expressed in male gametocytes and asexual blood stages	0.2143	0.1814	+
PBANKA_070770	conserved Plasmodium protein, unknown function	Expressed in male gametocytes and asexual blood stages	0.1873	0.2962	-
PBANKA_072030	proteasome regulatory component, putative	Expressed in male gametocytes and asexual blood stages	0.2546	0.1135	-
PBANKA_123920	cytosolic preribosomal GTP-binding protein, putative	Expressed in male gametocytes and asexual blood stages	0.2008	0.0000	-
PBANKA_131040	centrin, putative	Expressed in male gametocytes and asexual blood stages	0.1334	0.0210	-
PBANKA_041600	RhopH3, putative	Expressed in male gametocytes and asexual blood stages	0.2085	0.4057	-
PBANKA_091210	serine/threonine protein kinase, putative	Expressed in male gametocytes and asexual blood stages	0.0848	0.0000	-
PBANKA_145200	conserved Plasmodium protein, unknown function	Expressed in male gametocytes and asexual blood stages	0.2538	0.1119	-
PBANKA_110220	merozoite surface protein 8	Expressed in male gametocytes and asexual blood stages	0.2531	0.2987	+

PBANKA_120540	rhoGAP GTPase, putative	Expressed in male gametocytes and asexual blood stage:	0.1456	0.1570	+
PBANKA_083310	aspartyl aminopeptidase, putative	Expressed in male gametocytes and asexual blood stage:	0.2380	0.0790	-
PBANKA_113820	ATP-dependent DNA helicase, putative	Expressed in male gametocytes and asexual blood stage:	0.1446	0.0495	-
PBANKA_131810	peptidase, putative	Expressed in male gametocytes and asexual blood stage:	0.0911	0.3050	+
PBANKA_122580	peptidyl-prolyl cis-trans isomerase precursor, putative	Expressed in male gametocytes and asexual blood stage:	0.0878	0.1137	-
PBANKA_094290	phenylalanyl-tRNA synthetase beta chain, putative	Expressed in male gametocytes and asexual blood stage:	0.2433	0.1327	-
PBANKA_010100	MYND finger protein, putative	Expressed in male gametocytes and asexual blood stage:	0.0937	0.0568	-
PBANKA_143190	glycerol-3-phosphate dehydrogenase, putative	Expressed in male gametocytes and asexual blood stage:	0.0693	0.1500	-
PBANKA_110310	actin depolymerizing factor, putative	Expressed in male gametocytes and asexual blood stage:	0.4003	0.0394	-
PBANKA_051990	conserved Plasmodium protein, unknown function	Expressed in male gametocytes and asexual blood stage:	0.1046	0.2752	-
PBANKA_123780	multidrug resistance protein, putative	Expressed in male gametocytes and asexual blood stage:	0.1117	0.2464	+
PBANKA_031600	replication factor C subunit 1 putative	Expressed in male gametocytes and asexual blood stage:	0.2631	0.0728	-
PBANKA_081930	eukaryotic translation initiation factor 3 subunit 5 putative	Expressed in all 3 stages	0.2272	0.0724	-
PBANKA_121430	enolase, putative	Expressed in all 3 stages	0.1212	0.0165	-
PBANKA_091430	protein disulfide isomerase related protein, putative	Expressed in all 3 stages	0.1131	0.0740	+
PBANKA_092210	40S ribosomal protein S18, putative	Expressed in all 3 stages	0.1109	0.0000	-
PBANKA_041280	translation initiation factor E4, putative	Expressed in all 3 stages	0.1507	0.0000	-
PBANKA_020650	Rab5c, GTPase, putative	Expressed in all 3 stages	0.1706	0.0478	-
PBANKA_091230	EBNA2 binding protein P100 homologue, putative	Expressed in all 3 stages	0.0627	0.2491	-
PBANKA_123180	60S ribosomal protein L12, putative	Expressed in all 3 stages	0.2116	0.0135	+
PBANKA_090410	Rab6	Expressed in all 3 stages	0.2261	0.0226	-
PBANKA_051360	adenosine deaminase, putative	Expressed in all 3 stages	0.3278	0.1305	-
PBANKA_040470	glutaredoxin 1 putative	Expressed in all 3 stages	0.0506	0.2640	-
PBANKA_091020	protein phosphatase 2C, putative	Expressed in all 3 stages	0.1015	0.2190	-
PBANKA_051720	haloacid dehalogenase-like hydrolase, putative	Expressed in all 3 stages	0.1184	0.2096	-
PBANKA_050510	ADP-ribosylation factor, putative	Expressed in all 3 stages	0.2617	0.0000	-
PBANKA_103440	plasmepsin IV	Expressed in all 3 stages	0.1046	0.6385	+
PBANKA_136430	glycerol kinase, putative	Expressed in all 3 stages	0.1265	0.0446	-
PBANKA_080910	subunit of proteaseome activator complex, putative	Expressed in all 3 stages	0.1667	0.0597	-
PBANKA_121650	peptidyl-prolyl cis-trans isomerase, putative	Expressed in all 3 stages	0.1096	0.0395	-
PBANKA_101310	60S ribosomal protein L14, putative	Expressed in all 3 stages	0.3617	0.0914	-
PBANKA_061910	40S ribosomal protein S5, putative	Expressed in all 3 stages	0.1184	0.0000	-
PBANKA_091800	60S ribosomal protein, putative	Expressed in all 3 stages	0.0757	0.0599	-
PBANKA_135180	proteasome regulatory component, putative	Expressed in all 3 stages	0.4285	0.0886	-

PBANKA_121560	40S ribosomal protein S3A, putative	Expressed in all 3 stages	0.2102	0.0000	-
PBANKA_102660	GTP-binding protein, putative	Expressed in all 3 stages	0.1968	0.1567	-
PBANKA_121820	T-complex protein 1 epsilon subunit, putative	Expressed in all 3 stages	0.0738	0.0361	-
PBANKA_121020	QF122 antigen, putative	Expressed in all 3 stages	0.0914	0.1478	-
PBANKA_133190	helicase 45 putative	Expressed in all 3 stages	0.0854	0.0000	-
PBANKA_145250	conserved Plasmodium protein, unknown function	Expressed in all 3 stages	0.2453	0.1016	+
PBANKA_113110	adenylosuccinate synthetase, putative	Expressed in all 3 stages	0.1752	0.0862	-
PBANKA_121400	heat shock protein 60 putative	Expressed in all 3 stages	0.1886	0.0203	-
PBANKA_090360	dynamin-like protein, putative	Expressed in all 3 stages	0.3001	0.0585	-
PBANKA_121080	hypoxanthine phosphoribosyltransferase, putative	Expressed in all 3 stages	0.1421	0.1716	-
PBANKA_041030	26S proteasome regulatory subunit S14, putative	Expressed in all 3 stages	0.2565	0.1053	-
PBANKA_122900	hypothetical protein	Expressed in all 3 stages	0.1810	0.9360	-
PBANKA_120980	20S proteasome beta subunit, putative	Expressed in all 3 stages	0.1535	0.0518	-
PBANKA_010390	RNA-binding protein, putative	Expressed in all 3 stages	0.1070	0.1898	-
PBANKA_144400	macrophage migration inhibitory factor	Expressed in all 3 stages	0.2736	0.0462	-
PBANKA_071560	26S proteasome AAA-ATPase subunit RPT3, putative	Expressed in all 3 stages	0.1104	0.0000	-
PBANKA_133990	SNARE protein, putative	Expressed in all 3 stages	0.2271	0.0306	-
PBANKA_120240	60S ribosomal protein L13, putative	Expressed in all 3 stages	0.0687	0.0519	-
PBANKA_133430	exported protein 2 putative	Expressed in all 3 stages	0.1291	0.2140	+
PBANKA_132810	ATP synthase (C/AC39) subunit, putative	Expressed in all 3 stages	0.1335	0.0000	-
PBANKA_060480	eukaryotic translation initiation factor 3 subunit 8 putative	Expressed in all 3 stages	0.1328	0.0851	-
PBANKA_092000	multiprotein bridging factor type 1 putative	Expressed in all 3 stages	0.1194	0.0453	-
PBANKA_143420	arginyl-tRNA synthetase, putative	Expressed in all 3 stages	0.1008	0.1102	-
PBANKA_052280	40S ribosomal protein S19, putative	Expressed in all 3 stages	0.3992	0.0105	-
PBANKA_102620	NADP-specific glutamate dehydrogenase, putative	Expressed in all 3 stages	0.1494	0.0597	-
PBANKA_103270	RNA-binding protein, putative	Expressed in all 3 stages	0.0991	0.1283	-
PBANKA_061520	calcium-dependent protein kinase 4	Expressed in all 3 stages	0.1157	0.0210	-
PBANKA_092440	high mobility group-like protein NHP2, putative	Expressed in all 3 stages	0.1696	0.2502	-
PBANKA_113510	40S ribosomal protein S13, putative	Expressed in all 3 stages	0.0400	0.0000	-
PBANKA_093130	dipeptidyl aminopeptidase, putative	Expressed in all 3 stages	0.1374	0.2840	+
PBANKA_102200	40S ribosomal protein S25, putative	Expressed in all 3 stages	0.2664	0.0000	+
PBANKA_103030	nuclear transport factor 2 putative	Expressed in all 3 stages	0.0601	0.1212	-
PBANKA_101050	Hsp70/Hsp90 organizing protein, putative	Expressed in all 3 stages	0.0892	0.0415	-
PBANKA_094250	conserved Plasmodium protein, unknown function	Expressed in all 3 stages	0.2260	0.0427	+

PBANKA_122650	proteasome beta-subunit, putative	Expressed in all 3 stages	0.1543	0.0396	-
PBANKA_145930	actin, putative	Expressed in all 3 stages	0.2530	0.0000	-
PBANKA_070390	receptor for activated C kinase, putative	Expressed in all 3 stages	0.0887	0.0000	-
PBANKA_040530	40S ribosomal protein S23, putative	Expressed in all 3 stages	0.0907	0.0000	-
PBANKA_145030	ATP synthase beta chain, mitochondrial precursor, putative	Expressed in all 3 stages	0.0810	0.1285	-
PBANKA_050580	deoxyribose-phosphate aldolase, putative	Expressed in all 3 stages	0.1796	0.1714	-
PBANKA_020860	conserved Plasmodium protein, unknown function	Expressed in all 3 stages	0.2597	0.1380	-
PBANKA_070800	sec23, putative	Expressed in all 3 stages	0.2477	0.0187	-
PBANKA_112560	pyruvate kinase, putative	Expressed in all 3 stages	0.1046	0.0169	-
PBANKA_121760	histone H2A, putative	Expressed in all 3 stages	0.0822	0.0425	-
PBANKA_070670	oxoglutarate/malate translocator, putative	Expressed in all 3 stages	0.0920	0.0987	+
PBANKA_145610	40S ribosomal protein S17, putative	Expressed in all 3 stages	0.0200	0.2644	-
PBANKA_124230	58 kDa phosphoprotein	Expressed in all 3 stages	0.1726	0.0861	-
PBANKA_091620	TCP-1/cpn60 chaperonin family, putative	Expressed in all 3 stages	0.1270	0.0340	-
PBANKA_136520	thioredoxin-related protein, putative	Expressed in all 3 stages	0.1517	0.2222	+
PBANKA_041460	40S ribosomal protein S15A, putative	Expressed in all 3 stages	0.1875	0.0000	-
PBANKA_122280	tyrosyl-tRNA synthetase, putative	Expressed in all 3 stages	0.3205	0.0743	-
PBANKA_051870	methionine -- tRNA ligase, putative	Expressed in all 3 stages	0.1104	0.2019	-
PBANKA_142610	conserved Plasmodium protein, unknown function	Expressed in all 3 stages	0.1078	0.0930	-
PBANKA_130280	2-Cys peroxiredoxin	Expressed in all 3 stages	0.0539	0.2241	-
PBANKA_090500	40S ribosomal protein S21e, putative	Expressed in all 3 stages	0.1092	0.1315	-
PBANKA_121930	Cg4 protein, putative	Expressed in all 3 stages	0.1349	0.1027	-
PBANKA_143350	conserved Plasmodium protein, unknown function	Expressed in all 3 stages	0.0680	0.3703	+
PBANKA_145510	replication factor C subunit 4 putative	Expressed in all 3 stages	0.0614	0.0446	-
PBANKA_082170	inosine-5'-monophosphate dehydrogenase, putative	Expressed in all 3 stages	0.1776	0.0483	-
PBANKA_010530	conserved Plasmodium protein, unknown function	Expressed in all 3 stages	0.1944	0.3004	-
PBANKA_131750	glucose-6-phosphate dehydrogenase-6- phosphogluconolactonase, putative	Expressed in all 3 stages	0.0687	0.2471	-
PBANKA_040480	FAD-dependent glycerol-3-phosphate dehydrogenase, putative	Expressed in all 3 stages	0.2112	0.0711	+
PBANKA_131080	40S ribosomal protein S2, putative	Expressed in all 3 stages	0.1402	0.0000	-
PBANKA_142420	conserved Plasmodium protein, unknown function	Expressed in all 3 stages	0.1213	0.2001	-
PBANKA_113040	proteasome subunit, putative	Expressed in all 3 stages	0.0876	0.0627	-
PBANKA_142960	bi-functional aminoacyl-tRNA synthetase, putative	Expressed in all 3 stages	0.0492	0.0453	-
PBANKA_081640	6-phosphofructokinase, putative	Expressed in all 3 stages	0.2647	0.0362	-
PBANKA_102790	ribonucleoprotein, putative	Expressed in all 3 stages	0.1133	0.3335	-

PBANKA_080820	proteasome component C8, putative	Expressed in all 3 stages	0.0882	0.1466	-
PBANKA_122420	DnaJ protein, putative	Expressed in all 3 stages	0.2154	0.2696	-
PBANKA_120500	RNA-binding protein, putative	Expressed in all 3 stages	0.3371	0.0664	-
PBANKA_142270	fe-superoxide dismutase, putative	Expressed in all 3 stages	0.2246	0.0157	-
PBANKA_040550	60S ribosomal protein L7, putative	Expressed in all 3 stages	0.2256	0.0388	-
PBANKA_142360	40S ribosomal protein S16, putative	Expressed in all 3 stages	0.1601	0.0000	-
PBANKA_135550	casein kinase II beta chain, putative	Expressed in all 3 stages	0.1619	0.0566	-
PBANKA_082750	NAD synthase, putative	Expressed in all 3 stages	0.2322	0.1115	-
PBANKA_041660	replication factor A-related protein, putative	Expressed in all 3 stages	0.1004	0.1908	+
PBANKA_021150	proteasome subunit alpha type 5 putative	Expressed in all 3 stages	0.2474	0.0254	-
PBANKA_103390	40S ribosomal protein S8e, putative	Expressed in all 3 stages	0.0512	0.0885	-
PBANKA_110670	60S ribosomal protein L4, putative	Expressed in all 3 stages	0.2250	0.0909	-
PBANKA_135440	60S ribosomal protein L18, putative	Expressed in all 3 stages	0.3206	0.0263	-
PBANKA_144070	ubiquitin-activating enzyme e1, putative	Expressed in all 3 stages	0.2503	0.1377	-
PBANKA_040520	T-complex protein beta subunit, putative	Expressed in all 3 stages	0.2262	0.0498	-
PBANKA_143800	CAMP-dependent protein kinase regulatory subunit, putative	Expressed in all 3 stages	0.0905	0.4211	-
PBANKA_132090	thioredoxin, putative	Expressed in all 3 stages	0.0849	0.0886	-
PBANKA_140670	carbamoyl phosphate synthetase, putative	Expressed in all 3 stages	0.0982	0.1979	-
PBANKA_132640	glyceraldehyde-3-phosphate dehydrogenase, putative	Expressed in all 3 stages	0.0677	0.0596	-
PBANKA_114530	conserved Plasmodium protein, unknown function	Expressed in all 3 stages	0.0674	0.5087	+
PBANKA_083260	proteasome precursor, putative	Expressed in all 3 stages	0.1121	0.0701	-
PBANKA_123170	60S ribosomal protein L8, putative	Expressed in all 3 stages	0.1680	0.0437	-
PBANKA_120690	tubulin beta chain, putative	Expressed in all 3 stages	0.1059	0.0123	-
PBANKA_144580	small GTPase Rab2, putative	Expressed in all 3 stages	0.0649	0.0407	-
PBANKA_070280	protein disulfide isomerase	Expressed in all 3 stages	0.1086	0.1643	-
PBANKA_030360	proteasome 26S regulatory subunit, putative	Expressed in all 3 stages	0.1924	0.0976	-
PBANKA_143920	polyadenylate-binding protein, putative	Expressed in all 3 stages	0.2160	0.1220	-
PBANKA_082340	phosphoglycerate kinase, putative	Expressed in all 3 stages	0.1952	0.0443	-
PBANKA_136030	conserved Plasmodium protein, unknown function	Expressed in all 3 stages	0.0939	0.0502	-
PBANKA_143410	conserved Plasmodium protein, unknown function	Expressed in all 3 stages	0.0962	0.0783	-
PBANKA_041770	alpha tubulin 1	Expressed in all 3 stages	0.1047	0.0000	-
PBANKA_113050	proteasome subunit, putative	Expressed in all 3 stages	0.3717	0.0053	-
PBANKA_145260	calcyclin binding protein, putative	Expressed in all 3 stages	0.1834	0.1035	+
PBANKA_081990	nucleosome assembly protein	Expressed in all 3 stages	0.2955	0.0550	-

PBANKA_146180	tat-binding protein homolog, putative	Expressed in all 3 stages	0.2188	0.0310	-
PBANKA_031380	ATP synthase F1, alpha subunit, putative	Expressed in all 3 stages	0.1526	0.0606	-
PBANKA_122310	proteasome subunit alpha, putative	Expressed in all 3 stages	0.1901	0.0175	+
PBANKA_123990	acyl-CoA synthetase, putative	Expressed in all 3 stages	0.2177	0.1652	-
PBANKA_136420	60S ribosomal protein L17, putative	Expressed in all 3 stages	0.1092	0.0439	-
PBANKA_130990	leucine aminopeptidase, putative	Expressed in all 3 stages	0.2469	0.1189	-
PBANKA_092230	threonine -- tRNA ligase, putative	Expressed in all 3 stages	0.2556	0.0765	-
PBANKA_071930	bifunctional dihydrofolate reductase-thymidylate synthase, putative	Expressed in all 3 stages	0.2299	0.1618	-
PBANKA_145620	rabGDI protein, putative	Expressed in all 3 stages	0.0571	0.0522	-
PBANKA_111860	cell division cycle protein 48 homologue, putative	Expressed in all 3 stages	0.1663	0.0106	-
PBANKA_103190	eukaryotic translation initiation factor 2 gamma subunit, putative	Expressed in all 3 stages	0.1089	0.0143	-
PBANKA_050220	26s proteasome subunit p55, putative	Expressed in all 3 stages	0.1365	0.0539	-
PBANKA_091790	26S protease subunit regulatory subunit 6a, putative	Expressed in all 3 stages	0.3000	0.0191	-
PBANKA_080570	heat shock protein 90 putative	Expressed in all 3 stages	0.1056	0.0338	-
PBANKA_130620	tRNA binding protein, putative	Expressed in all 3 stages	0.1171	0.2155	-
PBANKA_121360	cysteinyl-tRNA synthetase, putative	Expressed in all 3 stages	0.2014	0.0678	-
PBANKA_010910	transketolase, putative	Expressed in all 3 stages	0.1613	0.0629	-
PBANKA_093840	endoplasmic reticulum-resident calcium binding protein, putative	Expressed in all 3 stages	0.1442	0.0427	+
PBANKA_123420	40S ribosomal protein S24, putative	Expressed in all 3 stages	0.2945	0.0120	-
PBANKA_102830	serine/threonine protein phosphatase, putative	Expressed in all 3 stages	0.2153	0.0000	-
PBANKA_112620	4-methyl-5(B-hydroxyethyl)-thiazol monophosphate biosynthesis enzyme, putative	Expressed in all 3 stages	0.0867	0.1207	-
PBANKA_021020	aspartyl-tRNA synthetase, putative	Expressed in all 3 stages	0.1803	0.0958	-
PBANKA_093090	deubiquinating/deneddylating enzyme, putative	Expressed in all 3 stages	0.3269	0.1840	-
PBANKA_091910	conserved Plasmodium protein, unknown function	Expressed in all 3 stages	0.1035	1..293	-
PBANKA_101860	60S ribosomal protein L21e, putative	Expressed in all 3 stages	0.1766	0.0905	-
PBANKA_144690	dihydrolipoamide dehydrogenase, putative	Expressed in all 3 stages	0.2582	0.0638	-
PBANKA_132970	26S proteasome subunit, putative	Expressed in all 3 stages	0.2223	0.1003	-
PBANKA_135190	60S ribosomal protein L6-2, putative	Expressed in all 3 stages	0.2117	0.3200	-
PBANKA_145230	conserved Plasmodium protein, unknown function	Expressed in all 3 stages	0.1520	0.2656	+
PBANKA_082670	conserved Plasmodium protein, unknown function	Expressed in all 3 stages	0.3945	0.0340	-
PBANKA_130860	fructose-bisphosphate aldolase 2	Expressed in all 3 stages	0.0729	0.0288	-
PBANKA_141040	vacuolar ATP synthase subunit a, putative	Expressed in all 3 stages	0.0919	0.0000	-
PBANKA_123480	40S ribosomal protein S9, putative	Expressed in all 3 stages	0.2701	0.0209	-
PBANKA_050360	60S ribosomal protein L30e, putative	Expressed in all 3 stages	0.1083	0.0434	-

PBANKA_041820	Rab7, putative	Expressed in all 3 stages	0.1608	0.0000	-
PBANKA_060190	high mobility group protein, putative	Expressed in all 3 stages	0.1941	0.0451	-
PBANKA_083020	rhoptry protein-2, putative	Expressed in all 3 stages	0.2851	0.2144	-
PBANKA_134030	phosphoribosylpyrophosphate synthetase, putative	Expressed in all 3 stages	0.1418	0.0352	-
PBANKA_111750	ribosomal protein L27a, putative	Expressed in all 3 stages	0.2158	0.0583	-
PBANKA_040540	40S ribosomal protein S12, putative	Expressed in all 3 stages	0.2559	0.0516	-
PBANKA_131840	6-phosphogluconate dehydrogenase, decarboxylating, putative	Expressed in all 3 stages	0.0814	0.0756	-
PBANKA_031090	T-complex protein 1 putative	Expressed in all 3 stages	0.3261	0.0937	-
PBANKA_114240	nucleoside diphosphate kinase b, putative	Expressed in all 3 stages	0.2816	0.0331	-
PBANKA_121420	ribonucleotide reductase small subunit, putative	Expressed in all 3 stages	0.1842	0.1437	-
PBANKA_135200	elongation factor 1-gamma, putative	Expressed in all 3 stages	0.2255	0.1348	-
PBANKA_051090	40S ribosomal protein S2B, putative	Expressed in all 3 stages	0.1236	0.0275	-
PBANKA_135510	40S ribosomal protein S6, putative	Expressed in all 3 stages	0.1956	0.0999	-
PBANKA_140760	60S ribosomal protein L24, putative	Expressed in all 3 stages	0.1541	0.0000	-
PBANKA_145330	acyl-CoA synthetase, putative	Expressed in all 3 stages	0.1589	0.1659	-
PBANKA_040650	TCP-1/cpn60 chaperonin, putative	Expressed in all 3 stages	0.2584	0.0210	-
PBANKA_081130	ran-binding protein, putative	Expressed in all 3 stages	0.0856	0.0298	-
PBANKA_031470	replication factor C, subunit 2 putative	Expressed in all 3 stages	0.2160	0.0656	-
PBANKA_102300	conserved Plasmodium protein, unknown function	Expressed in all 3 stages	0.1240	0.1609	-
PBANKA_010550	glutaredoxin-like protein, putative	Expressed in all 3 stages	0.1373	0.1156	-
PBANKA_111770	malate dehydrogenase, putative	Expressed in all 3 stages	0.1458	0.1446	-
PBANKA_091930	spermidine synthase, putative	Expressed in all 3 stages	0.1929	0.0925	+
PBANKA_101840	arginine methyltransferase 1 putative	Expressed in all 3 stages	0.2486	0.0777	-
PBANKA_061040	ATPase, putative	Expressed in all 3 stages	0.1838	0.1375	+
PBANKA_134010	L-lactate dehydrogenase	Expressed in all 3 stages	0.0987	0.0230	+
PBANKA_010740	ornithine aminotransferase, putative	Expressed in all 3 stages	0.2018	0.0448	-
PBANKA_142690	RNA helicase, putative	Expressed in all 3 stages	0.1179	0.0743	-
PBANKA_031420	calcium-dependent protein kinase 1 putative	Expressed in all 3 stages	0.1833	0.0229	-
PBANKA_030860	GDP-fructose GMP antiporter, putative	Expressed in all 3 stages	0.2784	0.0628	-
PBANKA_131480	elongation factor 2 putative	Expressed in all 3 stages	0.0620	0.0000	-
PBANKA_092130	deoxyuridine 5'-triphosphate nucleotidohydrolase, putative	Expressed in all 3 stages	0.2567	0.0657	-
PBANKA_143730	endoplasmin homolog precursor, putative	Expressed in all 3 stages	0.0856	0.0965	-
PBANKA_132500	40S ribosomal protein S28e, putative	Expressed in all 3 stages	0.0000	-1.0000	-
PBANKA_100880	glucose-6-phosphate isomerase, putative	Expressed in all 3 stages	0.0515	0.2432	-

PBANKA_135920	nucleic acid binding protein, putative	Expressed in all 3 stages	0.1066	0.1094	-
PBANKA_141030	M1-family aminopeptidase, putative	Expressed in all 3 stages	0.0676	0.1811	+
PBANKA_122920	60S ribosomal protein L19, putative	Expressed in all 3 stages	0.0000	-1.0000	-
PBANKA_021210	eukaryotic translation initiation factor 2 alpha subunit, putative	Expressed in all 3 stages	0.1790	0.0377	-
PBANKA_144110	hydrolase, putative	Expressed in all 3 stages	0.3477	0.0143	-
PBANKA_061710	60S ribosomal protein L11a, putative	Expressed in all 3 stages	0.2388	0.0000	-
PBANKA_010710	proteasome subunit alpha type 2 putative	Expressed in all 3 stages	0.2788	0.0172	-
PBANKA_133410	20S proteasome beta 4 subunit, putative	Expressed in all 3 stages	0.0450	0.0498	-
PBANKA_130510	60S ribosomal protein L10a, putative	Expressed in all 3 stages	0.0602	0.0638	-
PBANKA_113330	elongation factor 1 alpha	Expressed in all 3 stages	0.0769	0.0437	-
PBANKA_101080	ABC transporter, putative	Expressed in all 3 stages	0.0888	0.0342	-
PBANKA_030250	hexose transporter, putative	Expressed in all 3 stages	0.2696	0.0895	+
PBANKA_145070	clustered-asparagine-rich protein, putative	Expressed in all 3 stages	0.3373	0.0654	-
PBANKA_124280	eukaryotic translation initiation factor 3 subunit 6 putative	Expressed in all 3 stages	0.2841	0.0344	+
PBANKA_040280	EH (Eps15 homology) protein, putative	Expressed in all 3 stages	0.2533	0.0079	-
PBANKA_091440	heat shock protein hsp70 homologue, putative	Expressed in all 3 stages	0.0787	0.0304	-
PBANKA_141130	conserved Plasmodium protein, unknown function	Expressed in all 3 stages	0.0896	0.2611	-
PBANKA_112640	transportin, putative	Expressed in all 3 stages	0.0900	0.0864	-
PBANKA_130530	structure specific recognition protein, putative	Expressed in all 3 stages	0.0822	0.0521	-
PBANKA_145020	serine hydroxymethyltransferase, putative	Expressed in all 3 stages	0.2301	0.1052	-
PBANKA_040760	asparagine synthetase, putative	Expressed in all 3 stages	0.1477	0.2161	-
PBANKA_142350	60S ribosomal protein L13-2, putative	Expressed in all 3 stages	0.0992	0.0629	-
PBANKA_092990	heat shock protein 90 putative	Expressed in all 3 stages	0.3235	0.0642	-
PBANKA_130140	proteosome subunit alpha type 1 putative	Expressed in all 3 stages	0.1260	0.0719	-
PBANKA_071550	phosphoglycerate mutase, putative	Expressed in all 3 stages	0.2261	0.0423	+
PBANKA_082480	RNA-binding protein, putative	Expressed in all 3 stages	0.2086	0.0263	-
PBANKA_121100	GMP synthetase, putative	Expressed in all 3 stages	0.1025	0.0757	-
PBANKA_071260	14-3-2003 protein, putative	Expressed in all 3 stages	0.0523	0.1062	-
PBANKA_081390	26S proteasome regulatory subunit, putative	Expressed in all 3 stages	0.0912	0.0000	-
PBANKA_111300	purine nucleotide phosphorylase, putative	Expressed in all 3 stages	0.3011	0.0296	-
PBANKA_142510	importin alpha, putative	Expressed in all 3 stages	0.0684	0.0000	-
PBANKA_123560	adenosylhomocysteinase, putative	Expressed in all 3 stages	0.2098	0.0453	-
PBANKA_141630	40S ribosomal protein S15/S19, putative	Expressed in all 3 stages	0.0351	0.0000	-
PBANKA_071190	heat shock protein, putative	Expressed in all 3 stages	0.2623	0.0000	-

PBANKA_101280	conserved Plasmodium protein, unknown function	Expressed in all 3 stages	0.1269	0.0460	-
PBANKA_091220	conserved Plasmodium protein, unknown function	Expressed in all 3 stages	0.1050	0.2747	-
PBANKA_142870	eukaryotic translation initiation factor 3 subunit 10 putative	Expressed in all 3 stages	0.0812	0.0740	-
PBANKA_061990	GTP-binding protein, putative	Expressed in all 3 stages	0.2734	0.0295	-
PBANKA_101950	60S ribosomal protein L5, putative	Expressed in all 3 stages	0.3652	0.0620	+
PBANKA_103660	ribonucleotide reductase small subunit, putative	Expressed in all 3 stages	0.2002	0.0277	-
PBANKA_113500	conserved Plasmodium protein, unknown function	Expressed in all 3 stages	0.1365	0.0554	-
PBANKA_070740	RNA-binding protein, putative	Expressed in all 3 stages	0.2721	0.0419	-
PBANKA_120610	eukaryotic translation initiation factor 3 subunit 7 putative	Expressed in all 3 stages	0.1887	0.0201	-
PBANKA_143080	thioredoxin peroxidase 2 putative	Expressed in all 3 stages	0.1102	0.1272	-
PBANKA_122840	mannose-6-phosphate isomerase, putative	Expressed in all 3 stages	0.1737	0.2030	-
PBANKA_123250	eukaryotic translation initiation factor 3 subunit, putative	Expressed in all 3 stages	0.2048	0.0191	-
PBANKA_123310	proteasome subunit beta type 1 putative	Expressed in all 3 stages	0.3037	0.1018	-
PBANKA_061090	HSP40, subfamily A, putative	Expressed in all 3 stages	0.2936	0.0404	-
PBANKA_102340	glutathione reductase, putative	Expressed in all 3 stages	0.1556	0.0720	-
PBANKA_144510	ATP-dependent protease hslV, putative	Expressed in all 3 stages	0.1381	0.0225	-
PBANKA_040160	N-ethylmaleimide-sensitive fusion protein, putative	Expressed in all 3 stages	0.2438	0.0577	-
PBANKA_070910	60S ribosomal protein L22, putative	Expressed in all 3 stages	0.1839	0.0759	-
PBANKA_146040	peptidyl-propyl isomerase, putative	Expressed in all 3 stages	0.1871	0.0661	-
PBANKA_072170	ran binding protein 1 putative	Expressed in all 3 stages	0.2239	0.0229	-
PBANKA_094090	RuvB DNA helicase, putative	Expressed in all 3 stages	0.2181	0.0698	-
PBANKA_120660	26S proteasome regulatory subunit 4 putative	Expressed in all 3 stages	0.0710	0.0722	-
PBANKA_140060	cytoadherence linked asexual protein, putative	Expressed in all 3 stages	0.1822	0.3576	+
PBANKA_122330	GTPase, Rab18, putative	Expressed in all 3 stages	0.2426	0.0000	-
PBANKA_020540	beta3 proteasome subunit, putative	Expressed in all 3 stages	0.3670	0.0127	-
PBANKA_101940	60S ribosomal protein L7-3, putative	Expressed in all 3 stages	0.1838	0.0626	-
PBANKA_010730	chaperone, putative	Expressed in all 3 stages	0.2978	0.0259	-
PBANKA_061450	eukaryotic translation initiation factor subunit, putative	Expressed in all 3 stages	0.1118	0.0373	-
PBANKA_142330	DNA/RNA-binding protein, putative	Expressed in all 3 stages	0.0593	0.0000	-
PBANKA_093030	GTP-binding nuclear protein, putative	Expressed in all 3 stages	0.0635	0.0000	-
PBANKA_113410	TCP-1/cpn60 chaperonin, putative	Expressed in all 3 stages	0.0805	0.0106	-
PBANKA_113700	falcilysin, putative	Expressed in all 3 stages	0.0763	0.0796	-
PBANKA_100380	vacuolar ATP synthase subunit b, putative	Expressed in all 3 stages	0.0876	0.0207	-
PBANKA_081890	heat shock protein 70 putative	Expressed in all 3 stages	0.1353	0.0454	+

PBANKA_136290	lysine -- tRNA ligase, putative	Expressed in all 3 stages	0.2618	0.0814	-
PBANKA_142630	conserved Plasmodium protein, unknown function	Expressed in all 3 stages	0.1157	0.2920	+
PBANKA_091810	60S ribosomal protein L38e, putative	Expressed in all 3 stages	0.1479	0.0000	-
PBANKA_060110	mitochondrial phosphate carrier protein, putative	Expressed in all 3 stages	0.0702	0.1000	-
PBANKA_102840	60S ribosomal protein L10, putative	Expressed in all 3 stages	0.2262	0.0146	-
PBANKA_123100	40S ribosomal protein S14, putative	Expressed in all 3 stages	0.1543	0.0000	-
PBANKA_135520	aconitase, putative	Expressed in all 3 stages	0.1658	0.0944	-
PBANKA_030680	DEAD/DEAH helicase, putative	Expressed in all 3 stages	0.0714	0.0389	-
PBANKA_135860	isocitrate dehydrogenase (NADP), mitochondrial precursor, putative	Expressed in all 3 stages	0.0915	0.2245	-
PBANKA_144140	proliferating cell nuclear antigen 2 putative	Expressed in all 3 stages	0.1724	0.0000	-
PBANKA_060270	nucleosome assembly protein 1 putative	Expressed in all 3 stages	0.3050	0.0172	-
PBANKA_101850	transcription factor 3b, putative	Expressed in all 3 stages	0.2735	0.0771	-
PBANKA_082470	thioredoxin reductase, putative	Expressed in all 3 stages	0.1452	0.1126	-
PBANKA_122290	proteasome subunit alpha type 5 putative	Expressed in all 3 stages	0.4370	0.1115	-
PBANKA_130370	vesicle-associated membrane protein, putative	Expressed in all 3 stages	0.0810	0.0490	-
PBANKA_094150	40S ribosomal protein S4, putative	Expressed in all 3 stages	0.3264	0.0255	-
PBANKA_061540	seryl-tRNA synthetase, putative	Expressed in all 3 stages	0.1642	0.1238	-
PBANKA_060320	eukaryotic initiation factor 5a, putative	Expressed in all 3 stages	0.3213	0.0000	-
PBANKA_111050	histamine-releasing factor, putative	Expressed in all 3 stages	0.0679	0.1242	-
PBANKA_061720	40S ribosomal protein S10, putative	Expressed in all 3 stages	0.1604	0.0224	-
PBANKA_112290	hexokinase, putative	Expressed in all 3 stages	0.2784	0.0131	-
PBANKA_123880	karyopherin beta, putative	Expressed in all 3 stages	0.2443	0.0284	-
PBANKA_112770	conserved Plasmodium protein, unknown function	Expressed in all 3 stages	0.0837	0.1984	-
PBANKA_120720	adenylate kinase, putative	Expressed in all 3 stages	0.1558	0.0298	-
PBANKA_143470	clathrin heavy chain, putative	Expressed in all 3 stages	0.1014	0.0983	-
PBANKA_141890	Rab GTPase 11a	Expressed in all 3 stages	0.0997	0.0000	+
PBANKA_052020	ADP/ATP transporter on adenylate translocase, putative	Expressed in all 3 stages	0.1172	0.0407	+
PBANKA_020700	calcium-transporting ATPase, putative	Expressed in all 3 stages	0.2865	0.0749	+
PBANKA_140130	40S ribosomal protein S7, putative	Expressed in all 3 stages	0.0837	0.0882	-
PBANKA_093220	peptidyl-prolyl cis-trans isomerase, putative	Expressed in all 3 stages	0.1160	0.0634	+
PBANKA_020300	chromatin assembly factor 1 protein WD40 domain, putative	Expressed in all 3 stages	0.1866	0.0678	-
PBANKA_041800	DEAD/DEAH helicase, putative	Expressed in all 3 stages	0.1282	0.0962	-
