# Supplementary material for: Why are male malaria parasites in such a rush? Sex-specific evolution and host–parasite interactions
Source: Evol Med Public Health. 2012 Nov 26;2013(1):3–13. doi: 10.1093/emph/eos003 (PMC4183958; doi:10.1093/emph/eos003)
Supplement: Supplementary Data [file supp_eos003_suppl_data.zip › REECE_Table_S1_PF.pdf]

**Pf/Pr Table A: dn/ds, dn,ds for orthologs of P. berghei male proteins**

| P.berghei gene | P.falciparum ortholog | dn/ds | dn | ds |
|---|---|---|---|---|
| **Orthologs of non-membrane proteins** | | | | |
| PB000182.01.0 | PFC0720w | 0.2332 | 0.0162 | 0.0694 |
| PB000733.03.0 | PF11_0240 | 1.2314 | 0.0982 | 0.0797 |
| PB000781.01.0 | PF11_0318 | 0.0391 | 0.0073 | 0.1878 |
| PB000020.01.0 | PF14_0712 | 1.2782 | 0.0665 | 0.0520 |
| PB000427.02.0 | PF11_0360 | 0.6545 | 0.0913 | 0.1396 |
| PB000009.01.0 | PF11_0413 | 0.0686 | 0.0322 | 0.4699 |
| PB000059.02.0 | PF11_0433 | 0.9517 | 0.0963 | 0.1012 |
| PB001397.02.0 | PFA0535c | 0.2116 | 0.0196 | 0.0926 |
| PB000005.01.0 | PF14_0626 | 0.1586 | 0.0087 | 0.0552 |
| PB000867.02.0 | PFL0150w | 0.1612 | 0.0191 | 0.1188 |
| PB300899.00.0 | PFL0350c | 0.3673 | 0.0217 | 0.0590 |
| PB000558.02.0 | PFL0445w | 0.2112 | 0.0106 | 0.0503 |
| PB000306.03.0 | PF13_0301 | 0.0000 | 0.0000 | 0.0338 |
| PB000171.00.0 | MAL13P1.347 | 0.4974 | 0.0229 | 0.0460 |
| PB000002.03.0 | MAL13P1.336 | 0.2844 | 0.0606 | 0.2132 |
| PB000318.01.0 | MAL13P1.328 | 0.3063 | 0.0095 | 0.0310 |
| PB000293.00.0 | MAL13P1.279 | 0.0354 | 0.0019 | 0.0532 |
| PB001076.00.0 | PF13_0308 | 0.1766 | 0.0083 | 0.0472 |
| PB001187.00.0 | PFB0720c | 0.1187 | 0.0092 | 0.0773 |
| PB001162.01.0 | PFB0400w | 0.2030 | 0.0237 | 0.1166 |
| PB000953.02.0 | MAL13P1.32 | 0.5434 | 0.0662 | 0.1219 |
| PB000726.03.0 | PFL0580w | 0.0602 | 0.0035 | 0.0577 |
| PB000159.02.0 | PFL1445w | 0.4420 | 0.0478 | 0.1081 |
| PB001200.02.0 | PFL0660w | 0.0000 | 0.0000 | 0.0181 |
| PB000554.01.0 | PFL1980c | 0.4787 | 0.0785 | 0.1639 |
| PB000535.02.0 | PFL1180w | 0.0000 | 0.0000 | 0.0198 |
| PB001094.01.0 | PFL0760w | 0.8256 | 0.1173 | 0.1420 |
| PB000619.01.0 | PFL0805w | 4.4615 | 0.2306 | 0.0517 |
| PB000662.00.0 | PFL1730c | 0.3204 | 0.0144 | 0.0451 |
| PB000821.00.0 | PFL2190c | -1.0000 | 0.0352 | 0.0000 |
| PB001114.03.0 | PFC0305w | 0.3085 | 0.0103 | 0.0334 |
| PB000609.01.0 | PFC0135c | 0.0445 | 0.0027 | 0.0614 |
| PB000189.00.0 | PFC0595c | 0.0253 | 0.0021 | 0.0840 |
| PB000747.02.0 | PFC0260w | 0.2746 | 0.0162 | 0.0591 |
| PB104504.00.0 | PFB0095c | 0.4998 | 0.0288 | 0.0577 |
| PB000161.02.0 | PF13_0189 | 0.0482 | 0.0375 | 0.7778 |
| PB001008.01.0 | MAL13P1.69 | 1.3754 | 0.0149 | 0.0109 |
| PB001086.02.0 | MAL13P1.93 | 0.1975 | 0.0123 | 0.0622 |
| PB000269.01.0 | MAL13P1.96 | 0.0000 | 0.0000 | 0.0816 |
| PB000651.02.0 | MAL13P1.123 | 0.3143 | 0.0220 | 0.0701 |
| PB001064.02.0 | MAL13P1.57 | 0.0000 | 0.0000 | 0.0381 |
| PB001267.00.0 | PF14_0352 | 0.0159 | 0.0010 | 0.0633 |
| PB000433.03.0 | PF14_0436 | 0.0713 | 0.0049 | 0.0683 |
| PB000074.01.0 | PF14_0458 | 0.2328 | 0.1030 | 0.4424 |
| PB000094.02.0 | PF14_0419 | 0.9432 | 0.0732 | 0.0776 |
| PB001662.02.0 | PF14_0243 | 0.1355 | 0.0053 | 0.0389 |
| PB000359.00.0 | PF14_0081 | 0.4061 | 0.0565 | 0.1391 |
| PB100371.00.0 | PF14_0030 | -1.0000 | 0.1089 | 0.0000 |
| PB000952.01.0 | PF14_0115 | 0.3175 | 0.0195 | 0.0615 |
| PB001172.00.0 | PF14_0120 | 0.9352 | 0.1032 | 0.1103 |
| PB001067.03.0 | PF11_0056 | 0.1542 | 0.0158 | 0.1027 |
| PB000607.03.0 | PF11_0057 | 0.5324 | 0.0262 | 0.0491 |
| PB001145.01.0 | PF11_0086 | 0.2638 | 0.0264 | 0.1000 |
| PB001116.00.0 | PF10_0128 | -1.0000 | 0.0834 | 0.0000 |

| | | | | |
|---|---|---|---|---|
| PB000408.01.0 | PF10_0292 | 0.2478 | 0.0310 | 0.1252 |
| PB001056.02.0 | PF10_0224 | 0.1822 | 0.0117 | 0.0643 |
| PB001011.00.0 | PF10_0232 | 0.2678 | 0.0167 | 0.0624 |
| PB000469.02.0 | PF10_0066 | 0.0867 | 0.0086 | 0.0991 |
| PB000382.03.0 | PF10_0244 | -1.0000 | 0.0741 | 0.0000 |
| PB301567.00.0 | PF10_0251 | 0.5094 | 0.0453 | 0.0888 |
| PB000739.01.0 | PFD0420c | 0.3494 | 0.0300 | 0.0860 |
| PB000958.00.0 | PFE0415w | 0.2665 | 0.0121 | 0.0455 |
| PB000569.03.0 | PFE0450w | 0.3633 | 0.1587 | 0.4367 |
| PB000365.01.0 | PFE0455w | 0.0916 | 0.0080 | 0.0878 |
| PB000192.02.0 | PFE0465c | 0.4084 | 0.0191 | 0.0467 |
| PB001175.02.0 | PFE0495w | 0.2386 | 0.0135 | 0.0565 |
| PB000914.01.0 | PFE0540w | 0.2001 | 0.0055 | 0.0273 |
| PB301405.00.0 | PFE0595w | 0.2779 | 0.0553 | 0.1988 |
| PB001351.02.0 | PFE1345c | 0.1023 | 0.0096 | 0.0941 |
| PB000305.03.0 | PFD0855c | 0.5656 | 0.0469 | 0.0829 |
| PB000174.03.0 | PFD0905w | -1.0000 | 0.1148 | 0.0000 |
| PB000608.02.0 | PFD0590c | 0.2188 | 0.0717 | 0.3278 |
| PB000794.02.0 | PFD0250c | 0.3823 | 0.0416 | 0.1088 |
| PB000254.03.0 | PFE0155w | -1.0000 | 0.0354 | 0.0000 |
| PB300815.00.0 | PFD0285c | 0.3505 | 0.0278 | 0.0793 |
| PB001273.00.0 | PFF1095w | 0.1205 | 0.0227 | 0.1883 |
| PB000449.01.0 | PFF1470c | 0.0295 | 0.0020 | 0.0686 |
| PB000801.03.0 | PF07_0014 | 0.3816 | 0.1000 | 0.2621 |
| PB000814.00.0 | MAL7P1.19 | 0.1946 | 0.0179 | 0.0921 |
| PB001534.02.0 | MAL7P1.25 | 0.2041 | 0.0105 | 0.0515 |
| PB000346.02.0 | MAL7P1.77 | 0.5421 | 0.1161 | 0.2143 |
| PB000404.03.0 | PF07_0047 | 0.2709 | 0.1484 | 0.5478 |
| PB000351.03.0 | PFF0375c | -1.0000 | 0.0976 | 0.0000 |
| PB000402.00.0 | PFF0500c | 0.0852 | 0.0038 | 0.0447 |
| PB001113.02.0 | PFF0115c | 0.0995 | 0.0045 | 0.0456 |
| PB001156.02.0 | MAL8P1.65 | 0.2363 | 0.0188 | 0.0798 |
| PB000536.01.0 | MAL8P1.47 | 0.1263 | 0.0062 | 0.0491 |
| PB000535.01.0 | MAL8P1.46 | 0.0000 | 0.0000 | 0.0358 |
| PB000955.02.0 | MAL8P1.34 | 0.5985 | 0.0416 | 0.0695 |
| PB000316.00.0 | MAL8P1.12 | 0.2639 | 0.0327 | 0.1240 |
| PB000791.03.0 | PFI0260c | 0.1231 | 0.0057 | 0.0465 |
| PB000085.00.0 | PF07_0101 | 0.5406 | 0.0459 | 0.0849 |
| PB001294.02.0 | PF08_0108 | 0.0935 | 0.0066 | 0.0711 |
| PB000515.01.0 | PF08_0100 | 0.1045 | 0.0648 | 0.6205 |
| PB000360.01.0 | PFI0890c | 0.0703 | 0.0018 | 0.0250 |
| PB000658.03.0 | PFI1345c | 0.2848 | 0.0154 | 0.0541 |
| PB001113.01.0 | PFI1080w | 0.1323 | 0.0069 | 0.0523 |
| PB001114.01.0 | PFI1085w | 0.4376 | 0.0214 | 0.0489 |
| PB001091.02.0 | PFI1490c | 0.4425 | 0.0051 | 0.0116 |
| PB001159.00.0 | PFI1165c | 0.5852 | 0.0101 | 0.0172 |
| **Orthologs of membrane P.berghei genes** | | | | |
| PB000402.03.0 | MAL13P1.299 | 0.0956 | 0.0058 | 0.0606 |
| PB000755.03.0 | MAL13P1.246 | 0.3020 | 0.0197 | 0.0653 |
| PB000148.00.0 | PF13_0252 | 0.1236 | 0.0071 | 0.0574 |
| PB001005.00.0 | PFB0460c | -1.0000 | 0.0911 | 0.0000 |
| PB000826.03.0 | PFB0395w | 0.3298 | 0.0190 | 0.0577 |
| PB001392.02.0 | PFL1370w | 0.2746 | 0.1226 | 0.4464 |
| PB000760.03.0 | PF11_0092 | 0.2118 | 0.0230 | 0.1085 |
| PB000017.02.0 | PF10_0067 | 0.3488 | 0.0530 | 0.1518 |
| PB001432.02.0 | PFE0340c | 0.2082 | 0.0163 | 0.0784 |
| PB000911.00.0 | PFE0965c | 0.0460 | 0.0035 | 0.0753 |
| PB000022.03.0 | PFD1110w | 0.0369 | 0.0035 | 0.0943 |
| PB000309.01.0 | PF07_0087 | 0.0773 | 0.0137 | 0.1779 |

| | | | | |
|---|---|---|---|---|
| PB000048.00.0 | MAL8P1.60 | 0.3119 | 0.0302 | 0.0968 |
| PB000782.00.0 | PF08_0113 | 0.0581 | 0.0098 | 0.1680 |

**Pf/Pr Table B: dn/ds, dn,ds for orthologs of P. berghei female proteins**

| P.berghei gene | P.falciparum ortholog | dn/ds | dn | ds |
|---|---|---|---|---|
| **Orthologs of non-membrane proteins** | | | | |
| PB000120.01.0 | PFC1065w | 0.5419 | 0.1253 | 0.2311 |
| PB000359.02.0 | PFC0775w | 0.0000 | 0.0000 | 0.0219 |
| PB300486.00.0 | PF11_0258 | 0.3252 | 0.0104 | 0.0320 |
| PB001112.00.0 | PF11_0317 | 0.1640 | 0.0225 | 0.1369 |
| PB000700.00.0 | PFA0190c | 0.2037 | 0.0284 | 0.1393 |
| PB000755.01.0 | PF14_0586 | 0.1084 | 0.0053 | 0.0489 |
| PB000149.00.0 | PF13_0250 | 0.0841 | 0.0026 | 0.0312 |
| PB001128.00.0 | PFL0955c | 0.8256 | 0.0068 | 0.0082 |
| PB000124.01.0 | PFL0965c | 0.0552 | 0.0028 | 0.0514 |
| PB000450.01.0 | PFL1630c | 0.1768 | 0.0114 | 0.0648 |
| PB001294.00.0 | PFL1685w | 0.1410 | 0.0061 | 0.0434 |
| PB001146.02.0 | PFC0500w | 0.2455 | 0.0223 | 0.0910 |
| PB000260.00.0 | PFC0535w | -1.0000 | 0.0000 | 0.1049 |
| PB000860.02.0 | PF13_0121 | 0.2181 | 0.0083 | 0.0380 |
| PB000590.02.0 | PF14_0327 | 0.0957 | 0.0099 | 0.1037 |
| PB000874.03.0 | PF14_0334 | 0.2401 | 0.0158 | 0.0657 |
| PB001200.00.0 | PF14_0338 | 0.6749 | 0.0203 | 0.0301 |
| PB001025.02.0 | PF14_0529 | 0.0896 | 0.0083 | 0.0922 |
| PB000187.00.0 | PF14_0538 | 0.1306 | 0.0084 | 0.0641 |
| PB001201.00.0 | PF14_0282 | 0.2165 | 0.0855 | 0.3950 |
| PB000715.01.0 | PF14_0288 | 0.0542 | 0.0025 | 0.0461 |
| PB000918.01.0 | PF11_0043 | 0.0000 | 0.0000 | 0.0426 |
| PB001002.01.0 | PF11_0097 | 0.3737 | 0.0400 | 0.1069 |
| PB000668.02.0 | PF10_0031 | -1.0000 | 0.0530 | 0.0000 |
| PB000032.01.0 | PF10_0040 | 0.3263 | 0.0241 | 0.0740 |
| PB001224.00.0 | PF10_0212 | 0.3269 | 0.0284 | 0.0870 |
| PB000496.03.0 | PF10_0245 | 0.1418 | 0.0069 | 0.0487 |
| PB000503.03.0 | PFE0690c | 0.0228 | 0.0041 | 0.1804 |
| PB001259.02.0 | PFD0470c | 0.1717 | 0.0246 | 0.1435 |
| PB001635.02.0 | PFD0311w | 0.2594 | 0.0069 | 0.0266 |
| PB000982.02.0 | PFD0705c | 0.0498 | 0.0018 | 0.0369 |
| PB001094.00.0 | MAL7P1.100 | 0.0000 | 0.0000 | 0.0323 |
| PB000400.02.0 | PFF0765c | 0.3940 | 0.0309 | 0.0783 |
| PB000291.01.0 | PFF0095c | 0.1223 | 0.0146 | 0.1197 |
| PB001043.01.0 | PF08_0126 | 0.0937 | 0.0094 | 0.1002 |
| PB000846.00.0 | PFI0315c | 0.4883 | 0.0851 | 0.1743 |
| PB000320.02.0 | PFI0365w | 0.4279 | 0.0169 | 0.0395 |
| PB000839.00.0 | MAL8P1.96 | 0.4442 | 0.0385 | 0.0866 |
| PB000989.00.0 | MAL7P1.162 | 0.0792 | 0.0030 | 0.0373 |
| PB000116.00.0 | PFI1330c | 0.2978 | 0.0145 | 0.0487 |
| PB001579.02.0 | PFI1365w | 0.0549 | 0.0070 | 0.1278 |
| PB300680.00.0 | PFI1435w | 0.3791 | 0.0213 | 0.0561 |
| PB000373.01.0 | PFI0655c | 0.1492 | 0.0103 | 0.0693 |
| PB300677.00.0 | PFI0735c | 0.0605 | 0.0064 | 0.1054 |
| **Orthologs of membrane P.berghei genes** | | | | |
| PB000652.01.0 | PF14_0723 | 0.0995 | 0.0057 | 0.0570 |
| PB000768.01.0 | PFL0655w | 0.0000 | 0.0000 | 0.0212 |
| PB001386.02.0 | PFC0381c | 0.3098 | 0.0102 | 0.0329 |
| PB000977.02.0 | PF14_0067 | 0.1237 | 0.0076 | 0.0612 |
| PB000809.01.0 | PFE1340w | 0.1127 | 0.0071 | 0.0627 |

| | | | | |
|---|---|---|---|---|
| PB000125.00.0 | PFF0170w | 0.0511 | 0.0040 | 0.0778 |
| PB000731.00.0 | PFI0935w | 0.0648 | 0.0015 | 0.0232 |
| PB001578.02.0 | PFI1370c | 0.0485 | 0.0072 | 0.1480 |

**Pf/Pr Table C: dn/ds, dn,ds for orthologs of P. berghei proteins expressed in all 3 stages**

| P.berghei gene | P.falciparum ortholog | dn/ds | dn | ds |
|---|---|---|---|---|
| **Orthologs of non-membrane proteins** | | | | |
| PB001124.02.0 | PFC0975c | 0.3969 | 0.1971 | 0.4967 |
| PB000857.02.0 | PFC0635c | 0.0000 | 0.0000 | 0.0148 |
| PB001037.03.0 | PF11_0331 | 0.2533 | 0.0196 | 0.0774 |
| PB000139.01.0 | PF11_0208 | 0.0892 | 0.0018 | 0.0203 |
| PB001300.00.0 | PF11_0270 | 0.1675 | 0.0226 | 0.1352 |
| PB000342.02.0 | PF11_0282 | 0.0000 | 0.0000 | 0.0651 |
| PB000970.00.0 | PF11_0302 | 0.1783 | 0.0190 | 0.1066 |
| PB000391.01.0 | PF11_0313 | 0.0812 | 0.0069 | 0.0845 |
| PB000684.02.0 | MAL13P1.190 | 0.0220 | 0.0017 | 0.0760 |
| PB000686.02.0 | PF13_0213 | 0.0837 | 0.0058 | 0.0688 |
| PB000169.03.0 | PF13_0224 | 0.0377 | 0.0024 | 0.0623 |
| PB000663.03.0 | PF13_0232 | 0.0473 | 0.0434 | 0.9175 |
| PB000914.03.0 | PFA0145c | 0.1176 | 0.0073 | 0.0624 |
| PB001587.02.0 | PF11_0375 | 0.6509 | 0.0463 | 0.0712 |
| PB000103.02.0 | PF11_0396 | 0.1738 | 0.0076 | 0.0437 |
| PB001055.02.0 | PF11_0437 | 0.0000 | 0.0000 | 0.0227 |
| PB000999.00.0 | PF11_0454 | 0.0000 | 0.0000 | 0.0378 |
| PB000374.02.0 | PFA0400c | 0.0446 | 0.0022 | 0.0485 |
| PB000892.02.0 | PFA0520c | 0.0474 | 0.0028 | 0.0600 |
| PB000756.01.0 | PF14_0585 | 0.0000 | 0.0000 | 0.0209 |
| PB000084.03.0 | PF14_0598 | 0.4688 | 0.0190 | 0.0405 |
| PB000303.02.0 | PFL0185c | 0.0207 | 0.0012 | 0.0577 |
| PB000465.03.0 | PFL0210c | 0.0271 | 0.0027 | 0.0993 |
| PB001089.02.0 | PFL0310c | 0.1759 | 0.0108 | 0.0613 |
| PB000738.02.0 | PF13_0322 | 0.0564 | 0.0025 | 0.0438 |
| PB000423.03.0 | PF13_0305 | 0.0000 | 0.0000 | 0.0905 |
| PB000812.02.0 | MAL13P1.233 | 0.6266 | 0.0106 | 0.0168 |
| PB000676.03.0 | PF13_0316 | 0.0000 | 0.0000 | 0.0403 |
| PB000191.00.0 | PFB0635w | 0.0655 | 0.0073 | 0.1113 |
| PB000584.03.0 | PFB0525w | 0.1655 | 0.0084 | 0.0507 |
| PB000272.03.0 | PFB0445c | 0.0900 | 0.0047 | 0.0518 |
| PB000372.03.0 | PFL1420w | 0.2123 | 0.0155 | 0.0729 |
| PB000346.03.0 | PFL1425w | 0.0607 | 0.0071 | 0.1175 |
| PB000323.01.0 | PFL2215w | 0.0666 | 0.0653 | 0.9815 |
| PB000248.00.0 | PFL2345c | 0.1121 | 0.0474 | 0.4228 |
| PB000649.02.0 | PFL1465c | 0.0000 | 0.0000 | 0.0399 |
| PB000555.01.0 | PFL0625c | 0.0575 | 0.0578 | 1.0037 |
| PB000053.00.0 | PFL1070c | 0.1898 | 0.0963 | 0.5076 |
| PB000878.03.0 | PFL1110c | 0.1821 | 0.0029 | 0.0160 |
| PB000966.02.0 | PFL1550w | 0.0677 | 0.0052 | 0.0765 |
| PB000038.03.0 | PFL2005w | 0.0000 | 0.0000 | 0.0138 |
| PB001545.02.0 | PFL0725w | 0.0433 | 0.0020 | 0.0455 |
| PB001286.00.0 | PFL1170w | 0.4222 | 0.0063 | 0.0149 |
| PB000303.03.0 | PFL2055w | 0.0000 | 0.0000 | 0.0243 |
| PB000300.03.0 | PFL2060c | 0.0257 | 0.0019 | 0.0737 |
| PB000204.03.0 | PFL1245w | 0.0989 | 0.0063 | 0.0634 |
| PB001056.01.0 | PFL1270w | 0.0980 | 0.0102 | 0.1041 |
| PB000406.00.0 | PFL0895c | 0.0114 | 0.0012 | 0.1054 |
| PB001126.00.0 | PFL0930w | 0.1078 | 0.0083 | 0.0772 |

| | | | | |
|---|---|---|---|---|
| PB001005.01.0 | PFB0840w | 0.0000 | 0.0000 | 0.0339 |
| PB001184.00.0 | PFC0290w | 0.0000 | 0.0000 | 0.0547 |
| PB001183.00.0 | PFC0295c | 0.0000 | 0.0000 | 0.0111 |
| PB000831.01.0 | PFC0350c | 0.0263 | 0.0032 | 0.1218 |
| PB001185.00.0 | PFC0190c | 0.0000 | 0.0000 | 0.0331 |
| PB000158.03.0 | PFB0815w | 0.0124 | 0.0017 | 0.1351 |
| PB000183.00.0 | PF13_0143 | 0.0221 | 0.0039 | 0.1777 |
| PB001087.02.0 | MAL13P1.92 | 0.0000 | 0.0000 | 0.0777 |
| PB000178.03.0 | MAL13P1.135 | 0.0330 | 0.0020 | 0.0620 |
| PB000079.01.0 | MAL13P1.63 | 0.0194 | 0.0884 | 4.5513 |
| PB000709.01.0 | PF14_0053 | 0.0000 | 0.0000 | 0.0552 |
| PB000909.03.0 | PF14_0324 | 0.0536 | 0.0052 | 0.0966 |
| PB001040.00.0 | PF14_0359 | 0.0168 | 0.0010 | 0.0596 |
| PB000037.01.0 | PF14_0368 | 0.0702 | 0.0022 | 0.0315 |
| PB001103.02.0 | PF14_0391 | 0.0000 | 0.0000 | 0.0148 |
| PB001359.02.0 | PF14_0448 | 0.0000 | 0.0000 | 0.0840 |
| PB001100.02.0 | PF14_0393 | 0.0349 | 0.0016 | 0.0467 |
| PB001028.00.0 | PF14_0401 | 0.2645 | 0.0153 | 0.0580 |
| PB001310.02.0 | PF14_0511 | 0.0650 | 0.0063 | 0.0963 |
| PB000629.00.0 | PF14_0520 | 0.0000 | 0.0000 | 0.0368 |
| PB000757.02.0 | PF14_0425 | 0.0000 | 0.0000 | 0.0115 |
| PB000617.02.0 | PF14_0146 | 0.0944 | 0.0044 | 0.0462 |
| PB001143.02.0 | PF14_0083 | 0.1106 | 0.0070 | 0.0636 |
| PB300230.00.0 | PF14_0164 | 0.0756 | 0.0046 | 0.0605 |
| PB001285.00.0 | PF14_0096 | 0.2956 | 0.0808 | 0.2732 |
| PB000776.00.0 | PF14_0104 | 0.0000 | 0.0000 | 0.0598 |
| PB001317.02.0 | PF14_0192 | 0.0897 | 0.0053 | 0.0589 |
| PB000993.03.0 | PF14_0196 | 1.8933 | 0.0949 | 0.0501 |
| PB000415.00.0 | PF10_0264 | 0.0223 | 0.0016 | 0.0738 |
| PB000212.00.0 | PF11_0071 | 0.2000 | 0.1127 | 0.5635 |
| PB000240.03.0 | PF11_0142 | 0.1405 | 0.0068 | 0.0486 |
| PB000857.01.0 | PF11_0183 | 0.0000 | 0.0000 | 0.0538 |
| PB000524.01.0 | PF11_0188 | 0.1906 | 0.0329 | 0.1724 |
| PB000776.01.0 | PF10_0111 | 0.0000 | 0.0000 | 0.0059 |
| PB000924.03.0 | PF10_0115 | 0.0636 | 0.0094 | 0.1478 |
| PB000253.01.0 | PF10_0123 | 0.0480 | 0.0030 | 0.0634 |
| PB000578.03.0 | PF10_0289 | 0.1259 | 0.0058 | 0.0457 |
| PB001420.02.0 | PF10_0325 | 0.1243 | 0.0059 | 0.0471 |
| PB001167.00.0 | PF10_0174 | 0.1639 | 0.0195 | 0.1191 |
| PB000601.02.0 | PF10_0081 | 0.3711 | 0.1919 | 0.5171 |
| PB000164.02.0 | PF10_0084 | 0.9808 | 0.2430 | 0.2478 |
| PB001437.02.0 | PF10_0086 | 0.0298 | 0.0019 | 0.0642 |
| PB000500.03.0 | PFE0660c | 0.1267 | 0.0071 | 0.0561 |
| PB000246.02.0 | PFE1050w | 0.0530 | 0.0018 | 0.0335 |
| PB000652.02.0 | PFE1370w | 0.0627 | 0.0112 | 0.1788 |
| PB001230.00.0 | PFE1195w | 0.0166 | 0.0012 | 0.0703 |
| PB000511.03.0 | PFE0975c | 0.1893 | 0.0196 | 0.1038 |
| PB000187.03.0 | PFE1250w | 0.3163 | 0.0195 | 0.0616 |
| PB001067.00.0 | PFE1005w | 0.0413 | 0.0024 | 0.0578 |
| PB000415.02.0 | PFD0830w | 0.3036 | 0.0062 | 0.0204 |
| PB000557.01.0 | PFD0665c | 0.2626 | 0.1194 | 0.4546 |
| PB001177.02.0 | PFD0305c | 0.0000 | 0.0000 | 0.1275 |
| PB000269.02.0 | PFF0325c | 0.4176 | 0.0319 | 0.0763 |
| PB300823.00.0 | PF07_0029 | 0.1808 | 0.0123 | 0.0680 |
| PB000242.00.0 | PF07_0033 | 0.0548 | 0.0063 | 0.1154 |
| PB001409.02.0 | PF07_0079 | 0.0000 | 0.0000 | 0.0545 |
| PB001410.02.0 | PF07_0080 | 0.2513 | 0.0033 | 0.0130 |
| PB000600.00.0 | PF07_0072 | 0.2646 | 0.1584 | 0.5987 |

| | | | | |
|---|---|---|---|---|
| PB000680.00.0 | MAL7P1.122 | 0.0292 | 0.0023 | 0.0775 |
| PB000727.00.0 | PFF1155w | 0.0392 | 0.0009 | 0.0221 |
| PB001086.01.0 | PFF0430w | 0.0241 | 0.0027 | 0.1101 |
| PB000128.01.0 | PFF0895w | 0.1174 | 0.0083 | 0.0706 |
| PB001252.02.0 | PFF0530w | 0.0912 | 0.0032 | 0.0349 |
| PB000171.02.0 | PFF0940c | 0.0346 | 0.0009 | 0.0254 |
| PB000458.03.0 | PFF1345w | 0.1052 | 0.0074 | 0.0706 |
| PB000923.00.0 | PFF0250w | 0.0314 | 0.0017 | 0.0544 |
| PB001129.00.0 | PF08_0115 | 0.4553 | 0.1229 | 0.2699 |
| PB001208.00.0 | PF08_0110 | 0.1986 | 0.0099 | 0.0499 |
| PB000282.03.0 | PF08_0109 | 0.4648 | 0.1074 | 0.2311 |
| PB000281.03.0 | MAL8P1.125 | 0.0845 | 0.0046 | 0.0539 |
| PB000705.00.0 | PF07_0112 | 0.0501 | 0.0070 | 0.1397 |
| PB001264.00.0 | PF07_0117 | 0.0135 | 0.0017 | 0.1244 |
| PB000057.03.0 | PF08_0096 | 0.2422 | 0.0274 | 0.1129 |
| PB000359.01.0 | PFI0895c | 0.0835 | 0.0053 | 0.0637 |
| PB001284.02.0 | PFI0930c | 0.1483 | 0.0826 | 0.5571 |
| PB001037.00.0 | PFI1310w | 0.1743 | 0.0099 | 0.0568 |
| PB000019.03.0 | PFI0490c | 0.0266 | 0.0018 | 0.0668 |
| PB000440.00.0 | PFI1020c | 0.0407 | 0.0027 | 0.0657 |
| PB001562.02.0 | PFI1445w | 0.3126 | 0.0222 | 0.0709 |
| PB001445.02.0 | PFI0630w | 0.1654 | 0.0076 | 0.0458 |
| PB001157.00.0 | PFI1170c | 0.0202 | 0.0008 | 0.0395 |
| PB000520.00.0 | PFI0755c | 0.0593 | 0.0034 | 0.0566 |
| **Orthologs of membrane P.berghei genes** | | | | |
| PB000058.03.0 | PF11_0301 | 0.0214 | 0.0014 | 0.0634 |
| PB000390.00.0 | PF14_0678 | 0.3544 | 0.0308 | 0.0870 |
| PB000189.03.0 | MAL13P1.351 | 0.8816 | 0.1024 | 0.1161 |
| PB001022.02.0 | PFC0275w | 0.2578 | 0.0133 | 0.0515 |
| PB000405.02.0 | PFC0110w | 0.0785 | 0.0231 | 0.2943 |
| PB000843.02.0 | MAL13P1.56 | 0.0437 | 0.0042 | 0.0964 |
| PB000185.00.0 | PF13_0141 | 0.0000 | 0.0000 | 0.0623 |
| PB001066.03.0 | PF11_0055 | 0.1337 | 0.0135 | 0.1012 |
| PB000982.00.0 | PF11_0164 | 0.1961 | 0.0113 | 0.0578 |
| PB000856.03.0 | PF11_0174 | 0.0351 | 0.0024 | 0.0675 |
| PB001003.01.0 | PF11_0098 | 0.2293 | 0.0121 | 0.0529 |
| PB001210.00.0 | MAL8P1.128 | 0.2592 | 0.0047 | 0.0179 |

**Pf/Pr Table D: dn/ds, dn,ds for orthologs of P. berghei proteins asexual blood stages**

| P.berghei gene | P.falciparum orthol | dn/ds | dn | ds |
|---|---|---|---|---|
| **Orthologs of non-membrane proteins** | | | | |
| PB000567.03.0 | PFC0710w | 0.2399 | 0.0223 | 0.0930 |
| PB000887.00.0 | PF11_0268 | 0.8631 | 0.0252 | 0.0292 |
| PB000649.03.0 | PF13_0219 | 0.1205 | 0.0146 | 0.1213 |
| PB001070.00.0 | PF13_0234 | 0.0592 | 0.0059 | 0.0991 |
| PB001039.01.0 | PFA0260c | 0.0000 | 0.0000 | 0.0317 |
| PB000636.02.0 | PFA0440w | 0.5135 | 0.0038 | 0.0073 |
| PB000862.03.0 | PF13_0315 | 0.0369 | 0.0030 | 0.0810 |
| PB000739.02.0 | PF13_0323 | 1.5860 | 0.1662 | 0.1048 |
| PB000065.02.0 | PF13_0350 | 0.0750 | 0.0068 | 0.0904 |
| PB301484.00.0 | MAL13P1.344 | 0.0120 | 0.0014 | 0.1162 |
| PB001222.02.0 | MAL13P1.221 | 0.4183 | 0.0150 | 0.0360 |
| PB000190.00.0 | PFB0640c | 0.0887 | 0.0067 | 0.0751 |
| PB000472.03.0 | PFB0680w | -1.0000 | 0.0760 | 0.0000 |
| PB000091.03.0 | PFB0745w | 0.3199 | 0.0392 | 0.1224 |
| PB001623.02.0 | PFL0975w | 0.7099 | 0.0601 | 0.0847 |
| PB000193.03.0 | PFL2225w | 0.1031 | 0.0062 | 0.0599 |
| PB000192.03.0 | PFL2230c | 0.2421 | 0.0127 | 0.0523 |
| PB000523.03.0 | PFL2245w | 0.0612 | 0.0034 | 0.0547 |
| PB000522.03.0 | PFL2250c | 0.2909 | 0.0382 | 0.1312 |
| PB300427.00.0 | PFL2355w | 0.6657 | 0.1102 | 0.1656 |
| PB001121.02.0 | MAL13P1.19 | -1.0000 | 0.0357 | 0.0000 |
| PB000042.01.0 | PFL1065c | 0.1115 | 0.0035 | 0.0585 |
| PB001149.01.0 | PFL1480w | 0.2373 | 0.0166 | 0.0698 |
| PB001011.01.0 | PFL1530w | 0.1129 | 0.0536 | 0.4749 |
| PB000206.00.0 | PFL1605w | 0.3241 | 0.0266 | 0.0820 |
| PB000549.03.0 | PFL2100w | -1.0000 | 0.0000 | 0.0000 |
| PB000668.01.0 | PFL1385c | -1.0000 | 0.0610 | 0.0000 |
| PB000615.00.0 | PFC0340w | 0.5167 | 0.0666 | 0.1288 |
| PB000833.01.0 | PFC0365w | 0.1374 | 0.0100 | 0.0731 |
| PB000523.01.0 | PFC0400w | 0.1379 | 0.0039 | 0.0280 |
| PB000108.03.0 | PFB0355c | 0.4970 | 0.0336 | 0.0675 |
| PB000902.01.0 | PFB0200c | 0.2136 | 0.0117 | 0.0546 |
| PB000368.00.0 | MAL13P1.121 | 1.1421 | 0.1002 | 0.0878 |
| PB000665.00.0 | PF13_0137 | -1.0000 | 0.0404 | 0.0000 |
| PB000626.00.0 | PF14_0224 | 0.0671 | 0.0052 | 0.0771 |
| PB000730.02.0 | PF14_0344 | -1.0000 | 0.1672 | 0.0000 |
| PB001039.00.0 | PF14_0360 | 0.0593 | 0.0042 | 0.0701 |
| PB000893.01.0 | PF14_0068 | 0.0698 | 0.0043 | 0.0614 |
| PB000848.00.0 | PF14_0088 | 0.4541 | 0.0178 | 0.0393 |
| PB000638.02.0 | PF14_0020 | 0.0636 | 0.0038 | 0.0598 |
| PB001570.02.0 | PF14_0026 | 0.5857 | 0.0615 | 0.1049 |
| PB000779.00.0 | PF14_0102 | 0.7385 | 0.0284 | 0.0385 |
| PB000713.00.0 | PF14_0277 | 0.1260 | 0.0162 | 0.1284 |
| PB300187.00.0 | PF10_0093 | 0.2529 | 0.0758 | 0.2998 |
| PB000011.01.0 | PF10_0099 | 0.2372 | 0.0237 | 0.1001 |
| PB000772.02.0 | PF11_0114 | 0.1552 | 0.0094 | 0.0603 |
| PB001627.02.0 | PF11_0189 | -1.0000 | 0.2043 | 0.0000 |
| PB000828.01.0 | PF10_0028 | 0.2494 | 0.0066 | 0.0266 |
| PB000096.01.0 | PF10_0039 | 0.0000 | 0.0000 | 0.0241 |
| PB000603.00.0 | PF10_0306 | 0.0698 | 0.0069 | 0.0993 |

| | | | | |
|---|---|---|---|---|
| PB000497.03.0 | PF10_0246 | 0.2841 | 0.0152 | 0.0535 |
| PB001435.02.0 | PF10_0087 | 0.0241 | 0.0031 | 0.1280 |
| PB001632.02.0 | PFE0375w | -1.0000 | 0.0556 | 0.0000 |
| PB300407.00.0 | PFE0605c | 0.4651 | 0.0233 | 0.0501 |
| PB000735.02.0 | PFD0515w | 0.0853 | 0.0064 | 0.0744 |
| PB000152.01.0 | PFD0605c | 0.0910 | 0.0173 | 0.1898 |
| PB000695.01.0 | PF07_0057 | 0.4270 | 0.0846 | 0.1981 |
| PB001175.01.0 | PFF0950w | -1.0000 | 0.0468 | 0.0000 |
| PB000459.03.0 | PFF1350c | 0.1607 | 0.0133 | 0.0826 |
| PB000093.00.0 | PFF0675c | 0.4407 | 0.0328 | 0.0744 |
| PB000895.03.0 | PFF1055c | 0.2168 | 0.0178 | 0.0821 |
| PB000019.02.0 | PF08_0086 | 0.1683 | 0.0107 | 0.0635 |
| PB001117.03.0 | MAL8P1.62 | 0.6149 | 0.1196 | 0.1945 |
| PB001369.02.0 | MAL8P1.55 | 0.2791 | 0.1147 | 0.4111 |
| PB001042.03.0 | PFI0190w | 0.0000 | 0.0000 | 0.0522 |
| PB001041.03.0 | PFI0195c | 0.3620 | 0.0088 | 0.0244 |
| PB000084.00.0 | MAL7P1.126 | 0.3338 | 0.0222 | 0.0665 |
| PB000722.00.0 | MAL8P1.73 | 0.2692 | 0.0250 | 0.0927 |
| PB000805.02.0 | PFI0820c | 1.0032 | 0.1323 | 0.1318 |
| PB000532.02.0 | PFI1685w | 0.0000 | 0.0000 | 0.0517 |
| PB000051.00.0 | PFI1130c | 0.0852 | 0.0038 | 0.0450 |
| PB000214.02.0 | PFI0705w | 0.0872 | 0.0037 | 0.0421 |
| PB001160.00.0 | PFI1155w | 0.1333 | 0.0499 | 0.3746 |
| **Orthologs of membrane P.berghei genes** | | | | |
| PB000162.00.0 | PF11_0203 | 0.0808 | 0.0055 | 0.0679 |
| PB001081.01.0 | PF11_0246 | 0.2300 | 0.0519 | 0.2257 |
| PB000821.01.0 | PF11_0344 | 0.6248 | 0.0412 | 0.0660 |
| PB000392.02.0 | PF14_0660 | 0.3327 | 0.0147 | 0.0443 |
| PB000519.03.0 | PFL0410w | 0.6781 | 0.0260 | 0.0383 |
| PB000944.03.0 | PF13_0270 | 1.1168 | 0.1385 | 0.1240 |
| PB000884.03.0 | MAL13P1.309 | 0.3086 | 0.0074 | 0.0241 |
| PB000345.03.0 | PF13_0265 | -1.0000 | 0.0711 | 0.0000 |
| PB000909.00.0 | PFL2505c | 0.3131 | 0.0205 | 0.0655 |
| PB000683.03.0 | PF13_0032 | 0.4185 | 0.0821 | 0.1963 |
| PB000072.03.0 | MAL13P1.39 | 0.6865 | 0.0936 | 0.1364 |
| PB000992.02.0 | PFL1700c | 0.2535 | 0.0114 | 0.0451 |
| PB000443.01.0 | PF13_0102 | 0.0763 | 0.0062 | 0.0807 |
| PB001439.02.0 | PF13_0133 | 0.1880 | 0.0100 | 0.0532 |
| PB000379.02.0 | PF14_0495 | -1.0000 | 0.0565 | 0.0000 |
| PB000505.02.0 | PF14_0530 | 0.1509 | 0.0658 | 0.4359 |
| PB000294.03.0 | PF14_0186 | 0.2682 | 0.0339 | 0.1263 |
| PB001106.00.0 | PF10_0363 | 0.0464 | 0.0034 | 0.0725 |
| PB000986.00.0 | PF11_0107 | 0.2668 | 0.0281 | 0.1055 |
| PB001171.02.0 | PF11_0112 | 0.1273 | 0.0129 | 0.1012 |
| PB000995.02.0 | PFE1445c | 0.0167 | 0.0018 | 0.1047 |
| PB000619.02.0 | PFD0720w | 0.6934 | 0.0091 | 0.0131 |
| PB001406.02.0 | MAL8P1.53 | 0.0930 | 0.0068 | 0.0732 |
