# Supplementary material for: Why are male malaria parasites in such a rush? Sex-specific evolution and host–parasite interactions
Source: Evol Med Public Health. 2012 Nov 26;2013(1):3–13. doi: 10.1093/emph/eos003 (PMC4183958; doi:10.1093/emph/eos003)
Supplement: Supplementary Data [file supp_eos003_suppl_data.zip › REECE_Table_S1_PV.pdf]

Pv/Pk Table A: dn/ds, dn,ds for orthologs of P. berghei male proteins

P.berghei gene	P.vivax ortholog	dn/ds	dn	ds
		**Orthologs of non-membrane genes**		
PB000801.03.0	Pv087730	0.0896	0.0387	0.4317
PB000814.00.0	Pv087770	0.1079	0.0621	0.5756
PB000792.01.0	Pv087805	0.0422	0.0250	0.5928
PB001534.02.0	Pv087925	0.1228	0.0740	0.6029
PB001294.02.0	Pv088125	0.0421	0.0355	0.8424
PB000404.03.0	Pv088085	0.0306	0.0312	1.0199
PB000246.00.0	Pv003715	0.0441	0.0458	1.0370
PB001162.01.0	Pv003900	0.0458	0.0475	1.0368
PB000658.03.0	Pv099810	0.1458	0.0838	0.5750
PB000659.03.0	Pv099815	0.0000	0.0000	0.4562
PB000403.00.0	Pv003905	0.0998	0.0784	0.7860
PB001581.02.0	Pv099850	0.0842	0.0470	0.5579
PB001156.02.0	Pv089480	0.0747	0.0606	0.8120
PB000536.01.0	Pv089245	0.2045	0.0980	0.4791
PB000316.00.0	Pv088915	0.1041	0.0618	0.5940
PB000535.01.0	Pv089240	0.0194	0.0148	0.7603
PB000955.02.0	Pv089150	0.0767	0.0591	0.7706
PB000958.02.0	Pv089130	0.1352	0.0657	0.4859
PB000085.00.0	Pv096155	0.0546	0.0579	1.0604
PB000313.02.0	Pv098920	0.0741	0.0539	0.7264
PB000742.02.0	Pv099935	0.0692	0.0425	0.6137
PB000360.01.0	Pv099330	0.0626	0.0294	0.4691
PB001091.02.0	Pv099995	0.1167	0.0404	0.3467
PB000898.02.0	Pv099295	0.0454	0.0384	0.8477
PB300096.00.0	Pv089995	0.0219	0.0217	0.9925
PB000174.03.0	Pv090025	0.1345	0.0587	0.4364
PB000847.00.0	Pv098760	0.0858	0.0475	0.5536
PB001159.00.0	Pv099595	0.1121	0.0793	0.7077
PB000413.02.0	Pv099190	0.1318	0.0463	0.3516
PB000138.01.0	Pv098720	0.1010	0.0579	0.5731
PB000791.03.0	Pv098710	0.0366	0.0218	0.5966
PB001114.01.0	Pv099520	0.0831	0.0552	0.6647
PB001113.01.0	Pv099515	0.0748	0.0525	0.7022
PB000608.02.0	Pv000650	0.0337	0.0380	1.1295
PB000439.03.0	Pv082640	0.0459	0.0483	1.0518
PB000161.02.0	Pv082635	0.0404	0.0365	0.9052
PB001391.02.0	Pv081420	0.0000	0.0000	0.9035
PB001614.02.0	Pv096120	0.1468	0.0936	0.6379
PB000729.02.0	Pv000720	0.0352	0.0206	0.5837
PB000739.01.0	Pv000820	0.0309	0.0161	0.5193
PB000919.03.0	Pv083195	0.0391	0.0199	0.5079
PB300815.00.0	Pv000955	0.0511	0.0396	0.7750
PB000031.01.0	Pv093605	0.0116	0.0139	1.1998
PB000336.00.0	Pv083065	0.0637	0.0396	0.6214
PB000794.02.0	Pv000985	0.1274	0.0589	0.4620
PB000754.03.0	Pv083330	0.0714	0.0603	0.8447
PB000547.03.0	Pv000600	0.1028	0.0563	0.5473
PB000346.02.0	Pv000610	0.0407	0.0419	1.0313
PB301132.00.0	Pv084780	0.0538	0.0549	1.0197

PB000808.00.0	Pv085440	0.0626	0.0746	1.1912
PB000322.00.0	Pv085845	0.3017	0.0886	0.2937
PB001172.00.0	Pv085850	0.1177	0.0523	0.4443
PB000952.01.0	Pv085870	0.1553	0.0648	0.4172
PB106442.00.0	Pv086225	0.5435	0.0867	0.1595
PB100371.00.0	Pv086280	0.3005	0.0845	0.2811
PB000852.00.0	Pv086010	0.0914	0.0340	0.3726
PB000359.00.0	Pv086025	0.0708	0.0458	0.6467
PB001056.02.0	Pv111560	0.0222	0.0219	0.9869
PB000872.03.0	Pv111535	0.0952	0.0567	0.5962
PB000977.01.0	Pv086105	0.0519	0.0395	0.7604
PB001011.00.0	Pv111520	0.0281	0.0321	1.1457
PB000382.03.0	Pv111460	0.0270	0.0268	0.9911
PB301567.00.0	Pv111435	0.1351	0.0831	0.6151
PB001397.02.0	Pv081250	0.0302	0.0225	0.7468
PB001662.02.0	Pv085210	0.0460	0.0557	1.2123
PB300899.00.0	Pv084385	0.0609	0.0439	0.7214
PB000463.03.0	Pv084910	0.0731	0.0391	0.5352
PB000558.02.0	Pv084475	0.0607	0.0328	0.5409
PB000718.01.0	Pv085000	0.1080	0.0549	0.5086
PB001204.00.0	Pv085035	0.0565	0.0349	0.6177
PB000726.03.0	Pv084615	0.0105	0.0078	0.7433
PB000867.02.0	Pv084195	0.0625	0.0501	0.8016
PB001267.00.0	Pv084685	0.0152	0.0169	1.1135
PB000336.03.0	Pv084235	0.0000	0.0000	0.4889
PB000651.02.0	Pv116760	0.0603	0.0639	1.0595
PB000539.02.0	Pv116755	0.0442	0.0408	0.9234
PB300889.00.0	Pv118285	0.0250	0.0061	0.2458
PB000094.02.0	Pv118290	0.1967	0.0717	0.3642
PB000020.01.0	Pv118600	0.2446	0.0795	0.3251
PB001029.00.0	Pv118390	0.0121	0.0050	0.4132
PB000139.00.0	Pv118460	0.0405	0.0212	0.5241
PB000324.00.0	Pv118465	0.0452	0.0324	0.7164
PB000487.03.0	Pv118560	0.0562	0.0356	0.6338
PB000485.00.0	Pv110870	0.0000	0.0000	0.2156
PB001088.00.0	Pv117895	0.0000	0.0000	0.3203
PB000878.01.0	Pv110865	0.1135	0.0449	0.3951
PB000433.03.0	Pv118190	0.0639	0.0239	0.3733
PB000849.01.0	Pv117295	0.0618	0.0273	0.4417
PB000774.03.0	Pv111195	0.0305	0.0233	0.7652
PB000074.01.0	Pv118085	0.1271	0.0929	0.7307
PB000435.01.0	Pv117185	0.2288	0.0858	0.3749
PB000005.01.0	Pv117175	0.0539	0.0362	0.6727
PB001418.02.0	Pv111045	0.0480	0.0247	0.5136
PB000673.03.0	Pv117530	0.0325	0.0241	0.7412
PB000914.01.0	Pv080690	0.2141	0.0371	0.1734
PB301405.00.0	Pv080640	0.0986	0.0201	0.2037
PB000535.02.0	Pv123855	0.0103	0.0072	0.7034
PB000159.02.0	Pv124135	0.4389	0.0661	0.1506
PB000619.01.0	Pv123515	0.0812	0.0447	0.5510
PB001094.01.0	Pv123470	0.1274	0.0576	0.4524
PB001200.02.0	Pv123370	0.0000	0.0000	0.2518
PB000265.02.0	Pv123320	0.1279	0.0519	0.4059

PB000515.01.0	Pv123295	0.0355	0.0128	0.3599
PB000182.01.0	Pv095410	0.0319	0.0452	1.4168
PB000469.02.0	Pv094525	0.0495	0.0196	0.3963
PB000273.00.0	Pv080070	0.0249	0.0059	0.2370
PB000680.01.0	Pv080035	0.2851	0.0932	0.3269
PB301233.00.0	Pv094770	0.0295	0.0136	0.4615
PB000846.01.0	Pv095235	0.0454	0.0216	0.4768
PB001351.02.0	Pv079890	0.0323	0.0154	0.4754
PB001116.00.0	Pv094875	0.0926	0.0480	0.5182
PB000189.00.0	Pv119815	0.0000	0.0000	0.5319
PB000099.01.0	Pv002615	0.0000	0.0000	0.5251
PB001187.00.0	Pv002750	0.0188	0.0216	1.1480
PB001287.00.0	Pv002855	0.1709	0.0751	0.4397
PB000254.03.0	Pv097665	0.0896	0.0532	0.5936
PB000958.00.0	Pv097980	0.0573	0.0407	0.7106
PB000569.03.0	Pv098020	0.0240	0.0365	1.5248
PB000192.02.0	Pv098025	0.0200	0.0169	0.8472
PB001175.02.0	Pv098060	0.1479	0.0836	0.5653
PB001130.01.0	Pv098085	0.0852	0.0348	0.4089
PB001071.01.0	Pv097850	0.0687	0.0277	0.4026
PB001058.03.0	Pv097595	0.1768	0.0569	0.3217
PB000945.02.0	Pv123260	0.1928	0.0547	0.2837
PB000269.01.0	Pv122740	0.0420	0.0262	0.6245
PB001086.02.0	Pv122715	0.1058	0.0565	0.5344
PB000292.00.0	Pv122235	0.2632	0.0663	0.2520
PB001422.02.0	Pv123220	0.0720	0.0314	0.4358
PB000953.02.0	Pv122160	0.2883	0.1000	0.3468
PB000609.01.0	Pv119270	0.0087	0.0097	1.1187
PB000070.01.0	Pv122060	0.1323	0.0457	0.3453
PB000747.02.0	Pv119415	0.0414	0.0492	1.1877
PB000132.01.0	Pv122560	0.0617	0.0412	0.6670
PB001007.00.0	Pv122035	0.0794	0.0246	0.3094
PB001008.01.0	Pv122505	0.1268	0.0544	0.4293
PB001114.03.0	Pv119485	0.0246	0.0142	0.5758
PB001305.02.0	Pv122935	0.0801	0.0321	0.4007
PB001064.02.0	Pv122445	0.1488	0.0562	0.3779
PB001137.00.0	Pv122385	0.0869	0.0406	0.4669
PB000733.03.0	Pv091760	0.0367	0.0340	0.9274
PB000993.01.0	Pv091775	0.1113	0.0520	0.4671
PB001060.00.0	Pv091055	0.1033	0.0741	0.7167
PB000644.03.0	Pv091025	0.0518	0.0137	0.2649
PB000607.03.0	Pv090910	0.1146	0.0711	0.6201
PB001067.03.0	Pv090905	0.0753	0.0647	0.8595
PB001145.01.0	Pv091040	0.1460	0.0917	0.6277
PB000334.00.0	Pv087110	0.1338	0.0340	0.2542
PB000690.03.0	Pv114930	0.0389	0.0151	0.3890
PB000318.01.0	Pv115240	0.1346	0.0374	0.2776
PB001060.02.0	Pv114995	0.0779	0.0475	0.6090
PB000002.03.0	Pv115305	0.0842	0.0465	0.5529
PB001373.02.0	Pv115030	0.0174	0.0098	0.5633
PB000171.00.0	Pv115405	0.0955	0.0599	0.6270
PB000059.02.0	Pv092710	0.0686	0.0474	0.6913
PB001051.02.0	Pv092745	0.0545	0.0188	0.3455

PB000354.00.0	Pv091345	0.1603	0.0571	0.3562
PB000659.00.0	Pv091340	0.0228	0.0156	0.6829
PB000427.02.0	Pv092355	0.1760	0.0748	0.4252
PB000611.02.0	Pv091955	0.0381	0.0418	1.0983
PB000089.03.0	Pv092025	0.0383	0.0406	1.0598
PB001615.02.0	Pv092500	0.1348	0.0508	0.3765
PB300319.00.0	Pv092510	0.2495	0.0880	0.3525
PB001001.01.0	Pv091095	0.0028	0.0040	1.4286
PB000781.01.0	Pv092145	0.0415	0.0484	1.1663
PB000656.03.0	Pv092600	0.0457	0.0244	0.5346
PB000009.01.0	Pv092605	0.1089	0.0438	0.4026
PB000827.02.0	Pv100665	0.0429	0.0451	1.0505
PB000662.00.0	Pv100740	0.0777	0.0673	0.8664
PB000554.01.0	Pv100960	0.0609	0.0427	0.7004
PB000550.03.0	Pv101080	0.0490	0.0127	0.2599
PB000821.00.0	Pv101175	0.0098	0.0198	2.0309
PB001113.02.0	Pv113285	0.0492	0.0300	0.6087
PB000402.00.0	Pv113655	0.0959	0.0613	0.6395
PB000449.01.0	Pv114615	0.0362	0.0230	0.6337
PB001273.00.0	Pv114255	0.0169	0.0286	1.6937
PB000378.00.0	Pv114640	0.1617	0.0373	0.2310
PB000351.03.0	Pv113545	0.1099	0.0580	0.5273
PB000249.02.0	Pv114310	0.0476	0.0228	0.4785
PB000309.00.0	Pv114340	0.0426	0.0302	0.7085
PB000306.03.0	Pv114795	0.0111	0.0042	0.3764
PB001179.01.0	Pv113910	0.0192	0.0178	0.9264
PB000293.00.0	Pv114825	0.0206	0.0063	0.3038
PB001081.00.0	Pv114840	0.0815	0.0501	0.6147
PB001076.00.0	Pv114850	0.0904	0.0473	0.5231
PB000865.02.0	Pv113440	0.1281	0.0624	0.4867
PB000570.03.0	Pv114430	0.0643	0.0531	0.8256
PB000820.02.0	Pv113360	0.0528	0.0270	0.5108
**Orthologs of membrane genes**				
PB000782.00.0	Pv088205	0.0043	0.0113	2.6501
PB000250.01.0	Pv088185	0.1237	0.0608	0.4910
PB000826.03.0	Pv003895	0.0610	0.0332	0.5437
PB001005.00.0	Pv003960	0.0821	0.0650	0.7909
PB000048.00.0	Pv089375	0.2209	0.0784	0.3548
PB000665.02.0	Pv089095	0.1007	0.0236	0.2341
PB000309.01.0	Pv096280	0.0124	0.0203	1.6416
PB000022.03.0	Pv090215	0.0187	0.0072	0.3835
PB001036.01.0	Pv082715	0.0304	0.0307	1.0088
PB000602.02.0	Pv082600	0.0667	0.0407	0.6107
PB000148.00.0	Pv083260	0.0683	0.0529	0.7742
PB000755.03.0	Pv083335	0.0701	0.0540	0.7696
PB001426.02.0	Pv117830	0.1536	0.0616	0.4013
PB000096.02.0	Pv117330	0.0954	0.0411	0.4311
PB000912.01.0	Pv080675	0.0906	0.0239	0.2637
PB001392.02.0	Pv124045	0.0212	0.0198	0.9325
PB000183.01.0	Pv095405	0.0465	0.0402	0.8653
PB000017.02.0	Pv094530	0.0997	0.0855	0.8580
PB000073.02.0	Pv095230	0.0164	0.0067	0.4072
PB000911.00.0	Pv080285	0.0232	0.0091	0.3911

PB001432.02.0	Pv097905	0.1084	0.0588	0.5423
PB000674.02.0	Pv122045	0.0311	0.0179	0.5766
PB000489.01.0	Pv091015	0.2220	0.0960	0.4326
PB000760.03.0	Pv091075	0.0355	0.0288	0.8095
PB000390.01.0	Pv092105	0.0411	0.0459	1.1170
PB000003.01.0	Pv114705	0.0000	0.0000	0.2269

Pv/Pk Table B: dn/ds, dn,ds for orthologs of P. berghei female proteins

P.berghei gene	P.vivax ortholog	dn/ds	dn	ds
Orthologs of non-membrane genes				
PB000995.03.0	Pv003955	0.0000	0.0000	0.4963
PB000116.00.0	Pv099790	0.0480	0.0401	0.8356
PB001579.02.0	Pv099830	0.0694	0.0324	0.4676
PB000982.02.0	Pv089685	0.0773	0.0244	0.3158
PB001366.02.0	Pv089625	0.0000	0.0000	0.6478
PB000456.01.0	Pv089590	0.0000	0.0000	0.6689
PB300680.00.0	Pv099915	0.1324	0.0607	0.4585
PB107186.00.0	Pv098905	0.1639	0.0477	0.2909
PB000320.02.0	Pv098825	0.0692	0.0452	0.6527
PB000846.00.0	Pv098765	0.1361	0.0572	0.4202
PB300677.00.0	Pv099180	0.0845	0.0594	0.7029
PB000790.02.0	Pv098695	0.0568	0.0311	0.5471
PB000373.01.0	Pv099105	0.0503	0.0287	0.5711
PB001127.02.0	Pv082470	0.0072	0.0085	1.1863
PB000573.02.0	Pv083490	0.0781	0.0530	0.6782
PB001172.01.0	Pv081330	0.1149	0.0478	0.4161
PB001224.00.0	Pv001950	0.0303	0.0292	0.9649
PB001216.00.0	Pv001740	0.0496	0.0313	0.6314
PB000727.02.0	Pv000715	0.0656	0.0675	1.0284
PB001259.02.0	Pv000765	0.1099	0.0814	0.7407
PB000149.00.0	Pv083250	0.0597	0.0319	0.5343
PB001094.00.0	Pv096360	0.0055	0.0053	0.9564
PB000499.02.0	Pv083200	0.1519	0.0656	0.4320
PB000888.03.0	Pv093640	0.0216	0.0222	1.0316
PB001635.02.0	Pv000925	0.0415	0.0253	0.6098
PB001043.01.0	Pv093575	0.0191	0.0286	1.5012
PB000711.02.0	Pv000535	0.1239	0.0469	0.3789
PB001543.02.0	Pv001075	0.0785	0.0414	0.5270
PB000874.03.0	Pv084770	0.0214	0.0165	0.7695
PB000590.02.0	Pv084805	0.0302	0.0217	0.7174
PB000666.02.0	Pv085460	0.0627	0.0446	0.7110
PB000392.03.0	Pv085575	0.0400	0.0251	0.6274
PB000310.02.0	Pv111495	0.0810	0.0671	0.8285
PB000496.03.0	Pv111455	0.0180	0.0164	0.9088
PB000700.00.0	Pv081570	0.0114	0.0047	0.4123
PB000715.01.0	Pv084995	0.0207	0.0219	1.0587
PB001201.00.0	Pv085025	0.0298	0.0301	1.0114
PB000566.01.0	Pv084695	0.0767	0.0406	0.5291
PB001200.00.0	Pv084750	0.1723	0.0746	0.4329
PB001416.02.0	Pv117075	0.0881	0.0523	0.5934
PB000668.02.0	Pv094335	0.0766	0.0408	0.5325
PB000032.01.0	Pv094385	0.0434	0.0443	1.0211
PB000755.01.0	Pv117385	0.1204	0.0573	0.4760
PB001025.02.0	Pv117690	0.0670	0.0336	0.5016
PB001067.01.0	Pv117980	0.0133	0.0126	0.9481
PB000013.00.0	Pv079845	0.0171	0.0127	0.7385
PB000503.03.0	Pv080550	0.0088	0.0189	2.1530
PB001194.02.0	Pv123495	0.0396	0.0131	0.3298

PB000124.01.0	Pv123640	0.1307	0.0403	0.3083
PB001128.00.0	Pv123630	0.0151	0.0073	0.4829
PB000121.01.0	Pv094950	0.0086	0.0046	0.5367
PB000120.01.0	Pv095035	0.1253	0.0732	0.5845
PB000359.02.0	Pv095350	0.0000	0.0000	0.1812
PB001146.02.0	Pv119695	0.1115	0.0408	0.3659
PB000260.00.0	Pv119740	0.0000	0.0000	0.4108
PB000106.00.0	Pv002970	0.0900	0.0229	0.2545
PB000073.03.0	Pv122245	0.0000	0.0000	0.1251
PB000389.01.0	Pv122620	0.0497	0.0471	0.9485
PB000839.00.0	Pv123115	0.0836	0.0264	0.3162
PB000860.02.0	Pv122850	0.0674	0.0213	0.3160
PB000903.01.0	Pv091585	0.0668	0.0686	1.0266
PB000918.01.0	Pv090845	0.0480	0.0114	0.2383
PB001565.02.0	Pv087155	0.1768	0.0615	0.3480
PB000533.00.0	Pv115255	0.0000	0.0000	0.1063
PB300486.00.0	Pv091850	0.0674	0.0325	0.4829
PB000378.03.0	Pv092290	0.1422	0.0652	0.4583
PB000003.03.0	Pv091330	0.0235	0.0139	0.5938
PB001002.01.0	Pv091100	0.0468	0.0286	0.6107
PB001112.00.0	Pv092140	0.0382	0.0380	0.9960
PB000968.02.0	Pv100560	0.1521	0.0668	0.4391
PB000450.01.0	Pv100645	0.0434	0.0254	0.5859
PB001294.00.0	Pv100695	0.1426	0.0676	0.4738
PB000291.01.0	Pv113265	0.0864	0.0503	0.5816
PB000400.02.0	Pv113925	0.0742	0.0518	0.6977
PB001578.02.0	Pv099840	0.0392	0.0453	1.1561
**Orthologs of membrane genes**				
PB000731.00.0	Pv099370	0.0867	0.0248	0.2865
PB001055.00.0	Pv098845	0.0841	0.0445	0.5297
PB000042.02.0	Pv090140	0.1077	0.0391	0.3628
PB001526.02.0	Pv083240	0.2166	0.1225	0.5654
PB001222.00.0	Pv085905	0.2070	0.0673	0.3249
PB000977.02.0	Pv086080	0.0743	0.0476	0.6403
PB000020.02.0	Pv086270	0.2661	0.1071	0.4025
PB301230.00.0	Pv084630	0.0682	0.0334	0.4900
PB000859.03.0	Pv118305	0.0097	0.0023	0.2406
PB000652.01.0	Pv118650	0.1341	0.0397	0.2964
PB000776.02.0	Pv118510	0.0146	0.0058	0.3949
PB001026.02.0	Pv117695	0.1816	0.0667	0.3675
PB001609.02.0	Pv117210	0.1007	0.0445	0.4415
PB000201.02.0	Pv080425	0.0458	0.0352	0.7693
PB300930.00.0	Pv080590	0.0971	0.0506	0.5216
PB000768.01.0	Pv123365	0.0881	0.0255	0.2893
PB000809.01.0	Pv079895	0.0673	0.0431	0.6409
PB000834.00.0	Pv122910	0.1875	0.0336	0.1793
PB001386.02.0	Pv119570	0.0851	0.0655	0.7692
PB000125.00.0	Pv113340	0.0425	0.0190	0.4466
PB000616.00.0	Pv101400	0.0759	0.0702	0.9242
PB000962.02.0	Pv114410	0.0750	0.0378	0.5040

Pv/Pk Table C: dn/ds, dn,ds for orthologs of P. berghei expressed in all 3 stages

P.berghei gene	P.vivax ortholog	dn/ds	dn	ds
Orthologs of non-membrane genes				
PB300823.00.0	Pv087950	0.0048	0.0065	1.3407
PB000242.00.0	Pv087970	0.0358	0.0442	1.2368
PB001129.00.0	Pv088225	0.0214	0.0271	1.2698
PB001208.00.0	Pv088180	-1.0000	0.0122	-1.0000
PB000282.03.0	Pv088150	0.0471	0.0474	1.0052
PB000281.03.0	Pv088145	0.0120	0.0195	1.6288
PB000110.02.0	Pv099710	0.0244	0.0206	0.8434
PB000272.03.0	Pv003945	0.0172	0.0082	0.4787
PB001037.00.0	Pv099765	0.0942	0.0765	0.8121
PB001573.02.0	Pv003730	0.0382	0.0376	0.9823
PB000440.00.0	Pv099455	0.0116	0.0126	1.0915
PB000019.03.0	Pv098945	0.0165	0.0162	0.9802
PB000557.01.0	Pv089640	0.0103	0.0109	1.0535
PB000900.00.0	Pv089505	0.0053	0.0024	0.4585
PB000817.02.0	Pv089425	0.0084	0.0059	0.7022
PB000242.02.0	Pv089025	0.0057	0.0029	0.5095
PB000349.02.0	Pv089280	0.0150	0.0121	0.8088
PB001298.00.0	Pv088960	0.0179	0.0158	0.8864
PB000222.02.0	Pv089235	0.0068	0.0093	1.3716
PB000007.00.0	Pv089205	0.0571	0.0455	0.7954
PB000915.02.0	Pv096265	0.0055	0.0033	0.6008
PB000680.00.0	Pv096235	0.0344	0.0305	0.8853
PB001284.02.0	Pv099365	0.0143	0.0192	1.3402
PB000415.02.0	Pv089950	0.0504	0.0283	0.5611
PB001562.02.0	Pv099925	0.0364	0.0466	1.2797
PB000359.01.0	Pv099335	0.0509	0.0191	0.3755
PB000321.02.0	Pv098830	0.0263	0.0308	1.1707
PB000251.02.0	Pv090070	0.0513	0.0294	0.5738
PB001161.00.0	Pv099605	0.1450	0.0752	0.5187
PB001157.00.0	Pv099600	0.0622	0.0565	0.9084
PB000520.00.0	Pv099200	0.0207	0.0189	0.9109
PB000998.02.0	Pv090160	0.0210	0.0114	0.5432
PB000318.00.0	Pv099535	0.0429	0.0299	0.6960
PB001445.02.0	Pv099080	0.0130	0.0123	0.9496
PB000857.00.0	Pv098630	0.0000	0.0000	0.2911
PB001045.00.0	Pv098615	0.1123	0.0457	0.4070
PB300822.00.0	Pv098605	0.0446	0.0084	0.1880
PB000049.03.0	Pv082845	0.0421	0.0393	0.9326
PB000686.02.0	Pv082840	0.0998	0.0426	0.4262
PB000684.02.0	Pv082835	0.0255	0.0223	0.8739
PB001583.02.0	Pv083400	0.0439	0.0269	0.6136
PB001521.02.0	Pv083465	0.0103	0.0038	0.3724
PB001520.02.0	Pv083470	0.0418	0.0305	0.7295
PB000148.02.0	Pv001835	0.0000	0.0000	0.3456
PB000446.01.0	Pv001905	0.0000	0.0000	0.8851
PB000632.03.0	Pv001945	0.0559	0.0380	0.6800
PB000374.02.0	Pv081375	0.0299	0.0259	0.8658
PB001167.00.0	Pv001760	0.0361	0.0549	1.5212
PB000519.01.0	Pv081430	0.0700	0.0192	0.2744

PB000642.01.0	Pv083270	0.0238	0.0144	0.6059
PB001410.02.0	Pv096335	0.0052	0.0047	0.9117
PB001409.02.0	Pv096340	0.0029	0.0042	1.4605
PB000812.02.0	Pv083215	0.0694	0.0206	0.2963
PB000917.03.0	Pv083185	0.0207	0.0176	0.8498
PB001177.02.0	Pv000935	0.0093	0.0130	1.3994
PB000874.02.0	Pv093555	0.0307	0.0318	1.0359
PB000663.03.0	Pv083020	0.0203	0.0270	1.3289
PB001120.02.0	Pv083005	0.0268	0.0252	0.9433
PB000942.03.0	Pv083000	0.1168	0.0451	0.3862
PB000169.03.0	Pv082965	0.0658	0.0269	0.4093
PB000709.02.0	Pv000545	0.0343	0.0235	0.6851
PB000600.00.0	Pv000555	0.0116	0.0098	0.8510
PB001139.01.0	Pv000590	0.0307	0.0392	1.2747
PB000315.01.0	Pv084310	0.0379	0.0136	0.3587
PB000993.03.0	Pv085470	0.1051	0.0482	0.4582
PB001317.02.0	Pv085490	0.0415	0.0344	0.8294
PB300230.00.0	Pv085625	0.0307	0.0283	0.9213
PB000066.01.0	Pv085645	0.0419	0.0370	0.8842
PB000617.02.0	Pv085710	0.0561	0.0245	0.4364
PB000761.03.0	Pv085735	0.0043	0.0021	0.4753
PB000429.03.0	Pv085840	0.0250	0.0125	0.4991
PB000776.00.0	Pv085920	0.0073	0.0032	0.4378
PB001285.00.0	Pv085960	0.0259	0.0116	0.4482
PB001143.02.0	Pv086020	0.0000	0.0000	0.4449
PB000102.01.0	Pv085730	0.0000	0.0000	0.2680
PB000709.01.0	Pv086155	0.0108	0.0089	0.8258
PB000892.02.0	Pv081265	0.0314	0.0371	1.1816
PB001089.02.0	Pv084345	0.0272	0.0235	0.8669
PB000909.03.0	Pv084820	0.0391	0.0368	0.9408
PB000925.00.0	Pv084835	0.0803	0.0387	0.4817
PB001663.02.0	Pv085215	0.0073	0.0055	0.7633
PB001002.03.0	Pv085220	0.0026	0.0062	2.3606
PB000174.01.0	Pv081530	0.0507	0.0355	0.6997
PB000126.01.0	Pv085270	0.0793	0.0289	0.3639
PB000842.03.0	Pv084940	0.0742	0.0669	0.9020
PB000282.02.0	Pv084955	0.0282	0.0181	0.6428
PB000914.03.0	Pv081610	0.0788	0.0518	0.6581
PB000234.03.0	Pv084135	0.0384	0.0141	0.3668
PB000705.00.0	Pv081675	0.0481	0.0200	0.4153
PB001264.00.0	Pv081705	0.0158	0.0070	0.4419
PB000540.03.0	Pv084190	0.0140	0.0092	0.6581
PB001040.00.0	Pv084650	0.0137	0.0084	0.6129
PB000303.02.0	Pv084230	0.0135	0.0071	0.5216
PB000465.03.0	Pv084255	0.0065	0.0064	0.9857
PB001005.02.0	Pv084735	0.0433	0.0306	0.7072
PB000800.00.0	Pv117925	0.0080	0.0039	0.4917
PB000126.03.0	Pv117030	0.0257	0.0075	0.2914
PB000621.00.0	Pv116925	0.0372	0.0192	0.5163
PB000178.03.0	Pv116640	0.0286	0.0413	1.4438
PB000183.00.0	Pv116620	0.0209	0.0301	1.4376
PB000757.02.0	Pv118255	0.0848	0.0185	0.2187
PB000867.01.0	Pv118620	0.1854	0.0477	0.2574

PB001028.00.0	Pv118375	0.1059	0.0335	0.3166
PB001100.02.0	Pv118420	0.0471	0.0250	0.5298
PB001103.02.0	Pv118430	0.0000	0.0000	0.3368
PB001163.00.0	Pv118500	0.0479	0.0399	0.8345
PB000360.03.0	Pv094400	0.0192	0.0115	0.6010
PB000037.01.0	Pv118545	0.0713	0.0203	0.2853
PB000415.00.0	Pv111380	0.0152	0.0078	0.5142
PB000756.01.0	Pv117390	0.0000	0.0000	0.1647
PB001310.02.0	Pv117790	0.0341	0.0256	0.7490
PB000578.03.0	Pv111245	0.1055	0.0446	0.4224
PB000863.03.0	Pv118180	0.0387	0.0318	0.8219
PB000629.00.0	Pv117745	0.0629	0.0278	0.4428
PB001359.02.0	Pv118145	0.0106	0.0049	0.4638
PB300802.00.0	Pv117225	0.0182	0.0078	0.4265
PB000593.00.0	Pv117605	0.0312	0.0176	0.5652
PB000822.02.0	Pv117170	0.0131	0.0054	0.4134
PB000751.01.0	Pv117150	0.0208	0.0190	0.9133
PB001420.02.0	Pv111055	0.0695	0.0502	0.7230
PB000705.02.0	Pv110980	0.0227	0.0299	1.3185
PB000857.02.0	Pv095480	0.0149	0.0071	0.4814
PB001067.00.0	Pv080245	0.0376	0.0114	0.3030
PB001107.01.0	Pv080405	0.0243	0.0089	0.3681
PB000178.02.0	Pv080440	0.0233	0.0085	0.3661
PB000913.01.0	Pv080685	0.0397	0.0065	0.1639
PB000500.03.0	Pv080575	0.0146	0.0093	0.6379
PB001103.00.0	Pv124195	0.0337	0.0064	0.1892
PB000649.02.0	Pv124160	0.0636	0.0174	0.2738
PB000346.03.0	Pv124100	0.0262	0.0333	1.2711
PB001286.00.0	Pv123845	0.0252	0.0258	1.0271
PB000372.03.0	Pv124095	0.1908	0.1094	0.5732
PB000878.03.0	Pv123785	0.0695	0.0492	0.7082
PB001545.02.0	Pv123435	0.0743	0.0269	0.3620
PB000053.00.0	Pv123745	0.0287	0.0206	0.7170
PB001198.02.0	Pv123380	0.0259	0.0146	0.5647
PB001043.03.0	Pv123960	0.0086	0.0063	0.7355
PB000555.01.0	Pv123340	0.0657	0.0304	0.4619
PB001056.01.0	Pv123945	0.0044	0.0045	1.0074
PB000204.03.0	Pv123920	0.0607	0.0498	0.8204
PB001126.00.0	Pv123620	0.0354	0.0226	0.6383
PB000406.00.0	Pv123595	0.0207	0.0098	0.4726
PB000878.02.0	Pv094505	0.0000	0.0000	0.2285
PB000960.00.0	Pv094980	0.0265	0.0180	0.6793
PB001345.02.0	Pv095390	0.0181	0.0132	0.7299
PB000246.02.0	Pv080200	0.0422	0.0217	0.5129
PB000012.02.0	Pv094535	0.1266	0.0592	0.4674
PB000879.01.0	Pv095000	0.0217	0.0091	0.4181
PB000058.02.0	Pv095010	0.0142	0.0079	0.5592
PB000456.03.0	Pv095015	0.0317	0.0174	0.5496
PB000672.03.0	Pv095380	0.0345	0.0376	1.0909
PB000799.03.0	Pv094590	0.0310	0.0266	0.8556
PB001643.02.0	Pv095080	0.0234	0.0088	0.3781
PB000601.02.0	Pv094615	0.0000	0.0000	0.3825
PB000164.02.0	Pv094635	0.0000	0.0000	0.3526

PB001437.02.0	Pv094660	0.0248	0.0089	0.3596
PB001124.02.0	Pv095135	0.0093	0.0037	0.4017
PB001230.00.0	Pv080050	0.0093	0.0085	0.9126
PB001096.02.0	Pv095190	0.0000	0.0000	0.1556
PB000187.03.0	Pv079990	0.0522	0.0377	0.7221
PB000069.00.0	Pv095220	0.0056	0.0061	1.1029
PB000776.01.0	Pv094790	0.0332	0.0124	0.3727
PB000924.03.0	Pv094810	0.0482	0.0440	0.9137
PB000958.03.0	Pv095255	0.0978	0.0335	0.3430
PB000639.00.0	Pv080365	0.0140	0.0223	1.5917
PB001079.03.0	Pv080330	0.0354	0.0320	0.9034
PB000652.02.0	Pv079865	0.0875	0.0341	0.3897
PB001303.00.0	Pv094840	0.0304	0.0160	0.5274
PB000253.01.0	Pv094850	0.0623	0.0290	0.4660
PB000511.03.0	Pv080275	0.0326	0.0111	0.3403
PB000194.03.0	Pv122285	0.0468	0.0158	0.3370
PB000289.00.0	Pv119720	0.0402	0.0583	1.4517
PB001005.01.0	Pv002640	0.0163	0.0078	0.4777
PB000158.03.0	Pv002665	0.0088	0.0120	1.3539
PB000824.00.0	Pv002685	0.0074	0.0114	1.5511
PB000191.00.0	Pv002835	0.0302	0.0294	0.9721
PB000584.03.0	Pv002940	0.0549	0.0422	0.7698
PB000541.02.0	Pv097915	0.0385	0.0343	0.8907
PB000057.03.0	Pv123240	0.0627	0.0394	0.6294
PB001087.02.0	Pv122710	0.0067	0.0036	0.5336
PB000291.00.0	Pv122240	0.0337	0.0193	0.5724
PB000957.01.0	Pv123200	0.0378	0.0170	0.4494
PB000507.01.0	Pv119275	0.0603	0.0394	0.6540
PB001062.00.0	Pv123150	0.0112	0.0058	0.5145
PB001185.00.0	Pv119335	0.0241	0.0112	0.4650
PB000594.03.0	Pv123105	0.1132	0.0589	0.5208
PB000222.03.0	Pv123075	0.0000	0.0000	0.5892
PB000221.03.0	Pv123070	0.0977	0.0375	0.3836
PB000862.00.0	Pv123060	0.0623	0.0226	0.3632
PB000490.02.0	Pv123030	0.0303	0.0156	0.5135
PB000751.02.0	Pv119440	0.1963	0.0314	0.1601
PB000354.02.0	Pv119465	0.0116	0.0130	1.1194
PB001184.00.0	Pv119470	0.0000	0.0000	0.7465
PB001183.00.0	Pv119475	0.0193	0.0074	0.3848
PB000079.01.0	Pv122470	0.1347	0.0623	0.4628
PB000180.03.0	Pv121980	0.0369	0.0168	0.4540
PB001182.00.0	Pv119480	0.0224	0.0162	0.7217
PB000844.02.0	Pv122430	0.0096	0.0051	0.5310
PB000831.01.0	Pv119530	0.0163	0.0173	1.0573
PB000521.01.0	Pv119585	0.0236	0.0186	0.7861
PB000857.01.0	Pv091515	0.0000	0.0000	0.9974
PB000524.01.0	Pv091545	0.0509	0.0390	0.7664
PB000139.01.0	Pv091640	0.0270	0.0207	0.7661
PB000212.00.0	Pv090975	0.0177	0.0152	0.8574
PB000920.03.0	Pv090950	0.0133	0.0033	0.2496
PB000332.00.0	Pv087115	0.0572	0.0203	0.3554
PB000676.03.0	Pv114910	0.0000	0.0000	0.1034
PB000738.02.0	Pv115000	0.0384	0.0289	0.7542

PB000066.02.0	Pv115285	0.0164	0.0155	0.9456
PB000394.00.0	Pv091810	0.0408	0.0182	0.4455
PB001037.03.0	Pv092215	0.0121	0.0087	0.7239
PB001074.01.0	Pv092310	0.0171	0.0072	0.4202
PB001055.02.0	Pv092730	0.1338	0.0455	0.3401
PB000240.03.0	Pv091315	0.0432	0.0297	0.6869
PB001300.00.0	Pv091920	0.0383	0.0393	1.0257
PB000560.01.0	Pv091925	0.0138	0.0041	0.2992
PB000999.00.0	Pv092805	0.1942	0.0346	0.1781
PB300286.00.0	Pv092850	0.0119	0.0041	0.3417
PB000342.02.0	Pv091970	0.0122	0.0091	0.7427
PB001586.02.0	Pv092430	0.0379	0.0467	1.2338
PB001587.02.0	Pv092435	0.0848	0.0495	0.5836
PB000088.03.0	Pv092030	0.0205	0.0140	0.6852
PB000970.00.0	Pv092070	0.2596	0.1471	0.5666
PB000103.02.0	Pv092540	0.1099	0.0580	0.5279
PB000162.01.0	Pv091485	0.0122	0.0325	2.6605
PB000393.01.0	Pv092115	0.0402	0.0242	0.6025
PB000391.01.0	Pv092120	0.0481	0.0307	0.6380
PB000512.03.0	Pv092125	0.0097	0.0118	1.2225
PB000458.03.0	Pv114490	0.0364	0.0216	0.5933
PB000966.02.0	Pv100555	0.0584	0.0407	0.6958
PB000894.02.0	Pv100730	0.0395	0.0380	0.9634
PB000896.02.0	Pv100735	0.0144	0.0163	1.1334
PB001116.03.0	Pv100890	0.0539	0.0427	0.7915
PB000038.03.0	Pv100985	0.0143	0.0129	0.9063
PB000303.03.0	Pv101035	0.0000	0.0000	0.3304
PB000300.03.0	Pv101040	0.0134	0.0064	0.4794
PB000248.00.0	Pv101335	0.0128	0.0177	1.3780
PB301525.00.0	Pv101260	0.0729	0.0511	0.7009
PB000323.01.0	Pv101200	0.0000	0.0000	0.3721
PB000171.02.0	Pv114095	0.0093	0.0045	0.4819
PB001515.02.0	Pv114555	0.0781	0.0365	0.4672
PB001252.02.0	Pv113675	0.0507	0.0394	0.7775
PB000702.03.0	Pv114680	0.0172	0.0067	0.3885
PB000618.00.0	Pv113600	0.0426	0.0205	0.4802
PB001086.01.0	Pv113595	0.0148	0.0223	1.5015
PB001404.02.0	Pv113585	0.0116	0.0097	0.8340
PB000393.02.0	Pv114685	0.0131	0.0036	0.2743
PB001073.02.0	Pv114710	0.0506	0.0332	0.6554
PB000672.01.0	Pv113860	0.0137	0.0064	0.4634
PB000727.00.0	Pv114315	0.0200	0.0055	0.2726
PB000796.02.0	Pv113510	0.1440	0.1076	0.7475
PB000269.02.0	Pv113500	0.0510	0.0451	0.8848
PB000423.03.0	Pv114830	0.0031	0.0029	0.9333
PB001077.00.0	Pv114865	0.0135	0.0102	0.7582
PB000923.00.0	Pv113420	0.0395	0.0247	0.6249
PB000967.01.0	Pv114040	0.0221	0.0135	0.6078
PB000128.01.0	Pv114050	0.1416	0.0788	0.5567
PB000521.02.0	Pv114445	0.0220	0.0115	0.5246
PB001025.01.0	Pv114480	0.0973	0.0200	0.2060
PB001258.02.0	Pv114905	0.1387	0.0380	0.2737

Orthologs of membrane genes

PB001210.00.0	Pv088170	0.0602	0.0220	0.3650
PB000562.01.0	Pv003665	0.0303	0.0277	0.9136
PB000556.01.0	Pv089635	0.0000	0.0000	1.1759
PB000370.02.0	Pv089165	0.0394	0.0235	0.5954
PB001177.00.0	Pv099315	0.0087	0.0069	0.7999
PB000785.02.0	Pv098685	0.0399	0.0231	0.5791
PB000262.01.0	Pv083515	0.0490	0.0286	0.5826
PB000675.00.0	Pv081455	0.0273	0.0284	1.0396
PB000265.03.0	Pv085420	0.0067	0.0074	1.1032
PB000298.03.0	Pv086040	0.0539	0.0305	0.5665
PB000636.03.0	Pv085275	0.0449	0.0474	1.0551
PB000394.02.0	Pv084625	0.0260	0.0271	1.0404
PB000390.00.0	Pv116915	0.0885	0.0341	0.3852
PB000185.00.0	Pv116630	0.0178	0.0217	1.2143
PB001072.01.0	Pv110895	0.0251	0.0092	0.3650
PB000015.00.0	Pv079840	0.0300	0.0197	0.6562
PB001110.01.0	Pv080400	0.0000	0.0000	0.6625
PB001082.02.0	Pv123570	0.1514	0.0688	0.4544
PB000856.03.0	Pv091465	0.0488	0.0509	1.0430
PB000982.00.0	Pv091425	0.0412	0.0145	0.3521
PB000003.00.0	Pv123210	0.1307	0.0459	0.3512
PB001022.02.0	Pv119445	0.0465	0.0478	1.0266
PB000843.02.0	Pv122425	0.0504	0.0363	0.7201
PB000405.02.0	Pv121885	0.2735	0.0728	0.2662
PB000861.02.0	Pv122840	0.0166	0.0028	0.1680
PB001066.03.0	Pv090900	0.0241	0.0224	0.9296
PB000330.03.0	Pv092315	0.0903	0.0273	0.3024
PB000275.02.0	Pv091145	0.0215	0.0063	0.2938
PB001003.01.0	Pv091105	0.0217	0.0197	0.9099
PB000058.03.0	Pv092065	0.0434	0.0405	0.9348
PB000925.02.0	Pv100835	0.0594	0.0458	0.7709
PB000924.02.0	Pv100845	0.0403	0.0404	1.0040
PB000926.02.0	Pv100855	0.0790	0.0472	0.5979
PB000189.03.0	Pv115430	0.2037	0.0920	0.4519

Pv/Pk Table D: dn/ds, dn,ds for orthologs of P. berghei asexual blood stage proteins

P.berghei gene	P.vivax ortholog	dn/ds	dn	ds
Orthologs of non-membrane genes				
PB000979.02.0	Pv088110	0.0456	0.0365	0.8015
PB000902.01.0	Pv003655	0.1320	0.0576	0.4364
PB000558.01.0	Pv003995	0.3594	0.0699	0.1943
PB000618.02.0	Pv089705	0.0029	0.0029	0.9845
PB000596.02.0	Pv089715	0.1145	0.0666	0.5818
PB000872.00.0	Pv089580	0.0120	0.0185	1.5371
PB000722.00.0	Pv089530	0.0173	0.0160	0.9204
PB001117.03.0	Pv089385	0.0830	0.0412	0.4972
PB000011.03.0	Pv096245	0.0219	0.0299	1.3643
PB000084.00.0	Pv096150	0.0675	0.0424	0.6290
PB000214.02.0	Pv099145	0.0000	0.0000	0.7947
PB001160.00.0	Pv099585	0.0229	0.0121	0.5277
PB000051.00.0	Pv099560	0.0226	0.0207	0.9176
PB001041.03.0	Pv098645	0.0742	0.0249	0.3359
PB001042.03.0	Pv098640	0.1244	0.0179	0.1435
PB000868.02.0	Pv090175	0.0046	0.0102	2.2156
PB000900.02.0	Pv083280	0.0933	0.0587	0.6295
PB000451.03.0	Pv082415	0.0660	0.0424	0.6422
PB000636.02.0	Pv081335	0.0866	0.0555	0.6409
PB000784.02.0	Pv001725	0.0472	0.0413	0.8752
PB000735.02.0	Pv000730	0.0287	0.0164	0.5706
PB001222.02.0	Pv083135	0.0821	0.0357	0.4353
PB000338.00.0	Pv083080	0.0139	0.0068	0.4920
PB001070.00.0	Pv083035	0.0287	0.0219	0.7630
PB001069.00.0	Pv083030	0.0344	0.0234	0.6803
PB000649.03.0	Pv082935	0.0929	0.0569	0.6126
PB000152.01.0	Pv000635	0.0999	0.0789	0.7904
PB000779.00.0	Pv085930	0.1331	0.0667	0.5008
PB001331.02.0	Pv086230	0.0336	0.0165	0.4902
PB000848.00.0	Pv085995	0.1178	0.0534	0.4532
PB000229.02.0	Pv111600	0.1685	0.0250	0.1486
PB001570.02.0	Pv086310	0.1926	0.0675	0.3503
PB000897.00.0	Pv111555	0.2618	0.0530	0.2025
PB000893.01.0	Pv086075	0.0317	0.0103	0.3256
PB000434.02.0	Pv086095	0.0256	0.0121	0.4730
PB000638.02.0	Pv086340	0.1500	0.0318	0.2118
PB000497.03.0	Pv111445	0.0987	0.0814	0.8253
PB000682.01.0	Pv085265	0.1806	0.0798	0.4419
PB000468.02.0	Pv084370	0.0346	0.0338	0.9757
PB001039.01.0	Pv081500	0.0544	0.0484	0.8895
PB000626.00.0	Pv085315	0.0222	0.0246	1.1103
PB000713.00.0	Pv085050	0.0151	0.0338	2.2337
PB001039.00.0	Pv084645	0.0534	0.0523	0.9802
PB301388.00.0	Pv085125	0.0142	0.0108	0.7628
PB000726.02.0	Pv084705	0.0158	0.0139	0.8759
PB000730.02.0	Pv084720	0.0720	0.0711	0.9877
PB000819.03.0	Pv117020	0.0423	0.0468	1.1067
PB000368.00.0	Pv116770	0.1210	0.0655	0.5410
PB000665.00.0	Pv116670	0.3576	0.1002	0.2801

PB000482.00.0	Pv118235	0.1224	0.0541	0.4420
PB000828.01.0	Pv094320	0.1276	0.0483	0.3788
PB000096.01.0	Pv094380	0.0380	0.0278	0.7311
PB000747.01.0	Pv111355	0.0994	0.0749	0.7537
PB000417.00.0	Pv111330	0.0224	0.0101	0.4517
PB000852.01.0	Pv117290	0.2989	0.0383	0.1280
PB000603.00.0	Pv111165	0.0133	0.0082	0.6185
PB001021.00.0	Pv117700	0.1498	0.0667	0.4451
PB001012.01.0	Pv117215	0.0657	0.0254	0.3862
PB001229.02.0	Pv111025	0.0473	0.0386	0.8167
PB001068.01.0	Pv117985	0.0000	0.0000	0.4169
PB300407.00.0	Pv080630	0.0671	0.0432	0.6440
PB000310.01.0	Pv124185	0.1395	0.0441	0.3162
PB001149.01.0	Pv124175	0.0461	0.0412	0.8934
PB000668.01.0	Pv124060	0.1401	0.0665	0.4750
PB000042.01.0	Pv123740	0.0964	0.0623	0.6459
PB001623.02.0	Pv123655	0.1122	0.0569	0.5075
PB000567.03.0	Pv095420	0.0691	0.0334	0.4837
PB000533.03.0	Pv080160	0.0710	0.0376	0.5292
PB000470.01.0	Pv094640	0.0198	0.0114	0.5784
PB001435.02.0	Pv094665	0.0930	0.0328	0.3522
PB300187.00.0	Pv094695	0.0271	0.0117	0.4328
PB000011.01.0	Pv094730	0.0967	0.0520	0.5375
PB000059.03.0	Pv095160	0.0560	0.0665	1.1888
PB001111.01.0	Pv080385	0.0732	0.0503	0.6864
PB000207.03.0	Pv079955	0.0734	0.0296	0.4037
PB000639.03.0	Pv094865	0.0143	0.0102	0.7137
PB000091.03.0	Pv002725	0.2471	0.1076	0.4355
PB000470.03.0	Pv002785	0.1016	0.0816	0.8035
PB000472.03.0	Pv002790	0.0918	0.0443	0.4824
PB000190.00.0	Pv002830	0.0486	0.0445	0.9163
PB001632.02.0	Pv097940	0.0233	0.0125	0.5363
PB000219.02.0	Pv091450	0.0257	0.0210	0.8186
PB000722.02.0	Pv091435	0.0610	0.0530	0.8688
PB000198.02.0	Pv123190	0.1122	0.0722	0.6437
PB001378.02.0	Pv122690	0.1855	0.0847	0.4565
PB000019.02.0	Pv123145	0.1379	0.0598	0.4339
PB001121.02.0	Pv122030	0.2311	0.0948	0.4100
PB000749.02.0	Pv119435	0.1246	0.0372	0.2985
PB000695.01.0	Pv122950	0.1420	0.0628	0.4421
PB000615.00.0	Pv119520	0.0842	0.0546	0.6485
PB000833.01.0	Pv119545	0.0693	0.0730	1.0524
PB001627.02.0	Pv091550	0.0258	0.0245	0.9494
PB000097.03.0	Pv091655	0.0901	0.0416	0.4618
PB000669.02.0	Pv090865	0.0644	0.0302	0.4697
PB000862.03.0	Pv114940	0.0464	0.0251	0.5404
PB300308.00.0	Pv115235	0.0644	0.0268	0.4158
PB000532.02.0	Pv086975	0.0178	0.0230	1.2905
PB000004.00.0	Pv086960	0.0408	0.0502	1.2314
PB000739.02.0	Pv115010	0.0687	0.0409	0.5954
PB001372.02.0	Pv115015	0.0469	0.0398	0.8490
PB000065.02.0	Pv115290	0.0382	0.0279	0.7292
PB000569.00.0	Pv115340	0.0359	0.0216	0.6008

PB301484.00.0	Pv115370	0.0050	0.0070	1.4101
PB000887.00.0	Pv091910	0.4138	0.0890	0.2150
PB000473.03.0	Pv092365	0.1081	0.0750	0.6943
PB000772.02.0	Pv091185	0.0607	0.0255	0.4195
PB000833.03.0	Pv091965	0.0381	0.0693	1.8189
PB000973.03.0	Pv092040	0.0745	0.0562	0.7540
PB000459.03.0	Pv114495	0.0660	0.0261	0.3950
PB001011.01.0	Pv100535	0.0649	0.0446	0.6874
PB000206.00.0	Pv100625	0.0618	0.0576	0.9315
PB300224.00.0	Pv100795	0.0886	0.0780	0.8806
PB300427.00.0	Pv101345	0.0526	0.0366	0.6967
PB000549.03.0	Pv101085	0.0917	0.0550	0.5997
PB000193.03.0	Pv101215	0.0502	0.0251	0.5003
PB000192.03.0	Pv101220	0.1087	0.0720	0.6618
PB000523.03.0	Pv101235	0.0853	0.0361	0.4228
PB000522.03.0	Pv101240	0.1528	0.0704	0.4607
PB001175.01.0	Pv114105	0.1362	0.0897	0.6582
PB000526.00.0	Pv113785	0.0615	0.0350	0.5687
PB000895.03.0	Pv114210	0.0871	0.0632	0.7253
PB000093.00.0	Pv113830	0.0599	0.0578	0.9645
PB000467.02.0	Pv114375	0.1936	0.0902	0.4661
PB000489.03.0	Pv114000	0.0160	0.0308	1.9215
**Orthologs of membrane proteins**				
PB000619.02.0	Pv089700	0.1035	0.0172	0.1663
PB001406.02.0	Pv089310	0.0233	0.0245	1.0514
PB000172.01.0	Pv099980	0.1431	0.0830	0.5798
PB000442.03.0	Pv082680	0.2312	0.1047	0.4527
PB000345.03.0	Pv083420	0.0804	0.0715	0.8895
PB000944.03.0	Pv083480	0.1869	0.0767	0.4104
PB001179.02.0	Pv000945	0.2344	0.1011	0.4314
PB000963.01.0	Pv000995	0.1112	0.0662	0.5952
PB000294.03.0	Pv085520	0.0476	0.0252	0.5303
PB000435.02.0	Pv086090	0.0103	0.0054	0.5224
PB000910.03.0	Pv084815	0.0737	0.0438	0.5947
PB000519.03.0	Pv084445	0.1543	0.0780	0.5054
PB000392.02.0	Pv117005	0.0556	0.0472	0.8494
PB001439.02.0	Pv116695	0.0631	0.0576	0.9128
PB000011.00.0	Pv118295	0.0676	0.0238	0.3524
PB001106.00.0	Pv110905	0.0369	0.0243	0.6586
PB000379.02.0	Pv117880	0.1050	0.0522	0.4976
PB000282.01.0	Pv110810	0.5574	0.1632	0.2929
PB000505.02.0	Pv117685	0.1016	0.0403	0.3965
PB001654.02.0	Pv118100	0.0326	0.0256	0.7862
PB001115.00.0	Pv094885	0.1487	0.0875	0.5882
PB000995.02.0	Pv079800	0.0722	0.0418	0.5789
PB000489.00.0	Pv122810	0.0904	0.0436	0.4822
PB000443.01.0	Pv122755	0.0779	0.0328	0.4211
PB000072.03.0	Pv122250	0.1257	0.0684	0.5437
PB000683.03.0	Pv122145	0.0746	0.0322	0.4317
PB001081.01.0	Pv091790	0.0326	0.0467	1.4322
PB000162.00.0	Pv091615	0.0136	0.0188	1.3849
PB000362.00.0	Pv090960	0.0546	0.0603	1.1037
PB000821.01.0	Pv092275	0.0987	0.0587	0.5951

PB001171.02.0	Pv091175	0.0371	0.0342	0.9219
PB000986.00.0	Pv091155	0.1534	0.0661	0.4311
PB000992.02.0	Pv100710	0.0745	0.0370	0.4965
PB000909.00.0	Pv101485	0.1006	0.0522	0.5192
