# Supplementary material for: Why are male malaria parasites in such a rush? Sex-specific evolution and host–parasite interactions
Source: Evol Med Public Health. 2012 Nov 26;2013(1):3–13. doi: 10.1093/emph/eos003 (PMC4183958; doi:10.1093/emph/eos003)
Supplement: Supplementary Data [file supp_eos003_suppl_data.zip › REECE_Table_S2_PB.pdf]

Pb/Py Table A: dn/ds for stage-specific sets

Set	Number of genes	Mean dn/ds	CI 95%	CI 95%
Male	221	0.1946	0.1665	0.2307
Female	96	0.1460	0.1242	0.1695
Expressed in 3 stages	290	0.0902	0.0793	0.1016
Asexual blood stages	161	0.2315	0.2051	0.2599

Pb/Py Table B: dn/ds comparisons for stage-specific sets

Set 1	Mean dn/ds	Set 2	Mean dn/ds	Pvalue Set 1 > Set 2
Asexual blood stages	0.2315	Asexual blood stages	0.2315	0.5063
Asexual blood stages	0.2315	Male	0.1946	0.9177
Asexual blood stages	0.2315	Female	0.146	0.9999
Asexual blood stages	0.2315	Expressed in 3 stages	0.0902	1
Male	0.1946	Asexual blood stages	0.2315	0.0786
Male	0.1946	Male	0.1946	0.4888
Male	0.1946	Female	0.146	0.9868
Male	0.1946	Expressed in 3 stages	0.0902	1
Female	0.146	Asexual blood stages	0.2315	0
Female	0.146	Male	0.1946	0.0137
Female	0.146	Female	0.146	0.4975
Female	0.146	Expressed in 3 stages	0.0902	1
Expressed in 3 stages	0.0902	Asexual blood stages	0.2315	0
Expressed in 3 stages	0.0902	Male	0.1946	0
Expressed in 3 stages	0.0902	Female	0.146	0
Expressed in 3 stages	0.0902	Expressed in 3 stages	0.0902	0.5043

Pb/Py Table C: dn/ds for all sets genes with ds=0 excluded

Set	Number of genes	Mean dn/ds	CI 95%	CI 95%
Membrane male	29	0.2264	0.1648	0.2990
Male non membrane	192	0.1899	0.1592	0.2298
Membrane female	20	0.2192	0.1601	0.2815
Female non membrane	76	0.1267	0.1055	0.1505
Membrane expressed in 3 stages	33	0.1453	0.1051	0.1896
Expressed in 3 stages non membrane	257	0.0831	0.0727	0.0947
Membrane asexual	32	0.2845	0.2341	0.3377
Asexual non membrane	129	0.2183	0.1895	0.2504

Pb/Py Table D: dn/ds comparisons for all sets

Set 1	Mean dn/ds	Set 2	Mean dn/ds	Pvalue Set 1 > Set 2
asexual non membrane	0.2183	asexual non membrane	0.2183	0.5
asexual non membrane	0.2183	asexual membrane	0.2845	0.0343
asexual non membrane	0.2183	male non membrane	0.1899	0.845
asexual non membrane	0.2183	male membrane	0.2264	0.4491
asexual non membrane	0.2183	female non membrane	0.1267	1
asexual non membrane	0.2183	female membrane	0.2192	0.5051
asexual non membrane	0.2183	expressed in all 3 stages non membrane	0.0831	1
asexual non membrane	0.2183	expressed in all 3 stages membrane	0.1453	0.9866
asexual membrane	0.2845	asexual non membrane	0.2183	0.9665
asexual membrane	0.2845	asexual membrane	0.2845	0.4975
asexual membrane	0.2845	male non membrane	0.1899	0.9912
asexual membrane	0.2845	male membrane	0.2264	0.8697
asexual membrane	0.2845	female non membrane	0.1267	1
asexual membrane	0.2845	female membrane	0.2192	0.9096
asexual membrane	0.2845	expressed in all 3 stages non membrane	0.0831	1

asexual membrane	0.2845	expressed in all 3 stages membrane	0.1453	0.9992
male non membrane	0.1899	asexual non membrane	0.2183	0.1586
male non membrane	0.1899	asexual membrane	0.2845	0.0093
male non membrane	0.1899	male non membrane	0.1899	0.5012
male non membrane	0.1899	male membrane	0.2264	0.2153
male non membrane	0.1899	female non membrane	0.1267	0.9965
male non membrane	0.1899	female membrane	0.2192	0.2483
male non membrane	0.1899	expressed in all 3 stages non membrane	0.0831	1
male non membrane	0.1899	expressed in all 3 stages membrane	0.1453	0.9095
male membrane	0.2264	asexual non membrane	0.2183	0.5575
male membrane	0.2264	asexual membrane	0.2845	0.1326
male membrane	0.2264	male non membrane	0.1899	0.7792
male membrane	0.2264	male membrane	0.2264	0.4928
male membrane	0.2264	female non membrane	0.1267	0.9965
male membrane	0.2264	female membrane	0.2192	0.5452
male membrane	0.2264	expressed in all 3 stages non membrane	0.0831	1
male membrane	0.2264	expressed in all 3 stages membrane	0.1453	0.9586
female non membrane	0.1267	asexual non membrane	0.2183	0
female non membrane	0.1267	asexual membrane	0.2845	0
female non membrane	0.1267	male non membrane	0.1899	0.0029
female non membrane	0.1267	male membrane	0.2264	0.0028
female non membrane	0.1267	female non membrane	0.1267	0.4962
female non membrane	0.1267	female membrane	0.2192	0.0055
female non membrane	0.1267	expressed in all 3 stages non membrane	0.0831	0.9989
female non membrane	0.1267	expressed in all 3 stages membrane	0.1453	0.2658
female membrane	0.2192	asexual non membrane	0.2183	0.5012
female membrane	0.2192	asexual membrane	0.2845	0.092
female membrane	0.2192	male non membrane	0.1899	0.7578
female membrane	0.2192	male membrane	0.2264	0.4552
female membrane	0.2192	female non membrane	0.1267	0.9951
female membrane	0.2192	female membrane	0.2192	0.4969
female membrane	0.2192	expressed in all 3 stages non membrane	0.0831	0.9999
female membrane	0.2192	expressed in all 3 stages membrane	0.1453	0.9516
expressed in all 3 stages non membrane	0.0831	asexual non membrane	0.2183	0
expressed in all 3 stages non membrane	0.0831	asexual membrane	0.2845	0
expressed in all 3 stages non membrane	0.0831	male non membrane	0.1899	0

expressed in all 3 stages non membrane	0.0831	male membrane	0.2264	0
expressed in all 3 stages non membrane	0.0831	female non membrane	0.1267	0.0013
expressed in all 3 stages non membrane	0.0831	female membrane	0.2192	0
expressed in all 3 stages non membrane	0.0831	expressed in all 3 stages non membrane	0.0831	0.4927
expressed in all 3 stages non membrane	0.0831	expressed in all 3 stages membrane	0.1453	0.0056
expressed in all 3 stages membrane	0.1453	asexual non membrane	0.2183	0.0152
expressed in all 3 stages membrane	0.1453	asexual membrane	0.2845	0.0004
expressed in all 3 stages membrane	0.1453	male non membrane	0.1899	0.0953
expressed in all 3 stages membrane	0.1453	male membrane	0.2264	0.0452
expressed in all 3 stages membrane	0.1453	female non membrane	0.1267	0.7308
expressed in all 3 stages membrane	0.1453	female membrane	0.2192	0.0494
expressed in all 3 stages membrane	0.1453	expressed in all 3 stages non membrane	0.0831	0.9938
expressed in all 3 stages membrane	0.1453	expressed in all 3 stages membrane	0.1453	0.4927

Pb/Py Table E: ds for all sets genes ds.=0 included

Set	Number of genes	Mean ds	CI 95%	CI 95%
Membrane male	29	0.1586	0.1364	0.1830
Male non membrane	192	0.1636	0.1528	0.1750
Membrane female	20	0.1423	0.1169	0.1709
Female non membrane	76	0.1630	0.1493	0.1771
Membrane expressed in 3 stages	33	0.1677	0.1470	0.1889
Expressed in 3 stages non memb	259	0.1682	0.1595	0.1770
Membrane asexual	32	0.1445	0.1286	0.1617
Asexual non membrane	129	0.1621	0.1497	0.1748

Pb/Py Table F: ds comparisons for all sets

Set 1	Mean ds	Set 2	Mean ds	Pvalue Set 1 > Set 2
asexual non membrane	0.1621	asexual non membrane	0.1621	0.5056
asexual non membrane	0.1621	asexual membrane	0.1445	0.9134
asexual non membrane	0.1621	male non membrane	0.1636	0.4533
asexual non membrane	0.1621	male membrane	0.1586	0.5923
asexual non membrane	0.1621	female non membrane	0.1630	0.4617
asexual non membrane	0.1621	female membrane	0.1422	0.8662
asexual non membrane	0.1621	expressed in all 3 stages non membrane	0.1682	0.2466
asexual non membrane	0.1621	expressed in all 3 stages membrane	0.1677	0.3548
asexual membrane	0.1445	asexual non membrane	0.1621	0.0849
asexual membrane	0.1445	asexual membrane	0.1445	0.4958
asexual membrane	0.1445	male non membrane	0.1636	0.0639
asexual membrane	0.1445	male membrane	0.1586	0.2124
asexual membrane	0.1445	female non membrane	0.1630	0.0868
asexual membrane	0.1445	female membrane	0.1422	0.5622

asexual membrane	0.1445	expressed in all 3 stages non membrane	0.1682	0.0219
asexual membrane	0.1445	expressed in all 3 stages membrane	0.1677	0.0726
male non membrane	0.1636	asexual non membrane	0.1621	0.5582
male non membrane	0.1636	asexual membrane	0.1445	0.9388
male non membrane	0.1636	male non membrane	0.1636	0.4973
male non membrane	0.1636	male membrane	0.1586	0.6261
male non membrane	0.1636	female non membrane	0.1630	0.5260
male non membrane	0.1636	female membrane	0.1422	0.8803
male non membrane	0.1636	expressed in all 3 stages non membrane	0.1682	0.2865
male non membrane	0.1636	expressed in all 3 stages membrane	0.1677	0.3921
male membrane	0.1586	asexual non membrane	0.1621	0.4210
male membrane	0.1586	asexual membrane	0.1445	0.7882
male membrane	0.1586	male non membrane	0.1636	0.3837
male membrane	0.1586	male membrane	0.1586	0.4937
male membrane	0.1586	female non membrane	0.1630	0.3940
male membrane	0.1586	female membrane	0.1422	0.7779
male membrane	0.1586	expressed in all 3 stages non membrane	0.1682	0.2678
male membrane	0.1586	expressed in all 3 stages membrane	0.1677	0.3133
female non membrane	0.1630	asexual non membrane	0.1621	0.5328
female non membrane	0.1630	asexual membrane	0.1445	0.9251
female non membrane	0.1630	male non membrane	0.1636	0.4757
female non membrane	0.1630	male membrane	0.1586	0.6033
female non membrane	0.1630	female non membrane	0.1630	0.4887
female non membrane	0.1630	female membrane	0.1422	0.8732
female non membrane	0.1630	expressed in all 3 stages non membrane	0.1682	0.2990
female non membrane	0.1630	expressed in all 3 stages membrane	0.1677	0.3887
female membrane	0.1422	asexual non membrane	0.1621	0.1387
female membrane	0.1422	asexual membrane	0.1445	0.4427
female membrane	0.1422	male non membrane	0.1636	0.1155
female membrane	0.1422	male membrane	0.1586	0.2207
female membrane	0.1422	female non membrane	0.1630	0.1250
female membrane	0.1422	female membrane	0.1422	0.4998
female membrane	0.1422	expressed in all 3 stages non membrane	0.1682	0.0720
female membrane	0.1422	expressed in all 3 stages membrane	0.1677	0.1096
expressed in all 3 stages non me	0.1682	asexual non membrane	0.1621	0.7429
expressed in all 3 stages non me	0.1682	asexual membrane	0.1445	0.9799

expressed in all 3 stages non me	0.1682	male non membrane	0.1636	0.7051
expressed in all 3 stages non me	0.1682	male membrane	0.1586	0.7394
expressed in all 3 stages non me	0.1682	female non membrane	0.1630	0.6983
expressed in all 3 stages non me	0.1682	female membrane	0.1422	0.9277
expressed in all 3 stages non me	0.1682	expressed in all 3 stages non membrane	0.1682	0.4967
expressed in all 3 stages non me	0.1682	expressed in all 3 stages membrane	0.1677	0.5152
expressed in all 3 stages membra	0.1677	asexual non membrane	0.1621	0.6490
expressed in all 3 stages membra	0.1677	asexual membrane	0.1445	0.9277
expressed in all 3 stages membra	0.1677	male non membrane	0.1636	0.6112
expressed in all 3 stages membra	0.1677	male membrane	0.1586	0.6829
expressed in all 3 stages membra	0.1677	female non membrane	0.1630	0.6027
expressed in all 3 stages membra	0.1677	female membrane	0.1422	0.8928
expressed in all 3 stages membra	0.1677	expressed in all 3 stages non membrane	0.1682	0.4827
expressed in all 3 stages membra	0.1677	expressed in all 3 stages membrane	0.1677	0.5124

Pb/Py Table G: dn for all sets

Set	Number of genes	Mean dn	CI 95%	CI 95%
Membrane male	29	0.032575862	0.024044828	0.042162241
Male non membrane	192	0.024810417	0.022354609	0.027508359
Membrane female	20	0.02626	0.019725	0.03352525
Female non membrane	76	0.017432895	0.014994737	0.019914605
Membrane expressed in 3 stages	33	0.019612121	0.015087727	0.024557576
Expressed in 3 stages non membrane	259	0.012743629	0.0111289	0.014533707
Membrane asexual blood stages	32	0.03743125	0.030959375	0.044081406
Asexual blood stages non membrane	129	0.029172093	0.025908488	0.032535814

Pb/Py Table H: dn comparisons for all sets

Set 1	Mean dn	Set 2	Mean dn	Pvalue Set 1 > Set 2
Asexual blood stages non membrane	0.0292	Asexual blood stages non membrane	0.0292	0.4871
Asexual blood stages non membrane	0.0292	Membrane asexual blood stages	0.0374	0.0353
Asexual blood stages non membrane	0.0292	Male non membrane	0.0248	0.9567
Asexual blood stages non membrane	0.0292	Membrane male	0.0326	0.2977
Asexual blood stages non membrane	0.0292	Female non membrane	0.0174	1
Asexual blood stages non membrane	0.0292	Membrane female	0.0263	0.7396
Asexual blood stages non membrane	0.0292	Expressed in 3 stages non membrane	0.0127	1
Asexual blood stages non membrane	0.0292	Membrane expressed in 3 stages	0.0196	0.9963
Membrane asexual blood stages	0.0374	Asexual blood stages non membrane	0.0292	0.9702
Membrane asexual blood stages	0.0374	Membrane asexual blood stages	0.0374	0.4924
Membrane asexual blood stages	0.0374	Male non membrane	0.0248	0.9995
Membrane asexual blood stages	0.0374	Membrane male	0.0326	0.7655
Membrane asexual blood stages	0.0374	Female non membrane	0.0174	1
Membrane asexual blood stages	0.0374	Membrane female	0.0263	0.9732

Membrane asexual blood stages	0.0374	Expressed in 3 stages non membrane	0.0127	1
Membrane asexual blood stages	0.0374	Membrane expressed in 3 stages	0.0196	0.9999
Male non membrane	0.0248	Asexual blood stages non membrane	0.0292	0.0475
Male non membrane	0.0248	Membrane asexual blood stages	0.0374	0.001
Male non membrane	0.0248	Male non membrane	0.0248	0.498
Male non membrane	0.0248	Membrane male	0.0326	0.0811
Male non membrane	0.0248	Female non membrane	0.0174	0.9999
Male non membrane	0.0248	Membrane female	0.0263	0.3816
Male non membrane	0.0248	Expressed in 3 stages non membrane	0.0127	1
Male non membrane	0.0248	Membrane expressed in 3 stages	0.0196	0.9384
Membrane male	0.0326	Asexual blood stages non membrane	0.0292	0.6967
Membrane male	0.0326	Membrane asexual blood stages	0.0374	0.2353
Membrane male	0.0326	Male non membrane	0.0248	0.9129
Membrane male	0.0326	Membrane male	0.0326	0.4951
Membrane male	0.0326	Female non membrane	0.0174	0.9995
Membrane male	0.0326	Membrane female	0.0263	0.8157
Membrane male	0.0326	Expressed in 3 stages non membrane	0.0127	1
Membrane male	0.0326	Membrane expressed in 3 stages	0.0196	0.9855
Female non membrane	0.0174	Asexual blood stages non membrane	0.0292	0
Female non membrane	0.0174	Membrane asexual blood stages	0.0374	0
Female non membrane	0.0174	Male non membrane	0.0248	0.0002
Female non membrane	0.0174	Membrane male	0.0326	0.0008
Female non membrane	0.0174	Female non membrane	0.0174	0.4941
Female non membrane	0.0174	Membrane female	0.0263	0.0201
Female non membrane	0.0174	Expressed in 3 stages non membrane	0.0127	0.9944
Female non membrane	0.0174	Membrane expressed in 3 stages	0.0196	0.2432
Membrane female	0.0263	Asexual blood stages non membrane	0.0292	0.2551
Membrane female	0.0263	Membrane asexual blood stages	0.0374	0.0297
Membrane female	0.0263	Male non membrane	0.0248	0.6085
Membrane female	0.0263	Membrane male	0.0326	0.1832
Membrane female	0.0263	Female non membrane	0.0174	0.9819
Membrane female	0.0263	Membrane female	0.0263	0.5003
Membrane female	0.0263	Expressed in 3 stages non membrane	0.0127	1
Membrane female	0.0263	Membrane expressed in 3 stages	0.0196	0.9073
Expressed in 3 stages non membrane	0.0127	Asexual blood stages non membrane	0.0292	0
Expressed in 3 stages non membrane	0.0127	Membrane asexual blood stages	0.0374	0

Expressed in 3 stages non membrane	0.0127	Male non membrane	0.0248	0
Expressed in 3 stages non membrane	0.0127	Membrane male	0.0326	0
Expressed in 3 stages non membrane	0.0127	Female non membrane	0.0174	0.0056
Expressed in 3 stages non membrane	0.0127	Membrane female	0.0263	0
Expressed in 3 stages non membrane	0.0127	Expressed in 3 stages non membrane	0.0127	0.498
Expressed in 3 stages non membrane	0.0127	Membrane expressed in 3 stages	0.0196	0.0101
Membrane expressed in 3 stages	0.0196	Asexual blood stages non membrane	0.0292	0.0043
Membrane expressed in 3 stages	0.0196	Membrane asexual blood stages	0.0374	0.0001
Membrane expressed in 3 stages	0.0196	Male non membrane	0.0248	0.0656
Membrane expressed in 3 stages	0.0196	Membrane male	0.0326	0.0134
Membrane expressed in 3 stages	0.0196	Female non membrane	0.0174	0.7576
Membrane expressed in 3 stages	0.0196	Membrane female	0.0263	0.0913
Membrane expressed in 3 stages	0.0196	Expressed in 3 stages non membrane	0.0127	0.9924
Membrane expressed in 3 stages	0.0196	Membrane expressed in 3 stages	0.0196	0.4922
