# Supplementary material for: Why are male malaria parasites in such a rush? Sex-specific evolution and host–parasite interactions
Source: Evol Med Public Health. 2012 Nov 26;2013(1):3–13. doi: 10.1093/emph/eos003 (PMC4183958; doi:10.1093/emph/eos003)
Supplement: Supplementary Data [file supp_eos003_suppl_data.zip › REECE_Table_S2_PF.pdf]

Pf/Pr Table A: dn/ds for stage-specific sets

Set	Number of genes	Mean dn/ds	CI 95%	CI 95%
Male	106	0.3294	0.2613	0.4128
Female	51	0.1973	0.1575	0.2397
Expressed in 3 stages	143	0.1365	0.1092	0.1689
Asexual blood stages	85	0.2996	0.2489	0.3529

Pf/Pr Table B: dn/ds comparisons for stage-specific sets

Set 1	Mean dn/ds	Set 2	Mean dn/ds	Pvalue Set 1 > Set 2
Asexual blood stages	0.2996	Asexual blood stages	0.2996	0.5046
Asexual blood stages	0.2996	Male	0.3294	0.3048
Asexual blood stages	0.2996	Female	0.1973	0.9945
Asexual blood stages	0.2996	Expressed in 3 stages	0.1365	1
Male	0.3294	Asexual blood stages	0.2996	0.6844
Male	0.3294	Male	0.3294	0.497
Male	0.3294	Female	0.1973	0.9978
Male	0.3294	Expressed in 3 stages	0.1365	1
Female	0.1973	Asexual blood stages	0.2996	0.0051
Female	0.1973	Male	0.3294	0.0012
Female	0.1973	Female	0.1973	0.4997
Female	0.1973	Expressed in 3 stages	0.1365	0.9776
Expressed in 3 stages	0.1365	Asexual blood stages	0.2996	0
Expressed in 3 stages	0.1365	Male	0.3294	0.0001
Expressed in 3 stages	0.1365	Female	0.1973	0.0259
Expressed in 3 stages	0.1365	Expressed in 3 stages	0.1365	0.4961

Pf/Pr Table C: dn/ds for all sets

Set	Number of genes	Mean dn/ds	CI 95%	CI 95%
Membrane male	13	0.1865	0.1344	0.2394
Male non membrane	93	0.3494	0.2733	0.4459
Membrane female	8	0.1013	0.0555	0.1572
Female non membrane	43	0.2152	0.1704	0.2630
Membrane expressed in 3 stages	12	0.2076	0.1110	0.3280
Expressed in 3 stages non membrane	131	0.1300	0.1015	0.1628
Membrane asexual	21	0.3319	0.2382	0.4340
Asexual non membrane	64	0.2890	0.2305	0.3524

Pf/Pr Table D: dn/ds comparisons for all sets

Set 1	Mean dn/ds	Set 2	Mean dn/ds	Pvalue Set 1 > Set 2
asexual non membrane	0.289	Asexual non membrane	0.289	0.5013
asexual non membrane	0.289	Membrane asexual	0.3319	0.2815
asexual non membrane	0.289	Male non membrane	0.3494	0.1754
asexual non membrane	0.289	Membrane male	0.1865	0.9869
asexual non membrane	0.289	Female non membrane	0.2152	0.9431
asexual non membrane	0.289	Membrane female	0.1013	0.9999
asexual non membrane	0.289	Expressed in 3 stages non membrane	0.13	1
asexual non membrane	0.289	Membrane expressed in 3 stages	0.2076	0.8516
asexual membrane	0.3319	Asexual non membrane	0.289	0.7227
asexual membrane	0.3319	Membrane asexual	0.3319	0.4924
asexual membrane	0.3319	Male non membrane	0.3494	0.4199
asexual membrane	0.3319	Membrane male	0.1865	0.9885
asexual membrane	0.3319	Female non membrane	0.2152	0.9706
asexual membrane	0.3319	Membrane female	0.1013	0.9999
asexual membrane	0.3319	Expressed in 3 stages non membrane	0.13	1

asexual membrane	0.3319	Membrane expressed in 3 stages	0.2076	0.9148
male non membrane	0.3494	Asexual non membrane	0.289	0.8256
male non membrane	0.3494	Membrane asexual	0.3319	0.5805
male non membrane	0.3494	Male non membrane	0.3494	0.4978
male non membrane	0.3494	Membrane male	0.1865	0.9997
male non membrane	0.3494	Female non membrane	0.2152	0.9945
male non membrane	0.3494	Membrane female	0.1013	1
male non membrane	0.3494	Expressed in 3 stages non membrane	0.13	1
male non membrane	0.3494	Membrane expressed in 3 stages	0.2076	0.9485
male membrane	0.1865	Asexual non membrane	0.289	0.0161
male membrane	0.1865	Membrane asexual	0.3319	0.0127
male membrane	0.1865	Male non membrane	0.3494	0.0006
male membrane	0.1865	Membrane male	0.1865	0.5028
male membrane	0.1865	Female non membrane	0.2152	0.2507
male membrane	0.1865	Membrane female	0.1013	0.9676
male membrane	0.1865	Expressed in 3 stages non membrane	0.13	0.9387
male membrane	0.1865	Membrane expressed in 3 stages	0.2076	0.4093
female non membrane	0.2152	Asexual non membrane	0.289	0.0497
female non membrane	0.2152	Membrane asexual	0.3319	0.0303
female non membrane	0.2152	Male non membrane	0.3494	0.0074
female non membrane	0.2152	Membrane male	0.1865	0.7515
female non membrane	0.2152	Female non membrane	0.2152	0.4908
female non membrane	0.2152	Membrane female	0.1013	0.9958
female non membrane	0.2152	Expressed in 3 stages non membrane	0.13	0.9948
female non membrane	0.2152	Membrane expressed in 3 stages	0.2076	0.5706
female membrane	0.1013	Asexual non membrane	0.289	0
female membrane	0.1013	Membrane asexual	0.3319	0.0001
female membrane	0.1013	Male non membrane	0.3494	0
female membrane	0.1013	Membrane male	0.1865	0.0321
female membrane	0.1013	Female non membrane	0.2152	0.0052
female membrane	0.1013	Membrane female	0.1013	0.4973
female membrane	0.1013	Expressed in 3 stages non membrane	0.13	0.208
female membrane	0.1013	Membrane expressed in 3 stages	0.2076	0.0604
expressed in all 3 stages non membrane	0.13	Asexual non membrane	0.289	0
expressed in all 3 stages non membrane	0.13	Membrane asexual	0.3319	0
expressed in all 3 stages non membrane	0.13	Male non membrane	0.3494	0

expressed in all 3 stages non membrane	0.13	Membrane male	0.1865	0.0634
expressed in all 3 stages non membrane	0.13	Female non membrane	0.2152	0.0056
expressed in all 3 stages non membrane	0.13	Membrane female	0.1013	0.7917
expressed in all 3 stages non membrane	0.13	Expressed in 3 stages non membrane	0.13	0.502
expressed in all 3 stages non membrane	0.13	Membrane expressed in 3 stages	0.2076	0.1194
expressed in all 3 stages membrane	0.2076	Asexual non membrane	0.289	0.1372
expressed in all 3 stages membrane	0.2076	Membrane asexual	0.3319	0.0823
expressed in all 3 stages membrane	0.2076	Male non membrane	0.3494	0.0531
expressed in all 3 stages membrane	0.2076	Membrane male	0.1865	0.5823
expressed in all 3 stages membrane	0.2076	Female non membrane	0.2152	0.4372
expressed in all 3 stages membrane	0.2076	Membrane female	0.1013	0.9403
expressed in all 3 stages membrane	0.2076	Expressed in 3 stages non membrane	0.13	0.8702
expressed in all 3 stages membrane	0.2076	Membrane expressed in 3 stages	0.2076	0.4968

Pf/Pr Table E: ds for all sets ds=0 included

Set	Number of genes	Mean ds	CI 95%	CI 95%
Membrane male	14	0.1170	0.0782	0.1672
Male non membrane	100	0.1043	0.0843	0.1264
Membrane female	8	0.0605	0.0402	0.0846
Female non membrane	44	0.0832	0.0677	0.1013
Membrane expressed in 3 stages	12	0.0890	0.0620	0.1250
Expressed in 3 stages non membrane	131	0.1535	0.1031	0.2209
Membrane asexual	23	0.0956	0.0673	0.1294
Asexual non membrane	73	0.0870	0.0711	0.1052

Pf/Pr Table F: ds comparisons for all sets

Set 1	Mean ds	Set 2	Mean ds	Pvalue Set 1 > Set 2
asexual non membrane	0.087	Asexual non membrane	0.087	0.502
asexual non membrane	0.087	Membrane asexual	0.0956	0.3603
asexual non membrane	0.087	Male non membrane	0.1043	0.146
asexual non membrane	0.087	Membrane male	0.117	0.149
asexual non membrane	0.087	Female non membrane	0.0832	0.6094
asexual non membrane	0.087	Membrane female	0.0605	0.936
asexual non membrane	0.087	Expressed in 3 stages non membrane	0.1535	0.0118
asexual non membrane	0.087	Membrane expressed in 3 stages	0.089	0.4821
asexual membrane	0.0956	Asexual non membrane	0.087	0.639
asexual membrane	0.0956	Membrane asexual	0.0956	0.5048
asexual membrane	0.0956	Male non membrane	0.1043	0.3359
asexual membrane	0.0956	Membrane male	0.117	0.2629
asexual membrane	0.0956	Female non membrane	0.0832	0.7124
asexual membrane	0.0956	Membrane female	0.0605	0.9412
asexual membrane	0.0956	Expressed in 3 stages non membrane	0.1535	0.0597

asexual membrane	0.0956	Membrane expressed in 3 stages	0.089	0.6013
male non membrane	0.1043	Asexual non membrane	0.087	0.8637
male non membrane	0.1043	Membrane asexual	0.0956	0.6558
male non membrane	0.1043	Male non membrane	0.1043	0.5019
male non membrane	0.1043	Membrane male	0.117	0.3679
male non membrane	0.1043	Female non membrane	0.0832	0.9065
male non membrane	0.1043	Membrane female	0.0605	0.9899
male non membrane	0.1043	Expressed in 3 stages non membrane	0.1535	0.0796
male non membrane	0.1043	Membrane expressed in 3 stages	0.089	0.7575
male membrane	0.117	Asexual non membrane	0.087	0.8538
male membrane	0.117	Membrane asexual	0.0956	0.7264
male membrane	0.117	Male non membrane	0.1043	0.6298
male membrane	0.117	Membrane male	0.117	0.4989
male membrane	0.117	Female non membrane	0.0832	0.8833
male membrane	0.117	Membrane female	0.0605	0.9783
male membrane	0.117	Expressed in 3 stages non membrane	0.1535	0.2138
male membrane	0.117	Membrane expressed in 3 stages	0.089	0.8009
female non membrane	0.0832	Asexual non membrane	0.087	0.3978
female non membrane	0.0832	Membrane asexual	0.0956	0.2949
female non membrane	0.0832	Male non membrane	0.1043	0.0967
female non membrane	0.0832	Membrane male	0.117	0.1144
female non membrane	0.0832	Female non membrane	0.0832	0.5025
female non membrane	0.0832	Membrane female	0.0605	0.9114
female non membrane	0.0832	Expressed in 3 stages non membrane	0.1535	0.0072
female non membrane	0.0832	Membrane expressed in 3 stages	0.089	0.4313
female membrane	0.0605	Asexual non membrane	0.087	0.068
female membrane	0.0605	Membrane asexual	0.0956	0.0574
female membrane	0.0605	Male non membrane	0.1043	0.011
female membrane	0.0605	Membrane male	0.117	0.0198
female membrane	0.0605	Female non membrane	0.0832	0.0916
female membrane	0.0605	Membrane female	0.0605	0.5062
female membrane	0.0605	Expressed in 3 stages non membrane	0.1535	0.0006
female membrane	0.0605	Membrane expressed in 3 stages	0.089	0.1062
expressed in all 3 stages non membrane	0.1535	Asexual non membrane	0.087	0.9877
expressed in all 3 stages non membrane	0.1535	Membrane asexual	0.0956	0.9377
expressed in all 3 stages non membrane	0.1535	Male non membrane	0.1043	0.9129

expressed in all 3 stages non membrane	0.1535	Membrane male	0.117	0.785
expressed in all 3 stages non membrane	0.1535	Female non membrane	0.0832	0.993
expressed in all 3 stages non membrane	0.1535	Membrane female	0.0605	0.9994
expressed in all 3 stages non membrane	0.1535	Expressed in 3 stages non membrane	0.1535	0.5056
expressed in all 3 stages non membrane	0.1535	Membrane expressed in 3 stages	0.089	0.9567
expressed in all 3 stages membrane	0.089	Asexual non membrane	0.087	0.5058
expressed in all 3 stages membrane	0.089	Membrane asexual	0.0956	0.3961
expressed in all 3 stages membrane	0.089	Male non membrane	0.1043	0.2474
expressed in all 3 stages membrane	0.089	Membrane male	0.117	0.2044
expressed in all 3 stages membrane	0.089	Female non membrane	0.0832	0.5729
expressed in all 3 stages membrane	0.089	Membrane female	0.0605	0.8939
expressed in all 3 stages membrane	0.089	Expressed in 3 stages non membrane	0.1535	0.0439
expressed in all 3 stages membrane	0.089	Membrane expressed in 3 stages	0.089	0.493

Pf/Pr Table G: dn for all sets

Set	Number of genes	Mean dn	CI 95%	CI 95%
Membrane male	14	0.029878571	0.0162	0.046571786
Male non membrane	100	0.036353	0.02968095	0.04322
Membrane female	8	0.0054125	0.0035375	0.00725
Female non membrane	44	0.019331818	0.013570114	0.025977614
Membrane expressed in 3 stages	12	0.018266667	0.007725	0.032591667
Expressed in 3 stages non membrane	131	0.019807634	0.014161832	0.026035382
Membrane asexual blood stages	23	0.034713043	0.023299348	0.047087391
Asexual blood stages non membrane	73	0.03459589	0.026402671	0.043398082

Pf/Pr Table H: dn comparisons for all sets

Set 1	Mean dn	Set 2	Mean dn	Pvalue Set 1 > Set 2
Asexual blood stages non membrane	0.0346	Asexual blood stages non membrane	0.0346	0.5027
Asexual blood stages non membrane	0.0346	Membrane asexual blood stages	0.0347	0.4977
Asexual blood stages non membrane	0.0346	Male non membrane	0.0364	0.3902
Asexual blood stages non membrane	0.0346	Membrane male	0.0299	0.6875
Asexual blood stages non membrane	0.0346	Female non membrane	0.0193	0.9919
Asexual blood stages non membrane	0.0346	Membrane female	0.0054	1
Asexual blood stages non membrane	0.0346	Expressed in 3 stages non membrane	0.0198	0.9922
Asexual blood stages non membrane	0.0346	Membrane expressed in 3 stages	0.0183	0.9543
Membrane asexual blood stages	0.0347	Asexual blood stages non membrane	0.0346	0.4948
Membrane asexual blood stages	0.0347	Membrane asexual blood stages	0.0347	0.5049
Membrane asexual blood stages	0.0347	Male non membrane	0.0364	0.4122
Membrane asexual blood stages	0.0347	Membrane male	0.0299	0.6679
Membrane asexual blood stages	0.0347	Female non membrane	0.0193	0.9759
Membrane asexual blood stages	0.0347	Membrane female	0.0054	1
Membrane asexual blood stages	0.0347	Expressed in 3 stages non membrane	0.0198	0.9739

Membrane asexual blood stages	0.0347	Membrane expressed in 3 stages	0.0183	0.9371
Male non membrane	0.0364	Asexual blood stages non membrane	0.0346	0.6107
Male non membrane	0.0364	Membrane asexual blood stages	0.0347	0.5865
Male non membrane	0.0364	Male non membrane	0.0364	0.5054
Male non membrane	0.0364	Membrane male	0.0299	0.7451
Male non membrane	0.0364	Female non membrane	0.0193	0.9987
Male non membrane	0.0364	Membrane female	0.0054	1
Male non membrane	0.0364	Expressed in 3 stages non membrane	0.0198	0.9986
Male non membrane	0.0364	Membrane expressed in 3 stages	0.0183	0.9691
Membrane male	0.0299	Asexual blood stages non membrane	0.0346	0.319
Membrane male	0.0299	Membrane asexual blood stages	0.0347	0.333
Membrane male	0.0299	Male non membrane	0.0364	0.2502
Membrane male	0.0299	Membrane male	0.0299	0.5041
Membrane male	0.0299	Female non membrane	0.0193	0.8585
Membrane male	0.0299	Membrane female	0.0054	1
Membrane male	0.0299	Expressed in 3 stages non membrane	0.0198	0.8457
Membrane male	0.0299	Membrane expressed in 3 stages	0.0183	0.8295
Female non membrane	0.0193	Asexual blood stages non membrane	0.0346	0.0078
Female non membrane	0.0193	Membrane asexual blood stages	0.0347	0.0229
Female non membrane	0.0193	Male non membrane	0.0364	0.0013
Female non membrane	0.0193	Membrane male	0.0299	0.1403
Female non membrane	0.0193	Female non membrane	0.0193	0.4963
Female non membrane	0.0193	Membrane female	0.0054	1
Female non membrane	0.0193	Expressed in 3 stages non membrane	0.0198	0.4678
Female non membrane	0.0193	Membrane expressed in 3 stages	0.0183	0.5753
Membrane female	0.0054	Asexual blood stages non membrane	0.0346	0
Membrane female	0.0054	Membrane asexual blood stages	0.0347	0
Membrane female	0.0054	Male non membrane	0.0364	0
Membrane female	0.0054	Membrane male	0.0299	0
Membrane female	0.0054	Female non membrane	0.0193	0.0001
Membrane female	0.0054	Membrane female	0.0054	0.5033
Membrane female	0.0054	Expressed in 3 stages non membrane	0.0198	0
Membrane female	0.0054	Membrane expressed in 3 stages	0.0183	0.0108
Expressed in 3 stages non membrane	0.0198	Asexual blood stages non membrane	0.0346	0.0092
Expressed in 3 stages non membrane	0.0198	Membrane asexual blood stages	0.0347	0.0293
Expressed in 3 stages non membrane	0.0198	Male non membrane	0.0364	0.0016
Expressed in 3 stages non membrane	0.0198	Membrane male	0.0299	0.1542

Expressed in 3 stages non membrane	0.0198	Female non membrane	0.0193	0.5331
Expressed in 3 stages non membrane	0.0198	Membrane female	0.0054	1
Expressed in 3 stages non membrane	0.0198	Expressed in 3 stages non membrane	0.0198	0.5037
Expressed in 3 stages non membrane	0.0198	Membrane expressed in 3 stages	0.0183	0.6002
Membrane expressed in 3 stages	0.0183	Asexual blood stages non membrane	0.0346	0.0542
Membrane expressed in 3 stages	0.0183	Membrane asexual blood stages	0.0347	0.067
Membrane expressed in 3 stages	0.0183	Male non membrane	0.0364	0.0322
Membrane expressed in 3 stages	0.0183	Membrane male	0.0299	0.1618
Membrane expressed in 3 stages	0.0183	Female non membrane	0.0193	0.4201
Membrane expressed in 3 stages	0.0183	Membrane female	0.0054	0.9865
Membrane expressed in 3 stages	0.0183	Expressed in 3 stages non membrane	0.0198	0.3969
Membrane expressed in 3 stages	0.0183	Membrane expressed in 3 stages	0.0183	0.4983
