# Supplementary material for: Why are male malaria parasites in such a rush? Sex-specific evolution and host–parasite interactions
Source: Evol Med Public Health. 2012 Nov 26;2013(1):3–13. doi: 10.1093/emph/eos003 (PMC4183958; doi:10.1093/emph/eos003)
Supplement: Supplementary Data [file supp_eos003_suppl_data.zip › REECE_Table_S2_PV.pdf]

Pv/Pk Table A: dn/ds for stage-specific sets

Set	Number of genes	Mean dn/ds	CI 95%	CI 95%
Male	213	0.0817	0.0738	0.0898
Female	97	0.0725	0.0632	0.0819
Expressed in 3 stages	293	0.0407	0.0368	0.0448
Asexual blood stages	158	0.0888	0.0791	0.0996

Pv/Pk Table B: dn/ds comparisons for stage-specific sets

Set 1	Mean dn/ds	Set 2	Mean dn/ds	Pvalue Set 1 > Set 2
Asexual blood stages	0.0888	Asexual blood stages	0.0888	0.5093
Asexual blood stages	0.0888	Male	0.0817	0.8197
Asexual blood stages	0.0888	Female	0.0725	0.9787
Asexual blood stages	0.0888	Expressed in 3 stages	0.0407	1
Male	0.0817	Asexual blood stages	0.0888	0.1896
Male	0.0817	Male	0.0817	0.4922
Male	0.0817	Female	0.0725	0.8907
Male	0.0817	Expressed in 3 stages	0.0407	1
Female	0.0725	Asexual blood stages	0.0888	0.0277
Female	0.0725	Male	0.0817	0.1086
Female	0.0725	Female	0.0725	0.5006
Female	0.0725	Expressed in 3 stages	0.0407	1
Expressed in 3 stages	0.0407	Asexual blood stages	0.0888	0
Expressed in 3 stages	0.0407	Male	0.0817	0
Expressed in 3 stages	0.0407	Female	0.0725	0
Expressed in 3 stages	0.0407	Expressed in 3 stages	0.0407	0.5013

Pv/Pk Table C: dn/ds for all sets

Set	Number of genes	Mean dn/ds	CI 95%	CI 95%
Membrane male	26	0.0709	0.0531	0.0898
Male non membrane	187	0.0832	0.0745	0.0923
Membrane female	23	0.1024	0.0805	0.1252
Female non membrane	74	0.0632	0.0542	0.0721
Membrane expressed in 3 stages	34	0.0556	0.0409	0.0728
Expressed in 3 stages non membrane	259	0.0387	0.0349	0.0427
Membrane asexual	34	0.1051	0.0810	0.1340
Asexual non membrane	124	0.0843	0.0739	0.0954

Pv/Pk Table D: dn/ds comparisons for all sets

Set 1	Mean dn/ds	Set 2	Mean dn/ds	Pvalue Set 1 > Set 2
asexual non membrane	0.0843	Asexual non membrane	0.0843	0.4992
asexual non membrane	0.0843	Membrane asexual	0.1051	0.1154
asexual non membrane	0.0843	Male non membrane	0.0832	0.5468
asexual non membrane	0.0843	Membrane male	0.0709	0.8447
asexual non membrane	0.0843	Female non membrane	0.0632	0.9937
asexual non membrane	0.0843	Membrane female	0.1024	0.1176
asexual non membrane	0.0843	Expressed in 3 stages non membrane	0.0387	1
asexual non membrane	0.0843	Membrane expressed in 3 stages	0.0556	0.9887
asexual membrane	0.1051	Asexual non membrane	0.0843	0.8936
asexual membrane	0.1051	Membrane asexual	0.1051	0.5007
asexual membrane	0.1051	Male non membrane	0.0832	0.9123
asexual membrane	0.1051	Membrane male	0.0709	0.9654
asexual membrane	0.1051	Female non membrane	0.0632	0.9992
asexual membrane	0.1051	Membrane female	0.1024	0.5429
asexual membrane	0.1051	Expressed in 3 stages non membrane	0.0387	1
asexual membrane	0.1051	Membrane expressed in 3 stages	0.0556	0.998

male non membrane	0.0832	Asexual non membrane	0.0843	0.4342
male non membrane	0.0832	Membrane asexual	0.1051	0.0891
male non membrane	0.0832	Male non membrane	0.0832	0.4943
male non membrane	0.0832	Membrane male	0.0709	0.8348
male non membrane	0.0832	Female non membrane	0.0632	0.995
male non membrane	0.0832	Membrane female	0.1024	0.0936
male non membrane	0.0832	Expressed in 3 stages non membrane	0.0387	1
male non membrane	0.0832	Membrane expressed in 3 stages	0.0556	0.9906
male membrane	0.0709	Asexual non membrane	0.0843	0.1606
male membrane	0.0709	Membrane asexual	0.1051	0.0321
male membrane	0.0709	Male non membrane	0.0832	0.1682
male membrane	0.0709	Membrane male	0.0709	0.4999
male membrane	0.0709	Female non membrane	0.0632	0.7174
male membrane	0.0709	Membrane female	0.1024	0.0373
male membrane	0.0709	Expressed in 3 stages non membrane	0.0387	0.9986
male membrane	0.0709	Membrane expressed in 3 stages	0.0556	0.8451
female non membrane	0.0632	Asexual non membrane	0.0843	0.0054
female non membrane	0.0632	Membrane asexual	0.1051	0.0011
female non membrane	0.0632	Male non membrane	0.0832	0.0049
female non membrane	0.0632	Membrane male	0.0709	0.2777
female non membrane	0.0632	Female non membrane	0.0632	0.4956
female non membrane	0.0632	Membrane female	0.1024	0.0022
female non membrane	0.0632	Expressed in 3 stages non membrane	0.0387	0.9999
female non membrane	0.0632	Membrane expressed in 3 stages	0.0556	0.7564
female non membrane	0.0632	Membrane all other sections	0.0888	0.0313
female membrane	0.1024	Asexual non membrane	0.0843	0.8826
female membrane	0.1024	Membrane asexual	0.1051	0.4633
female membrane	0.1024	Male non membrane	0.0832	0.9087
female membrane	0.1024	Membrane male	0.0709	0.9609
female membrane	0.1024	Female non membrane	0.0632	0.998
female membrane	0.1024	Membrane female	0.1024	0.4979
female membrane	0.1024	Expressed in 3 stages non membrane	0.0387	1
female membrane	0.1024	Membrane expressed in 3 stages	0.0556	0.9978
female membrane	0.1024	Membrane all other sections	0.0888	0.7683
expressed in all 3 stages non membrane	0.0387	Asexual non membrane	0.0843	0
expressed in all 3 stages non membrane	0.0387	Membrane asexual	0.1051	0
expressed in all 3 stages non membrane	0.0387	Male non membrane	0.0832	0

expressed in all 3 stages non membrane	0.0387	Membrane male	0.0709	0.0003
expressed in all 3 stages non membrane	0.0387	Female non membrane	0.0632	0
expressed in all 3 stages non membrane	0.0387	Membrane female	0.1024	0
expressed in all 3 stages non membrane	0.0387	Expressed in 3 stages non membrane	0.0387	0.4988
expressed in all 3 stages non membrane	0.0387	Membrane expressed in 3 stages	0.0556	0.0344
expressed in all 3 stages membrane	0.0556	Asexual non membrane	0.0843	0.011
expressed in all 3 stages membrane	0.0556	Membrane asexual	0.1051	0.003
expressed in all 3 stages membrane	0.0556	Male non membrane	0.0832	0.0119
expressed in all 3 stages membrane	0.0556	Membrane male	0.0709	0.1489
expressed in all 3 stages membrane	0.0556	Female non membrane	0.0632	0.2462
expressed in all 3 stages membrane	0.0556	Membrane female	0.1024	0.0022
expressed in all 3 stages membrane	0.0556	Expressed in 3 stages non membrane	0.0387	0.963
expressed in all 3 stages membrane	0.0556	Membrane expressed in 3 stages	0.0556	0.4981
expressed in all 3 stages membrane	0.0556	Membrane all other sections	0.0888	0.0239

Pv/Pk Table E: ds for all sets

Set	Number of genes	Mean ds	CI 95%	CI 95%
Membrane male	26	0.7119	0.5657	0.8840
Male non membrane	187	0.6315	0.5962	0.6689
Membrane female	23	0.5019	0.4266	0.5829
Female non membrane	74	0.6279	0.5684	0.6917
Membrane expressed in 3 stages	34	0.6964	0.6132	0.7802
Expressed in 3 stages non membrane	260	0.6839	0.6458	0.7230
Membrane asexual	34	0.6296	0.5544	0.7129
Asexual non membrane	124	0.6804	0.6276	0.7362

Pv/Pk Table F: ds comparisons for all sets

Set 1	Mean ds	Set 2	Mean ds	Pvalue Set 1 > Set 2
asexual non membrane	0.6804	Asexual non membrane	0.6804	0.4911
asexual non membrane	0.6804	Membrane asexual	0.6296	0.8055
asexual non membrane	0.6804	Male non membrane	0.6315	0.8891
asexual non membrane	0.6804	Membrane male	0.7118	0.4076
asexual non membrane	0.6804	Female non membrane	0.6279	0.8583
asexual non membrane	0.6804	Membrane female	0.5019	0.9986
asexual non membrane	0.6804	Expressed in 3 stages non membrane	0.6839	0.4703
asexual non membrane	0.6804	Membrane expressed in 3 stages	0.6964	0.4002
asexual non membrane	0.6804	All other sections non membrane	0.7051	0.2989
asexual non membrane	0.6804	Membrane all other sections	0.6411	0.7266
asexual membrane	0.6296	Asexual non membrane	0.6804	0.1947
asexual membrane	0.6296	Membrane asexual	0.6296	0.4997
asexual membrane	0.6296	Male non membrane	0.6315	0.4701
asexual membrane	0.6296	Membrane male	0.7118	0.2299
asexual membrane	0.6296	Female non membrane	0.6279	0.5195

asexual membrane	0.6296	Membrane female	0.5019	0.9671
asexual membrane	0.6296	Expressed in 3 stages non membrane	0.6839	0.1504
asexual membrane	0.6296	Membrane expressed in 3 stages	0.6964	0.1706
male non membrane	0.6315	Asexual non membrane	0.6804	0.1063
male non membrane	0.6315	Membrane asexual	0.6296	0.5275
male non membrane	0.6315	Male non membrane	0.6315	0.5032
male non membrane	0.6315	Membrane male	0.7118	0.213
male non membrane	0.6315	Female non membrane	0.6279	0.5417
male non membrane	0.6315	Membrane female	0.5019	0.9903
male non membrane	0.6315	Expressed in 3 stages non membrane	0.6839	0.0536
male non membrane	0.6315	Membrane expressed in 3 stages	0.6964	0.124
male membrane	0.7118	Asexual non membrane	0.6804	0.5987
male membrane	0.7118	Membrane asexual	0.6296	0.7665
male membrane	0.7118	Male non membrane	0.6315	0.7809
male membrane	0.7118	Membrane male	0.7118	0.4997
male membrane	0.7118	Female non membrane	0.6279	0.7842
male membrane	0.7118	Membrane female	0.5019	0.9837
male membrane	0.7118	Expressed in 3 stages non membrane	0.6839	0.586
male membrane	0.7118	Membrane expressed in 3 stages	0.6964	0.5332
female non membrane	0.6279	Asexual non membrane	0.6804	0.1375
female non membrane	0.6279	Membrane asexual	0.6296	0.4912
female non membrane	0.6279	Male non membrane	0.6315	0.4606
female non membrane	0.6279	Membrane male	0.7118	0.2095
female non membrane	0.6279	Female non membrane	0.6279	0.4986
female non membrane	0.6279	Membrane female	0.5019	0.9789
female non membrane	0.6279	Expressed in 3 stages non membrane	0.6839	0.0964
female non membrane	0.6279	Membrane expressed in 3 stages	0.6964	0.1485
female membrane	0.5019	Asexual non membrane	0.6804	0.0015
female membrane	0.5019	Membrane asexual	0.6296	0.0286
female membrane	0.5019	Male non membrane	0.6315	0.01
female membrane	0.5019	Membrane male	0.7118	0.0141
female membrane	0.5019	Female non membrane	0.6279	0.0181
female membrane	0.5019	Membrane female	0.5019	0.4973
female membrane	0.5019	Expressed in 3 stages non membrane	0.6839	0.0005
female membrane	0.5019	Membrane expressed in 3 stages	0.6964	0.0042

Pv/Pk Table G: dn for all sets

Set	Number of genes	Mean dn	CI 95%	CI 95%
Membrane male	26	0.038976923	0.030838462	0.047138654
Male non membrane	187	0.041840107	0.039012166	0.044685615
Membrane female	23	0.046569565	0.037656304	0.056278261
Female non membrane	74	0.034151351	0.03002027	0.038307095
Membrane expressed in 3 stages	34	0.03055	0.024805882	0.036397059
Expressed in 3 stages non membrane	260	0.022943077	0.020988827	0.025015
Asexual blood stages membrane	34	0.053288235	0.044999853	0.062226471
Asexual blood stages non membrane	124	0.043479032	0.039923347	0.047014556

Pv/Pk Table H: dn comparisons for all sets

Set 1	Mean dn	Set 2	Mean dn	Pvalue Set 1 > Set 2
Asexual blood stages non membrane	0.0435	Asexual blood stages non membrane	0.0435	0.4937
Asexual blood stages non membrane	0.0435	Asexual blood stage membrane	0.0533	0.0365
Asexual blood stages non membrane	0.0435	Male non membrane	0.0418	0.7162
Asexual blood stages non membrane	0.0435	Membrane male	0.039	0.7962
Asexual blood stages non membrane	0.0435	Female non membrane	0.0342	0.997
Asexual blood stages non membrane	0.0435	Membrane female	0.0466	0.3168
Asexual blood stages non membrane	0.0435	Expressed in 3 stages non membrane	0.0229	1
Asexual blood stages non membrane	0.0435	Membrane expressed in 3 stages	0.0306	0.9981
Asexual blood stages membrane	0.0533	Asexual blood stages non membrane	0.0435	0.9636
Asexual blood stages membrane	0.0533	Asexual blood stage membrane	0.0533	0.5044
Asexual blood stages membrane	0.0533	Male non membrane	0.0418	0.9866
Asexual blood stages membrane	0.0533	Membrane male	0.039	0.9779
Asexual blood stages membrane	0.0533	Female non membrane	0.0342	0.9998
Asexual blood stages membrane	0.0533	Membrane female	0.0466	0.8091
Asexual blood stages membrane	0.0533	Expressed in 3 stages non membrane	0.0229	1
Asexual blood stages membrane	0.0533	Membrane expressed in 3 stages	0.0306	1

Male non membrane	0.0418	Asexual blood stages non membrane	0.0435	0.2765
Male non membrane	0.0418	Asexual blood stage membrane	0.0533	0.0123
Male non membrane	0.0418	Male non membrane	0.0418	0.491
Male non membrane	0.0418	Membrane male	0.039	0.7115
Male non membrane	0.0418	Female non membrane	0.0342	0.9944
Male non membrane	0.0418	Membrane female	0.0466	0.2176
Male non membrane	0.0418	Expressed in 3 stages non membrane	0.0229	1
Male non membrane	0.0418	Membrane expressed in 3 stages	0.0306	0.9958
Membrane male	0.039	Asexual blood stages non membrane	0.0435	0.2012
Membrane male	0.039	Asexual blood stage membrane	0.0533	0.0218
Membrane male	0.039	Male non membrane	0.0418	0.291
Membrane male	0.039	Membrane male	0.039	0.4964
Membrane male	0.039	Female non membrane	0.0342	0.8055
Membrane male	0.039	Membrane female	0.0466	0.1569
Membrane male	0.039	Expressed in 3 stages non membrane	0.0229	0.9998
Membrane male	0.039	Membrane expressed in 3 stages	0.0306	0.9122
Female non membrane	0.0342	Asexual blood stages non membrane	0.0435	0.0026
Female non membrane	0.0342	Asexual blood stage membrane	0.0533	0.0001
Female non membrane	0.0342	Male non membrane	0.0418	0.0052
Female non membrane	0.0342	Membrane male	0.039	0.1936
Female non membrane	0.0342	Female non membrane	0.0342	0.5005
Female non membrane	0.0342	Membrane female	0.0466	0.0185
Female non membrane	0.0342	Expressed in 3 stages non membrane	0.0229	0.9999
Female non membrane	0.0342	Membrane expressed in 3 stages	0.0306	0.7974
Membrane female	0.0466	Asexual blood stages non membrane	0.0435	0.687
Membrane female	0.0466	Asexual blood stage membrane	0.0533	0.193
Membrane female	0.0466	Male non membrane	0.0418	0.7832
Membrane female	0.0466	Membrane male	0.039	0.8401
Membrane female	0.0466	Female non membrane	0.0342	0.9814
Membrane female	0.0466	Membrane female	0.0466	0.4996
Membrane female	0.0466	Expressed in 3 stages non membrane	0.0229	1
Membrane female	0.0466	Membrane expressed in 3 stages	0.0306	0.9933
Expressed in 3 stages non membrane	0.0229	Asexual blood stages non membrane	0.0435	0
Expressed in 3 stages non membrane	0.0229	Asexual blood stage membrane	0.0533	0
Expressed in 3 stages non membrane	0.0229	Male non membrane	0.0418	0
Expressed in 3 stages non membrane	0.0229	Membrane male	0.039	0.0005
Expressed in 3 stages non membrane	0.0229	Female non membrane	0.0342	0

Expressed in 3 stages non membrane	0.0229	Membrane female	0.0466	0
Expressed in 3 stages non membrane	0.0229	Expressed in 3 stages non membrane	0.0229	0.5056
Expressed in 3 stages non membrane	0.0229	Membrane expressed in 3 stages	0.0306	0.0188
Membrane expressed in 3 stages	0.0306	Asexual blood stages non membrane	0.0435	0.0007
Membrane expressed in 3 stages	0.0306	Asexual blood stage membrane	0.0533	0
Membrane expressed in 3 stages	0.0306	Male non membrane	0.0418	0.0025
Membrane expressed in 3 stages	0.0306	Membrane male	0.039	0.0793
Membrane expressed in 3 stages	0.0306	Female non membrane	0.0342	0.1972
Membrane expressed in 3 stages	0.0306	Membrane female	0.0466	0.0073
Membrane expressed in 3 stages	0.0306	Expressed in 3 stages non membrane	0.0229	0.9805
Membrane expressed in 3 stages	0.0306	Membrane expressed in 3 stages	0.0306	0.5003
