# Supplementary material for: Why are male malaria parasites in such a rush? Sex-specific evolution and host–parasite interactions
Source: Evol Med Public Health. 2012 Nov 26;2013(1):3–13. doi: 10.1093/emph/eos003 (PMC4183958; doi:10.1093/emph/eos003)
Supplement: Supplementary Data [file supp_eos003_suppl_data.zip › REECE_Table_S3.pdf]

Table A: Acumulation of nonsens mutations (stop-codons) during the evolution of P. berghei and P. yoelii

Gene	Organism	Genomic location	Description	Gene type
		**Male genes = 1/227 (~ 0.02%)**		
PBANKA_094240	*P. berghei str. ANKA*	PB_RP3938: 3,717 - 4,886 (-)	conserved hypothetical protein	protein coding
		**Female genes = 2/100 (2%)**		
PBANKA_071440	*P. berghei str. ANKA*	PB_RP1356: 1,026 - 1,726 (-)	conserved hypothetical protein	protein coding
PBANKA_103870	*P. berghei str. ANKA*	PB_RP2018: 50 - 9,284 (-)	conserved hypothetical protein	protein coding
		**Asexual genes = 5/167 (~ 3%)**		
PBANKA_135370	*P. berghei str. ANKA*	PB_RP3535: 3,091 - 5,349 (-)	conserved hypothetical protein	protein coding
PBANKA_101630	*P. berghei str. ANKA*	PB_PH5417: 1,275 - 1,724 (-)	proliferation-associated protein 2g4, putative	protein coding
PBANKA_071690	*P. berghei str. ANKA*	PB_RP2559: 741 - 2,830 (+)	conserved hypothetical protein	protein coding
PBANKA_121170	*P. berghei str. ANKA*	PB_RP0873: 2,122 - 3,924 (+)	conserved hypothetical protein	protein coding
PBANKA_141830	*P. berghei str. ANKA*	PB_RP0417: 3,669 - 7,487 (-)	rhoptry protein 2 putative	protein coding
		**Genes expressed in all 3 stages = 2/295 (~ 1.5%)**		
PBANKA_060430	*P. berghei str. ANKA*	PB_RP1262: 4,734 - 6,562 (-)	conserved hypothetical protein	protein coding
PBANKA_093770	*P. berghei str. ANKA*	PB_RP2272: 385 - 753 (+)	apicoplast ribosomal protein L36e precursor, putative	protein coding
